# Qualitative Dynamical Modelling Can Formally Explain Mesoderm Specification and Predict Novel Developmental Phenotypes

**DOI:** 10.1371/journal.pcbi.1005073

**Published:** 2016-09-06

**Authors:** Abibatou Mbodj, E. Hilary Gustafson, Lucia Ciglar, Guillaume Junion, Aitor Gonzalez, Charles Girardot, Laurent Perrin, Eileen E. M. Furlong, Denis Thieffry

**Affiliations:** 1 Aix-Marseille Université, Marseille, France; 2 INSERM, Marseille, France; 3 European Molecular Biology Laboratory (EMBL), Genome Biology Unit, Heidelberg, Germany; 4 Génétique Reproduction et Développement, INSERM, Clermont-Ferrand, France; 5 CNRS, Marseille, France; 6 Computational Systems Biology team, Institut de Biologie de l’Ecole Normale Supérieure (IBENS), INSERM, Ecole Normale Supérieure, PSL Research University, Paris, France; University of Virginia, UNITED STATES

## Abstract

Given the complexity of developmental networks, it is often difficult to predict the effect of genetic perturbations, even within coding genes. Regulatory factors generally have pleiotropic effects, exhibit partially redundant roles, and regulate highly interconnected pathways with ample cross-talk. Here, we delineate a logical model encompassing 48 components and 82 regulatory interactions involved in mesoderm specification during *Drosophila* development, thereby providing a formal integration of all available genetic information from the literature. The four main tissues derived from mesoderm correspond to alternative stable states. We demonstrate that the model can predict known mutant phenotypes and use it to systematically predict the effects of over 300 new, often non-intuitive, loss- and gain-of-function mutations, and combinations thereof. We further validated several novel predictions experimentally, thereby demonstrating the robustness of model. Logical modelling can thus contribute to formally explain and predict regulatory outcomes underlying cell fate decisions.

## Author Summary

We delineate a logical model encompassing 48 components and 82 regulatory interactions controlling mesoderm specification during Drosophila development, thereby integrating all major genetic processes underlying the formation of four mesodermal tissues. The model is based on in vivo genetic data, partly confirmed by functional genomic data.

Model simulations qualitatively recapitulate the expression of the main lineage markers of each mesodermal derivative, from developmental stage 8 to 10, for the wild type case, as well as for over twenty reported mutant genotypes.

We further use this model to systematically predict the effects of over 300 loss- and gain-of-function mutations, and combinations thereof. By generating specific mutant combinations, we validated several novel predictions experimentally demonstrating the robustness of model.

This modelling study is the first to tackle the regulatory network controlling the specification of mesoderm during Drosophila development, and more broadly deals with one of the most comprehensive developmental networks that have been modelled to date.

## Introduction

**Fig 1:**
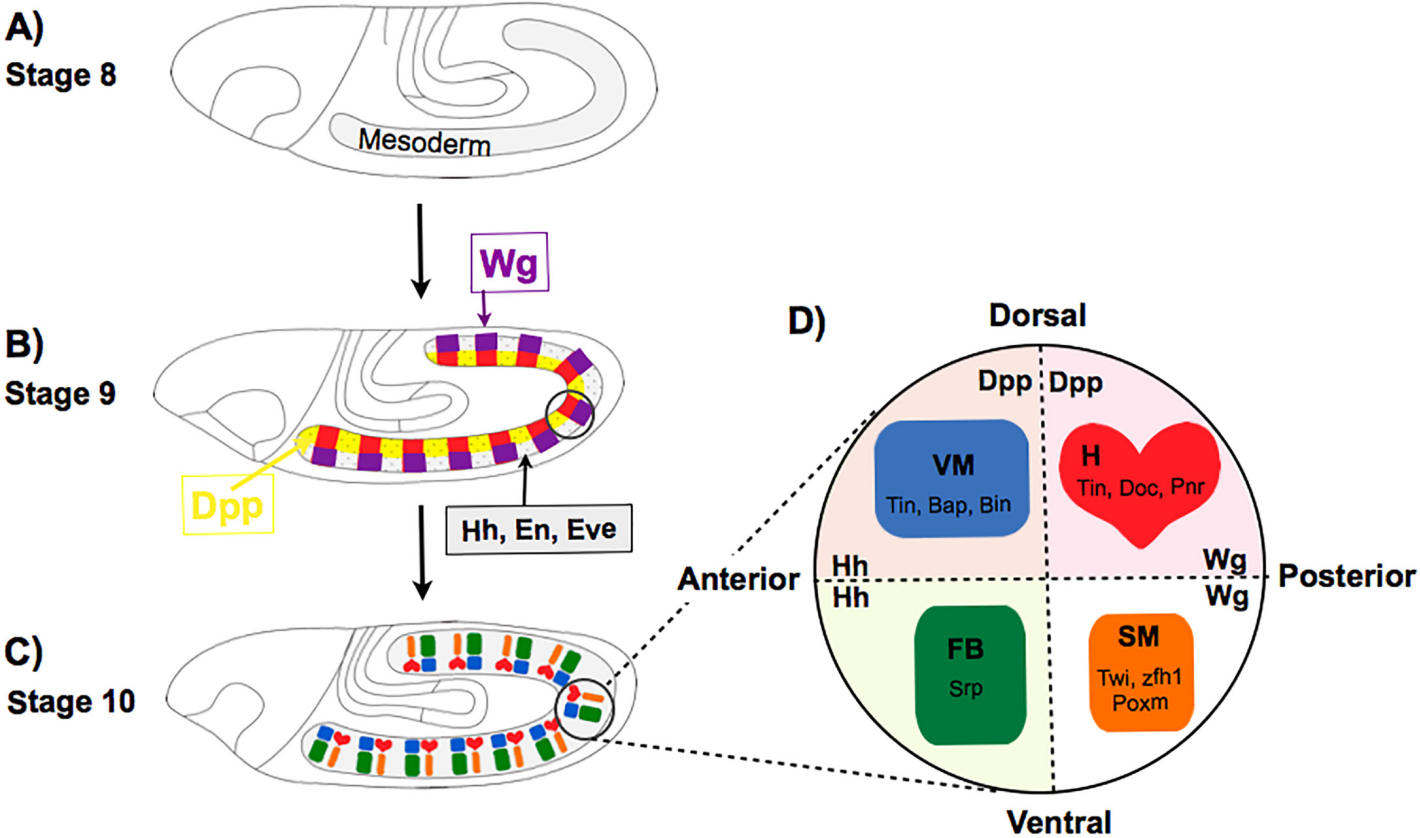
Early stages of drosophila mesoderm specification. **A-C:** Schematic description of the establishment of mesoderm anterior-posterior and dorsal-ventral patterning. At stage 8, the presumptive mesoderm is largely homogeneous. At stage 9, ectodermal signals outline a characteristic pattern, with stripes of Hh, Eve and En alternating with stripes of Wg and Slp, which delimit anterior/posterior segmental borders, respectively. Dpp signalling further delimits dorsal versus ventral mesoderm domains. Mesoderm specification is achieved at stage 10, when Wg/Slp domains give rise to heart precursors (H, in red, dorsally located) and somatic muscles (SM, orange, ventrally located), whereas En/Eve/Hh domains give rise to visceral mesoderm (VM, blue, dorsal) and fat body (FB, green, ventral). **D:** Schematic representation of the four main tissues originating from the mesoderm in each segment, with key associated markers (e.g. Srp expression for FB).

Functional genomic approaches (based on microarrays and next-generation sequencing) provide a powerful means to decipher the molecular mechanisms underlying the control of development and cell differentiation, as well as deregulations thereof associated with diseases such as cancer. Together with low-throughput experimental data, these high-throughput methods enable the delineation of large and sophisticate regulatory networks. Understanding and predicting the behaviour of such complex networks require the use of proper mathematical modelling frameworks. Various dynamical models have been proposed for a handful of relatively well known developmental processes, many using differential equations and referring to *Drosophila* development (see e.g. [1–5] and references therein). However, these modelling studies consider relatively limited numbers of regulatory components (at most a dozen) and require the quantitative determination of poorly documented parameters. In this context, formal qualitative modelling approaches constitute an interesting alternative, at least as a first step towards more quantitative modelling. In particular, logical (Boolean or multilevel) modelling has been applied to various regulatory and signalling networks of increasing sizes over the past decade (see e.g. [6–19] and references therein). But only few attempts were made to predict novel phenotypes, and therefore the full predictive value of the network and its usefulness to test hypotheses regarding novel genetic perturbations remain unclear. Here, we set out to decipher the network controlling the specification of mesoderm, one of the three germ-layers, into its four main derivatives, namely visceral muscle, heart, somatic muscle, and fat body, the primordia of which are iterated in segmentally repeated units along the anterior-posterior axis of the *Drosophila* embryo (Fig 1).

**Fig 2:**
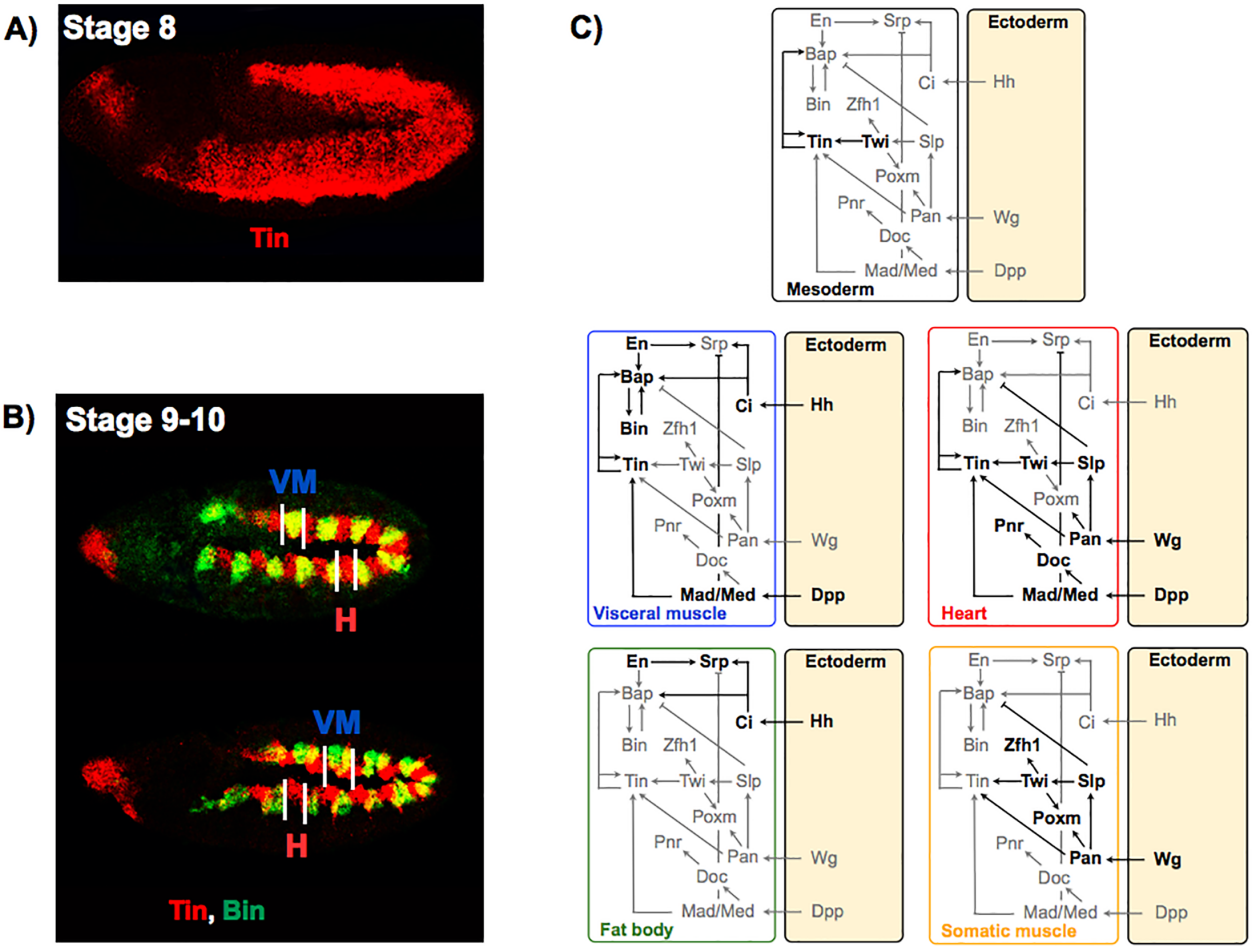
Key signalling pathways and markers genes involved in mesoderm specification. A, B: In situ hybridizations for Tin and Bin during mesoderm specification at stages 8 and 9–10. Tin is implicated in the formation of VM and H, while Bin participates only in the development of VM. Initially, the expression of Tin is mainly due to Twist activation. Later, Tin expression needs the presence of Dpp, Tin itself, in combination with Pan. C: Graphical representations of the main pathways activated by signals coming from the ectoderm, encompassing target transcription factors and cross-regulations underlying the specification of VM, H, FB and SM. In the absence of these factors, these tissues do not form or are severely reduced. Black and light grey arcs denote active and inactive regulations, depending on stage or tissue. Normal and blunt end arrows denote activations and inhibitions, respectively.

Mesoderm specification is induced by ectodermal signals such as Decapentaplegic (Dpp), which controls dorsal-ventral differentiation [20–25], Wingless (Wg), which is essential for dorsally located heart cell precursors and to the majority of somatic muscles that develop from more ventrally located cells [26–28], and Hedgehog (Hh), which specifies the visceral mesoderm dorsally and the fat body ventrally, itself characterised by the expression of Serpent (Srp) [29–31]. During embryonic stages 8–10, the mesoderm is thereby progressively specified into four different tissue primordia, each of which is characterised by the expression of specific lineage transcription factors (Fig 2) [27, 32].

Collating all phenotypic data from the literature into a mathematical model allows to formally assess the coherence between the current view of the network with individual published results on single or multiple mutant phenotypes. More specifically, we aim to further characterise the crucial regulatory components and interactions driving mesoderm specification. As we mostly rely on published qualitative molecular and genetic data, we use a flexible logical modelling framework and the software *GINsim* (cf. Material and methods), which enables the use of multilevel variables whenever justified, along with fully asynchronous updating. Systematic simulations of the resulting logical model were then performed to (i) assess the coherence and comprehensiveness of our representation of the underlying network, (ii) identify gaps in the current understanding and characterisation of mesoderm specification, and (iii) ultimately predict phenotypic outcomes of novel genetic perturbations. We demonstrate that the resulting logical model can recapitulate all known mutant phenotypes, therefore indicating that this formal representation of the network is sufficient and coherent to explain mesoderm cell fate decisions. By running simulations on over 300 genetic mutation combinations (many of which are double mutants with non-intuitive outcomes), the model could predict the phenotypic outcome for each novel mutant background, at least in terms of gene expression patterns, thereby providing new testable hypothesis that we experimentally confirmed. This approach thus provides developmental biologists with a very useful tool kit to test novel hypotheses, which are often very difficult to carry out experimentally. Moreover, the model provides novel insights into the underlying regulatory network driving these cell fate decisions.

## Results

### Establishment of a model for mesoderm specification

To initiate this study, we performed an extensive analysis of all reported genetic and molecular data in the literature to identify the main regulatory components involved in *Drosophila* mesoderm specification, along with the known interactions between them. Indeed, dozens of articles extensively cover the genetic bases of the sub-division of *Drosophila* mesoderm (this is evident by the bibliographical entries linked to key regulatory components in the model file and model documentation provided as S1 Text). *Cis*-regulatory information is sometimes available, enabling us to infer direct interactions and epistatic relations. In particular, we relied on recent ChIP data reporting the *in vivo* occupancy of six key mesoderm transcription factors (Bagpipe (Bap), Biniou (Bin), Dorsocross 1, 2 and 3 (Doc), Myocyte Enhancer Factor 2 (Mef2), Tinman (Tin) and Twist (Twi)) [33–35] to assess direct interactions inferred from genetic experiments.

**Fig 3:**
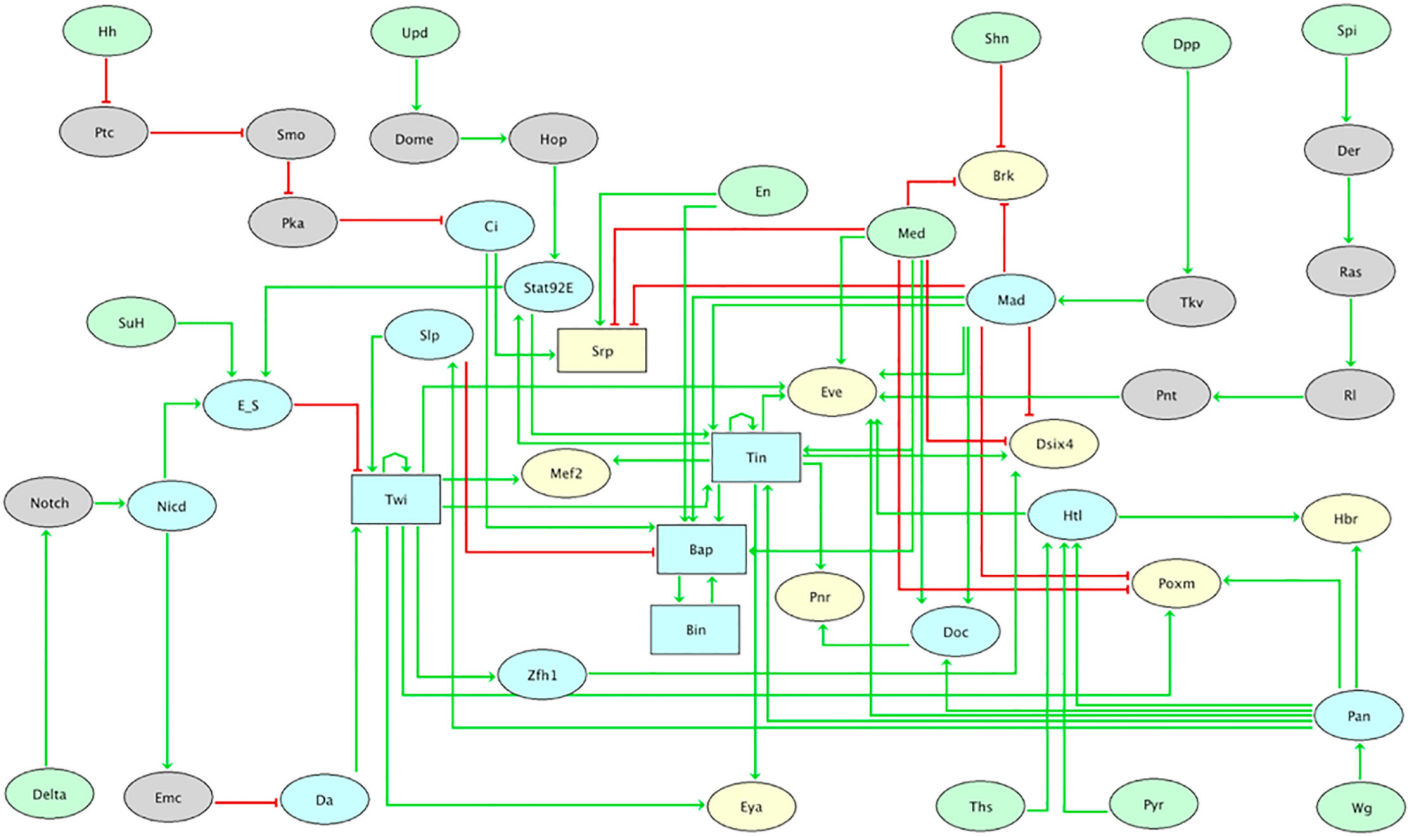
Regulatory graph for the signalling/transcriptional network controlling drosophila mesoderm specification. Built with the software GINsim, this regulatory graph encompasses the main regulatory factors and interactions involved in mesoderm specification (stages 8–10), as documented by published (molecular) genetic and functional genomic data. Ellipses denote Boolean nodes, whereas rectangles denote multilevel nodes. Light green filling denotes input nodes, most corresponding to factors expressed in and acting from the ectoderm. Yellow filling denotes output factors, mostly effector genes and tissue markers. Blue or grey filling denotes internal nodes expressed in the mesoderm. Green arrows and red blunt arrows denote activations and inhibitions, respectively. Logical rules are further associated with each node to define its behaviour depending on regulatory inputs (cf. S1 Table). To ease the dynamical analysis of this regulatory graph, we performed a reduction of this regulatory graph (cf. Material and methods), making implicit the twelve grey components. This logical model is provided as supporting data, including comprehensive annotations and bibliographical references.

Encoded using the software GINsim (Computational and experimental procedures), the resulting regulatory graph (Fig 3) is provided in a computer readable format, along with extensive annotations (text and links to relevant literature and database entries, see S1 File).

This regulatory graph encompasses 48 nodes (including 12 input components, representing mainly ectodermal signals) and 82 regulatory interactions. In many cases, the definition of the logical rule associated with each node is straightforward (e.g. when a node is the target of a unique regulator). However, for more complex regulatory relationships, i.e. when multiple interactions converge on the same component, we examine the following scenarios: (i) Is the presence of an inhibitor sufficient to completely or partially block gene expression? (ii) Which activator(s) is (are) sufficient to drive the expression of the target gene? (iii) Can the activators do so in the presence of repressor(s)? After several iterations, we obtained a set of logical rules consistent with all available knowledge on the regulation of each gene in the network, which further enabled the recapitulation of all published phenotypes (see below), demonstrating the robustness of the model.

### Delineation of tissue specific gene expression pattern

Before attempting to simulate the specification of the mesoderm into its four main presumptive tissues (visceral muscle (VM), heart (H), somatic muscle (SM), and the fat body (FB), we needed to specify the patterns of gene expression expected as a result of wild type development. Based on published data (mainly *in-situ* hybridization or immunostaining assays), we have derived the qualitative levels of expression of the 48 network components in each of the four presumptive territories (VM, H, SM and FB) from the literature (S1 Fig). Only subsets of these components are crucial in the specification of each of the four tissue subtypes. These tissue markers can be readily identified based on the phenotypes reported in loss-of-function mutant embryos, leading to severe defects in tissue formation, or following ectopic expression, often leading to specific tissue expansion. Embryos lacking Tin, Bap or Bin, for example, do not develop VM. Moreover, *tin* mutant embryos fail to develop H cells, and have severe defects in all tissues derived from the dorsal mesoderm [32, 36, 37]. Overall, ten network components play such dramatic roles in specific tissues (emphasised by bold contours for the corresponding coloured cells in the S1 Fig). Note that the ventral mesoderm territory that gives rise to both SM and FB is subdivided into regions that have low (yet significant, hence the use of the value 1) and high Twi expression. Indeed, the inhibition of Notch (N) combined with the presence of Wg and Daughterless (DA) activates Twi at a higher level (maximal level, i.e. value 2), thereby delimiting a region of high Twi expression [38, 39]. We systematically searched for relevant information and refined the logical rules until model behaviour was found fully consistent with all published data.

### Simulation settings

To ease simulations, our regulatory graph was reduced by hiding intermediate signalling components (components in grey in Fig 3, see also Material and methods). Provided that we do not delete any regulatory circuit, the resulting reduced model preserves the stable states of the system, which represent the different specification states (i.e. mesoderm derivatives for wild-type or mutant situations). To perform simulations, the initial values for each component must be specified, in particular for the signalling input components coming from the ectoderm. For each of the four presumptive tissue territories, we thus have a specific input combination (S1 Fig, left).

For the sake of simplicity, we set all internal nodes to zero for each wild-type initial state, with the notable exception of Twi, which was set to the value 1. For each territory, the target values at stage 10 were evaluated based on published data (S1 Fig, right). For example, in the region that will form VM, the initial state (stage 8) is characterised by the presence of Twi, which activates Tin [40] and Mef2 [41] expression. Bap is then activated by Tin at stage 9 [37], which is followed by the activation of Bin by Bap [42, 43] in late stage 9 embryos. Finally (stage 10), Bap is activated at its maximum level (value 3) by Cubitus Interruptus (Ci) and Engrailed (En) [27].

To recapitulate the formation of each mesodermal tissue derivative in the wild-type situation, we thus ran four different simulations using an asynchronous updating policy (Material and methods).

### The model can simulate the known regulatory events driving mesoderm specification

**Fig 4:**
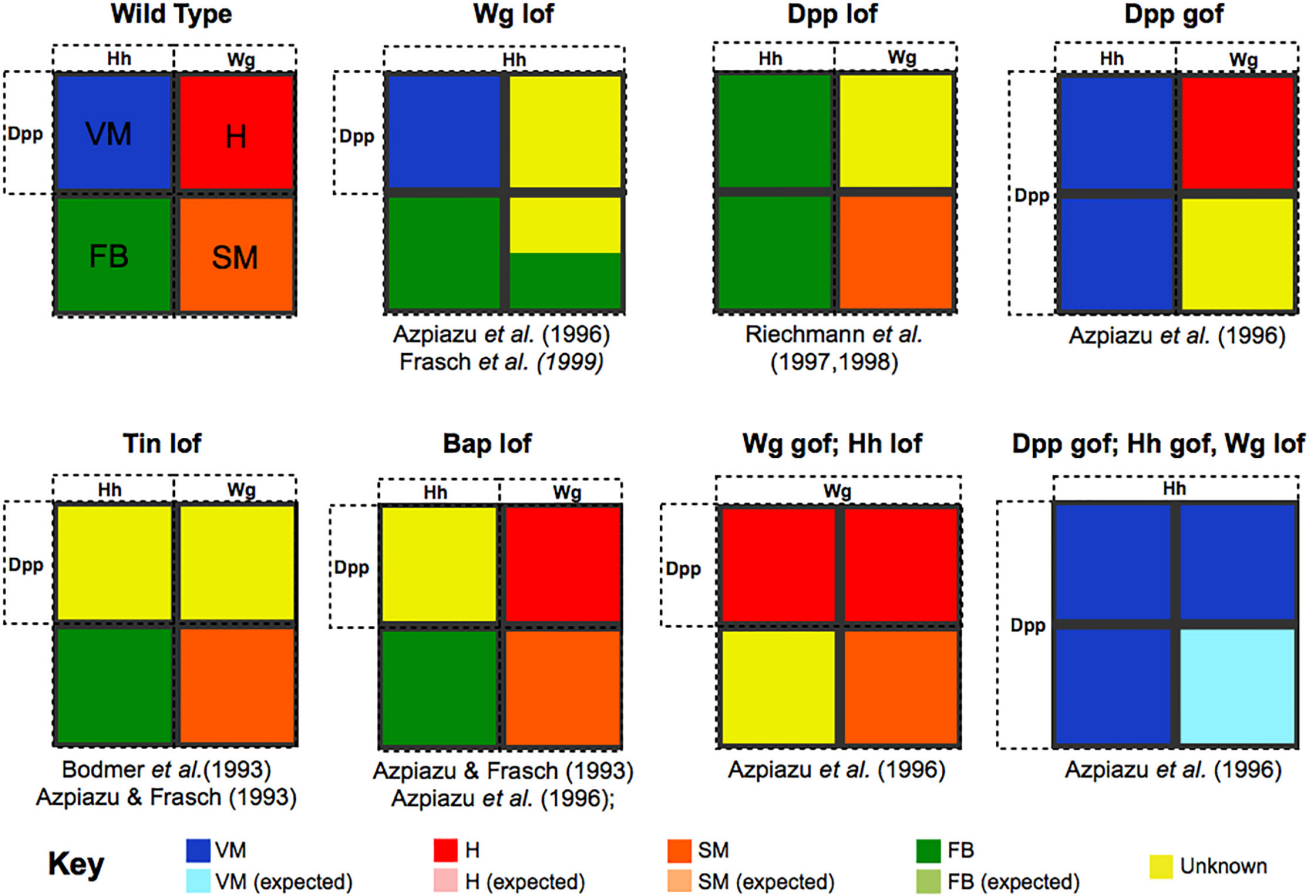
Simulations of known genetic perturbations. The results of selected simulations of loss-of-function (lof), gain-of-function (gof) mutations, and of combination thereof are shown in the form of coloured square vignettes, along with references to articles presenting matching data. The first vignette (top left) correspond to the wild type situation, with VM, H, FB and SM presumptive territories coloured in blue, red, green and orange, respectively. In the following vignettes, the coloration of the four presumptive territories are modified to reflect the absence or important markers, or the combination of markers associated with different tissues. Wg lof leads to the loss of Wg/Slp domain, resulting in an expansion of the En/Hh domain; consequently, the model correctly predicts the loss of H along with a potential perturbation of SM (yellow domains). Dpp lof leads to the loss of dorsal derivatives (VM and H), along with an expansion of FB. Dpp gof leads to an expansion of VM at the expense of FB, along with a perturbation of SM. Tin lof shows a loss of dorsal tissues, while Bap lof exhibits only the loss of VM. Finally, the combination of Wg gof and Hh lof leads to a dorsal expansion of H, along with a loss of FB, while the combination of Dpp gof, Hh gof and Wg lof leads to an expansion of VM in the whole mesoderm.

A detailed comparison of simulation results with experimental data led us to refine the logical rules, and sometimes even to consider additional regulators, until we converged on the regulatory graph shown in Fig 3, along with the rules listed in S1 Table (see also the S2 Text for more information about the delineation of the logical rules associated with Tin and Bap). Our final model qualitatively recapitulates all aspects of the major events in the specification of the four main domains of the mesoderm, from stage 8 to stage 10 (S2 Fig). In parallel, we also simulated the effects of genetic perturbations reported in the literature, the results of which led to some model adjustments. Iterating this procedure for all known mesodermal mutants led to a model that is robust and consistent with all relevant published data. The simulated phenotypes resulting from seven selected genetic perturbations are illustrated in Fig 4.

For example, the simulation of a *wg* loss-of- function (lof) gives rise to a loss of cardiac tissue, as observed experimentally [27, 44], while *dpp* lof gives rise to an extension of FB at the expenses of VM, mirroring previously reported experimental data [30, 32]. We can also simulate more complex genetic backgrounds. For example, a double gof of *dpp* and *hh* combined with a lof of *wg* leads to an expansion of VM in the entire mesoderm [27]. To date, our simulations recapitulate all mutants reported in the literature. Although expected, since this literature information was used as input to generate the model, these results demonstrate the coherence of the model, which was based on disjoint information generated from published studies from different labs, mostly based on single mutant phenotypes, with only a small number of documented multiple genetic perturbations. A study focused on heart or VM development, for example, often will not have examined markers for FB, yet the model can simulate the phenotypes in all four mesodermal domains.

### Logical modelling can predict phenotypes by simulating novel genotypes

Given the accuracy of our model to recapitulate all known published phenotypes, we reasoned that the model provides a very useful tool to perform systematic novel *in silico* perturbations at large scale. In fact, some of the results obtained with the simulations described above already correspond to new predictions, as biologists typically check only subsets of markers for each mutant studied (see in particular the tissue domains shown in yellow in Fig 4, which correspond to situations that have not been fully analysed experimentally, and for which we obtain combinations of markers associated with different tissues). Nevertheless, our aim here is to go beyond this and perform a more systematic assessment of the effects of combinations of two perturbations affecting different pathways and/or tissue markers. Single and multiple mutants can be readily defined using GINsim (cf. Material and methods), while they can often be very difficult, and sometimes impossible, to generate experimentally. To this end, we simulated the effects of single or double perturbations in each of the four tissue domains. The interpretation of the results generated is not trivial. To assess these results more efficiently, we used the expression of the key lineage transcription factors for each tissue as defining signature: for VM, expression of Tin (level 1), Bap (level 2), and Bin; for H, expression of Tin (level 2), Doc, and Pannier (Pnr); for FB, expression of Srp; and for SM, expression of Twi (level 2), Pox meso (Poxm), DSix4 and Zfh1. These tissue signatures were then matched against the stable states reached during model simulations, thereby automating the interpretation of the resulting phenotypes. Practical considerations (mutant strain availability) led us to consider fourteen components (Twi, Tin, Bap, Bin, Ci, Doc, Pan, Pnr, Slp, Srp, Mef2, Mad, Med, Nicd) for systematic single and pairwise combinations of loss- and gain-of function *in silico* perturbations.

**Fig 5:**
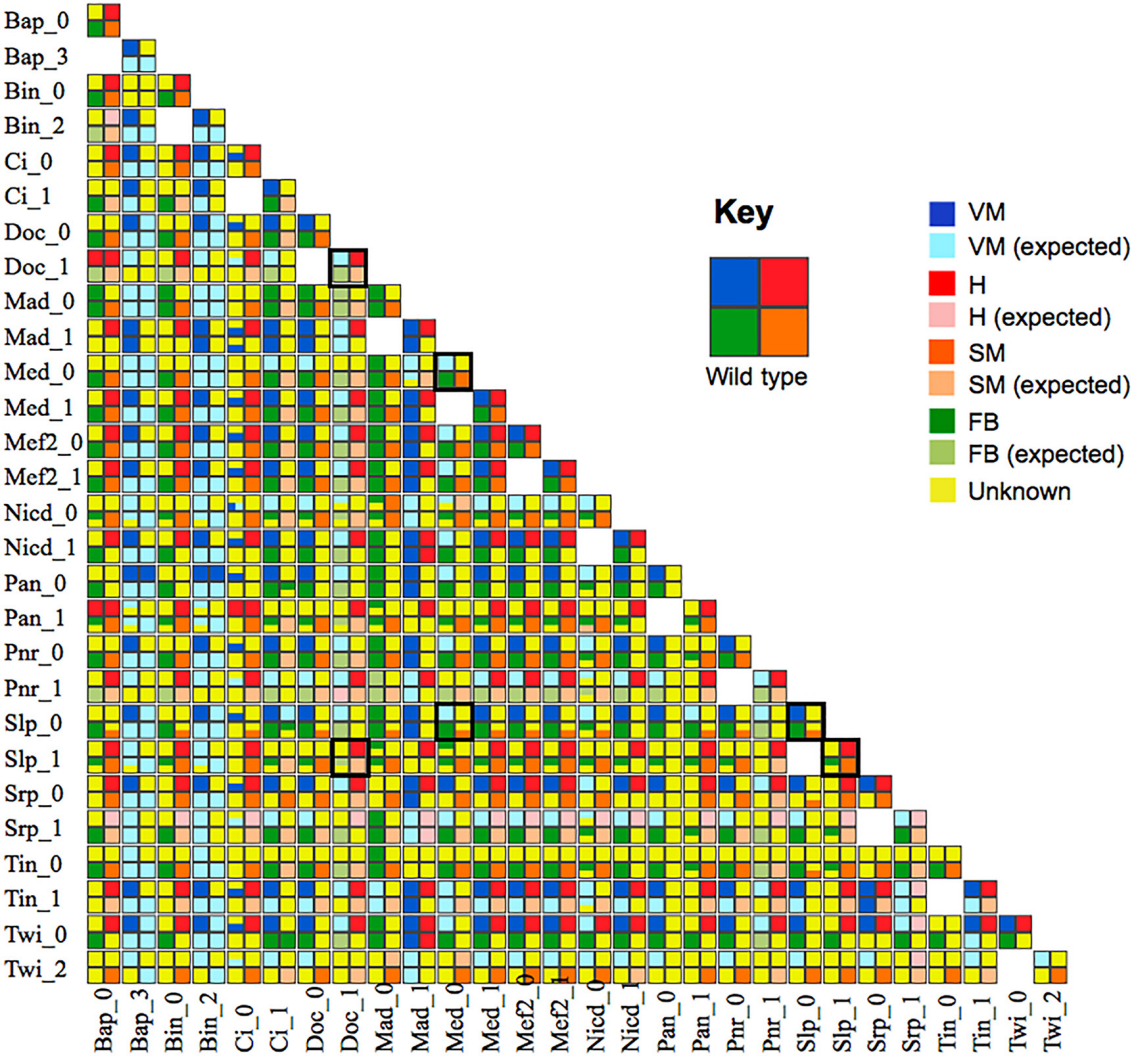
Systematic simulations of double mutants. This matrix displays the results of systematic perturbations. Loss- and gain-of-function mutations (rows and column) were simulated iteratively using a set of Python scripts, along with pairwise combinations (cf. Material and methods). The results of the simulations of single mutants are displayed on the diagonal of the matrix. The predicted phenotype for each double mutant is shown at the intersection of the corresponding column and row. Note that the cells corresponding to the crossing of a lof and a gof for the same gene are left empty. Simulation results are graphically depicted using vignettes as in Fig 4, with specific colours denoting situations with miss-expressed genes (cf. colour key top right). This presentation eases the comparison of the results of multiple mutant simulations and enables the identification of dominant or synergic effects. This matrix encompasses numerous predictions, along with a few dozens of documented phenotypes. The web version of the matrix (S2 File) further provides access to detailed information regarding the predicted patterns of expression for each mutant in each region. We have selected six perturbations (four single and two double ones, surrounded by tick squares in the matrix) for experimental validation (see Fig 6).

The results of the 338 mutant simulations performed are displayed in a matrix form (Fig 5) and can be browsed in a convenient searchable web archive (S2 File). This format enables an easy comparison of the effects of different perturbations, which facilitates the detection of dominant or synergic effects of different perturbations.

For example, *slp* lof generally shows a loss of H tissue, a result similar to that obtained for *wg* lof [26, 27]. Although some of these mutants have been partly documented experimentally, most of the double perturbations listed in Fig 5 have not been fully experimentally assessed in all four-tissue domains.

**Fig 6:**
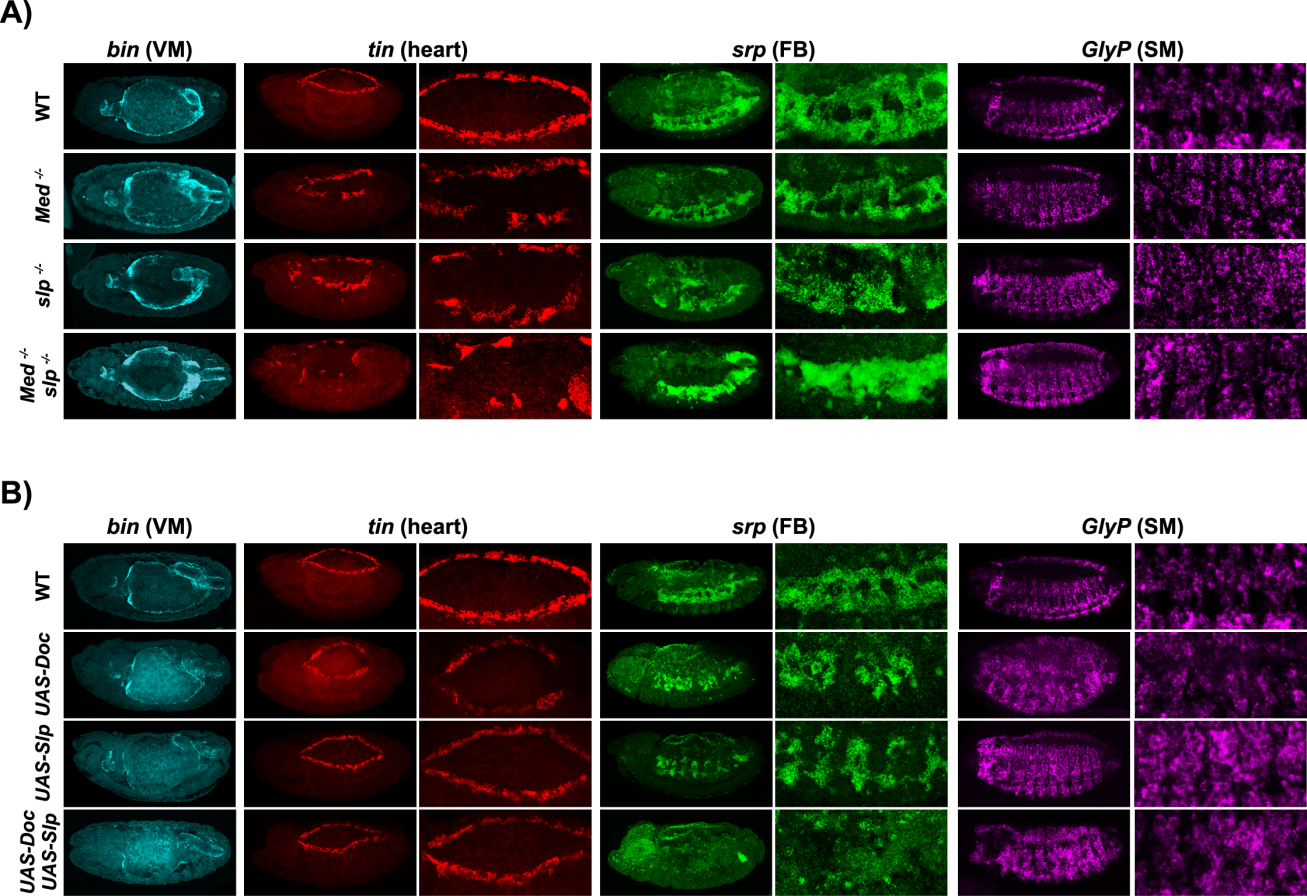
In situ RNA staining for two sets of single and double mutants. **A:** Slp lof and Med lof each results in a perturbation of H. The double mutant displays an even stronger disruption of H, along with a clear expansion of Srp expression. These experimental results are largely consistent with our model predictions and further provide interpretational clues regarding mixed expression patterns. **B:** Slp gof exhibits a loss of VM, while FB appears perturbed in both Doc gof and Slp gof mutants. The combination of these perturbations leads to stronger losses of FB and VM, while H and SM are barely affected. These results qualitatively agree with model predictions.

To demonstrate the usefulness and accuracy of these predictions, we experimentally tested six genetic perturbations (two double mutants and the associated four single mutants), examining the effects within all four tissue domains (Fig 6 and S3 Fig). Model predictions for each of these mutants are highlighted in Fig 5.

To examine the phenotype of each tissue, Tin, Srp, Glycogen phosphorylase (GlyP) and Bin were used as markers for the development of H, FB, SM and VM, respectively. We first assessed our predictions for lof mutants of Medea (Med) and Sloppy-paired (Slp), and the double mutant. Medea is directly required for the induction of *tinman* (Tin) by Dpp via the *tin-D* dorsal mesoderm enhancer [23]. Once expressed, Tin and Med have a direct protein-protein interaction that is required for dorsal mesoderm specification [28]. As the heart specification requires both activation by Med-Tin and repression of the VM within the heart domain by Slp, we were interested to examine if loss of Med and Slp would completely abolished cardiac mesoderm specification and be sufficient to extend the VM territory. For *Med* lof, our model simulations predict a loss of H, which is indeed what we observed experimentally (Fig 6A). Although the expression status of some genes within the VM region is changed in the mutant, the VM develops largely unperturbed, as predicted. The simulation of *slp* lof also results in a loss of H, and two stable states within the SM, one leading to normal SM development, while the other state lacks some marker expression, and therefore should perturb SM development. When we examine *slp* lof mutant experimentally, we observe the predicted loss of H, while SM appears largely normal, indicating that the corresponding stable state is the correct outcome. Simulations of the double lof mutant give the combined phenotype of the two single mutants, which again qualitatively fits with experimental data, with the H even more severely affected in the double mutant (Fig 6A, in situ for *tin* expression). In contrast, the VM develops largely unperturbed, indicating that loss of heart, even the severe disruption seen in the double mutant, is not sufficient to lead to expansion of VM, in this genetic background.

We next tested a combination of two gof conditions, where it is not a priori obvious what the phenotypic consequence would be within the FB or SM domains. Slp is normally expressed in the H region, where it inhibits VM development through the direct inhibition of Bap expression [32, 45]. Doc is expressed within the Heart domain (segmentally repeated patches of cells within the dorsal mesoderm) at stage 10, where it is essential for heart development [46, 47]. For a gof of Doc, our simulations predicts normal H development, with minor perturbations of VM, FB and SM. Our experimental results largely confirm these predictions, with very minor perturbations on the development of each tissue (based on the expression of the corresponding tissue markers), despite the ubiquitous expression of Doc (Fig 6B). The simulation of a gof of Slp predicts a severe perturbation of VM (yellow cell), characterised by the lack of expression of the key cell markers Bap (level 1 instead of 3) and Bin, as expected [32, 45], along with a potential perturbation of FB (obtention of two stable states, both with Srp expression). When the two gof genotypes are combined, our model predicts normal H and SM specification, but a loss of VM and FB, which is exactly what we observe experimentally, as seen in the *in situ* shown in Fig 6B. These results therefore demonstrate that our qualitative model can correctly predict the interaction between two gof causing a severe loss of FB. The expansion of heart cells can be further explained by the ectopic expression of heart markers in our simulations.

## Discussion

The logical model presented here integrates all major genetic processes underlying the formation of four tissues during *Drosophila* mesoderm specification. The model is based on the integration of extensive analysis of *in vivo* experimental data, especially genetic data (patterns of gene expression and mutant phenotypes), partly confirmed by functional genomic data (ChIP data for transcription factor occupancy). These data were translated mathematically in terms of a regulatory graph and logical rules. The simulation of our model qualitatively recapitulates the expression of the main lineage markers of each region from developmental stage 8 to 10, for the wild type case, as well as for over twenty reported mutant genotypes.

This study is the first attempt to model the regulatory network controlling the specification of mesoderm during *Drosophila* development, and more broadly represents one of the most comprehensive developmental networks that have been modelled to date. Mesoderm specification has been extensively studied in many species, including the sea urchin [48]. Recently, the Davidson group developed a Boolean model that recapitulates the specification of the sea urchin endo-mesoderm in the wild-type case, as well as experimental data for three genetic perturbations [49]. The approach of Davidson's group converge with ours in the delineation of a reference network with reliable annotations, which then serve as a scaffold to define logical rules and perform simulations. Both approaches implement the crucial components and interactions, along with the dynamical unfolding of the corresponding developmental network in an intuitive manner. Importantly, we demonstrate that we can not only recapitulate the known mutant phenotypes, but also predict various novel phenotypes.

In the case of our study, several regulatory mechanisms were simplified, in particular regarding the signalling pathways involved. We have developed more complete models of most *Drosophila* signalling pathways [50], but we retained simpler implementations of these pathways to keep our mesoderm specification model computationally tractable.

A limitation of this study resides in the poor documentation of specific markers associated with each type of embryonic domain. In particular, our marker set is limited to Srp in the case of FB. Presumably, others regulatory factors must be implicated in the specification of this tissue, which remain to be discovered. This lack of information complicates the interpretation of mutant phenotypes. For example, it is known that Bap lof leads to the loss of VM, but we miss information about effects on other tissues. Although Bap is crucial for VM development, it is also expressed at later stages in H. At this point, we assume that H, SM and FB develop normally in Bap lof mutant, as no other experimental defect has been reported.

Finally, Boolean models of embryonic processes generally rely on qualitative expression data from *in-situ* hybridisation. Our discrete model (as the sea urchin model [49]) is therefore limited to qualitative results, such as the presence or absence of a given tissue in a given presumptive territory. Although we cannot reproduce quantitative data, such as an increase or a decrease of specific cell numbers, we can still recapitulate the presence of different cell types. Our logical model could further serve as a scaffold to build more quantitative models when more quantitative and systematic experimental datasets will become available. For now, the advantage of logical modelling is that models can be easily abstracted at a level subsuming missing data, which is less straightforward for more quantitative modelling frameworks, such as differential or stochastic equations. Given the complexity of embryonic development, the shear number of parameters involved and the high inter-connected nature of regulatory networks, logical modelling offers an accurate solution that can be applied to many systems with the amount of data that is available today.

## Materials and Methods

### Multilevel logical formalism

We use the multilevel logical formalism, originally proposed by René Thomas [51], which has already been used to model various networks involved in the control of cell differentiation or proliferation (see e.g. [6, 8, 9, 11, 12, 19]. In short, both the structure of a logical model and its dynamics are represented in terms of graphs (in the sense of the graph theory), called regulatory graphs and state transition graphs, which are briefly described hereafter.

### Regulatory graphs

In a regulatory graph, the vertices (or nodes) represent regulatory genes or products (transcription factors, kinases, etc.). In many cases, these regulatory components can be satisfactorily represented by Boolean variables, which can take only two values, 0 or 1, corresponding to the absence or presence of the component, respectively. However, in some situations (e.g. the consideration of a morphogen), more qualitatively different levels may be required. The arcs (or arrows) connecting pairs of vertices represent regulatory interactions between components (e.g. transcriptional activations or inhibitions, phosphorylation, etc.). These arcs are usually associated with a plus (+) or minus (-) sign, denoting an activation or inhibition effect of the source node onto the target node, respectively. When the source of an arc is associated with a multilevel variable, a threshold (i.e. minimal level) must be specified. To complete this model description, logical rules (or logical parameters) are further defined to indicate how each component reacts to different combinations of regulatory interactions (S2 Text and S1 Table).

### State transition graphs

The simulation of a logical model can be represented by a state transition graph (STG), whose vertices represent logical states (i.e. a vector encompassing values for all components), whereas arcs represent transitions between states enabled by the corresponding regulatory graph and logical rules. In this work, we use an asynchronous updating mode, meaning that we consider all possible unitary transitions (affecting only one variable at a time) whenever there is a call to change some component value(s) at a given state. One recurrent problem with logical simulations (in particular when using asynchronous updating) is the potential combinatory explosion of the STG when dealing with large regulatory graphs. Consequently, it is often difficult to generate and analyse the STG for complex networks encompassing several dozens of components. However, using proper algorithms and software tools (see below), it is possible to characterise the asymptotical behaviour of the systems, which is of special interest for us here. Indeed, attractors, especially stable states (states with no successor), are usually associated with specific differentiated states. Logical models provide a realistic description of cellular events, as they are capable of reproducing time dependent processes in a qualitative manner (i.e. focusing on the sequential order of transitions).

### Logical modelling software *GINsim*

The software GINsim (for Gene Interaction Network simulation) implements the logical formalism [52]. It allows the edition, analysis and simulation of regulatory graphs. Freely available (http://ginsim.org), GINsim supports the annotation of components and interactions with free text and URLs. Once a model is defined, the user can select a simulation mode and define a set of initial states. GINsim can then be used to compute state transition graphs and report the stable states. GINsim also enables the definition and the simulation of different types of mutants (loss-of-function, ectopic gene expression, and combinations thereof) by blocking the levels of expression of the corresponding variables in defined intervals. To further ease the analysis of multiple perturbations, we have written a set of scripts in python, which iteratively compute the behaviour of our mesoderm specification model for each region and mutant considered, process the results and generate a synthetic web page (cf. Results and S2 File).

### Model reduction

To enable the dynamical analysis of comprehensive regulatory graphs, we take advantage of a novel reduction method implemented in GINsim. This functionality allows the user to select components of a regulatory graph to be made implicit. The software verifies that the proposed reduction does not fundamentally change the network topology (elimination of regulatory circuits) and update the logical rules for the components targeted by reduced nodes. The original and reduced networks have the same stable states (in terms of levels of common variables), while differences may appear as to their reachability [53].

### Fly stocks

The following *Drosophila* lines were used: UAS-Slp and UAS-Doc lines were kindly provided by M. Frasch (Doc line C2 [46]). We crossed both stocks with a marked double balancer to generate the homozygous stock x/y;UAS-Doc;UAS-Slp. Males from the UAS-Doc, UAS-Slp and UAS-Doc;UAS-Slp lines were crossed with females carrying a homozygous twist-GAL4 driver, kindly provided by Maria Leptin. Slp^1^ and Med^1e^ loss-of-function mutations were obtained from the Bloomington stock centre (stock numbers 5349 and 9033), and crossed together to make the double loss-of-function stock, which were placed over lacZ-marked balancers.

### *In situ* hybridization of mutant embryos

Embryos were collected using standard procedures. Fluorescent *in situ* hybridisation was performed as described previously [54]. The following ESTs were used to generate anti-sense probes: RE01329 (*tin*), SD07261 (*srp*), and LD24485 (*Glyp*), while a full length cDNA was used for *bin* (gift from M. Frasch) and *lacZ*. The probes were detected with peroxidase-conjugated antibodies (Roche) and developed using the TSA system (Perkin Elmer). *slp and Med* mutant embryos were unambiguously identified based on the absence of *lacZ* expression from the balancer chromosome.

## Supporting Information

S1 Fig This supporting fig displays discretised expression levels for inputs signals (from stage 8 to stage 10) and mesodermal genes (at stage 10), which serve as a reference to fix initial states and interpret final states in model simulations. (TIF)

S2 Fig This supporting fig provides list the mains steps of the dynamics of gene expression during mesoderm specification, from developmental stages 8 to 10, for the four mesoderm presumptive territories. (TIF)

S3 Fig This supporting fig shows additional representative embryos for the mutant genotypes shown in Fig 6A. Med and slp loss-of-function mutant embryos and the double mutant (Med-Slp) have defects in heart development (marked by tin expression, red), with the double mutant being more severe. The visceral muscle (VM, marked by bin) develops normally.
(TIF)

S1 Text This supporting text (in pdf format) provide textual annotations plus hyperlinks to entries to external database for each node of the model. It also includes information about ChIP data supporting transcriptional regulations of various nodes by master mesoderm transcriptional factors.
(PDF)

S2 Text This supporting text (in pdf format) provides further details about the experimental evidence at the basis of the delineation of the number of distinct levels and of the logical rules associated with Tin and Bap. (PDF)

S1 Table This supporting table (in pdf format) lists the logical rules associated with each node of the model (but the input nodes, for which the initial values are maintained in the absence of external perturbation). (PDF)

S1 File Model. This supporting file contains the full Drosophila mesoderm specification model, which can be opened and simulated using the software GINsim (GINsim is freely available from http://ginsim.org).
(ZGINML)

S2 File Web Archive. The content of this supporting web archive folder documents known and novel gene expression pattern simulated with the drosophila mesoderm specification. Open the file “index.html” with a web browser to access this information.
(ZIP)
