# Supplementary figures and images for: Qualitative Dynamical Modelling Can Formally Explain Mesoderm Specification and Predict Novel Developmental Phenotypes
Source: PLoS Comput Biol. 2016 Sep 6;12(9):e1005073. doi: 10.1371/journal.pcbi.1005073 (PMC5012701; doi:10.1371/journal.pcbi.1005073)

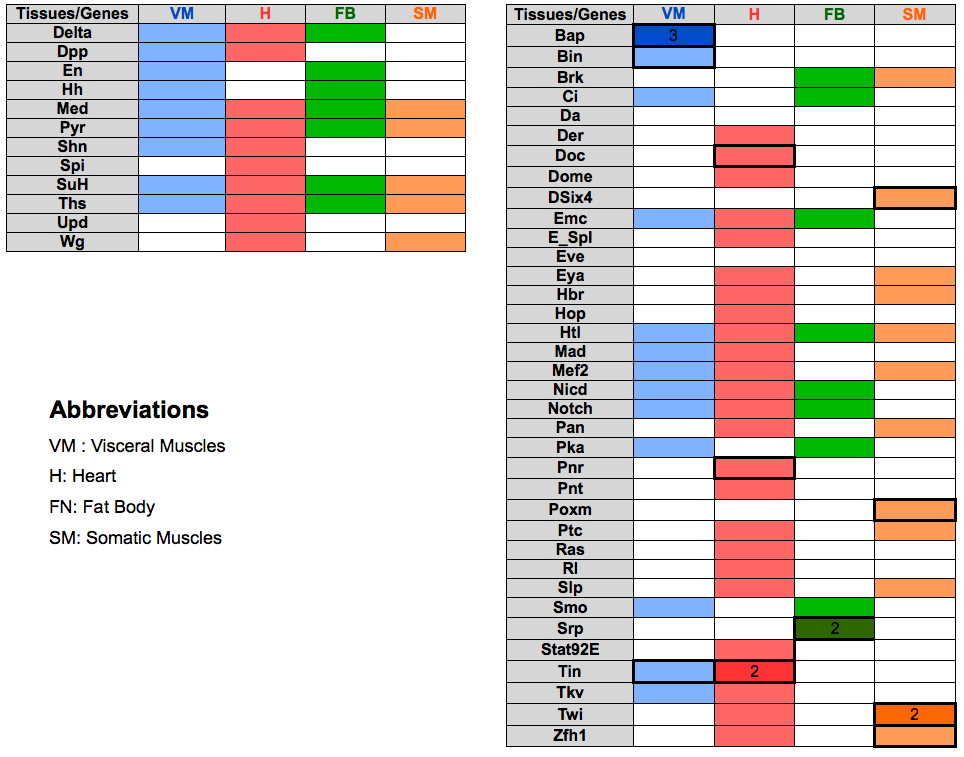

Supplement: S1 Fig — (TIF) [file pcbi.1005073.s001.tif]

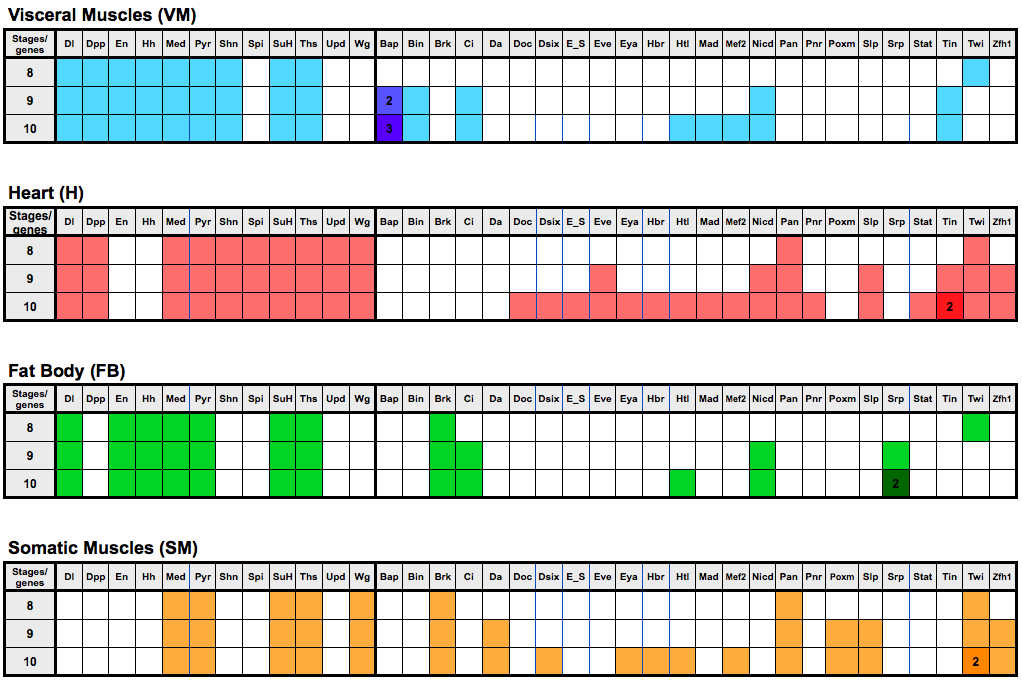

Supplement: S2 Fig — (TIF) [file pcbi.1005073.s002.tif]

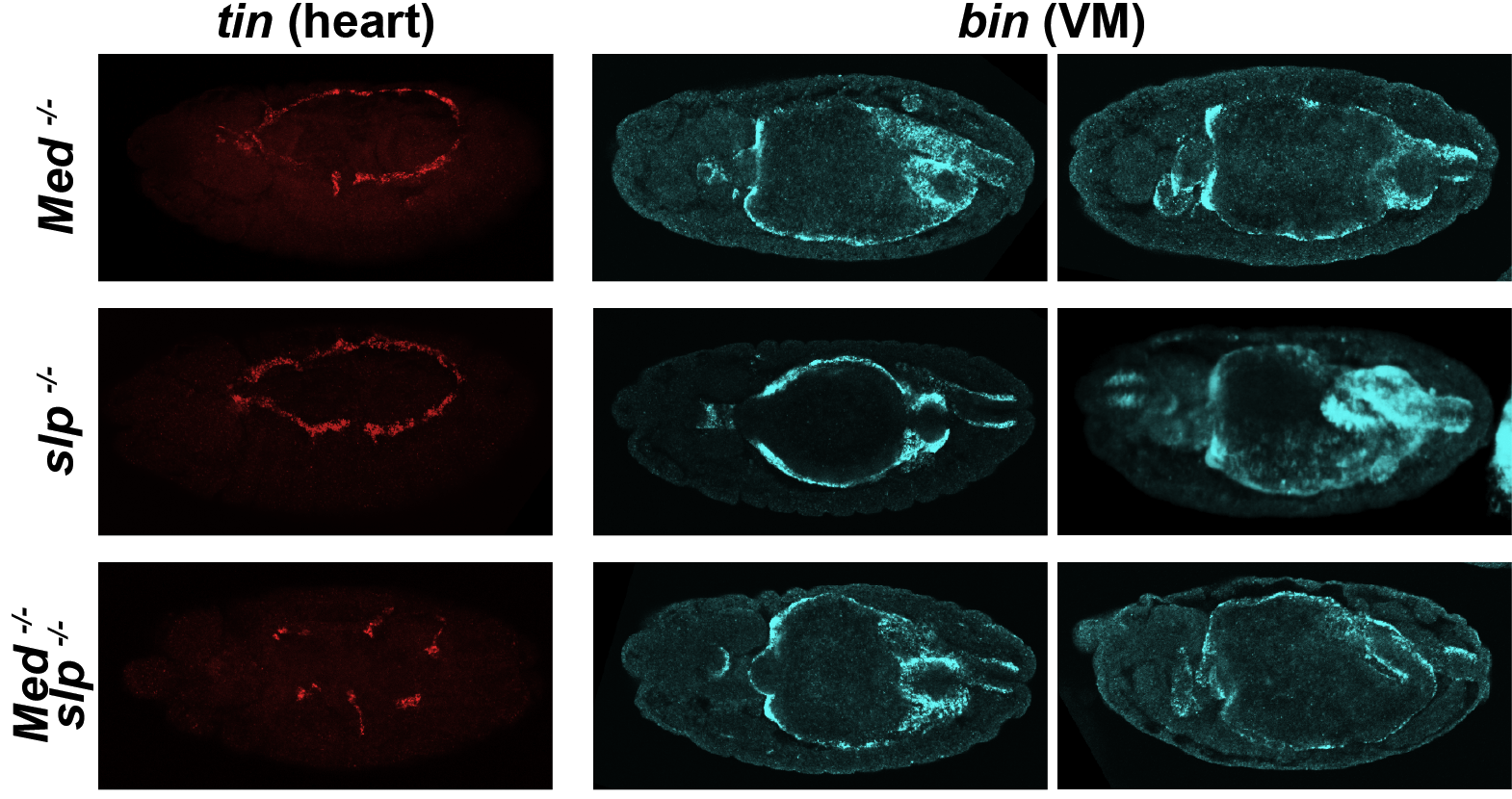

Supplement: S3 Fig — Med and slp loss-of-function mutant embryos and the double mutant (Med-Slp) have defects in heart development (marked by tin expression, red), with the double mutant being more severe. The visceral muscle (VM, marked by bin) develops normally. (TIF) [file pcbi.1005073.s003.tif]

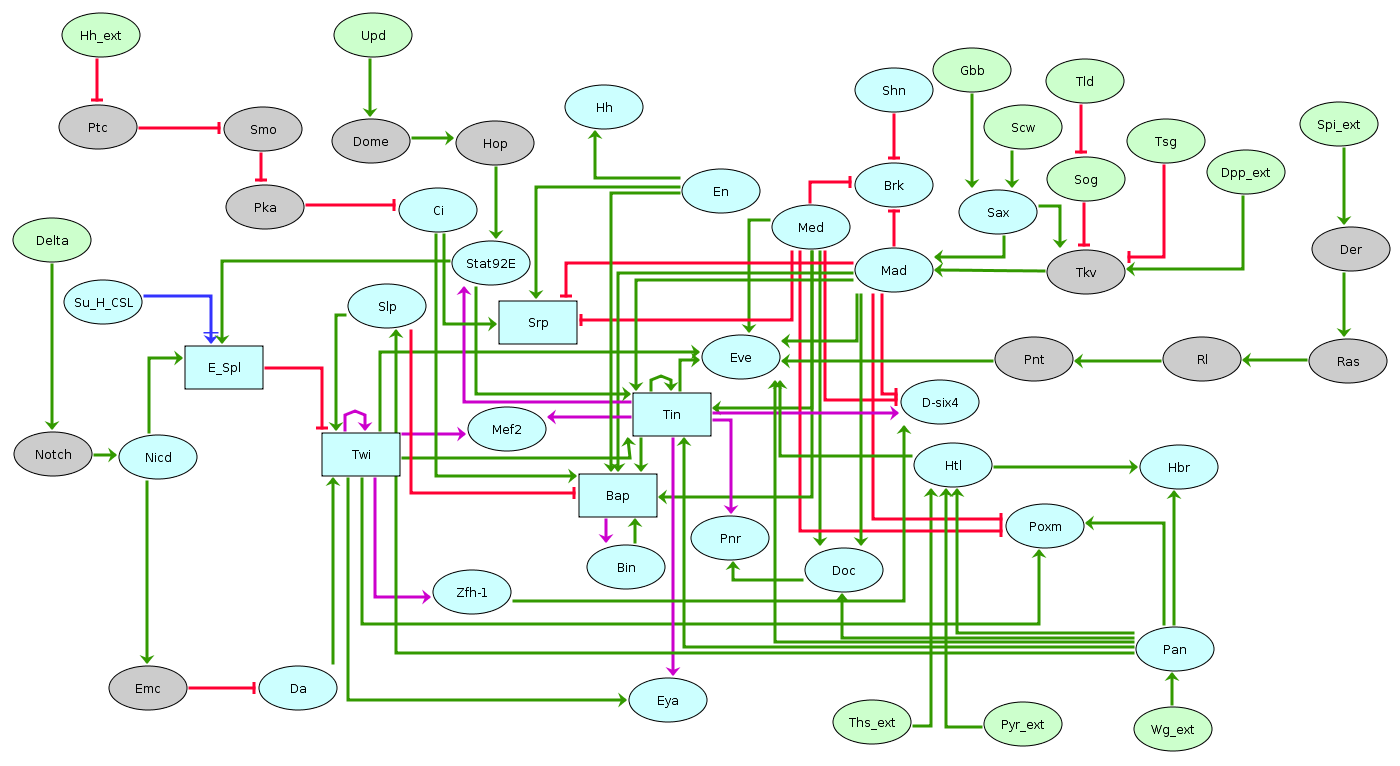

Supplement: S2 File — The content of this supporting web archive folder documents known and novel gene expression pattern simulated with the drosophila mesoderm specification. Open the file “index.html” with a web browser to access this information. (ZIP) [file pcbi.1005073.s008.zip › SupWebArchive_W1/model.png]
