# Supplementary material for: Qualitative Dynamical Modelling Can Formally Explain Mesoderm Specification and Predict Novel Developmental Phenotypes
Source: PLoS Comput Biol. 2016 Sep 6;12(9):e1005073. doi: 10.1371/journal.pcbi.1005073 (PMC5012701; doi:10.1371/journal.pcbi.1005073)
Supplement: S1 Text — It also includes information about ChIP data supporting transcriptional regulations of various nodes by master mesoderm transcriptional factors. (PDF) [file pcbi.1005073.s004.pdf]

Description of the model "DrosoMeso03Dec2015"

This logical model encompasses the main transcription factors involved in mesoderm specification during stages 8 to 10 of Drosophila development.
It encompasses 48 components and 82 regulatory interactions, providing a formal integration of diverse genetic information and an intuitive platform to visualise the complexity of the system.
This model is sufficient to explain known phenotypes from genetic mutants and to predict novel mutant phenotypes.
More specifically, the model accounts for the specification of the mesoderm into its four main presumptive tissue domains: visceral muscle (VM), heart (H), somatic muscle (SM), and fat body (FB). These different domains correspond to alternative stable states, which are defined by specific combinations of transcription factors and positional clues from signalling cascades.
Ellipses denote Boolean nodes, whereas square boxes denote multilevel nodes.
Light green nodes represent factors expressed in and acting from the ectoderm. Blue and grey nodes denote factors expressed and acting in the mesoderm.
Green arrows denote activation, whereas red blunt end arrows denote inhibitions.
This model has been defined and analysed using the software GINsim (http://ginsim.org).
To each regulatory node is associated a logical rule defining its behaviour depending on regulatory inputs.

Nodes

Node	Annotation and links
**Delta**	Delta is expressed throughout the mesoderm at late stage 9 and stage 10.<br>Delta is a ligand for the Notch signaling pathway (Kooh et al.,1993).<br>• PMID:8330521<br>• http://flybase.org/reports/FBgn0000463.html<br>• http://www.uniprot.org/uniprot/P10041<br>• http://www.sdbonline.org/fly/neural/delta.htm
**Dpp**	Decapentaplegic (Dpp) is required in the specification of dorsal derivatives.<br>In the model, Dpp is provided as an initial self-sustained input, which represents the presence of external Dpp signal.<br>Dpp regulates its target genes through two mechanisms: directly by activating gene expression and indirectly by Shn-dependent repression of Brk (Yao et al., 2008).<br>• PMID:18506030<br>• http://flybase.org/reports/FBgn0000490.html<br>• http://www.sdbonline.org/sites/fly/torstoll/decapen1.htm<br>• http://www.uniprot.org/uniprot/P07713
**En**	Engrailed (En) is expressed early due to the expression of even-skipped, which activates En. En promotes the development of VM and dorsolateral FB (Azpiazu et al., 1996; Reichmann et al., 1998).<br>• PMID:8985186<br>• PMID:9435291<br>• http://flybase.org/reports/FBgn0000577.html<br>• http://www.uniprot.org/uniprot/P02836<br>• http://www.sdbonline.org/fly/segment/engrail1.htm
**Hh**	Hedgehog (Hh) is a segment polarity gene (Nusslein- Volhard and Wieschaus, 1980; Lee et al., 1992) encoding a 47-kD protein that undergoes an intramolecular cleavage to yield a mature, N-terminal signaling polypeptide.<br>Binding of Hh to Patched (Ptc) initiates pathway response (Michelson et al., 2003).<br>• PMID:6776413

- PMID:1394430
- PMID:12881613
- http://flybase.org/reports/FBgn0004644.html
- http://www.sdbonline.org/fly/segment/hedghog1.htm
- http://www.uniprot.org/uniprot/Q02936

Med Mothers against dpp (Mad) and Medea (Med) transduce Dpp signal during Mesoderm specification (from stage 8 to 10 or 11) Dpp signaling is initiated by binding of the ligand to a complex of the type I and type II serine/threonine kinase receptors, including Tkv. Activated Tkv phosphorylates the BMP specific Mad, leading to its association with the co-Smad Med and accumulation of the Mad/Med complex in the nucleus.
Mad and Med-binding sites have been found in the promoters of many Dpp responsive genes (Xu et al., 1998 and Yao et al., 2008).

- PMID:9694800
- PMID:18506030
- http://flybase.org/reports/FBgn0011655.html
- http://www.sdbonline.org/fly/torstoll/medea1.htm
- http://www.uniprot.org/uniprot/P42003

Pyr Thisbe (Ths) and Pyramus (Pyr), encode FGF signaling molecules that appear to function in a partially redundant fashion to activate Htl.
They are required for the development of different mesoderm lineages, including the neurogenic ectoderm (early mesoderm spreading), muscle precursors (dorsal muscles, visceral muscles, and heart), hindgut (visceral musculature), and neuroblasts (Stathopoulos et al., 2004).

- PMID:15075295
- http://www.sdbonline.org/fly/sturtevant/pyramusthisbe1.htm
- http://flybase.org/reports/FBgn0033649.html

Shn Shnurri (Shn) codes for a large protein containing eight zinc fingers (Arora et al.,1995).
Shn is required to repress brk transcription upon Dpp signaling (Muller et al.,2003).
This repression is mediated by a Shn/Mad/Med complex that antagonises transcriptional activation (Yao et al., 2008).

- PMID:7774017
- PMID:12705870
- PMID:18506030
- http://flybase.org/reports/FBgn0003396.html
- http://www.sdbonline.org/sites/fly/gene/schnurri.htm
- http://www.uniprot.org/uniprot/Q0E9C5
- http://www.uniprot.org/uniprot/A1Z8F4
- http://www.uniprot.org/uniprot/A0A0B4KFC0

Spi Spitz (Spi) is a transforming growth factor responsible for DER activation in most tissues.
Spitz is produced as an inactive membrane precursor and is ubiquitously expressed.
Even when expressed at high levels, the precursor form is inactive.
Once reaching the Golgi, Spitz encounters Rhomboid, a seven-transmembrane domain protein that is essential for Spitz cleavage and activation (Shiloh et al., 2003).

- PMID:12648473
- http://flybase.org/reports/FBgn0005672.html
- http://www.sdbonline.org/fly/torstoll/spitz.htm
- http://www.uniprot.org/uniprot/Q01083

SuH Suppressor of Hairless (Su(H)) modulates Twist levels, leading to the low and high Twist characteristic pattern of wild-type embryos at stage 10.
Pan-mesodermal expression of a constitutively transactivating Su(H) form Su(H)-VP16 leads to expanded high Twist domains.
Notch requires Su(H) to repress Twist. In Notch mutant embryos, Twist phenotype results

from the loss of a transcriptional switch that converts Su(H) from a constitutive repressor into an activator (Tapanes-Castillo et al., 2004).

- PMID:15128668
- http://flybase.org/reports/FBgn0004837.html
- http://www.uniprot.org/uniprot/P28159
- http://www.sdbonline.org/fly/neural/suphair.htm

Ths Thisbe (Ths) and Pyramus (Pyr), encode FGF signaling molecules that appear to function in a partially redundant fashion to activate Htl.
They are required for the development of different mesoderm lineages, including the neurogenic ectoderm (early mesoderm spreading), muscle precursors (dorsal muscles, visceral muscles, and heart), hindgut (visceral musculature), and neuroblasts (Stathopoulos et al., 2004).

- PMID:15075295
- http://www.sdbonline.org/fly/sturtevant/pyramusthisbe1.htm
- http://flybase.org/reports/FBgn0033652.html

Upd Unpaired (UPD) is a secreted glycoprotein released by heparin (Harrison et al, 1998).
Drosophila JAK/STAT pathway activity is mediated exclusively by the ligands UPD, UPD2 and UPD3 (Harrison et al, 1998).
Upd and Upd2 have overlapping segmental expression patterns during mesoderm development (Hombria et al, 2005).

- PMID:9784499
- PMID:16277982
- http://flybase.org/reports/FBgn0004956.html
- http://flybase.org/reports/FBgn0030904.html
- http://flybase.org/reports/FBgn0053542.html
- http://www.sdbonline.org/sites/fly/dbzhnsky/outstrh1.htm
- http://www.uniprot.org/uniprot/Q9VWW4

Wg Wingless (Wg) input is provided as an initial self-sustained conditions, which represent the presence of external Wg signal.
Wg expression is limited to the anterior embryonic segment domains.
Wg is required for the development of H and SM (Azpiazu et al., 1996 ; Frasch et al., 1999).

- PMID:8985186
- PMID:10355030
- http://www.sdbonline.org/fly/segment/wingles1.htm
- http://flybase.org/reports/FBgn0004009.html
- http://www.uniprot.org/uniprot/P09615

Bap The main area for Bagpipe (Bap) expresion is the dorsal mesoderm.
Bap is required for VM specification, as no VM is formed in bap loss-of-function mutants (Azpiazu et al., 1993).
Wg represses Bap in the H domain via Slp.
Slp and Bap are expressed in mutually exclusive domains in the dorsal mesoderm (Azpiazu et al.,1996 and Reichmann et al.,1997).

- PMID:8985186
- PMID:9247334
- http://www.sdbonline.org/fly/gene/tinman.htm
- http://flybase.org/reports/FBgn0004862.html
- http://www.uniprot.org/uniprot/P22809

Bin Biniou (Bin) is expressed in progenitors of all three types of visceral musculature (from stage 10 to late 11; Zaffran et al., 2001).
Bin requires the combined activities of bap and dpp for its normal activation.
Bin is required for VM specification.

- PMID:11691840
- http://flybase.org/reports/FBgn0045759.html
- http://www.sdbonline.org/fly/gene/biniou1.htm
- http://www.uniprot.org/uniprot/Q9VS05

Regulator	Comment
Bap	Interaction strengthened by CHIP-Seq experiment: Bap binds to a predicted enhancer near Bin gene at stage 8 to 10 (Data from Furlong's lab)<br>Details about the CRM.<br>MesoCRM-4011<br>Chr: 3L<br>Start: 6978420<br>Stop: 6978620<br>Determined TSS:<br>TSS2 (6983721) found for the transcript FBtr0076900 and the CRM is found downstream the gene.

Brk Brinker (Brk) plays an essential role in the regulation of most Dpp targets.
Brk binds to the enhancers of Dpp target genes and functions as a constitutive repressor (Yao et al., 2008).

- PMID:18506030
- http://www.sdbonline.org/fly/torstoll/brinkr1.htm
- http://flybase.org/reports/FBgn0024250.html
- http://www.uniprot.org/uniprot/Q9XTN4

Ci Cubitus interruptus (Ci) is the downstream transcription factor of Hh signalling (Hosono et al., 2003).
Ci exists in an activatory and an inhbitory forms.
Th node Ci here represents the activatory form.
Hh signalling prmotes promotes the development of VM and FB via Ci (Azpiazu et al., 1996 ; Reichmann et al.,1998).

- PMID::8985186
- PMID:9435291
- http://www.sdbonline.org/fly/segment/cubitus.htm
- http://flybase.org/reports/FBgn0004859.html
- http://www.uniprot.org/uniprot/P52172

Da Daughterless (Da) is ubiquitously expressed throughout development and required to maintain uniform high Twist expression throughout the mesoderm during gastrulation (Cronmiller et al.,1993).
Loss of Da in early embryos reduces Twist expression, indicating that Da is required for high levels of Twist (Castanon et al., 2001).

- PMID:11688563
- PMID:8217842
- http://www.sdbonline.org/fly/gene/dauterls.htm
- http://flybase.org/reports/FBgn0000413.html
- http://www.uniprot.org/uniprot/P11420

Dsix4 Drosophila Six 4 (Dsix4) is a key factor for the development of H and SM (Yvan et al., 2006).

- PMID:16595131
- http://flybase.org/reports/FBgn0027364.html
- http://www.sdbonline.org/fly/genebrief/six4.htm

Regulator	Comment
Tin	Interaction strengthened by CHIP-Seq experiment: Tin binds to a predicted enhancer near Six4 gene at stage 8 to 10 (Data from Furlong's lab).

Details about the CRM:
Mesoderm CRM-4997
Chr: 3L
Start: 20782427
Stop: 20782860
Determined TSS:
TSS2 (20785454) found for the transcript FBtr0078275 and the CRM is found within the third intron.

Dome Domeless (Dome) is the receptor of the JAK/STAT pathway (Brown and Hombria, 2001).
- PMID:11696329
- http://flybase.org/reports/FBgn0043903.html
- http://www.sdbonline.org/sites/fly/dbzhnsky/domeles1.htm

Der Deranged (Der), is the Drosophila epidermal growth factor (EGF) receptor.
The presence of four activating ligands (Spitz, Keren, Gurken and Vein) and one inhibitory ligand allows versatile combinations of DER activation (Shiloh et al.,1991 and 2003).
- PMID:1820687
- PMID:12648473
- http://flybase.org/reports/FBgn0003731.html
- http://www.sdbonline.org/fly/torstoll/egf-r1.htm

Doc T-box factors Dorsocross (Doc 1, 2 and 3) are co-expressed in the dorsal mesoderm (Reim et al., 2003 and Tao et al., 2007).
In the model, they are encoded in a single node (Doc).
Doc is expressed in H at stages 10-11 at the intersection of Dpp and Wg signalling instructions.
In doc mutant embryo, Pnr expression is abolished (Tao et al., 2007).
- PMID:12783790
- PMID:17208472
- http://flybase.org/reports/FBgn0028789.html
- http://flybase.org/reports/FBgn0035956.html
- http://flybase.org/reports/FBgn0035954.html
- http://www.sdbonline.org/fly/sturtevant/droscro1.htm
- http://www.uniprot.org/uniprot/D6X1T1

Emc In the embryonic mesoderm, Emc is expressed uniformly during gastrulation until stage 10 (Ellis et al.,1990 and Garell et al.,1990).
Emc has been shown to genetically and biochemically interact with Da, inhibiting its activity (Tapanas-Castillo et al., 2004).
- PMID:1690604
- PMID:1690605
- PMID:15128668
- http://www.sdbonline.org/fly/gene/emc.htm
- http://flybase.org/reports/FBgn0000575.html
- http://www.uniprot.org/uniprot/P18491

E_S Su(H)-mediated Notch signaling represses Twist expression during subdivision and thus plays a critical role in patterning mesodermal segments.
Notch acts as a transcriptional switch on mesodermal target genes.
Notch signaling targets two distinct 'Repressors of twist' - the proteins encoded by the Enhancer of split complex [E(spl)C] and the HLH gene extra machrochaetae (emc) (Tapanes-Castillo and Baylies, 2004).
- http://www.sdbonline.org/sites/fly/neural/enhsplit.htm
- http://www.ncbi.nlm.nih.gov.gate1.inist.fr/pubmed/15128668

Eve Even-skipped (Eve) is a transcriptional repressor.

Eve expression depends on dpp, wg, tin, and slp.
During the onset of its expression, it is coexpressed with Tin and Slp in mesodermal cells (Knirr et al., 2001; Liu et al., 2008).
Tin and Dpp-activated Smads appear to synergize in allowing eve induction (Knirr et al., 2001).
In eve loss-of-function mutants, the mesoderm is unsegmented in the trunk region (Azpiazu et al.,1996).
No Eve positive H cells can be detected in Tin loss-of-function (Bordmer et al., 1993).

- PMID:11783990
- PMID:18067885
- PMID:8985186
- PMID:7915669
- http://www.sdbonline.org/fly/segment/evenskp1.htm
- http://flybase.org/reports/FBgn0000606.html
- http://www.uniprot.org/uniprot/P06602

Eya Eyes absent (Eya) is required for muscle development (Liu et al., 2009; Busser at al., 2012)

- PMID:19217429
- PMID:22412381
- http://www.uniprot.org/uniprot/Q05201
- http://flybase.org/reports/FBgn0000320.html
- http://www.sdbonline.org/fly/newgene/eyesab1.htm

Regulator	Comment
Tin	Interaction strengthened by CHIP-Seq experiment: Tin binds to predicted enhancers near Eya gene at stage 8 to 10 (Data from Furlong's lab).<br>Details about CRMs:<br>Mesoderm CRM-5586,<br>Chr: 2L:<br>start: 6531029<br>stop: 6531421<br>Determined TSSs:<br>TSS1 (6530509) found for the transcript FBtr0306113 and the CRM is found uptream the gene (ensembl gene prediction).<br>TSS2 (6536111) found for the transcript FBtr0079335 and the CRM is found within the second intron.<br>Dorsal Mesoderm CRM-5556<br>Chr: 2L<br>Start: 6530922<br>Stop: 6531337<br>Determined TSSs:<br>TSS1 (6530509) found for the transcript FBtr0306113 and the CRM is found uptream the gene (ensembl gene prediction).<br>TSS2 (6536111) found for the transcript FBtr0079335 and the CRM is found within the second intron.<br>Dorsal Mesoderm CRM-5557<br>Chr: 2L<br>Start: 6532369<br>Stop: 6532569<br>TSS1: 6530509<br>Determined TSSs:<br>TSS1 (6530509) found for the transcript FBtr0306113 and the CRM is found uptream the gene (ensembl gene prediction).

TSS2 (6536111) found for the transcript FBtr0079335 and the CRM is found within the second intron.
Dorsal Mesoderm CRM-5559
Chr: 2L
Start: 6536168
Stop: 6536368
Determined TSSs:
TSS1 (6536111) found for the transcript FBtr0079335 and the CRM is found uptream the gene.
TSS2 (6530509) found for the transcript FBtr0306113 and the CRM is found upstream the gene.
Dorsal Mesoderm CRM-5561
Chr: 2L
Start: 6545230
Stop: 6545430
Determined TSSs:
TSS1 (6546972) found for the transcript FBtr0079334 and the CRM is found within the first intron.
TSS2 (6536111) found for the transcript FBtr0079335 and the CRM is found upstream the gene.

Twi — Interaction strengthened by CHIP-Seq experiment:
Twi binds to a predicted enhancer near the Eya gene at stage 8 to 10 (Data from Furlong's lab).
Details about the CRM:
Mesoderm CRM-5586
Chr: 2L;
Start: 6531029
Stop: 6531421
Determined TSSs:
TSS1 (6530509) found for the transcript FBtr0306113 and the CRM is found uptream the gene (ensembl gene prediction).
TSS2 (6536111) found for the transcript FBtr0079335 and the CRM is found within the second intron.

Hbr Hbr or Stumps acts downstream of FGF receptor, but upstream or in parallel to Ras1. Hbr is involved in both Heartless and Breathless signalling (Michelson et al., 1998).

- PMID:9778498
- http://flybase.org/reports/FBgn0020299.html
- http://www.sdbonline.org/sites/fly/dbzhnsky/heartbk1.htm
- http://www.uniprot.org/uniprot/Q9VFH9
- http://www.uniprot.org/uniprot/A0A0B4KGP0
- http://www.uniprot.org/uniprot/A0A0B4KFZ6
- http://www.uniprot.org/uniprot/Q9VFI0

Htl Heartless (Htl) is Drosophila FGF pathway receptor.
Expressed throughout the mesoderm of early embryos, Htl is required for proper mesoderm spreading (Shishido et al., 1997; Stathopoulos et al., 2004 and Wilson et al., 2005).
In Htl mutant, there is no spreading of the mesoderm.

- PMID:9187139
- PMID:15634694
- PMID:15075295
- http://flybase.org/reports/FBgn0010389.html
- http://www.sdbonline.org/fly/newgene/fgfr1-1.htm

- http://www.uniprot.org/uniprot/Q07407

Hop Upon ligand binding, Dome activates a receptor-associated JAK (Hopscotch), which in turn phosphorylates pre-associated Stat (Stat92E) dimers (Novak et al., 1998; Stancato et al., 1996).

- PMID:9647732
- PMID:8626752
- http://flybase.org/reports/FBgn0004864.html
- http://www.sdbonline.org/sites/fly/gene/hopscotc.htm
- http://www.uniprot.org/uniprot/B4Q623

Mad Mothers against dpp (Mad) and Medea (Med) transduce Dpp signal during Mesoderm specification (from stage 8 to 10 or 11).

Dpp signaling is initiated by binding of the ligand to a complex of the type I and type II serine/threonine kinase receptors,including Thickveins (Tkv).

Activated Tkv phosphorylates the BMP specific Mad, leading to its association with the co-Smad Med and accumulation of the Mad/Med complex in the nucleus.

Mad and Med-binding sites have been found in the promoters of many Dpp responsive genes (Xu et al., 1998 and Yao et al., 2008).

- PMID:9694800
- PMID:18506030
- http://flybase.org/reports/FBgn0011648.html
- http://www.sdbonline.org/fly/newgene/mothadpp.htm
- http://www.uniprot.org/uniprot/P42003

Mef2 Myocyte enhancer factor 2 (Mef2) is required for specification and differentiation of skeletal, cardiac, and visceral muscles (Gajewski et al., 1997; Cripps et al., 1998).

- PMID:9034334
- PMID:9450935
- http://www.sdbonline.org/fly/gene/mef2.htm
- http://flybase.org/reports/FBgn0011656.html
- http://www.uniprot.org/uniprot/P40791

Regulator	Comment
Tin	Interaction strengthened by CHIP-Seq experiment: Twi binds to predicted enhancers near Mef2 gene at stage 8 to 10 (Data from Furlong's lab).<br>Details about CRMs.<br>Mesoderm CRM-318<br>Chr: 2R<br>Start: 5819099<br>Stop: 5819743<br>Determined TSSs:<br>TSS1 (5817117) found for the transcript FBtr0088445 and the CRM is localized upstream the gene.<br>TSS2 (5832269) found for the transcript FBtr0330707 (Ensembl gene prediction) and the CRM is localized within the first intron.<br>Mesoderm CRM-320<br>Chr: 2R<br>Start: 5821186<br>Stop: 5821679<br>Determined TSSs:<br>TSS1 (5817117) found for the transcript FBtr0088445 and the CRM is localized upstream the gene.<br>TSS2 (5832269) found for the transcript FBtr0330707 (Ensembl gene prediction) and the CRM is localized within the first intron.<br>Mesoderm CRM-323

	Chr: 2R<br>Location: within the gene (intron)<br>Start: 5822684<br>Stop: 5823176<br>Determined TSSs:<br>TSS1 (5817117) found for the transcript FBtr0088445 and the CRM is localized upstream the gene.<br>TSS2 (5832269) found for the transcript FBtr0330707 (Ensembl gene prediction) and the CRM is localized within the first intron.
Twi	Interaction strengthened by CHIP-Seq experiment: Twi binds to predicted enhancers near Mef2 gene at stage 8 to 10 (Data from Furlong's lab)<br>Details about CRMs.<br>Mesoderm CRM-317<br>Chr: 2R<br>Start: 5817522<br>Stop: 5817722<br>Determined TSSs:<br>TSS1 (5817117) found for the transcript FBtr0088445 and the CRM is found upstream the gene.<br>TSS2 (5832269) found for the transcript FBtr0330707 and the CRM is found within the first intron (Ensembl gene prediction).<br>Mesoderm CRM-318<br>Chr: 2R<br>Start: 5819099<br>Stop: 5819743<br>TSS1 (5817117) found for the transcript FBtr0088445 and the CRM is found upstream the gene.<br>TSS2 (5832269) found for the transcript FBtr0330707 and the CRM is found within the first intron (Ensembl gene prediction).<br>Mesoderm CRM-320<br>Chr: 2R<br>Start: 5821186<br>Stop: 5821679<br>Determined TSSs:<br>TSS1 (5817117) found for the transcript FBtr0088445 and the CRM is found upstream the gene.<br>TSS2 (5832269) found for the transcript FBtr0330707 and the CRM is found within the first intron (Ensembl gene prediction).<br>Mesoderm CRM-323<br>Chr: 2R<br>Start: 5822684<br>Stop: 5823176<br>Determined TSSs:<br>TSS1 (5817117) found for the transcript FBtr0088445 and the CRM is found upstream the gene.<br>TSS2 (5832269) found for the transcript FBtr0330707 and the CRM is found within the first intron (Ensembl gene prediction).<br>Mesoderm CRM-324<br>Chr: 2R<br>Start: 5823318<br>Stop: 5824141<br>Determined TSSs:

TSS1 (5817117) found for the transcript FBtr0088445 and the CRM is found upstream the gene.
TSS2 (5832269) found for the transcript FBtr0330707 and the CRM is found within the first intron (Ensembl gene prediction).
Mesoderm CRM-327
Chr: 2R
Start: 5825713
Stop: 5826107
Determined TSSs:
TSS1 (5832269) found for the transcript FBtr0330707 and the CRM is found within the first intron (Ensembl gene prediction).
TSS2 (5817117) found for the transcript FBtr0088445 and the CRM is found upstream the gene.

Nicd A proteolytic processing mediates the release of the Notch intracellular domain Nicd, which then enters the nucleus and interacts with the CSL (CBF1, Su(H) and LAG-1) complex.
For references and links, see the Notch node.

Notch Notch is ubiquitously express during gastrulation and mesoderm subdivision.
It is activated by Delta (Dl) and Serrate ligands.
Loss- and gain-of function experiments revealed that Notch is required to repress Twist expression at stage 10 (Tapanes-Castillo and Baylies, 2004).
- PMID:15128668
- http://www.sdbonline.org/fly/neural/notch1.htm
- http://flybase.org/reports/FBgn0004647.html
- http://www.uniprot.org/uniprot/P07207

Pan Pangolin (Pan) is the Wg signalling effector.
Wg is required for the development of H and muscle during mesoderm development (Wu et al., 1995; Baylies et al., 1995; Frasch et al., 1999).
- PMID:7781903
- PMID:8582292
- http://www.sdbonline.org/fly/segment/pangoln1.htm
- http://flybase.org/reports/FBgn0085432.html
- http://www.uniprot.org/uniprot/P91943
- http://www.uniprot.org/uniprot/Q8IMA8

Pka Protein kinase A (Pka) acts at two levels in Hh pathway.
First, it phosphorylates Smoothened (Smo) cytoplasmic to alleviate Smo-mediated recruitment of the cytoplasmic regulatory complex (Cos2, Fu, and Ci).
Second, its phosphorylates Ci and thereby targets it for proteolytic processing into a transcriptional repressor (Aikin et al., 2008).
- PMID:18379584
- http://flybase.org/reports/FBgn0000273.html
- http://www.sdbonline.org/fly/newgene/pka-c1.htm
- http://www.uniprot.org/uniprot/P91682
- http://www.uniprot.org/uniprot/P12370

Pnr Pannier (Pnr) is a GATA class transcription factor expressed in both the dorsal ectoderm and dorsal mesoderm.
Pnr is required for cardiac cell specification.
Its expression in the dorsal mesoderm is controled by Dpp and Tin (Reim et al., 2005 ; Tao et al., 2007).
- PMID:16221729
- PMID:17208472
- http://flybase.org/reports/FBgn0003117.html

- http://www.sdbonline.org/fly/gene/pannier.htm
- http://www.uniprot.org/uniprot/P52168

Regulator	Comment
Tin	Interaction strengthened by CHIP-Seq experiment: Twi binds to a predicted enhancer near Pnr gene at stage 8 to 10 (Data from Furlong's lab)<br>Details about the CRM.<br>MesoCRM-2429<br>Chr: 3R<br>Start: 11861426<br>Stop: 11861659<br>Determined TSS:<br>TSS1 (11860307) found for the transcript FBtr0083221 and the CRM is found within the first intron.

Pnt Phosphorylated Pointed P2 (PntP2 or Pnt in the model) is the effector (activator) of Epidermal Growth Factor signalling pathway (Alvarez et al., 2003 ; Shiloh et al., 2005).

- PMID:12756183
- PMID:16123311
- http://flybase.org/reports/FBgn0003118.html
- http://www.sdbonline.org/fly/neural/pointed.htm
- http://www.uniprot.org/uniprot/P51022
- http://www.uniprot.org/uniprot/P51023

Poxm The Pax gene Pox meso (Poxm) was the first and so far only gene whose initial expression was shown to occur specifically in the anlage of the somatic mesoderm.
The development of larval body wall muscles involve two distinct Pox meso functions (Duan et al., 2007).
One is the specification within the high Twi or Slp domain, of a subdomain of competence for lateral and ventral muscle development.
The second function is the involvement of poxm in muscle differentiation.

- PMID:17942482
- http://flybase.org/reports/FBgn0003129.html
- http://www.sdbonline.org/fly/genebrief/poxmeso.htm

Ptc In the embryo, the distribution of Hh is regulated by the receptor Patched (Ptc).
In the absence of Hh binding, Ptc suppresses the function of Smoothened (Smo) (Taipale et al., 2002).

- PMID:12192414
- http://www.sdbonline.org/fly/segment/patched1.htm
- http://flybase.org/reports/FBgn0003892.html
- http://www.uniprot.org/uniprot/P18502

Ras Ras functions downstream of the EGF-receptor to promote activation of Raf (pole holein Drosophila), MEK (Dsor 1 in Drosophila) and eventually activation of the ERK MAP kinase Rolled (McKay et al., 2007).
Ras is required for H formation (Liu et al., 2006).

- PMID:17496910
- PMID:14515177
- http://flybase.org/reports/FBgn0003204.html
- http://www.sdbonline.org/fly/torstoll/ras85-1.htm
- http://www.uniprot.org/uniprot/Q07152

Rl Rolled/MAP kinase (Rl) is essential to the proper functioning of the Ras signalling pathway. After phosphorylation by Dsor1 and translocation in the nucleus, Rl phosphorylates Pointed P2 (Pnt in the model).
EGF target genes expression are then activated by Pnt (Roignant et al., 2006).

- PMID:16600911
- http://flybase.org/reports/FBgn0003256.html
- http://www.sdbonline.org/fly/torstoll/mapkin1.htm
- http://www.uniprot.org/uniprot/A8Y4W5

Slp Sloppy paired (Slp) is a direct target of the Wg signalling cascade and acts within the mesoderm to regulate the development of visceral mesoderm, somatic muscle and heart progenitors (Lee et al., 2000).
Wg and Slp are required to abrogate bap expression in H region (Azpiazu et al 1996; Riechmann et al 1997).

- PMID:11076769
- PMID:8985186
- PMID:9247334
- http://www.sdbonline.org/fly/segment/slopyprd.htm
- http://flybase.org/reports/FBgn0003430.html
- http://flybase.org/reports/FBgn0004567.html
- http://www.uniprot.org/uniprot/P32031
- http://www.uniprot.org/uniprot/P32030

Smo Smoothened (Smo) is a Hedgehog (Hh) signalling molecule that binds Ptc.
n the absence of Hh, Smo activity is inhibited.
Upon inactivation of Ptc by Hh binding, Smo recruits a large cytoplasmic complex required for Ci_act formation (Taipale et al., 2002 ; Ayers et al., 2010).

- PMID:12192414
- PMID:20207148
- http://flybase.org/reports/FBgn0003444.html
- http://www.sdbonline.org/fly/newgene/smoothen.htm
- http://www.uniprot.org/uniprot/P91682

Srp Serpent (Srp) is required for FB formation (Riechmann et al., 1998; Azpiazu et al., 1996).

- PMID:8985186
- PMID:9435291
- http://www.sdbonline.org/fly/gene/serpent.htm
- http://flybase.org/reports/FBgn0003507.html
- http://www.uniprot.org/uniprot/P52172

Stat92E Signal-transducer and activator of transcription protein at 92E (Stat92E) is the transcriptional effector of the JAK/STAT pathway.
Phosphorylated Stat92E homodimers translocate to the nucleus to regulate the expression of specific target genes.
Stat92E encodes a 761 amino acid Stat and is the only known Stat protein encoded protein in Drosophila (Hou et al, 1996; Yan et al, 1996).

- PMID:9066269
- PMID:8608596
- http://flybase.org/reports/FBgn0016917.html
- http://www.sdbonline.org/sites/fly/gene/marelle.htm
- http://www.uniprot.org/uniprot/Q24151

Regulator	Comment
Tin	Interaction strengthened by CHIP-Seq experiment: Tin binds to a predicted enhancer near Stat92E gene at stage 8 to 10 (Data from<br>Details about the CRM:<br>Mesoderm CRM-2754<br>Chr: 3R<br>Location: within the gene 0<br>Start: 16376083<br>Stop: 16376629

Determined TSSs:
TSS1 (16376829) found for the transcript FBtr0089485 and the CRM is found near the 5' UTR in the first intron.
TSS2 (16378033) found for the transcript FBtr0334581 (not found in genome database).

Tin Tinman (Tin) is expressed at stage 8 in the whole mesoderm before being restricted to dorsal mesoderm (H+VM) at stage 10 (Bodmer, 1993 and Frasch et al.,1995).
Tin is required for H and VM development (Bordmer, 1993 and Azpiazu et al.,1993).
Tin needs Twist for his initial activation (Bodmer et al., 1993).

- PMID:7915669
- PMID:7700357
- PMID:8101173
- http://flybase.org/reports/FBgn0004110.html
- http://www.uniprot.org/uniprot/P22711
- http://www.sdbonline.org/fly/gene/tinman.htm

Tkv Dpp signalling is initiated by binding of the ligand to a complex involving the serine/threonine kinase receptor Thickveins (Tkv) (Wartlick et al., 2011).

- PMID:21385708
- http://flybase.org/reports/FBgn0003716.html
- http://www.sdbonline.org/fly/gene/thickvan.htm
- http://www.uniprot.org/uniprot/Q27933

Twi Twist (Twi) is required for the formation of most body wall muscle and H precursor.
Twi mutant embryo develop no mesoderm (Furlong et al., 2001).
Wg and Slp are required to generate segmentally elevated levels of Twi expression (Bodmer et al., 1993; Baylies et al.,1996).

- PMID:11486054
- PMID:7915669
- PMID:9635422
- http://www.sdbonline.org/fly/torstoll/twist1.htm
- http://flybase.org/reports/FBgn0003900.html
- http://www.uniprot.org/uniprot/P10627

Zfh-1 Zn finger homeodomain 1 (Zfh1) is required for the specification and differentiation of mesodermal tissue, including the heart and muscle (Laic et al., 1991 and 1993).

- PMID:1680377
- PMID:8097886
- http://flybase.org/reports/FBgn0004606.html
- http://www.sdbonline.org/fly/gene/zincfh11.htm
- http://www.uniprot.org/uniprot/P28166

Regulator	Comment
Twi	Interaction strengthened by CHIP-Seq experiment: Twi binds to predicted enhancers near Zfh1 gene at stage 8 to 10 (Data from Furlong's lab).<br>Details about CRMs:<br>Mesoderm CRM-3413<br>Chr: 3R<br>Start: 26591711<br>Stop: 26591911<br>Determined TSSs:<br>TSS1 (26591647) found for the transcript FBtr0331180 and the CRM is found near the 5' UTR in the first intron (ensembl gene prediction).<br>TSS2 (26608979) found for the transcript FBtr0085702 and the CRM is found upstream the gene.<br>Mesoderm CRM-3415

Chr: 3R
Location within the gene (intron)
Start: 26595914
Stop: 26596265
Determined TSSs:
TSS1 (26591647) found for the transcript FBtr0331180 and the CRM is found near the 5' UTR in the first intron (ensembl gene prediction).
TSS2 (26608979) found for the transcript FBtr0085702 and the CRM is found upstream the gene.
