# Supplementary material for: Qualitative Dynamical Modelling Can Formally Explain Mesoderm Specification and Predict Novel Developmental Phenotypes
Source: PLoS Comput Biol. 2016 Sep 6;12(9):e1005073. doi: 10.1371/journal.pcbi.1005073 (PMC5012701; doi:10.1371/journal.pcbi.1005073)
Supplement: S2 Text — (PDF) [file pcbi.1005073.s005.pdf]

Supporting Text 2

Experimental evidence supporting the choice of logical rules for Tin and Bap.

Logical rule for Tin (three levels: 0,1 and 2)

Tin is expressed in the visceral muscles (VM) (level 1) and at an even higher level in the heart (H) (level 2).

In the model, we consider six activators of Tin: Mad, Med, Twi, Tin, Pan, Stat92E
Four of them are expressed in the VM: Mad, Med, Twi (only early), Tin (1), while all of them are present in the H.

Bap initial expression initially requires Tin in conjunction with Dpp signaling, in VM.
[Azpiazu and Frasch, 1993, Bodmer, 1993; Lee and Frasch, Zaffran et al., 2001]. Its maintenance requires Tin and Bin in the VM [Azpiazu and Frasch, 1993; Zaffran et al., 2001].

In Med loss-of-function mutants, some heart cells are specified [Data from Furlong's lab]. Tentatively, Medea might be produced from maternal transcripts and contribute to the specification of some heart cells. Furthermore, Wisotzkey (1998 - Development 25(8): 1433-45) demonstrated that Medea in not required for all Dpp dependent signaling.

This led us to define the following rule for Tin expression:

LR1: Mad & (Med | Twi | Tin)

In H territory, Tin level further increases in response to Wg (via Pan) and Upd (via StarE92) signaling [Liu et al., 2009 - Dev Cell 16(2): 280-91]. Hence, we defined the following requirement to enable Tin to reach its highest level (level 2):

LR2: Mad & Med & Pan & Stat92E

Final rules for Tin

Tin => 1 Iff
Mad & (Med | Twi | Tin)
& !(Mad & Med & Pan & Stat92E)

Tin => 2 Iff
Mad & Med & Pan & Stat92E

Logical rule for Bap (values 0-3)

Bap is expressed only in VM (at maximal level 3 at stage 10)
In the model, we consider seven regulators, including six activators, Mad & Med (heterodimers), Tin, Bin, Ci and En, and one inhibitor, Slp.
Slp is able to inhibit Bap expression. Indeed, in Slp KO, Bap expression domains expand anteriorly [Azpiazu and Frasch, 1993; Azpiazu et al., 1996; Lee and Frasch, 2000].
Bap expression is initiated by Dpp signal through Mad and Medea. Bap then activates bin, which in turns activates bap expression. When initiated, Bap expression needs Tin to be maintained [Azpiazu and Frasch, 1993, Bodmer, 1993; Zaffran et al., 2001; Lee and Frasch, 2005].
This led us to define the following rule LR1 (=> level 1)

LR1: !Slp & Tin & ((Mad & Med) | Bin)

In SLP gain-of-function mutants, small cluster of VM and a diminution of Fat body is observed [data from Furlong's lab].
Bap expression further increases in response to En and Ci, up to maximal level [Azpiazu et al., 1996].
En or Ci alone cannot mediate full Bap activation [Azpiazu et al., 1996].
Embryos mutant for both en and ci exhibit a stronger reduction of Bap expression compared to embryos mutant for en or ci only.
Furthermore, the over-expression of Bin leads to high Bap expression [data from Furlong's lab].
This leads us to consider two levels 2 and 3, which can be reached according to the following rules:

LR2: LR1 & ((Ci | En) & !(Ci & En))

LR3: (LR1 & Ci & En) | Bin:2

The final rules associated with the activation of Bap to levels 1, 2 and 3 are completed in order to avoid overlaps between the rules corresponding to each of these levels.

Final rules for Bap

Bab => 1 Iff
Bin:1 | (Mad & Med)) & Tin & !Slp
& !(Bin:1 & Mad & Med & Tin & En) & !(Ci & Tin & En) & !(Ci & Mad & Med & Tin & Bin:1) & !Bin:2
OR
Slp:1 & Mad:1 & Med & Ci & En & Tin & !Bin:2
OR
Ci & Tin & En & Slp & !(Mad & Med) & !Bin:2
OR
Ci & Tin & !En & Slp & Mad & Med & !Bin:2

Bab => 2 Iff
Bin:1 & Mad & Med & !(Ci & En) & !Slp & Tin & (Ci | En)
OR
Ci & Tin & En & !Slp & !(Mad & Med) & !Bin:2

Bab => 3 Iff
Bin:2 | (Ci:1 & En:1 & !Slp & Mad & Med & Tin)
