# Supplementary material for: Qualitative Dynamical Modelling Can Formally Explain Mesoderm Specification and Predict Novel Developmental Phenotypes
Source: PLoS Comput Biol. 2016 Sep 6;12(9):e1005073. doi: 10.1371/journal.pcbi.1005073 (PMC5012701; doi:10.1371/journal.pcbi.1005073)
Supplement: S1 Table — (PDF) [file pcbi.1005073.s006.pdf]

Supplementary Table S1. Logical formulas.

Node	Value	Logical function
Bap	1	(Bin:1 | (Mad & Med)) & Tin & !Slp & !(Bin:1 & Mad & Med & Tin & En) & !(Ci & Tin & En) & !(Ci & Mad & Med & Tin & Bin:1) & !Bin:2
		Slp:1 & Mad:1 & Med & Ci & En & Tin & !Bin:2
		Ci & Tin & En & Slp & !(Mad & Med) & !Bin:2
		Ci & Tin & !En & Slp & Mad & Med & !Bin:2
	2	Bin:1 & Mad & Med & !(Ci & En) & !Slp & Tin & (Ci | En)
		Ci & Tin & En & !Slp & !(Mad & Med) & !Bin:2
	3	Bin:2 | (Ci:1 & En:1 & !Slp & Mad & Med & Tin)
Bin	1	Bap
Brk	1	!(Shn & Mad & Med)
Ci	1	!Pka
Da	1	!Emc
Dsix4	1	(Tin:1 | Zfh-1:1) & !(Mad & Med)
Dome	1	Upd
Der	1	Spi:1
Doc	1	Mad:1 & Med & Pan:1
Emc	1	Nicd
E_Spl	1	Nicd & Stat92E & SuH
Eve	1	Pnt:1 & Htl:1 & Mad:1 & Pan:1 & Med & Tin:2 & Twi
Eya	1	Twi | Tin:2
Hbr	1	Pan & Htl
Htl	1	(((Pyr & Pan) | Ths) & Pan) | (Pyr & Ths)
Hop	1	Dome
Mad	1	Tkv
Mef2	1	Tin | Twi:2
Nicd	1	Notch
Notch	1	Delta
Pan	1	Wg:1
Pka	1	!Smo
Pnr	1	Doc:1 & Tin
Pnt	1	Rl:1
Poxm	1	Pan & Twi & !(Mad & Med)
Ptc	1	(basal value)
Ras	1	Der:1
Rl	1	Ras:1
Slp	1	Pan:1
Smo	1	(basal value)
Srp	1	Ci & !En & !(Mad & Med)
	2	Ci & En & !(Mad & Med)
Stat92E	1	Hop | Tin:2
Tin	1	Mad & (Med | Twi | Tin) & !(Mad & Med & Pan & Stat92E)
	2	Mad & Med & Pan & Stat92E
Tkv	1	Dpp:1
Twi	1	(Slp | Da) & Twi & !(Da & Slp) & !E_Spl
		Slp & Twi & !(Da & !E_Spl)
	2	Slp & Da & Twi & !E_Spl
Zfh-1	1	Twi

The Boolean operators NOT, AND and OR ar denoted by the symbols !, & and |, respectively.
Multilevel nodes have a Boolean rule associated with each target value. When several formulas are given for a single node target value (e.g. Twi => value 1), these are combined with OR operators.
Not listed, the input nodes Delta, Dpp, En, Hh, Med, Pyr, Shn, Spi, SuH, Ths, Upd and Wg keep their intial values.
