# Supplementary material for: Qualitative Dynamical Modelling Can Formally Explain Mesoderm Specification and Predict Novel Developmental Phenotypes
Source: PLoS Comput Biol. 2016 Sep 6;12(9):e1005073. doi: 10.1371/journal.pcbi.1005073 (PMC5012701; doi:10.1371/journal.pcbi.1005073)
Supplement: S2 File — The content of this supporting web archive folder documents known and novel gene expression pattern simulated with the drosophila mesoderm specification. Open the file “index.html” with a web browser to access this information. (ZIP) [file pcbi.1005073.s008.zip › SupWebArchive_W1/index.html]

Drosophila Mesoderm Model


- About
- Model
- Selected simulations
- Perturbation matrix

# Qualitative dynamical modelling can formally explain mesoderm specification and predict novel developmental phenotypes

Abibatou MBODJ, E. Hilary GUSTAFSON, Lucia CIGLAR, Guillaume JUNION, Aitor GONZALEZ, Charles GIRARDOT, Laurent PERRIN, Eileen E.M. FURLONG\*, Denis THIEFFRY\*

We have delineated a logical model encompassing 48 components and 82 regulatory interactions (page 2) involved in mesoderm specification during Drosophila development, thereby providing a formal integration of all available genetic information from the literature. The four main tissues derived from mesoderm correspond to alternative stable states of the model. We demonstrate that the model can predict known mutant phenotypes (page 3) and use it to systematically predict the effects of over 300 new in silico perturbations, both loss- and gain-of-function and combinations thereof (page 4). By generating specific genetic perturbations, we validated several of these new predictions experimentally. These robust predictions demonstrate the value of logically modelling to formally explain and predict complex developmental defects.
