# Supplementary material for: Qualitative Dynamical Modelling Can Formally Explain Mesoderm Specification and Predict Novel Developmental Phenotypes
Source: PLoS Comput Biol. 2016 Sep 6;12(9):e1005073. doi: 10.1371/journal.pcbi.1005073 (PMC5012701; doi:10.1371/journal.pcbi.1005073)
Supplement: S2 File — The content of this supporting web archive folder documents known and novel gene expression pattern simulated with the drosophila mesoderm specification. Open the file “index.html” with a web browser to access this information. (ZIP) [file pcbi.1005073.s008.zip › SupWebArchive_W1/matrix.html]

Drosophila Mesoderm Model


- About
- Model
- Selected simulations
- Perturbation matrix

# Systematic simulations of single and double mutants

This large matrix displays the results of systematic perturbations. Loss- and gain-of-function mutants (rows and column) were simulated iteratively using a Python script, along with pairwise combinations. The results of simulations for single mutants are displayed on the diagonal of the large matrix. The predicted phenotypes for double mutants are shown at the intersections of the corresponding columns and rows. Note that the cells corresponding to the crossing of a loss-function (lof) and a gain-of-function (gof) for the same gene are left empty. Each simulation is interpreted in terms of the formation of mesodermal tissue depending on the stables states reachable from the mutant initial states (i.e. wild-type initial conditions modified according to the genetic perturbation). Simulation results are graphically depicted using vignettes, with specific colours denoting situations with miss-expressed genes in each tissue (cf. colour key). This matrix encompasses numerous predictions, along with a few dozens of documented phenotypes. By clicking on a vignette, one can access to detailed information of the predicted patterns of gene expression in the four regions for the corresponding mutant.

## Cell types

|  |  |
| --- | --- |
| Visceral Mesoderm | Heart |
| Fat Body | Somatic Muscle |

## Phenotype keys

|  |  |  |  |  |
| --- | --- | --- | --- | --- |
| VM     VM (expected) | H     H (expected) | FB     FB (expected) | SM     SM (expected) | Unknown |

Back To Top


### Genotype: Ci\_1; Pnr\_0

Delta, Dpp, En, Hh, Med, Pyr, Shn, Su\_H\_CSL, Ths, Bap(3), Bin, Ci, Htl, Mad, Nicd, Tin, Mef2

Delta, Dpp, Med, Pyr, Shn, Spi, Su\_H\_CSL, Ths, Upd, Wg, Bap, Ci, Doc, E\_Spl, Htl, Mad, Nicd, Pan, Slp, Stat92E, Tin(2), Twi, Zfh-1, Eve, Eya, Hbr, Mef2

Delta, En, Hh, Med, Pyr, Su\_H\_CSL, Ths, Ci, Htl, Nicd, Brk, Srp(2)

Med, Pyr, Su\_H\_CSL, Ths, Wg, Ci, Da, Htl, Pan, Slp, Twi(2), Zfh-1, Brk, D-six4, Eya, Hbr, Mef2, Poxm, Srp

### Genotype: Doc\_0; Pnr\_0

Delta, Dpp, En, Hh, Med, Pyr, Shn, Su\_H\_CSL, Ths, Bap(3), Bin, Ci, Htl, Mad, Nicd, Tin, Mef2

Delta, Dpp, Med, Pyr, Shn, Spi, Su\_H\_CSL, Ths, Upd, Wg, E\_Spl, Htl, Mad, Nicd, Pan, Slp, Stat92E, Tin(2), Twi, Zfh-1, Eve, Eya, Hbr, Mef2

Delta, En, Hh, Med, Pyr, Su\_H\_CSL, Ths, Ci, Htl, Nicd, Brk, Srp(2)

Med, Pyr, Su\_H\_CSL, Ths, Wg, Da, Htl, Pan, Slp, Twi(2), Zfh-1, Brk, D-six4, Eya, Hbr, Mef2, Poxm

### Genotype: Nicd\_0

Delta, Dpp, En, Hh, Med, Pyr, Shn, Su\_H\_CSL, Ths, Bap(3), Bin, Ci, Da, Htl, Mad, Tin, Mef2

Delta, Dpp, En, Hh, Med, Pyr, Shn, Su\_H\_CSL, Ths, Bap(3), Bin, Ci, Da, Htl, Mad, Tin, Twi, Zfh-1, Eya, Mef2

Delta, Dpp, Med, Pyr, Shn, Spi, Su\_H\_CSL, Ths, Upd, Wg, Da, Doc, Htl, Mad, Pan, Slp, Stat92E, Tin(2), Twi(2), Zfh-1, Eve, Eya, Hbr, Mef2, Pnr

Delta, En, Hh, Med, Pyr, Su\_H\_CSL, Ths, Ci, Da, Htl, Brk, Srp(2)

Delta, En, Hh, Med, Pyr, Su\_H\_CSL, Ths, Ci, Da, Htl, Twi, Zfh-1, Brk, D-six4, Eya, Srp(2)

Med, Pyr, Su\_H\_CSL, Ths, Wg, Da, Htl, Pan, Slp, Twi(2), Zfh-1, Brk, D-six4, Eya, Hbr, Mef2, Poxm

### Genotype: Pnr\_1; Tin\_0

Delta, Dpp, En, Hh, Med, Pyr, Shn, Su\_H\_CSL, Ths, Ci, Htl, Mad, Nicd, Pnr

Delta, Dpp, Med, Pyr, Shn, Spi, Su\_H\_CSL, Ths, Upd, Wg, Doc, E\_Spl, Htl, Mad, Nicd, Pan, Slp, Stat92E, Twi, Zfh-1, Eya, Hbr, Pnr

Delta, En, Hh, Med, Pyr, Su\_H\_CSL, Ths, Ci, Htl, Nicd, Brk, Pnr, Srp(2)

Med, Pyr, Su\_H\_CSL, Ths, Wg, Da, Htl, Pan, Slp, Twi(2), Zfh-1, Brk, D-six4, Eya, Hbr, Mef2, Pnr, Poxm

### Genotype: Bap\_3; Twi\_0

Delta, Dpp, En, Hh, Med, Pyr, Shn, Su\_H\_CSL, Ths, Bap(3), Bin, Ci, Htl, Mad, Nicd, Tin, Mef2

Delta, Dpp, Med, Pyr, Shn, Spi, Su\_H\_CSL, Ths, Upd, Wg, Bap(3), Bin, Doc, E\_Spl, Htl, Mad, Nicd, Pan, Slp, Stat92E, Tin(2), Eya, Hbr, Mef2, Pnr

Delta, En, Hh, Med, Pyr, Su\_H\_CSL, Ths, Bap(3), Bin, Ci, Htl, Nicd, Brk, Srp(2)

Med, Pyr, Su\_H\_CSL, Ths, Wg, Bap(3), Bin, Da, Htl, Pan, Slp, Brk, Hbr

### Genotype: Bap\_3; Twi\_2

Delta, Dpp, En, Hh, Med, Pyr, Shn, Su\_H\_CSL, Ths, Bap(3), Bin, Ci, Htl, Mad, Nicd, Tin, Twi(2), Zfh-1, Eya, Mef2

Delta, Dpp, Med, Pyr, Shn, Spi, Su\_H\_CSL, Ths, Upd, Wg, Bap(3), Bin, Doc, E\_Spl, Htl, Mad, Nicd, Pan, Slp, Stat92E, Tin(2), Twi(2), Zfh-1, Eve, Eya, Hbr, Mef2, Pnr

Delta, En, Hh, Med, Pyr, Su\_H\_CSL, Ths, Bap(3), Bin, Ci, Htl, Nicd, Twi(2), Zfh-1, Brk, D-six4, Eya, Mef2, Srp(2)

Med, Pyr, Su\_H\_CSL, Ths, Wg, Bap(3), Bin, Da, Htl, Pan, Slp, Twi(2), Zfh-1, Brk, D-six4, Eya, Hbr, Mef2, Poxm

### Genotype: Pan\_0; Srp\_2

Delta, Dpp, En, Hh, Med, Pyr, Shn, Su\_H\_CSL, Ths, Bap(3), Bin, Ci, Htl, Mad, Nicd, Tin, Mef2, Srp(2)

Delta, Dpp, Med, Pyr, Shn, Spi, Su\_H\_CSL, Ths, Upd, Wg, Bap, E\_Spl, Htl, Mad, Nicd, Stat92E, Tin, Mef2, Srp(2)

Delta, En, Hh, Med, Pyr, Su\_H\_CSL, Ths, Ci, Htl, Nicd, Brk, Srp(2)

Med, Pyr, Su\_H\_CSL, Ths, Wg, Da, Htl, Brk, Srp(2)

Med, Pyr, Su\_H\_CSL, Ths, Wg, Da, Htl, Twi, Zfh-1, Brk, D-six4, Eya, Srp(2)

### Genotype: Pan\_0; Srp\_0

Delta, Dpp, En, Hh, Med, Pyr, Shn, Su\_H\_CSL, Ths, Bap(3), Bin, Ci, Htl, Mad, Nicd, Tin, Mef2

Delta, Dpp, Med, Pyr, Shn, Spi, Su\_H\_CSL, Ths, Upd, Wg, Bap, E\_Spl, Htl, Mad, Nicd, Stat92E, Tin, Mef2

Delta, En, Hh, Med, Pyr, Su\_H\_CSL, Ths, Ci, Htl, Nicd, Brk

Med, Pyr, Su\_H\_CSL, Ths, Wg, Da, Htl, Brk

Med, Pyr, Su\_H\_CSL, Ths, Wg, Da, Htl, Twi, Zfh-1, Brk, D-six4, Eya

### Genotype: Bap\_3

Delta, Dpp, En, Hh, Med, Pyr, Shn, Su\_H\_CSL, Ths, Bap(3), Bin, Ci, Htl, Mad, Nicd, Tin, Mef2

Delta, Dpp, Med, Pyr, Shn, Spi, Su\_H\_CSL, Ths, Upd, Wg, Bap(3), Bin, Doc, E\_Spl, Htl, Mad, Nicd, Pan, Slp, Stat92E, Tin(2), Twi, Zfh-1, Eve, Eya, Hbr, Mef2, Pnr

Delta, En, Hh, Med, Pyr, Su\_H\_CSL, Ths, Bap(3), Bin, Ci, Htl, Nicd, Brk, Srp(2)

Med, Pyr, Su\_H\_CSL, Ths, Wg, Bap(3), Bin, Da, Htl, Pan, Slp, Twi(2), Zfh-1, Brk, D-six4, Eya, Hbr, Mef2, Poxm

### Genotype: Bin\_0; Ci\_0

Delta, Dpp, En, Hh, Med, Pyr, Shn, Su\_H\_CSL, Ths, Bap, Htl, Mad, Nicd, Tin, Mef2

Delta, Dpp, Med, Pyr, Shn, Spi, Su\_H\_CSL, Ths, Upd, Wg, Doc, E\_Spl, Htl, Mad, Nicd, Pan, Slp, Stat92E, Tin(2), Twi, Zfh-1, Eve, Eya, Hbr, Mef2, Pnr

Delta, En, Hh, Med, Pyr, Su\_H\_CSL, Ths, Htl, Nicd, Brk

Med, Pyr, Su\_H\_CSL, Ths, Wg, Da, Htl, Pan, Slp, Twi(2), Zfh-1, Brk, D-six4, Eya, Hbr, Mef2, Poxm

### Genotype: Bin\_0; Ci\_1

Delta, Dpp, En, Hh, Med, Pyr, Shn, Su\_H\_CSL, Ths, Bap(3), Ci, Htl, Mad, Nicd, Tin, Mef2

Delta, Dpp, Med, Pyr, Shn, Spi, Su\_H\_CSL, Ths, Upd, Wg, Bap, Ci, Doc, E\_Spl, Htl, Mad, Nicd, Pan, Slp, Stat92E, Tin(2), Twi, Zfh-1, Eve, Eya, Hbr, Mef2, Pnr

Delta, En, Hh, Med, Pyr, Su\_H\_CSL, Ths, Ci, Htl, Nicd, Brk, Srp(2)

Med, Pyr, Su\_H\_CSL, Ths, Wg, Ci, Da, Htl, Pan, Slp, Twi(2), Zfh-1, Brk, D-six4, Eya, Hbr, Mef2, Poxm, Srp

### Genotype: Ci\_0; Mef2\_0

Delta, Dpp, En, Hh, Med, Pyr, Shn, Su\_H\_CSL, Ths, Bap, Htl, Mad, Nicd, Tin

Delta, Dpp, En, Hh, Med, Pyr, Shn, Su\_H\_CSL, Ths, Bap(2), Bin, Htl, Mad, Nicd, Tin

Delta, Dpp, Med, Pyr, Shn, Spi, Su\_H\_CSL, Ths, Upd, Wg, Doc, E\_Spl, Htl, Mad, Nicd, Pan, Slp, Stat92E, Tin(2), Twi, Zfh-1, Eve, Eya, Hbr, Pnr

Delta, En, Hh, Med, Pyr, Su\_H\_CSL, Ths, Htl, Nicd, Brk

Med, Pyr, Su\_H\_CSL, Ths, Wg, Da, Htl, Pan, Slp, Twi(2), Zfh-1, Brk, D-six4, Eya, Hbr, Poxm

### Genotype: Ci\_0; Mef2\_1

Delta, Dpp, En, Hh, Med, Pyr, Shn, Su\_H\_CSL, Ths, Bap, Htl, Mad, Nicd, Tin, Mef2

Delta, Dpp, En, Hh, Med, Pyr, Shn, Su\_H\_CSL, Ths, Bap(2), Bin, Htl, Mad, Nicd, Tin, Mef2

Delta, Dpp, Med, Pyr, Shn, Spi, Su\_H\_CSL, Ths, Upd, Wg, Doc, E\_Spl, Htl, Mad, Nicd, Pan, Slp, Stat92E, Tin(2), Twi, Zfh-1, Eve, Eya, Hbr, Mef2, Pnr

Delta, En, Hh, Med, Pyr, Su\_H\_CSL, Ths, Htl, Nicd, Brk, Mef2

Med, Pyr, Su\_H\_CSL, Ths, Wg, Da, Htl, Pan, Slp, Twi(2), Zfh-1, Brk, D-six4, Eya, Hbr, Mef2, Poxm

### Genotype: Pan\_1; Pnr\_0

Delta, Dpp, En, Hh, Med, Pyr, Shn, Su\_H\_CSL, Ths, Bap, Ci, Doc, Htl, Mad, Nicd, Pan, Slp, Tin, Hbr, Mef2

Delta, Dpp, En, Hh, Med, Pyr, Shn, Su\_H\_CSL, Ths, Bap, Ci, Doc, Htl, Mad, Nicd, Pan, Slp, Tin, Twi, Zfh-1, Eya, Hbr, Mef2

Delta, Dpp, Med, Pyr, Shn, Spi, Su\_H\_CSL, Ths, Upd, Wg, Doc, E\_Spl, Htl, Mad, Nicd, Pan, Slp, Stat92E, Tin(2), Twi, Zfh-1, Eve, Eya, Hbr, Mef2

Delta, En, Hh, Med, Pyr, Su\_H\_CSL, Ths, Ci, Htl, Nicd, Pan, Slp, Brk, Hbr, Srp(2)

Delta, En, Hh, Med, Pyr, Su\_H\_CSL, Ths, Ci, Htl, Nicd, Pan, Slp, Twi, Zfh-1, Brk, D-six4, Eya, Hbr, Poxm, Srp(2)

Med, Pyr, Su\_H\_CSL, Ths, Wg, Da, Htl, Pan, Slp, Twi(2), Zfh-1, Brk, D-six4, Eya, Hbr, Mef2, Poxm

### Genotype: Pan\_1; Pnr\_1

Delta, Dpp, En, Hh, Med, Pyr, Shn, Su\_H\_CSL, Ths, Bap, Ci, Doc, Htl, Mad, Nicd, Pan, Slp, Tin, Hbr, Mef2, Pnr

Delta, Dpp, En, Hh, Med, Pyr, Shn, Su\_H\_CSL, Ths, Bap, Ci, Doc, Htl, Mad, Nicd, Pan, Slp, Tin, Twi, Zfh-1, Eya, Hbr, Mef2, Pnr

Delta, Dpp, Med, Pyr, Shn, Spi, Su\_H\_CSL, Ths, Upd, Wg, Doc, E\_Spl, Htl, Mad, Nicd, Pan, Slp, Stat92E, Tin(2), Twi, Zfh-1, Eve, Eya, Hbr, Mef2, Pnr

Delta, En, Hh, Med, Pyr, Su\_H\_CSL, Ths, Ci, Htl, Nicd, Pan, Slp, Brk, Hbr, Pnr, Srp(2)

Delta, En, Hh, Med, Pyr, Su\_H\_CSL, Ths, Ci, Htl, Nicd, Pan, Slp, Twi, Zfh-1, Brk, D-six4, Eya, Hbr, Pnr, Poxm, Srp(2)

Med, Pyr, Su\_H\_CSL, Ths, Wg, Da, Htl, Pan, Slp, Twi(2), Zfh-1, Brk, D-six4, Eya, Hbr, Mef2, Pnr, Poxm

### Genotype: Mef2\_1; Nicd\_1

Delta, Dpp, En, Hh, Med, Pyr, Shn, Su\_H\_CSL, Ths, Bap(3), Bin, Ci, Htl, Mad, Nicd, Tin, Mef2

Delta, Dpp, Med, Pyr, Shn, Spi, Su\_H\_CSL, Ths, Upd, Wg, Doc, E\_Spl, Htl, Mad, Nicd, Pan, Slp, Stat92E, Tin(2), Twi, Zfh-1, Eve, Eya, Hbr, Mef2, Pnr

Delta, En, Hh, Med, Pyr, Su\_H\_CSL, Ths, Ci, Htl, Nicd, Brk, Mef2, Srp(2)

Med, Pyr, Su\_H\_CSL, Ths, Wg, Htl, Nicd, Pan, Slp, Twi, Zfh-1, Brk, D-six4, Eya, Hbr, Mef2, Poxm

### Genotype: Mad\_0; Pan\_1

Delta, Dpp, En, Hh, Med, Pyr, Shn, Su\_H\_CSL, Ths, Ci, Htl, Nicd, Pan, Slp, Brk, Hbr, Srp(2)

Delta, Dpp, En, Hh, Med, Pyr, Shn, Su\_H\_CSL, Ths, Ci, Htl, Nicd, Pan, Slp, Twi, Zfh-1, Brk, D-six4, Eya, Hbr, Poxm, Srp(2)

Delta, Dpp, Med, Pyr, Shn, Spi, Su\_H\_CSL, Ths, Upd, Wg, E\_Spl, Htl, Nicd, Pan, Slp, Stat92E, Twi, Zfh-1, Brk, D-six4, Eya, Hbr, Poxm

Delta, En, Hh, Med, Pyr, Su\_H\_CSL, Ths, Ci, Htl, Nicd, Pan, Slp, Brk, Hbr, Srp(2)

Delta, En, Hh, Med, Pyr, Su\_H\_CSL, Ths, Ci, Htl, Nicd, Pan, Slp, Twi, Zfh-1, Brk, D-six4, Eya, Hbr, Poxm, Srp(2)

Med, Pyr, Su\_H\_CSL, Ths, Wg, Da, Htl, Pan, Slp, Twi(2), Zfh-1, Brk, D-six4, Eya, Hbr, Mef2, Poxm

### Genotype: Mad\_0; Pan\_0

Delta, Dpp, En, Hh, Med, Pyr, Shn, Su\_H\_CSL, Ths, Ci, Htl, Nicd, Brk, Srp(2)

Delta, Dpp, Med, Pyr, Shn, Spi, Su\_H\_CSL, Ths, Upd, Wg, E\_Spl, Htl, Nicd, Stat92E, Brk

Delta, En, Hh, Med, Pyr, Su\_H\_CSL, Ths, Ci, Htl, Nicd, Brk, Srp(2)

Med, Pyr, Su\_H\_CSL, Ths, Wg, Da, Htl, Brk

Med, Pyr, Su\_H\_CSL, Ths, Wg, Da, Htl, Twi, Zfh-1, Brk, D-six4, Eya

### Genotype: Mef2\_1; Nicd\_0

Delta, Dpp, En, Hh, Med, Pyr, Shn, Su\_H\_CSL, Ths, Bap(3), Bin, Ci, Da, Htl, Mad, Tin, Mef2

Delta, Dpp, En, Hh, Med, Pyr, Shn, Su\_H\_CSL, Ths, Bap(3), Bin, Ci, Da, Htl, Mad, Tin, Twi, Zfh-1, Eya, Mef2

Delta, Dpp, Med, Pyr, Shn, Spi, Su\_H\_CSL, Ths, Upd, Wg, Da, Doc, Htl, Mad, Pan, Slp, Stat92E, Tin(2), Twi(2), Zfh-1, Eve, Eya, Hbr, Mef2, Pnr

Delta, En, Hh, Med, Pyr, Su\_H\_CSL, Ths, Ci, Da, Htl, Brk, Mef2, Srp(2)

Delta, En, Hh, Med, Pyr, Su\_H\_CSL, Ths, Ci, Da, Htl, Twi, Zfh-1, Brk, D-six4, Eya, Mef2, Srp(2)

Med, Pyr, Su\_H\_CSL, Ths, Wg, Da, Htl, Pan, Slp, Twi(2), Zfh-1, Brk, D-six4, Eya, Hbr, Mef2, Poxm

### Genotype: Med\_0; Slp\_1

Delta, Dpp, Hh, Pyr, Shn, Su\_H\_CSL, Ths, Ci, Htl, Mad, Nicd, Slp, Tin, Brk, D-six4, Mef2, Srp

Delta, Dpp, Hh, Pyr, Shn, Su\_H\_CSL, Ths, Ci, Htl, Mad, Nicd, Slp, Tin, Twi, Zfh-1, Brk, D-six4, Eya, Mef2, Srp

Delta, Dpp, Pyr, Shn, Spi, Su\_H\_CSL, Ths, Upd, Wg, E\_Spl, Htl, Mad, Nicd, Pan, Slp, Stat92E, Tin, Twi, Zfh-1, Brk, D-six4, Eya, Hbr, Mef2, Poxm

Delta, Hh, Pyr, Su\_H\_CSL, Ths, Ci, Htl, Nicd, Slp, Brk, Srp

Delta, Hh, Pyr, Su\_H\_CSL, Ths, Ci, Htl, Nicd, Slp, Twi, Zfh-1, Brk, D-six4, Eya, Srp

Pyr, Su\_H\_CSL, Ths, Wg, Da, Htl, Pan, Slp, Twi(2), Zfh-1, Brk, D-six4, Eya, Hbr, Mef2, Poxm

### Genotype: Med\_0; Slp\_0

Delta, Dpp, En, Hh, Pyr, Shn, Su\_H\_CSL, Ths, Bap(2), Bin, Ci, Htl, Mad, Nicd, Tin, Brk, D-six4, Mef2, Srp(2)

Delta, Dpp, En, Pyr, Shn, Spi, Su\_H\_CSL, Ths, Upd, Wg, E\_Spl, Htl, Mad, Nicd, Pan, Stat92E, Tin, Brk, D-six4, Hbr, Mef2

Delta, En, Hh, Pyr, Su\_H\_CSL, Ths, Ci, Htl, Nicd, Brk, Srp(2)

En, Pyr, Su\_H\_CSL, Ths, Wg, Da, Htl, Pan, Brk, Hbr

En, Pyr, Su\_H\_CSL, Ths, Wg, Da, Htl, Pan, Twi, Zfh-1, Brk, D-six4, Eya, Hbr, Poxm

### Genotype: Med\_1; Twi\_0

Delta, Dpp, En, Hh, Med, Pyr, Shn, Su\_H\_CSL, Ths, Bap(3), Bin, Ci, Htl, Mad, Nicd, Tin, Mef2

Delta, Dpp, Med, Pyr, Shn, Spi, Su\_H\_CSL, Ths, Upd, Wg, Doc, E\_Spl, Htl, Mad, Nicd, Pan, Slp, Stat92E, Tin(2), Eya, Hbr, Mef2, Pnr

Delta, En, Hh, Med, Pyr, Su\_H\_CSL, Ths, Ci, Htl, Nicd, Brk, Srp(2)

Med, Pyr, Su\_H\_CSL, Ths, Wg, Da, Htl, Pan, Slp, Brk, Hbr

### Genotype: Med\_1; Twi\_2

Delta, Dpp, En, Hh, Med, Pyr, Shn, Su\_H\_CSL, Ths, Bap(3), Bin, Ci, Htl, Mad, Nicd, Tin, Twi(2), Zfh-1, Eya, Mef2

Delta, Dpp, Med, Pyr, Shn, Spi, Su\_H\_CSL, Ths, Upd, Wg, Doc, E\_Spl, Htl, Mad, Nicd, Pan, Slp, Stat92E, Tin(2), Twi(2), Zfh-1, Eve, Eya, Hbr, Mef2, Pnr

Delta, En, Hh, Med, Pyr, Su\_H\_CSL, Ths, Ci, Htl, Nicd, Twi(2), Zfh-1, Brk, D-six4, Eya, Mef2, Srp(2)

Med, Pyr, Su\_H\_CSL, Ths, Wg, Da, Htl, Pan, Slp, Twi(2), Zfh-1, Brk, D-six4, Eya, Hbr, Mef2, Poxm

### Genotype: Mad\_0; Mef2\_1

Delta, Dpp, En, Hh, Med, Pyr, Shn, Su\_H\_CSL, Ths, Ci, Htl, Nicd, Brk, Mef2, Srp(2)

Delta, Dpp, Med, Pyr, Shn, Spi, Su\_H\_CSL, Ths, Upd, Wg, E\_Spl, Htl, Nicd, Pan, Slp, Stat92E, Twi, Zfh-1, Brk, D-six4, Eya, Hbr, Mef2, Poxm

Delta, En, Hh, Med, Pyr, Su\_H\_CSL, Ths, Ci, Htl, Nicd, Brk, Mef2, Srp(2)

Med, Pyr, Su\_H\_CSL, Ths, Wg, Da, Htl, Pan, Slp, Twi(2), Zfh-1, Brk, D-six4, Eya, Hbr, Mef2, Poxm

### Genotype: Mad\_0; Mef2\_0

Delta, Dpp, En, Hh, Med, Pyr, Shn, Su\_H\_CSL, Ths, Ci, Htl, Nicd, Brk, Srp(2)

Delta, Dpp, Med, Pyr, Shn, Spi, Su\_H\_CSL, Ths, Upd, Wg, E\_Spl, Htl, Nicd, Pan, Slp, Stat92E, Twi, Zfh-1, Brk, D-six4, Eya, Hbr, Poxm

Delta, En, Hh, Med, Pyr, Su\_H\_CSL, Ths, Ci, Htl, Nicd, Brk, Srp(2)

Med, Pyr, Su\_H\_CSL, Ths, Wg, Da, Htl, Pan, Slp, Twi(2), Zfh-1, Brk, D-six4, Eya, Hbr, Poxm

### Genotype: Doc\_1; Tin\_0

Delta, Dpp, En, Hh, Med, Pyr, Shn, Su\_H\_CSL, Ths, Ci, Doc, Htl, Mad, Nicd

Delta, Dpp, Med, Pyr, Shn, Spi, Su\_H\_CSL, Ths, Upd, Wg, Doc, E\_Spl, Htl, Mad, Nicd, Pan, Slp, Stat92E, Twi, Zfh-1, Eya, Hbr

Delta, En, Hh, Med, Pyr, Su\_H\_CSL, Ths, Ci, Doc, Htl, Nicd, Brk, Srp(2)

Med, Pyr, Su\_H\_CSL, Ths, Wg, Da, Doc, Htl, Pan, Slp, Twi(2), Zfh-1, Brk, D-six4, Eya, Hbr, Mef2, Poxm

### Genotype: Doc\_1; Tin\_2

Delta, Dpp, En, Hh, Med, Pyr, Shn, Su\_H\_CSL, Ths, Bap(3), Bin, Ci, Doc, E\_Spl, Htl, Mad, Nicd, Stat92E, Tin(2), Eya, Mef2, Pnr

Delta, Dpp, Med, Pyr, Shn, Spi, Su\_H\_CSL, Ths, Upd, Wg, Doc, E\_Spl, Htl, Mad, Nicd, Pan, Slp, Stat92E, Tin(2), Twi, Zfh-1, Eve, Eya, Hbr, Mef2, Pnr

Delta, En, Hh, Med, Pyr, Su\_H\_CSL, Ths, Bap(2), Bin, Ci, Doc, E\_Spl, Htl, Nicd, Stat92E, Tin(2), Brk, D-six4, Eya, Mef2, Pnr, Srp(2)

Med, Pyr, Su\_H\_CSL, Ths, Wg, Da, Doc, Htl, Pan, Slp, Stat92E, Tin(2), Twi(2), Zfh-1, Brk, D-six4, Eya, Hbr, Mef2, Pnr, Poxm

### Genotype: Bin\_2; Twi\_2

Delta, Dpp, En, Hh, Med, Pyr, Shn, Su\_H\_CSL, Ths, Bap(3), Bin(2), Ci, Htl, Mad, Nicd, Tin, Twi(2), Zfh-1, Eya, Mef2

Delta, Dpp, Med, Pyr, Shn, Spi, Su\_H\_CSL, Ths, Upd, Wg, Bap(3), Bin(2), Doc, E\_Spl, Htl, Mad, Nicd, Pan, Slp, Stat92E, Tin(2), Twi(2), Zfh-1, Eve, Eya, Hbr, Mef2, Pnr

Delta, En, Hh, Med, Pyr, Su\_H\_CSL, Ths, Bap(3), Bin(2), Ci, Htl, Nicd, Twi(2), Zfh-1, Brk, D-six4, Eya, Mef2, Srp(2)

Med, Pyr, Su\_H\_CSL, Ths, Wg, Bap(3), Bin(2), Da, Htl, Pan, Slp, Twi(2), Zfh-1, Brk, D-six4, Eya, Hbr, Mef2, Poxm

### Genotype: Bin\_2; Twi\_0

Delta, Dpp, En, Hh, Med, Pyr, Shn, Su\_H\_CSL, Ths, Bap(3), Bin(2), Ci, Htl, Mad, Nicd, Tin, Mef2

Delta, Dpp, Med, Pyr, Shn, Spi, Su\_H\_CSL, Ths, Upd, Wg, Bap(3), Bin(2), Doc, E\_Spl, Htl, Mad, Nicd, Pan, Slp, Stat92E, Tin(2), Eya, Hbr, Mef2, Pnr

Delta, En, Hh, Med, Pyr, Su\_H\_CSL, Ths, Bap(3), Bin(2), Ci, Htl, Nicd, Brk, Srp(2)

Med, Pyr, Su\_H\_CSL, Ths, Wg, Bap(3), Bin(2), Da, Htl, Pan, Slp, Brk, Hbr

### Genotype: Mad\_1; Nicd\_0

Delta, Dpp, En, Hh, Med, Pyr, Shn, Su\_H\_CSL, Ths, Bap(3), Bin, Ci, Da, Htl, Mad, Tin, Mef2

Delta, Dpp, En, Hh, Med, Pyr, Shn, Su\_H\_CSL, Ths, Bap(3), Bin, Ci, Da, Htl, Mad, Tin, Twi, Zfh-1, Eya, Mef2

Delta, Dpp, Med, Pyr, Shn, Spi, Su\_H\_CSL, Ths, Upd, Wg, Da, Doc, Htl, Mad, Pan, Slp, Stat92E, Tin(2), Twi(2), Zfh-1, Eve, Eya, Hbr, Mef2, Pnr

Delta, En, Hh, Med, Pyr, Su\_H\_CSL, Ths, Bap(3), Bin, Ci, Da, Htl, Mad, Tin, Brk, Mef2

Delta, En, Hh, Med, Pyr, Su\_H\_CSL, Ths, Bap(3), Bin, Ci, Da, Htl, Mad, Tin, Twi, Zfh-1, Brk, Eya, Mef2

Med, Pyr, Su\_H\_CSL, Ths, Wg, Da, Doc, Htl, Mad, Pan, Slp, Tin, Twi(2), Zfh-1, Brk, Eya, Hbr, Mef2, Pnr

### Genotype: Mad\_1; Nicd\_1

Delta, Dpp, En, Hh, Med, Pyr, Shn, Su\_H\_CSL, Ths, Bap(3), Bin, Ci, Htl, Mad, Nicd, Tin, Mef2

Delta, Dpp, Med, Pyr, Shn, Spi, Su\_H\_CSL, Ths, Upd, Wg, Doc, E\_Spl, Htl, Mad, Nicd, Pan, Slp, Stat92E, Tin(2), Twi, Zfh-1, Eve, Eya, Hbr, Mef2, Pnr

Delta, En, Hh, Med, Pyr, Su\_H\_CSL, Ths, Bap(3), Bin, Ci, Htl, Mad, Nicd, Tin, Brk, Mef2

Med, Pyr, Su\_H\_CSL, Ths, Wg, Doc, Htl, Mad, Nicd, Pan, Slp, Tin, Twi, Zfh-1, Brk, Eya, Hbr, Mef2, Pnr

### Genotype: Doc\_0; Srp\_2

Delta, Dpp, En, Hh, Med, Pyr, Shn, Su\_H\_CSL, Ths, Bap(3), Bin, Ci, Htl, Mad, Nicd, Tin, Mef2, Srp(2)

Delta, Dpp, Med, Pyr, Shn, Spi, Su\_H\_CSL, Ths, Upd, Wg, E\_Spl, Htl, Mad, Nicd, Pan, Slp, Stat92E, Tin(2), Twi, Zfh-1, Eve, Eya, Hbr, Mef2, Srp(2)

Delta, En, Hh, Med, Pyr, Su\_H\_CSL, Ths, Ci, Htl, Nicd, Brk, Srp(2)

Med, Pyr, Su\_H\_CSL, Ths, Wg, Da, Htl, Pan, Slp, Twi(2), Zfh-1, Brk, D-six4, Eya, Hbr, Mef2, Poxm, Srp(2)

### Genotype: Mad\_0; Nicd\_1

Delta, Dpp, En, Hh, Med, Pyr, Shn, Su\_H\_CSL, Ths, Ci, Htl, Nicd, Brk, Srp(2)

Delta, Dpp, Med, Pyr, Shn, Spi, Su\_H\_CSL, Ths, Upd, Wg, E\_Spl, Htl, Nicd, Pan, Slp, Stat92E, Twi, Zfh-1, Brk, D-six4, Eya, Hbr, Poxm

Delta, En, Hh, Med, Pyr, Su\_H\_CSL, Ths, Ci, Htl, Nicd, Brk, Srp(2)

Med, Pyr, Su\_H\_CSL, Ths, Wg, Htl, Nicd, Pan, Slp, Twi, Zfh-1, Brk, D-six4, Eya, Hbr, Poxm

### Genotype: Mad\_0; Nicd\_0

Delta, Dpp, En, Hh, Med, Pyr, Shn, Su\_H\_CSL, Ths, Ci, Da, Htl, Brk, Srp(2)

Delta, Dpp, En, Hh, Med, Pyr, Shn, Su\_H\_CSL, Ths, Ci, Da, Htl, Twi, Zfh-1, Brk, D-six4, Eya, Srp(2)

Delta, Dpp, Med, Pyr, Shn, Spi, Su\_H\_CSL, Ths, Upd, Wg, Da, Htl, Pan, Slp, Stat92E, Twi(2), Zfh-1, Brk, D-six4, Eya, Hbr, Mef2, Poxm

Delta, En, Hh, Med, Pyr, Su\_H\_CSL, Ths, Ci, Da, Htl, Brk, Srp(2)

Delta, En, Hh, Med, Pyr, Su\_H\_CSL, Ths, Ci, Da, Htl, Twi, Zfh-1, Brk, D-six4, Eya, Srp(2)

Med, Pyr, Su\_H\_CSL, Ths, Wg, Da, Htl, Pan, Slp, Twi(2), Zfh-1, Brk, D-six4, Eya, Hbr, Mef2, Poxm

### Genotype: Med\_1; Pnr\_1

Delta, Dpp, En, Hh, Med, Pyr, Shn, Su\_H\_CSL, Ths, Bap(3), Bin, Ci, Htl, Mad, Nicd, Tin, Mef2, Pnr

Delta, Dpp, Med, Pyr, Shn, Spi, Su\_H\_CSL, Ths, Upd, Wg, Doc, E\_Spl, Htl, Mad, Nicd, Pan, Slp, Stat92E, Tin(2), Twi, Zfh-1, Eve, Eya, Hbr, Mef2, Pnr

Delta, En, Hh, Med, Pyr, Su\_H\_CSL, Ths, Ci, Htl, Nicd, Brk, Pnr, Srp(2)

Med, Pyr, Su\_H\_CSL, Ths, Wg, Da, Htl, Pan, Slp, Twi(2), Zfh-1, Brk, D-six4, Eya, Hbr, Mef2, Pnr, Poxm

### Genotype: Med\_1; Pnr\_0

Delta, Dpp, En, Hh, Med, Pyr, Shn, Su\_H\_CSL, Ths, Bap(3), Bin, Ci, Htl, Mad, Nicd, Tin, Mef2

Delta, Dpp, Med, Pyr, Shn, Spi, Su\_H\_CSL, Ths, Upd, Wg, Doc, E\_Spl, Htl, Mad, Nicd, Pan, Slp, Stat92E, Tin(2), Twi, Zfh-1, Eve, Eya, Hbr, Mef2

Delta, En, Hh, Med, Pyr, Su\_H\_CSL, Ths, Ci, Htl, Nicd, Brk, Srp(2)

Med, Pyr, Su\_H\_CSL, Ths, Wg, Da, Htl, Pan, Slp, Twi(2), Zfh-1, Brk, D-six4, Eya, Hbr, Mef2, Poxm

### Genotype: Nicd\_0; Slp\_0

Delta, Dpp, En, Hh, Med, Pyr, Shn, Su\_H\_CSL, Ths, Bap(3), Bin, Ci, Da, Htl, Mad, Tin, Mef2

Delta, Dpp, En, Hh, Med, Pyr, Shn, Su\_H\_CSL, Ths, Bap(3), Bin, Ci, Da, Htl, Mad, Tin, Twi, Zfh-1, Eya, Mef2

Delta, Dpp, En, Med, Pyr, Shn, Spi, Su\_H\_CSL, Ths, Upd, Wg, Bap, Da, Doc, Htl, Mad, Pan, Stat92E, Tin(2), Eya, Hbr, Mef2, Pnr

Delta, Dpp, En, Med, Pyr, Shn, Spi, Su\_H\_CSL, Ths, Upd, Wg, Bap, Da, Doc, Htl, Mad, Pan, Stat92E, Tin(2), Twi, Zfh-1, Eve, Eya, Hbr, Mef2, Pnr

Delta, En, Hh, Med, Pyr, Su\_H\_CSL, Ths, Ci, Da, Htl, Brk, Srp(2)

Delta, En, Hh, Med, Pyr, Su\_H\_CSL, Ths, Ci, Da, Htl, Twi, Zfh-1, Brk, D-six4, Eya, Srp(2)

En, Med, Pyr, Su\_H\_CSL, Ths, Wg, Da, Htl, Pan, Brk, Hbr

En, Med, Pyr, Su\_H\_CSL, Ths, Wg, Da, Htl, Pan, Twi, Zfh-1, Brk, D-six4, Eya, Hbr, Poxm

### Genotype: Nicd\_0; Slp\_1

Delta, Dpp, Hh, Med, Pyr, Shn, Su\_H\_CSL, Ths, Bap, Ci, Da, Htl, Mad, Slp, Tin, Mef2

Delta, Dpp, Hh, Med, Pyr, Shn, Su\_H\_CSL, Ths, Bap, Ci, Da, Htl, Mad, Slp, Tin, Twi(2), Zfh-1, Eya, Mef2

Delta, Dpp, Med, Pyr, Shn, Spi, Su\_H\_CSL, Ths, Upd, Wg, Da, Doc, Htl, Mad, Pan, Slp, Stat92E, Tin(2), Twi(2), Zfh-1, Eve, Eya, Hbr, Mef2, Pnr

Delta, Hh, Med, Pyr, Su\_H\_CSL, Ths, Ci, Da, Htl, Slp, Brk, Srp

Delta, Hh, Med, Pyr, Su\_H\_CSL, Ths, Ci, Da, Htl, Slp, Twi(2), Zfh-1, Brk, D-six4, Eya, Mef2, Srp

Med, Pyr, Su\_H\_CSL, Ths, Wg, Da, Htl, Pan, Slp, Twi(2), Zfh-1, Brk, D-six4, Eya, Hbr, Mef2, Poxm

### Genotype: Bin\_0; Twi\_0

Delta, Dpp, En, Hh, Med, Pyr, Shn, Su\_H\_CSL, Ths, Bap(3), Ci, Htl, Mad, Nicd, Tin, Mef2

Delta, Dpp, Med, Pyr, Shn, Spi, Su\_H\_CSL, Ths, Upd, Wg, Doc, E\_Spl, Htl, Mad, Nicd, Pan, Slp, Stat92E, Tin(2), Eya, Hbr, Mef2, Pnr

Delta, En, Hh, Med, Pyr, Su\_H\_CSL, Ths, Ci, Htl, Nicd, Brk, Srp(2)

Med, Pyr, Su\_H\_CSL, Ths, Wg, Da, Htl, Pan, Slp, Brk, Hbr

### Genotype: Bin\_0; Twi\_2

Delta, Dpp, En, Hh, Med, Pyr, Shn, Su\_H\_CSL, Ths, Bap(3), Ci, Htl, Mad, Nicd, Tin, Twi(2), Zfh-1, Eya, Mef2

Delta, Dpp, Med, Pyr, Shn, Spi, Su\_H\_CSL, Ths, Upd, Wg, Doc, E\_Spl, Htl, Mad, Nicd, Pan, Slp, Stat92E, Tin(2), Twi(2), Zfh-1, Eve, Eya, Hbr, Mef2, Pnr

Delta, En, Hh, Med, Pyr, Su\_H\_CSL, Ths, Ci, Htl, Nicd, Twi(2), Zfh-1, Brk, D-six4, Eya, Mef2, Srp(2)

Med, Pyr, Su\_H\_CSL, Ths, Wg, Da, Htl, Pan, Slp, Twi(2), Zfh-1, Brk, D-six4, Eya, Hbr, Mef2, Poxm

### Genotype: Med\_0; Pnr\_0

Delta, Dpp, En, Hh, Pyr, Shn, Su\_H\_CSL, Ths, Bap(2), Bin, Ci, Htl, Mad, Nicd, Tin, Brk, D-six4, Mef2, Srp(2)

Delta, Dpp, Pyr, Shn, Spi, Su\_H\_CSL, Ths, Upd, Wg, E\_Spl, Htl, Mad, Nicd, Pan, Slp, Stat92E, Tin, Twi, Zfh-1, Brk, D-six4, Eya, Hbr, Mef2, Poxm

Delta, En, Hh, Pyr, Su\_H\_CSL, Ths, Ci, Htl, Nicd, Brk, Srp(2)

Pyr, Su\_H\_CSL, Ths, Wg, Da, Htl, Pan, Slp, Twi(2), Zfh-1, Brk, D-six4, Eya, Hbr, Mef2, Poxm

### Genotype: Med\_0; Pnr\_1

Delta, Dpp, En, Hh, Pyr, Shn, Su\_H\_CSL, Ths, Bap(2), Bin, Ci, Htl, Mad, Nicd, Tin, Brk, D-six4, Mef2, Pnr, Srp(2)

Delta, Dpp, Pyr, Shn, Spi, Su\_H\_CSL, Ths, Upd, Wg, E\_Spl, Htl, Mad, Nicd, Pan, Slp, Stat92E, Tin, Twi, Zfh-1, Brk, D-six4, Eya, Hbr, Mef2, Pnr, Poxm

Delta, En, Hh, Pyr, Su\_H\_CSL, Ths, Ci, Htl, Nicd, Brk, Pnr, Srp(2)

Pyr, Su\_H\_CSL, Ths, Wg, Da, Htl, Pan, Slp, Twi(2), Zfh-1, Brk, D-six4, Eya, Hbr, Mef2, Pnr, Poxm

### Genotype: Mad\_1; Srp\_0

Delta, Dpp, En, Hh, Med, Pyr, Shn, Su\_H\_CSL, Ths, Bap(3), Bin, Ci, Htl, Mad, Nicd, Tin, Mef2

Delta, Dpp, Med, Pyr, Shn, Spi, Su\_H\_CSL, Ths, Upd, Wg, Doc, E\_Spl, Htl, Mad, Nicd, Pan, Slp, Stat92E, Tin(2), Twi, Zfh-1, Eve, Eya, Hbr, Mef2, Pnr

Delta, En, Hh, Med, Pyr, Su\_H\_CSL, Ths, Bap(3), Bin, Ci, Htl, Mad, Nicd, Tin, Brk, Mef2

Med, Pyr, Su\_H\_CSL, Ths, Wg, Da, Doc, Htl, Mad, Pan, Slp, Tin, Twi(2), Zfh-1, Brk, Eya, Hbr, Mef2, Pnr

### Genotype: Mad\_1; Srp\_2

Delta, Dpp, En, Hh, Med, Pyr, Shn, Su\_H\_CSL, Ths, Bap(3), Bin, Ci, Htl, Mad, Nicd, Tin, Mef2, Srp(2)

Delta, Dpp, Med, Pyr, Shn, Spi, Su\_H\_CSL, Ths, Upd, Wg, Doc, E\_Spl, Htl, Mad, Nicd, Pan, Slp, Stat92E, Tin(2), Twi, Zfh-1, Eve, Eya, Hbr, Mef2, Pnr, Srp(2)

Delta, En, Hh, Med, Pyr, Su\_H\_CSL, Ths, Bap(3), Bin, Ci, Htl, Mad, Nicd, Tin, Brk, Mef2, Srp(2)

Med, Pyr, Su\_H\_CSL, Ths, Wg, Da, Doc, Htl, Mad, Pan, Slp, Tin, Twi(2), Zfh-1, Brk, Eya, Hbr, Mef2, Pnr, Srp(2)

### Genotype: Pan\_0; Slp\_1

Delta, Dpp, Hh, Med, Pyr, Shn, Su\_H\_CSL, Ths, Bap, Ci, Htl, Mad, Nicd, Slp, Tin, Mef2

Delta, Dpp, Hh, Med, Pyr, Shn, Su\_H\_CSL, Ths, Bap, Ci, Htl, Mad, Nicd, Slp, Tin, Twi, Zfh-1, Eya, Mef2

Delta, Dpp, Med, Pyr, Shn, Spi, Su\_H\_CSL, Ths, Upd, Wg, E\_Spl, Htl, Mad, Nicd, Slp, Stat92E, Tin, Twi, Zfh-1, Eya, Mef2

Delta, Hh, Med, Pyr, Su\_H\_CSL, Ths, Ci, Htl, Nicd, Slp, Brk, Srp

Delta, Hh, Med, Pyr, Su\_H\_CSL, Ths, Ci, Htl, Nicd, Slp, Twi, Zfh-1, Brk, D-six4, Eya, Srp

Med, Pyr, Su\_H\_CSL, Ths, Wg, Da, Htl, Slp, Twi(2), Zfh-1, Brk, D-six4, Eya, Mef2

### Genotype: Ci\_0; Slp\_0

Delta, Dpp, En, Hh, Med, Pyr, Shn, Su\_H\_CSL, Ths, Bap, Htl, Mad, Nicd, Tin, Mef2

Delta, Dpp, En, Hh, Med, Pyr, Shn, Su\_H\_CSL, Ths, Bap(2), Bin, Htl, Mad, Nicd, Tin, Mef2

Delta, Dpp, En, Med, Pyr, Shn, Spi, Su\_H\_CSL, Ths, Upd, Wg, Bap, Doc, E\_Spl, Htl, Mad, Nicd, Pan, Stat92E, Tin(2), Eya, Hbr, Mef2, Pnr

Delta, En, Hh, Med, Pyr, Su\_H\_CSL, Ths, Htl, Nicd, Brk

En, Med, Pyr, Su\_H\_CSL, Ths, Wg, Da, Htl, Pan, Brk, Hbr

En, Med, Pyr, Su\_H\_CSL, Ths, Wg, Da, Htl, Pan, Twi, Zfh-1, Brk, D-six4, Eya, Hbr, Poxm

### Genotype: Ci\_0; Slp\_1

Delta, Dpp, Hh, Med, Pyr, Shn, Su\_H\_CSL, Ths, Htl, Mad, Nicd, Slp, Tin, Mef2

Delta, Dpp, Hh, Med, Pyr, Shn, Su\_H\_CSL, Ths, Htl, Mad, Nicd, Slp, Tin, Twi, Zfh-1, Eya, Mef2

Delta, Dpp, Med, Pyr, Shn, Spi, Su\_H\_CSL, Ths, Upd, Wg, Doc, E\_Spl, Htl, Mad, Nicd, Pan, Slp, Stat92E, Tin(2), Twi, Zfh-1, Eve, Eya, Hbr, Mef2, Pnr

Delta, Hh, Med, Pyr, Su\_H\_CSL, Ths, Htl, Nicd, Slp, Brk

Delta, Hh, Med, Pyr, Su\_H\_CSL, Ths, Htl, Nicd, Slp, Twi, Zfh-1, Brk, D-six4, Eya

Med, Pyr, Su\_H\_CSL, Ths, Wg, Da, Htl, Pan, Slp, Twi(2), Zfh-1, Brk, D-six4, Eya, Hbr, Mef2, Poxm

### Genotype: Pnr\_0; Twi\_2

Delta, Dpp, En, Hh, Med, Pyr, Shn, Su\_H\_CSL, Ths, Bap(3), Bin, Ci, Htl, Mad, Nicd, Tin, Twi(2), Zfh-1, Eya, Mef2

Delta, Dpp, Med, Pyr, Shn, Spi, Su\_H\_CSL, Ths, Upd, Wg, Doc, E\_Spl, Htl, Mad, Nicd, Pan, Slp, Stat92E, Tin(2), Twi(2), Zfh-1, Eve, Eya, Hbr, Mef2

Delta, En, Hh, Med, Pyr, Su\_H\_CSL, Ths, Ci, Htl, Nicd, Twi(2), Zfh-1, Brk, D-six4, Eya, Mef2, Srp(2)

Med, Pyr, Su\_H\_CSL, Ths, Wg, Da, Htl, Pan, Slp, Twi(2), Zfh-1, Brk, D-six4, Eya, Hbr, Mef2, Poxm

### Genotype: Pnr\_0; Twi\_0

Delta, Dpp, En, Hh, Med, Pyr, Shn, Su\_H\_CSL, Ths, Bap(3), Bin, Ci, Htl, Mad, Nicd, Tin, Mef2

Delta, Dpp, Med, Pyr, Shn, Spi, Su\_H\_CSL, Ths, Upd, Wg, Doc, E\_Spl, Htl, Mad, Nicd, Pan, Slp, Stat92E, Tin(2), Eya, Hbr, Mef2

Delta, En, Hh, Med, Pyr, Su\_H\_CSL, Ths, Ci, Htl, Nicd, Brk, Srp(2)

Med, Pyr, Su\_H\_CSL, Ths, Wg, Da, Htl, Pan, Slp, Brk, Hbr

### Genotype: Nicd\_0; Pnr\_0

Delta, Dpp, En, Hh, Med, Pyr, Shn, Su\_H\_CSL, Ths, Bap(3), Bin, Ci, Da, Htl, Mad, Tin, Mef2

Delta, Dpp, En, Hh, Med, Pyr, Shn, Su\_H\_CSL, Ths, Bap(3), Bin, Ci, Da, Htl, Mad, Tin, Twi, Zfh-1, Eya, Mef2

Delta, Dpp, Med, Pyr, Shn, Spi, Su\_H\_CSL, Ths, Upd, Wg, Da, Doc, Htl, Mad, Pan, Slp, Stat92E, Tin(2), Twi(2), Zfh-1, Eve, Eya, Hbr, Mef2

Delta, En, Hh, Med, Pyr, Su\_H\_CSL, Ths, Ci, Da, Htl, Brk, Srp(2)

Delta, En, Hh, Med, Pyr, Su\_H\_CSL, Ths, Ci, Da, Htl, Twi, Zfh-1, Brk, D-six4, Eya, Srp(2)

Med, Pyr, Su\_H\_CSL, Ths, Wg, Da, Htl, Pan, Slp, Twi(2), Zfh-1, Brk, D-six4, Eya, Hbr, Mef2, Poxm

### Genotype: Nicd\_0; Tin\_2

Delta, Dpp, En, Hh, Med, Pyr, Shn, Su\_H\_CSL, Ths, Bap(3), Bin, Ci, Da, Htl, Mad, Stat92E, Tin(2), Eya, Mef2

Delta, Dpp, En, Hh, Med, Pyr, Shn, Su\_H\_CSL, Ths, Bap(3), Bin, Ci, Da, Htl, Mad, Stat92E, Tin(2), Twi, Zfh-1, Eya, Mef2

Delta, Dpp, Med, Pyr, Shn, Spi, Su\_H\_CSL, Ths, Upd, Wg, Da, Doc, Htl, Mad, Pan, Slp, Stat92E, Tin(2), Twi(2), Zfh-1, Eve, Eya, Hbr, Mef2, Pnr

Delta, En, Hh, Med, Pyr, Su\_H\_CSL, Ths, Bap(2), Bin, Ci, Da, Htl, Stat92E, Tin(2), Brk, D-six4, Eya, Mef2, Srp(2)

Delta, En, Hh, Med, Pyr, Su\_H\_CSL, Ths, Bap(2), Bin, Ci, Da, Htl, Stat92E, Tin(2), Twi, Zfh-1, Brk, D-six4, Eya, Mef2, Srp(2)

Med, Pyr, Su\_H\_CSL, Ths, Wg, Da, Htl, Pan, Slp, Stat92E, Tin(2), Twi(2), Zfh-1, Brk, D-six4, Eya, Hbr, Mef2, Poxm

### Genotype: Mef2\_1; Tin\_2

Delta, Dpp, En, Hh, Med, Pyr, Shn, Su\_H\_CSL, Ths, Bap(3), Bin, Ci, E\_Spl, Htl, Mad, Nicd, Stat92E, Tin(2), Eya, Mef2

Delta, Dpp, Med, Pyr, Shn, Spi, Su\_H\_CSL, Ths, Upd, Wg, Doc, E\_Spl, Htl, Mad, Nicd, Pan, Slp, Stat92E, Tin(2), Twi, Zfh-1, Eve, Eya, Hbr, Mef2, Pnr

Delta, En, Hh, Med, Pyr, Su\_H\_CSL, Ths, Bap(2), Bin, Ci, E\_Spl, Htl, Nicd, Stat92E, Tin(2), Brk, D-six4, Eya, Mef2, Srp(2)

Med, Pyr, Su\_H\_CSL, Ths, Wg, Da, Htl, Pan, Slp, Stat92E, Tin(2), Twi(2), Zfh-1, Brk, D-six4, Eya, Hbr, Mef2, Poxm

### Genotype: Mef2\_1; Tin\_0

Delta, Dpp, En, Hh, Med, Pyr, Shn, Su\_H\_CSL, Ths, Ci, Htl, Mad, Nicd, Mef2

Delta, Dpp, Med, Pyr, Shn, Spi, Su\_H\_CSL, Ths, Upd, Wg, Doc, E\_Spl, Htl, Mad, Nicd, Pan, Slp, Stat92E, Twi, Zfh-1, Eya, Hbr, Mef2

Delta, En, Hh, Med, Pyr, Su\_H\_CSL, Ths, Ci, Htl, Nicd, Brk, Mef2, Srp(2)

Med, Pyr, Su\_H\_CSL, Ths, Wg, Da, Htl, Pan, Slp, Twi(2), Zfh-1, Brk, D-six4, Eya, Hbr, Mef2, Poxm

### Genotype: Ci\_0; Mad\_1

Delta, Dpp, En, Hh, Med, Pyr, Shn, Su\_H\_CSL, Ths, Bap, Htl, Mad, Nicd, Tin, Mef2

Delta, Dpp, En, Hh, Med, Pyr, Shn, Su\_H\_CSL, Ths, Bap(2), Bin, Htl, Mad, Nicd, Tin, Mef2

Delta, Dpp, Med, Pyr, Shn, Spi, Su\_H\_CSL, Ths, Upd, Wg, Doc, E\_Spl, Htl, Mad, Nicd, Pan, Slp, Stat92E, Tin(2), Twi, Zfh-1, Eve, Eya, Hbr, Mef2, Pnr

Delta, En, Hh, Med, Pyr, Su\_H\_CSL, Ths, Bap, Htl, Mad, Nicd, Tin, Brk, Mef2

Delta, En, Hh, Med, Pyr, Su\_H\_CSL, Ths, Bap(2), Bin, Htl, Mad, Nicd, Tin, Brk, Mef2

Med, Pyr, Su\_H\_CSL, Ths, Wg, Da, Doc, Htl, Mad, Pan, Slp, Tin, Twi(2), Zfh-1, Brk, Eya, Hbr, Mef2, Pnr

### Genotype: Ci\_0; Mad\_0

Delta, Dpp, En, Hh, Med, Pyr, Shn, Su\_H\_CSL, Ths, Htl, Nicd, Brk

Delta, Dpp, Med, Pyr, Shn, Spi, Su\_H\_CSL, Ths, Upd, Wg, E\_Spl, Htl, Nicd, Pan, Slp, Stat92E, Twi, Zfh-1, Brk, D-six4, Eya, Hbr, Poxm

Delta, En, Hh, Med, Pyr, Su\_H\_CSL, Ths, Htl, Nicd, Brk

Med, Pyr, Su\_H\_CSL, Ths, Wg, Da, Htl, Pan, Slp, Twi(2), Zfh-1, Brk, D-six4, Eya, Hbr, Mef2, Poxm

### Genotype: Bap\_0; Pan\_0

Delta, Dpp, En, Hh, Med, Pyr, Shn, Su\_H\_CSL, Ths, Ci, Htl, Mad, Nicd, Tin, Mef2

Delta, Dpp, Med, Pyr, Shn, Spi, Su\_H\_CSL, Ths, Upd, Wg, E\_Spl, Htl, Mad, Nicd, Stat92E, Tin, Mef2

Delta, En, Hh, Med, Pyr, Su\_H\_CSL, Ths, Ci, Htl, Nicd, Brk, Srp(2)

Med, Pyr, Su\_H\_CSL, Ths, Wg, Da, Htl, Brk

Med, Pyr, Su\_H\_CSL, Ths, Wg, Da, Htl, Twi, Zfh-1, Brk, D-six4, Eya

### Genotype: Bap\_0; Pan\_1

Delta, Dpp, En, Hh, Med, Pyr, Shn, Su\_H\_CSL, Ths, Ci, Doc, Htl, Mad, Nicd, Pan, Slp, Tin, Hbr, Mef2, Pnr

Delta, Dpp, En, Hh, Med, Pyr, Shn, Su\_H\_CSL, Ths, Ci, Doc, Htl, Mad, Nicd, Pan, Slp, Tin, Twi, Zfh-1, Eya, Hbr, Mef2, Pnr

Delta, Dpp, Med, Pyr, Shn, Spi, Su\_H\_CSL, Ths, Upd, Wg, Doc, E\_Spl, Htl, Mad, Nicd, Pan, Slp, Stat92E, Tin(2), Twi, Zfh-1, Eve, Eya, Hbr, Mef2, Pnr

Delta, En, Hh, Med, Pyr, Su\_H\_CSL, Ths, Ci, Htl, Nicd, Pan, Slp, Brk, Hbr, Srp(2)

Delta, En, Hh, Med, Pyr, Su\_H\_CSL, Ths, Ci, Htl, Nicd, Pan, Slp, Twi, Zfh-1, Brk, D-six4, Eya, Hbr, Poxm, Srp(2)

Med, Pyr, Su\_H\_CSL, Ths, Wg, Da, Htl, Pan, Slp, Twi(2), Zfh-1, Brk, D-six4, Eya, Hbr, Mef2, Poxm

### Genotype: Bap\_0; Pnr\_1

Delta, Dpp, En, Hh, Med, Pyr, Shn, Su\_H\_CSL, Ths, Ci, Htl, Mad, Nicd, Tin, Mef2, Pnr

Delta, Dpp, Med, Pyr, Shn, Spi, Su\_H\_CSL, Ths, Upd, Wg, Doc, E\_Spl, Htl, Mad, Nicd, Pan, Slp, Stat92E, Tin(2), Twi, Zfh-1, Eve, Eya, Hbr, Mef2, Pnr

Delta, En, Hh, Med, Pyr, Su\_H\_CSL, Ths, Ci, Htl, Nicd, Brk, Pnr, Srp(2)

Med, Pyr, Su\_H\_CSL, Ths, Wg, Da, Htl, Pan, Slp, Twi(2), Zfh-1, Brk, D-six4, Eya, Hbr, Mef2, Pnr, Poxm

### Genotype: Bap\_0; Pnr\_0

Delta, Dpp, En, Hh, Med, Pyr, Shn, Su\_H\_CSL, Ths, Ci, Htl, Mad, Nicd, Tin, Mef2

Delta, Dpp, Med, Pyr, Shn, Spi, Su\_H\_CSL, Ths, Upd, Wg, Doc, E\_Spl, Htl, Mad, Nicd, Pan, Slp, Stat92E, Tin(2), Twi, Zfh-1, Eve, Eya, Hbr, Mef2

Delta, En, Hh, Med, Pyr, Su\_H\_CSL, Ths, Ci, Htl, Nicd, Brk, Srp(2)

Med, Pyr, Su\_H\_CSL, Ths, Wg, Da, Htl, Pan, Slp, Twi(2), Zfh-1, Brk, D-six4, Eya, Hbr, Mef2, Poxm

### Genotype: Bap\_3; Srp\_0

Delta, Dpp, En, Hh, Med, Pyr, Shn, Su\_H\_CSL, Ths, Bap(3), Bin, Ci, Htl, Mad, Nicd, Tin, Mef2

Delta, Dpp, Med, Pyr, Shn, Spi, Su\_H\_CSL, Ths, Upd, Wg, Bap(3), Bin, Doc, E\_Spl, Htl, Mad, Nicd, Pan, Slp, Stat92E, Tin(2), Twi, Zfh-1, Eve, Eya, Hbr, Mef2, Pnr

Delta, En, Hh, Med, Pyr, Su\_H\_CSL, Ths, Bap(3), Bin, Ci, Htl, Nicd, Brk

Med, Pyr, Su\_H\_CSL, Ths, Wg, Bap(3), Bin, Da, Htl, Pan, Slp, Twi(2), Zfh-1, Brk, D-six4, Eya, Hbr, Mef2, Poxm

### Genotype: Bap\_3; Srp\_2

Delta, Dpp, En, Hh, Med, Pyr, Shn, Su\_H\_CSL, Ths, Bap(3), Bin, Ci, Htl, Mad, Nicd, Tin, Mef2, Srp(2)

Delta, Dpp, Med, Pyr, Shn, Spi, Su\_H\_CSL, Ths, Upd, Wg, Bap(3), Bin, Doc, E\_Spl, Htl, Mad, Nicd, Pan, Slp, Stat92E, Tin(2), Twi, Zfh-1, Eve, Eya, Hbr, Mef2, Pnr, Srp(2)

Delta, En, Hh, Med, Pyr, Su\_H\_CSL, Ths, Bap(3), Bin, Ci, Htl, Nicd, Brk, Srp(2)

Med, Pyr, Su\_H\_CSL, Ths, Wg, Bap(3), Bin, Da, Htl, Pan, Slp, Twi(2), Zfh-1, Brk, D-six4, Eya, Hbr, Mef2, Poxm, Srp(2)

### Genotype: Slp\_1; Tin\_0

Delta, Dpp, Hh, Med, Pyr, Shn, Su\_H\_CSL, Ths, Ci, Htl, Mad, Nicd, Slp

Delta, Dpp, Hh, Med, Pyr, Shn, Su\_H\_CSL, Ths, Ci, Htl, Mad, Nicd, Slp, Twi, Zfh-1, Eya

Delta, Dpp, Med, Pyr, Shn, Spi, Su\_H\_CSL, Ths, Upd, Wg, Doc, E\_Spl, Htl, Mad, Nicd, Pan, Slp, Stat92E, Twi, Zfh-1, Eya, Hbr

Delta, Hh, Med, Pyr, Su\_H\_CSL, Ths, Ci, Htl, Nicd, Slp, Brk, Srp

Delta, Hh, Med, Pyr, Su\_H\_CSL, Ths, Ci, Htl, Nicd, Slp, Twi, Zfh-1, Brk, D-six4, Eya, Srp

Med, Pyr, Su\_H\_CSL, Ths, Wg, Da, Htl, Pan, Slp, Twi(2), Zfh-1, Brk, D-six4, Eya, Hbr, Mef2, Poxm

### Genotype: Slp\_1; Tin\_2

Delta, Dpp, Hh, Med, Pyr, Shn, Su\_H\_CSL, Ths, Bap, Ci, E\_Spl, Htl, Mad, Nicd, Slp, Stat92E, Tin(2), Eya, Mef2

Delta, Dpp, Hh, Med, Pyr, Shn, Su\_H\_CSL, Ths, Bap, Ci, E\_Spl, Htl, Mad, Nicd, Slp, Stat92E, Tin(2), Twi, Zfh-1, Eya, Mef2

Delta, Dpp, Med, Pyr, Shn, Spi, Su\_H\_CSL, Ths, Upd, Wg, Doc, E\_Spl, Htl, Mad, Nicd, Pan, Slp, Stat92E, Tin(2), Twi, Zfh-1, Eve, Eya, Hbr, Mef2, Pnr

Delta, Hh, Med, Pyr, Su\_H\_CSL, Ths, Ci, E\_Spl, Htl, Nicd, Slp, Stat92E, Tin(2), Brk, D-six4, Eya, Mef2, Srp

Delta, Hh, Med, Pyr, Su\_H\_CSL, Ths, Ci, E\_Spl, Htl, Nicd, Slp, Stat92E, Tin(2), Twi, Zfh-1, Brk, D-six4, Eya, Mef2, Srp

Med, Pyr, Su\_H\_CSL, Ths, Wg, Da, Htl, Pan, Slp, Stat92E, Tin(2), Twi(2), Zfh-1, Brk, D-six4, Eya, Hbr, Mef2, Poxm

### Genotype: Med\_1; Pan\_0

Delta, Dpp, En, Hh, Med, Pyr, Shn, Su\_H\_CSL, Ths, Bap(3), Bin, Ci, Htl, Mad, Nicd, Tin, Mef2

Delta, Dpp, Med, Pyr, Shn, Spi, Su\_H\_CSL, Ths, Upd, Wg, Bap, E\_Spl, Htl, Mad, Nicd, Stat92E, Tin, Mef2

Delta, En, Hh, Med, Pyr, Su\_H\_CSL, Ths, Ci, Htl, Nicd, Brk, Srp(2)

Med, Pyr, Su\_H\_CSL, Ths, Wg, Da, Htl, Brk

Med, Pyr, Su\_H\_CSL, Ths, Wg, Da, Htl, Twi, Zfh-1, Brk, D-six4, Eya

### Genotype: Med\_1; Pan\_1

Delta, Dpp, En, Hh, Med, Pyr, Shn, Su\_H\_CSL, Ths, Bap, Ci, Doc, Htl, Mad, Nicd, Pan, Slp, Tin, Hbr, Mef2, Pnr

Delta, Dpp, En, Hh, Med, Pyr, Shn, Su\_H\_CSL, Ths, Bap, Ci, Doc, Htl, Mad, Nicd, Pan, Slp, Tin, Twi, Zfh-1, Eya, Hbr, Mef2, Pnr

Delta, Dpp, Med, Pyr, Shn, Spi, Su\_H\_CSL, Ths, Upd, Wg, Doc, E\_Spl, Htl, Mad, Nicd, Pan, Slp, Stat92E, Tin(2), Twi, Zfh-1, Eve, Eya, Hbr, Mef2, Pnr

Delta, En, Hh, Med, Pyr, Su\_H\_CSL, Ths, Ci, Htl, Nicd, Pan, Slp, Brk, Hbr, Srp(2)

Delta, En, Hh, Med, Pyr, Su\_H\_CSL, Ths, Ci, Htl, Nicd, Pan, Slp, Twi, Zfh-1, Brk, D-six4, Eya, Hbr, Poxm, Srp(2)

Med, Pyr, Su\_H\_CSL, Ths, Wg, Da, Htl, Pan, Slp, Twi(2), Zfh-1, Brk, D-six4, Eya, Hbr, Mef2, Poxm

### Genotype: Bap\_0; Slp\_0

Delta, Dpp, En, Hh, Med, Pyr, Shn, Su\_H\_CSL, Ths, Ci, Htl, Mad, Nicd, Tin, Mef2

Delta, Dpp, En, Med, Pyr, Shn, Spi, Su\_H\_CSL, Ths, Upd, Wg, Doc, E\_Spl, Htl, Mad, Nicd, Pan, Stat92E, Tin(2), Eya, Hbr, Mef2, Pnr

Delta, En, Hh, Med, Pyr, Su\_H\_CSL, Ths, Ci, Htl, Nicd, Brk, Srp(2)

En, Med, Pyr, Su\_H\_CSL, Ths, Wg, Da, Htl, Pan, Brk, Hbr

En, Med, Pyr, Su\_H\_CSL, Ths, Wg, Da, Htl, Pan, Twi, Zfh-1, Brk, D-six4, Eya, Hbr, Poxm

### Genotype: Bap\_0; Slp\_1

Delta, Dpp, Hh, Med, Pyr, Shn, Su\_H\_CSL, Ths, Ci, Htl, Mad, Nicd, Slp, Tin, Mef2

Delta, Dpp, Hh, Med, Pyr, Shn, Su\_H\_CSL, Ths, Ci, Htl, Mad, Nicd, Slp, Tin, Twi, Zfh-1, Eya, Mef2

Delta, Dpp, Med, Pyr, Shn, Spi, Su\_H\_CSL, Ths, Upd, Wg, Doc, E\_Spl, Htl, Mad, Nicd, Pan, Slp, Stat92E, Tin(2), Twi, Zfh-1, Eve, Eya, Hbr, Mef2, Pnr

Delta, Hh, Med, Pyr, Su\_H\_CSL, Ths, Ci, Htl, Nicd, Slp, Brk, Srp

Delta, Hh, Med, Pyr, Su\_H\_CSL, Ths, Ci, Htl, Nicd, Slp, Twi, Zfh-1, Brk, D-six4, Eya, Srp

Med, Pyr, Su\_H\_CSL, Ths, Wg, Da, Htl, Pan, Slp, Twi(2), Zfh-1, Brk, D-six4, Eya, Hbr, Mef2, Poxm

### Genotype: Bin\_2; Tin\_2

Delta, Dpp, En, Hh, Med, Pyr, Shn, Su\_H\_CSL, Ths, Bap(3), Bin(2), Ci, E\_Spl, Htl, Mad, Nicd, Stat92E, Tin(2), Eya, Mef2

Delta, Dpp, Med, Pyr, Shn, Spi, Su\_H\_CSL, Ths, Upd, Wg, Bap(3), Bin(2), Doc, E\_Spl, Htl, Mad, Nicd, Pan, Slp, Stat92E, Tin(2), Twi, Zfh-1, Eve, Eya, Hbr, Mef2, Pnr

Delta, En, Hh, Med, Pyr, Su\_H\_CSL, Ths, Bap(3), Bin(2), Ci, E\_Spl, Htl, Nicd, Stat92E, Tin(2), Brk, D-six4, Eya, Mef2, Srp(2)

Med, Pyr, Su\_H\_CSL, Ths, Wg, Bap(3), Bin(2), Da, Htl, Pan, Slp, Stat92E, Tin(2), Twi(2), Zfh-1, Brk, D-six4, Eya, Hbr, Mef2, Poxm

### Genotype: Bin\_2; Tin\_0

Delta, Dpp, En, Hh, Med, Pyr, Shn, Su\_H\_CSL, Ths, Bap(3), Bin(2), Ci, Htl, Mad, Nicd

Delta, Dpp, Med, Pyr, Shn, Spi, Su\_H\_CSL, Ths, Upd, Wg, Bap(3), Bin(2), Doc, E\_Spl, Htl, Mad, Nicd, Pan, Slp, Stat92E, Twi, Zfh-1, Eya, Hbr

Delta, En, Hh, Med, Pyr, Su\_H\_CSL, Ths, Bap(3), Bin(2), Ci, Htl, Nicd, Brk, Srp(2)

Med, Pyr, Su\_H\_CSL, Ths, Wg, Bap(3), Bin(2), Da, Htl, Pan, Slp, Twi(2), Zfh-1, Brk, D-six4, Eya, Hbr, Mef2, Poxm

### Genotype: Med\_0; Pan\_1

Delta, Dpp, En, Hh, Pyr, Shn, Su\_H\_CSL, Ths, Bap, Ci, Htl, Mad, Nicd, Pan, Slp, Tin, Brk, D-six4, Hbr, Mef2, Srp(2)

Delta, Dpp, En, Hh, Pyr, Shn, Su\_H\_CSL, Ths, Bap, Ci, Htl, Mad, Nicd, Pan, Slp, Tin, Twi, Zfh-1, Brk, D-six4, Eya, Hbr, Mef2, Poxm, Srp(2)

Delta, Dpp, Pyr, Shn, Spi, Su\_H\_CSL, Ths, Upd, Wg, E\_Spl, Htl, Mad, Nicd, Pan, Slp, Stat92E, Tin, Twi, Zfh-1, Brk, D-six4, Eya, Hbr, Mef2, Poxm

Delta, En, Hh, Pyr, Su\_H\_CSL, Ths, Ci, Htl, Nicd, Pan, Slp, Brk, Hbr, Srp(2)

Delta, En, Hh, Pyr, Su\_H\_CSL, Ths, Ci, Htl, Nicd, Pan, Slp, Twi, Zfh-1, Brk, D-six4, Eya, Hbr, Poxm, Srp(2)

Pyr, Su\_H\_CSL, Ths, Wg, Da, Htl, Pan, Slp, Twi(2), Zfh-1, Brk, D-six4, Eya, Hbr, Mef2, Poxm

### Genotype: Med\_0; Pan\_0

Delta, Dpp, En, Hh, Pyr, Shn, Su\_H\_CSL, Ths, Bap(2), Bin, Ci, Htl, Mad, Nicd, Tin, Brk, D-six4, Mef2, Srp(2)

Delta, Dpp, Pyr, Shn, Spi, Su\_H\_CSL, Ths, Upd, Wg, E\_Spl, Htl, Mad, Nicd, Stat92E, Tin, Brk, D-six4, Mef2

Delta, En, Hh, Pyr, Su\_H\_CSL, Ths, Ci, Htl, Nicd, Brk, Srp(2)

Pyr, Su\_H\_CSL, Ths, Wg, Da, Htl, Brk

Pyr, Su\_H\_CSL, Ths, Wg, Da, Htl, Twi, Zfh-1, Brk, D-six4, Eya

### Genotype: Ci\_1; Slp\_1

Delta, Dpp, Hh, Med, Pyr, Shn, Su\_H\_CSL, Ths, Bap, Ci, Htl, Mad, Nicd, Slp, Tin, Mef2

Delta, Dpp, Hh, Med, Pyr, Shn, Su\_H\_CSL, Ths, Bap, Ci, Htl, Mad, Nicd, Slp, Tin, Twi, Zfh-1, Eya, Mef2

Delta, Dpp, Med, Pyr, Shn, Spi, Su\_H\_CSL, Ths, Upd, Wg, Bap, Ci, Doc, E\_Spl, Htl, Mad, Nicd, Pan, Slp, Stat92E, Tin(2), Twi, Zfh-1, Eve, Eya, Hbr, Mef2, Pnr

Delta, Hh, Med, Pyr, Su\_H\_CSL, Ths, Ci, Htl, Nicd, Slp, Brk, Srp

Delta, Hh, Med, Pyr, Su\_H\_CSL, Ths, Ci, Htl, Nicd, Slp, Twi, Zfh-1, Brk, D-six4, Eya, Srp

Med, Pyr, Su\_H\_CSL, Ths, Wg, Ci, Da, Htl, Pan, Slp, Twi(2), Zfh-1, Brk, D-six4, Eya, Hbr, Mef2, Poxm, Srp

### Genotype: Ci\_1; Slp\_0

Delta, Dpp, En, Hh, Med, Pyr, Shn, Su\_H\_CSL, Ths, Bap(3), Bin, Ci, Htl, Mad, Nicd, Tin, Mef2

Delta, Dpp, En, Med, Pyr, Shn, Spi, Su\_H\_CSL, Ths, Upd, Wg, Bap(3), Bin, Ci, Doc, E\_Spl, Htl, Mad, Nicd, Pan, Stat92E, Tin(2), Eya, Hbr, Mef2, Pnr

Delta, En, Hh, Med, Pyr, Su\_H\_CSL, Ths, Ci, Htl, Nicd, Brk, Srp(2)

En, Med, Pyr, Su\_H\_CSL, Ths, Wg, Ci, Da, Htl, Pan, Brk, Hbr, Srp(2)

En, Med, Pyr, Su\_H\_CSL, Ths, Wg, Ci, Da, Htl, Pan, Twi, Zfh-1, Brk, D-six4, Eya, Hbr, Poxm, Srp(2)

### Genotype: Mef2\_1; Srp\_2

Delta, Dpp, En, Hh, Med, Pyr, Shn, Su\_H\_CSL, Ths, Bap(3), Bin, Ci, Htl, Mad, Nicd, Tin, Mef2, Srp(2)

Delta, Dpp, Med, Pyr, Shn, Spi, Su\_H\_CSL, Ths, Upd, Wg, Doc, E\_Spl, Htl, Mad, Nicd, Pan, Slp, Stat92E, Tin(2), Twi, Zfh-1, Eve, Eya, Hbr, Mef2, Pnr, Srp(2)

Delta, En, Hh, Med, Pyr, Su\_H\_CSL, Ths, Ci, Htl, Nicd, Brk, Mef2, Srp(2)

Med, Pyr, Su\_H\_CSL, Ths, Wg, Da, Htl, Pan, Slp, Twi(2), Zfh-1, Brk, D-six4, Eya, Hbr, Mef2, Poxm, Srp(2)

### Genotype: Mef2\_1; Srp\_0

Delta, Dpp, En, Hh, Med, Pyr, Shn, Su\_H\_CSL, Ths, Bap(3), Bin, Ci, Htl, Mad, Nicd, Tin, Mef2

Delta, Dpp, Med, Pyr, Shn, Spi, Su\_H\_CSL, Ths, Upd, Wg, Doc, E\_Spl, Htl, Mad, Nicd, Pan, Slp, Stat92E, Tin(2), Twi, Zfh-1, Eve, Eya, Hbr, Mef2, Pnr

Delta, En, Hh, Med, Pyr, Su\_H\_CSL, Ths, Ci, Htl, Nicd, Brk, Mef2

Med, Pyr, Su\_H\_CSL, Ths, Wg, Da, Htl, Pan, Slp, Twi(2), Zfh-1, Brk, D-six4, Eya, Hbr, Mef2, Poxm

### Genotype: Mef2\_0; Srp\_2

Delta, Dpp, En, Hh, Med, Pyr, Shn, Su\_H\_CSL, Ths, Bap(3), Bin, Ci, Htl, Mad, Nicd, Tin, Srp(2)

Delta, Dpp, Med, Pyr, Shn, Spi, Su\_H\_CSL, Ths, Upd, Wg, Doc, E\_Spl, Htl, Mad, Nicd, Pan, Slp, Stat92E, Tin(2), Twi, Zfh-1, Eve, Eya, Hbr, Pnr, Srp(2)

Delta, En, Hh, Med, Pyr, Su\_H\_CSL, Ths, Ci, Htl, Nicd, Brk, Srp(2)

Med, Pyr, Su\_H\_CSL, Ths, Wg, Da, Htl, Pan, Slp, Twi(2), Zfh-1, Brk, D-six4, Eya, Hbr, Poxm, Srp(2)

### Genotype: Mef2\_0; Srp\_0

Delta, Dpp, En, Hh, Med, Pyr, Shn, Su\_H\_CSL, Ths, Bap(3), Bin, Ci, Htl, Mad, Nicd, Tin

Delta, Dpp, Med, Pyr, Shn, Spi, Su\_H\_CSL, Ths, Upd, Wg, Doc, E\_Spl, Htl, Mad, Nicd, Pan, Slp, Stat92E, Tin(2), Twi, Zfh-1, Eve, Eya, Hbr, Pnr

Delta, En, Hh, Med, Pyr, Su\_H\_CSL, Ths, Ci, Htl, Nicd, Brk

Med, Pyr, Su\_H\_CSL, Ths, Wg, Da, Htl, Pan, Slp, Twi(2), Zfh-1, Brk, D-six4, Eya, Hbr, Poxm

### Genotype: Bin\_2; Pnr\_1

Delta, Dpp, En, Hh, Med, Pyr, Shn, Su\_H\_CSL, Ths, Bap(3), Bin(2), Ci, Htl, Mad, Nicd, Tin, Mef2, Pnr

Delta, Dpp, Med, Pyr, Shn, Spi, Su\_H\_CSL, Ths, Upd, Wg, Bap(3), Bin(2), Doc, E\_Spl, Htl, Mad, Nicd, Pan, Slp, Stat92E, Tin(2), Twi, Zfh-1, Eve, Eya, Hbr, Mef2, Pnr

Delta, En, Hh, Med, Pyr, Su\_H\_CSL, Ths, Bap(3), Bin(2), Ci, Htl, Nicd, Brk, Pnr, Srp(2)

Med, Pyr, Su\_H\_CSL, Ths, Wg, Bap(3), Bin(2), Da, Htl, Pan, Slp, Twi(2), Zfh-1, Brk, D-six4, Eya, Hbr, Mef2, Pnr, Poxm

### Genotype: Bin\_2; Pnr\_0

Delta, Dpp, En, Hh, Med, Pyr, Shn, Su\_H\_CSL, Ths, Bap(3), Bin(2), Ci, Htl, Mad, Nicd, Tin, Mef2

Delta, Dpp, Med, Pyr, Shn, Spi, Su\_H\_CSL, Ths, Upd, Wg, Bap(3), Bin(2), Doc, E\_Spl, Htl, Mad, Nicd, Pan, Slp, Stat92E, Tin(2), Twi, Zfh-1, Eve, Eya, Hbr, Mef2

Delta, En, Hh, Med, Pyr, Su\_H\_CSL, Ths, Bap(3), Bin(2), Ci, Htl, Nicd, Brk, Srp(2)

Med, Pyr, Su\_H\_CSL, Ths, Wg, Bap(3), Bin(2), Da, Htl, Pan, Slp, Twi(2), Zfh-1, Brk, D-six4, Eya, Hbr, Mef2, Poxm

### Genotype: Ci\_0; Med\_1

Delta, Dpp, En, Hh, Med, Pyr, Shn, Su\_H\_CSL, Ths, Bap, Htl, Mad, Nicd, Tin, Mef2

Delta, Dpp, En, Hh, Med, Pyr, Shn, Su\_H\_CSL, Ths, Bap(2), Bin, Htl, Mad, Nicd, Tin, Mef2

Delta, Dpp, Med, Pyr, Shn, Spi, Su\_H\_CSL, Ths, Upd, Wg, Doc, E\_Spl, Htl, Mad, Nicd, Pan, Slp, Stat92E, Tin(2), Twi, Zfh-1, Eve, Eya, Hbr, Mef2, Pnr

Delta, En, Hh, Med, Pyr, Su\_H\_CSL, Ths, Htl, Nicd, Brk

Med, Pyr, Su\_H\_CSL, Ths, Wg, Da, Htl, Pan, Slp, Twi(2), Zfh-1, Brk, D-six4, Eya, Hbr, Mef2, Poxm

### Genotype: Ci\_0; Med\_0

Delta, Dpp, En, Hh, Pyr, Shn, Su\_H\_CSL, Ths, Htl, Mad, Nicd, Tin, Brk, D-six4, Mef2

Delta, Dpp, Pyr, Shn, Spi, Su\_H\_CSL, Ths, Upd, Wg, E\_Spl, Htl, Mad, Nicd, Pan, Slp, Stat92E, Tin, Twi, Zfh-1, Brk, D-six4, Eya, Hbr, Mef2, Poxm

Delta, En, Hh, Pyr, Su\_H\_CSL, Ths, Htl, Nicd, Brk

Pyr, Su\_H\_CSL, Ths, Wg, Da, Htl, Pan, Slp, Twi(2), Zfh-1, Brk, D-six4, Eya, Hbr, Mef2, Poxm

### Genotype: Bin\_2

Delta, Dpp, En, Hh, Med, Pyr, Shn, Su\_H\_CSL, Ths, Bap(3), Bin(2), Ci, Htl, Mad, Nicd, Tin, Mef2

Delta, Dpp, Med, Pyr, Shn, Spi, Su\_H\_CSL, Ths, Upd, Wg, Bap(3), Bin(2), Doc, E\_Spl, Htl, Mad, Nicd, Pan, Slp, Stat92E, Tin(2), Twi, Zfh-1, Eve, Eya, Hbr, Mef2, Pnr

Delta, En, Hh, Med, Pyr, Su\_H\_CSL, Ths, Bap(3), Bin(2), Ci, Htl, Nicd, Brk, Srp(2)

Med, Pyr, Su\_H\_CSL, Ths, Wg, Bap(3), Bin(2), Da, Htl, Pan, Slp, Twi(2), Zfh-1, Brk, D-six4, Eya, Hbr, Mef2, Poxm

### Genotype: Pnr\_1; Slp\_0

Delta, Dpp, En, Hh, Med, Pyr, Shn, Su\_H\_CSL, Ths, Bap(3), Bin, Ci, Htl, Mad, Nicd, Tin, Mef2, Pnr

Delta, Dpp, En, Med, Pyr, Shn, Spi, Su\_H\_CSL, Ths, Upd, Wg, Bap, Doc, E\_Spl, Htl, Mad, Nicd, Pan, Stat92E, Tin(2), Eya, Hbr, Mef2, Pnr

Delta, En, Hh, Med, Pyr, Su\_H\_CSL, Ths, Ci, Htl, Nicd, Brk, Pnr, Srp(2)

En, Med, Pyr, Su\_H\_CSL, Ths, Wg, Da, Htl, Pan, Brk, Hbr, Pnr

En, Med, Pyr, Su\_H\_CSL, Ths, Wg, Da, Htl, Pan, Twi, Zfh-1, Brk, D-six4, Eya, Hbr, Pnr, Poxm

### Genotype: Pnr\_1; Slp\_1

Delta, Dpp, Hh, Med, Pyr, Shn, Su\_H\_CSL, Ths, Bap, Ci, Htl, Mad, Nicd, Slp, Tin, Mef2, Pnr

Delta, Dpp, Hh, Med, Pyr, Shn, Su\_H\_CSL, Ths, Bap, Ci, Htl, Mad, Nicd, Slp, Tin, Twi, Zfh-1, Eya, Mef2, Pnr

Delta, Dpp, Med, Pyr, Shn, Spi, Su\_H\_CSL, Ths, Upd, Wg, Doc, E\_Spl, Htl, Mad, Nicd, Pan, Slp, Stat92E, Tin(2), Twi, Zfh-1, Eve, Eya, Hbr, Mef2, Pnr

Delta, Hh, Med, Pyr, Su\_H\_CSL, Ths, Ci, Htl, Nicd, Slp, Brk, Pnr, Srp

Delta, Hh, Med, Pyr, Su\_H\_CSL, Ths, Ci, Htl, Nicd, Slp, Twi, Zfh-1, Brk, D-six4, Eya, Pnr, Srp

Med, Pyr, Su\_H\_CSL, Ths, Wg, Da, Htl, Pan, Slp, Twi(2), Zfh-1, Brk, D-six4, Eya, Hbr, Mef2, Pnr, Poxm

### Genotype: Pan\_0; Tin\_2

Delta, Dpp, En, Hh, Med, Pyr, Shn, Su\_H\_CSL, Ths, Bap(3), Bin, Ci, E\_Spl, Htl, Mad, Nicd, Stat92E, Tin(2), Eya, Mef2

Delta, Dpp, Med, Pyr, Shn, Spi, Su\_H\_CSL, Ths, Upd, Wg, Bap, E\_Spl, Htl, Mad, Nicd, Stat92E, Tin(2), Eya, Mef2

Delta, En, Hh, Med, Pyr, Su\_H\_CSL, Ths, Bap(2), Bin, Ci, E\_Spl, Htl, Nicd, Stat92E, Tin(2), Brk, D-six4, Eya, Mef2, Srp(2)

Med, Pyr, Su\_H\_CSL, Ths, Wg, Da, Htl, Stat92E, Tin(2), Brk, D-six4, Eya, Mef2

Med, Pyr, Su\_H\_CSL, Ths, Wg, Da, Htl, Stat92E, Tin(2), Twi, Zfh-1, Brk, D-six4, Eya, Mef2

### Genotype: Pan\_0; Tin\_0

Delta, Dpp, En, Hh, Med, Pyr, Shn, Su\_H\_CSL, Ths, Ci, Htl, Mad, Nicd

Delta, Dpp, Med, Pyr, Shn, Spi, Su\_H\_CSL, Ths, Upd, Wg, E\_Spl, Htl, Mad, Nicd, Stat92E

Delta, En, Hh, Med, Pyr, Su\_H\_CSL, Ths, Ci, Htl, Nicd, Brk, Srp(2)

Med, Pyr, Su\_H\_CSL, Ths, Wg, Da, Htl, Brk

Med, Pyr, Su\_H\_CSL, Ths, Wg, Da, Htl, Twi, Zfh-1, Brk, D-six4, Eya

### Genotype: Bin\_0; Tin\_0

Delta, Dpp, En, Hh, Med, Pyr, Shn, Su\_H\_CSL, Ths, Ci, Htl, Mad, Nicd

Delta, Dpp, Med, Pyr, Shn, Spi, Su\_H\_CSL, Ths, Upd, Wg, Doc, E\_Spl, Htl, Mad, Nicd, Pan, Slp, Stat92E, Twi, Zfh-1, Eya, Hbr

Delta, En, Hh, Med, Pyr, Su\_H\_CSL, Ths, Ci, Htl, Nicd, Brk, Srp(2)

Med, Pyr, Su\_H\_CSL, Ths, Wg, Da, Htl, Pan, Slp, Twi(2), Zfh-1, Brk, D-six4, Eya, Hbr, Mef2, Poxm

### Genotype: Bin\_0; Tin\_2

Delta, Dpp, En, Hh, Med, Pyr, Shn, Su\_H\_CSL, Ths, Bap(3), Ci, E\_Spl, Htl, Mad, Nicd, Stat92E, Tin(2), Eya, Mef2

Delta, Dpp, Med, Pyr, Shn, Spi, Su\_H\_CSL, Ths, Upd, Wg, Doc, E\_Spl, Htl, Mad, Nicd, Pan, Slp, Stat92E, Tin(2), Twi, Zfh-1, Eve, Eya, Hbr, Mef2, Pnr

Delta, En, Hh, Med, Pyr, Su\_H\_CSL, Ths, Bap(2), Ci, E\_Spl, Htl, Nicd, Stat92E, Tin(2), Brk, D-six4, Eya, Mef2, Srp(2)

Med, Pyr, Su\_H\_CSL, Ths, Wg, Da, Htl, Pan, Slp, Stat92E, Tin(2), Twi(2), Zfh-1, Brk, D-six4, Eya, Hbr, Mef2, Poxm

### Genotype: Bin\_2; Med\_1

Delta, Dpp, En, Hh, Med, Pyr, Shn, Su\_H\_CSL, Ths, Bap(3), Bin(2), Ci, Htl, Mad, Nicd, Tin, Mef2

Delta, Dpp, Med, Pyr, Shn, Spi, Su\_H\_CSL, Ths, Upd, Wg, Bap(3), Bin(2), Doc, E\_Spl, Htl, Mad, Nicd, Pan, Slp, Stat92E, Tin(2), Twi, Zfh-1, Eve, Eya, Hbr, Mef2, Pnr

Delta, En, Hh, Med, Pyr, Su\_H\_CSL, Ths, Bap(3), Bin(2), Ci, Htl, Nicd, Brk, Srp(2)

Med, Pyr, Su\_H\_CSL, Ths, Wg, Bap(3), Bin(2), Da, Htl, Pan, Slp, Twi(2), Zfh-1, Brk, D-six4, Eya, Hbr, Mef2, Poxm

### Genotype: Bin\_2; Med\_0

Delta, Dpp, En, Hh, Pyr, Shn, Su\_H\_CSL, Ths, Bap(3), Bin(2), Ci, Htl, Mad, Nicd, Tin, Brk, D-six4, Mef2, Srp(2)

Delta, Dpp, Pyr, Shn, Spi, Su\_H\_CSL, Ths, Upd, Wg, Bap(3), Bin(2), E\_Spl, Htl, Mad, Nicd, Pan, Slp, Stat92E, Tin, Twi, Zfh-1, Brk, D-six4, Eya, Hbr, Mef2, Poxm

Delta, En, Hh, Pyr, Su\_H\_CSL, Ths, Bap(3), Bin(2), Ci, Htl, Nicd, Brk, Srp(2)

Pyr, Su\_H\_CSL, Ths, Wg, Bap(3), Bin(2), Da, Htl, Pan, Slp, Twi(2), Zfh-1, Brk, D-six4, Eya, Hbr, Mef2, Poxm

### Genotype: Doc\_0; Mef2\_1

Delta, Dpp, En, Hh, Med, Pyr, Shn, Su\_H\_CSL, Ths, Bap(3), Bin, Ci, Htl, Mad, Nicd, Tin, Mef2

Delta, Dpp, Med, Pyr, Shn, Spi, Su\_H\_CSL, Ths, Upd, Wg, E\_Spl, Htl, Mad, Nicd, Pan, Slp, Stat92E, Tin(2), Twi, Zfh-1, Eve, Eya, Hbr, Mef2

Delta, En, Hh, Med, Pyr, Su\_H\_CSL, Ths, Ci, Htl, Nicd, Brk, Mef2, Srp(2)

Med, Pyr, Su\_H\_CSL, Ths, Wg, Da, Htl, Pan, Slp, Twi(2), Zfh-1, Brk, D-six4, Eya, Hbr, Mef2, Poxm

### Genotype: Doc\_0; Mef2\_0

Delta, Dpp, En, Hh, Med, Pyr, Shn, Su\_H\_CSL, Ths, Bap(3), Bin, Ci, Htl, Mad, Nicd, Tin

Delta, Dpp, Med, Pyr, Shn, Spi, Su\_H\_CSL, Ths, Upd, Wg, E\_Spl, Htl, Mad, Nicd, Pan, Slp, Stat92E, Tin(2), Twi, Zfh-1, Eve, Eya, Hbr

Delta, En, Hh, Med, Pyr, Su\_H\_CSL, Ths, Ci, Htl, Nicd, Brk, Srp(2)

Med, Pyr, Su\_H\_CSL, Ths, Wg, Da, Htl, Pan, Slp, Twi(2), Zfh-1, Brk, D-six4, Eya, Hbr, Poxm

### Genotype: Bin\_0; Mef2\_0

Delta, Dpp, En, Hh, Med, Pyr, Shn, Su\_H\_CSL, Ths, Bap(3), Ci, Htl, Mad, Nicd, Tin

Delta, Dpp, Med, Pyr, Shn, Spi, Su\_H\_CSL, Ths, Upd, Wg, Doc, E\_Spl, Htl, Mad, Nicd, Pan, Slp, Stat92E, Tin(2), Twi, Zfh-1, Eve, Eya, Hbr, Pnr

Delta, En, Hh, Med, Pyr, Su\_H\_CSL, Ths, Ci, Htl, Nicd, Brk, Srp(2)

Med, Pyr, Su\_H\_CSL, Ths, Wg, Da, Htl, Pan, Slp, Twi(2), Zfh-1, Brk, D-six4, Eya, Hbr, Poxm

### Genotype: Bin\_0; Mef2\_1

Delta, Dpp, En, Hh, Med, Pyr, Shn, Su\_H\_CSL, Ths, Bap(3), Ci, Htl, Mad, Nicd, Tin, Mef2

Delta, Dpp, Med, Pyr, Shn, Spi, Su\_H\_CSL, Ths, Upd, Wg, Doc, E\_Spl, Htl, Mad, Nicd, Pan, Slp, Stat92E, Tin(2), Twi, Zfh-1, Eve, Eya, Hbr, Mef2, Pnr

Delta, En, Hh, Med, Pyr, Su\_H\_CSL, Ths, Ci, Htl, Nicd, Brk, Mef2, Srp(2)

Med, Pyr, Su\_H\_CSL, Ths, Wg, Da, Htl, Pan, Slp, Twi(2), Zfh-1, Brk, D-six4, Eya, Hbr, Mef2, Poxm

### Genotype: Doc\_1

Delta, Dpp, En, Hh, Med, Pyr, Shn, Su\_H\_CSL, Ths, Bap(3), Bin, Ci, Doc, Htl, Mad, Nicd, Tin, Mef2, Pnr

Delta, Dpp, Med, Pyr, Shn, Spi, Su\_H\_CSL, Ths, Upd, Wg, Doc, E\_Spl, Htl, Mad, Nicd, Pan, Slp, Stat92E, Tin(2), Twi, Zfh-1, Eve, Eya, Hbr, Mef2, Pnr

Delta, En, Hh, Med, Pyr, Su\_H\_CSL, Ths, Ci, Doc, Htl, Nicd, Brk, Srp(2)

Med, Pyr, Su\_H\_CSL, Ths, Wg, Da, Doc, Htl, Pan, Slp, Twi(2), Zfh-1, Brk, D-six4, Eya, Hbr, Mef2, Poxm

### Genotype: Pnr\_0; Tin\_2

Delta, Dpp, En, Hh, Med, Pyr, Shn, Su\_H\_CSL, Ths, Bap(3), Bin, Ci, E\_Spl, Htl, Mad, Nicd, Stat92E, Tin(2), Eya, Mef2

Delta, Dpp, Med, Pyr, Shn, Spi, Su\_H\_CSL, Ths, Upd, Wg, Doc, E\_Spl, Htl, Mad, Nicd, Pan, Slp, Stat92E, Tin(2), Twi, Zfh-1, Eve, Eya, Hbr, Mef2

Delta, En, Hh, Med, Pyr, Su\_H\_CSL, Ths, Bap(2), Bin, Ci, E\_Spl, Htl, Nicd, Stat92E, Tin(2), Brk, D-six4, Eya, Mef2, Srp(2)

Med, Pyr, Su\_H\_CSL, Ths, Wg, Da, Htl, Pan, Slp, Stat92E, Tin(2), Twi(2), Zfh-1, Brk, D-six4, Eya, Hbr, Mef2, Poxm

### Genotype: Pnr\_0; Tin\_0

Delta, Dpp, En, Hh, Med, Pyr, Shn, Su\_H\_CSL, Ths, Ci, Htl, Mad, Nicd

Delta, Dpp, Med, Pyr, Shn, Spi, Su\_H\_CSL, Ths, Upd, Wg, Doc, E\_Spl, Htl, Mad, Nicd, Pan, Slp, Stat92E, Twi, Zfh-1, Eya, Hbr

Delta, En, Hh, Med, Pyr, Su\_H\_CSL, Ths, Ci, Htl, Nicd, Brk, Srp(2)

Med, Pyr, Su\_H\_CSL, Ths, Wg, Da, Htl, Pan, Slp, Twi(2), Zfh-1, Brk, D-six4, Eya, Hbr, Mef2, Poxm

### Genotype: Slp\_0; Twi\_0

Delta, Dpp, En, Hh, Med, Pyr, Shn, Su\_H\_CSL, Ths, Bap(3), Bin, Ci, Htl, Mad, Nicd, Tin, Mef2

Delta, Dpp, En, Med, Pyr, Shn, Spi, Su\_H\_CSL, Ths, Upd, Wg, Bap, Doc, E\_Spl, Htl, Mad, Nicd, Pan, Stat92E, Tin(2), Eya, Hbr, Mef2, Pnr

Delta, En, Hh, Med, Pyr, Su\_H\_CSL, Ths, Ci, Htl, Nicd, Brk, Srp(2)

En, Med, Pyr, Su\_H\_CSL, Ths, Wg, Da, Htl, Pan, Brk, Hbr

### Genotype: Slp\_0; Twi\_2

Delta, Dpp, En, Hh, Med, Pyr, Shn, Su\_H\_CSL, Ths, Bap(3), Bin, Ci, Htl, Mad, Nicd, Tin, Twi(2), Zfh-1, Eya, Mef2

Delta, Dpp, En, Med, Pyr, Shn, Spi, Su\_H\_CSL, Ths, Upd, Wg, Bap, Doc, E\_Spl, Htl, Mad, Nicd, Pan, Stat92E, Tin(2), Twi(2), Zfh-1, Eve, Eya, Hbr, Mef2, Pnr

Delta, En, Hh, Med, Pyr, Su\_H\_CSL, Ths, Ci, Htl, Nicd, Twi(2), Zfh-1, Brk, D-six4, Eya, Mef2, Srp(2)

En, Med, Pyr, Su\_H\_CSL, Ths, Wg, Da, Htl, Pan, Twi(2), Zfh-1, Brk, D-six4, Eya, Hbr, Mef2, Poxm

### Genotype: Mad\_0; Slp\_1

Delta, Dpp, Hh, Med, Pyr, Shn, Su\_H\_CSL, Ths, Ci, Htl, Nicd, Slp, Brk, Srp

Delta, Dpp, Hh, Med, Pyr, Shn, Su\_H\_CSL, Ths, Ci, Htl, Nicd, Slp, Twi, Zfh-1, Brk, D-six4, Eya, Srp

Delta, Dpp, Med, Pyr, Shn, Spi, Su\_H\_CSL, Ths, Upd, Wg, E\_Spl, Htl, Nicd, Pan, Slp, Stat92E, Twi, Zfh-1, Brk, D-six4, Eya, Hbr, Poxm

Delta, Hh, Med, Pyr, Su\_H\_CSL, Ths, Ci, Htl, Nicd, Slp, Brk, Srp

Delta, Hh, Med, Pyr, Su\_H\_CSL, Ths, Ci, Htl, Nicd, Slp, Twi, Zfh-1, Brk, D-six4, Eya, Srp

Med, Pyr, Su\_H\_CSL, Ths, Wg, Da, Htl, Pan, Slp, Twi(2), Zfh-1, Brk, D-six4, Eya, Hbr, Mef2, Poxm

### Genotype: Mad\_0; Slp\_0

Delta, Dpp, En, Hh, Med, Pyr, Shn, Su\_H\_CSL, Ths, Ci, Htl, Nicd, Brk, Srp(2)

Delta, Dpp, En, Med, Pyr, Shn, Spi, Su\_H\_CSL, Ths, Upd, Wg, E\_Spl, Htl, Nicd, Pan, Stat92E, Brk, Hbr

Delta, En, Hh, Med, Pyr, Su\_H\_CSL, Ths, Ci, Htl, Nicd, Brk, Srp(2)

En, Med, Pyr, Su\_H\_CSL, Ths, Wg, Da, Htl, Pan, Brk, Hbr

En, Med, Pyr, Su\_H\_CSL, Ths, Wg, Da, Htl, Pan, Twi, Zfh-1, Brk, D-six4, Eya, Hbr, Poxm

### Genotype: Bap\_3; Pan\_1

Delta, Dpp, En, Hh, Med, Pyr, Shn, Su\_H\_CSL, Ths, Bap(3), Bin, Ci, Doc, Htl, Mad, Nicd, Pan, Slp, Tin, Hbr, Mef2, Pnr

Delta, Dpp, En, Hh, Med, Pyr, Shn, Su\_H\_CSL, Ths, Bap(3), Bin, Ci, Doc, Htl, Mad, Nicd, Pan, Slp, Tin, Twi, Zfh-1, Eya, Hbr, Mef2, Pnr

Delta, Dpp, Med, Pyr, Shn, Spi, Su\_H\_CSL, Ths, Upd, Wg, Bap(3), Bin, Doc, E\_Spl, Htl, Mad, Nicd, Pan, Slp, Stat92E, Tin(2), Twi, Zfh-1, Eve, Eya, Hbr, Mef2, Pnr

Delta, En, Hh, Med, Pyr, Su\_H\_CSL, Ths, Bap(3), Bin, Ci, Htl, Nicd, Pan, Slp, Brk, Hbr, Srp(2)

Delta, En, Hh, Med, Pyr, Su\_H\_CSL, Ths, Bap(3), Bin, Ci, Htl, Nicd, Pan, Slp, Twi, Zfh-1, Brk, D-six4, Eya, Hbr, Poxm, Srp(2)

Med, Pyr, Su\_H\_CSL, Ths, Wg, Bap(3), Bin, Da, Htl, Pan, Slp, Twi(2), Zfh-1, Brk, D-six4, Eya, Hbr, Mef2, Poxm

### Genotype: Bap\_3; Pan\_0

Delta, Dpp, En, Hh, Med, Pyr, Shn, Su\_H\_CSL, Ths, Bap(3), Bin, Ci, Htl, Mad, Nicd, Tin, Mef2

Delta, Dpp, Med, Pyr, Shn, Spi, Su\_H\_CSL, Ths, Upd, Wg, Bap(3), Bin, E\_Spl, Htl, Mad, Nicd, Stat92E, Tin, Mef2

Delta, En, Hh, Med, Pyr, Su\_H\_CSL, Ths, Bap(3), Bin, Ci, Htl, Nicd, Brk, Srp(2)

Med, Pyr, Su\_H\_CSL, Ths, Wg, Bap(3), Bin, Da, Htl, Brk

Med, Pyr, Su\_H\_CSL, Ths, Wg, Bap(3), Bin, Da, Htl, Twi, Zfh-1, Brk, D-six4, Eya

### Genotype: Pan\_1; Slp\_1

Delta, Dpp, Hh, Med, Pyr, Shn, Su\_H\_CSL, Ths, Bap, Ci, Doc, Htl, Mad, Nicd, Pan, Slp, Tin, Hbr, Mef2, Pnr

Delta, Dpp, Hh, Med, Pyr, Shn, Su\_H\_CSL, Ths, Bap, Ci, Doc, Htl, Mad, Nicd, Pan, Slp, Tin, Twi, Zfh-1, Eya, Hbr, Mef2, Pnr

Delta, Dpp, Med, Pyr, Shn, Spi, Su\_H\_CSL, Ths, Upd, Wg, Doc, E\_Spl, Htl, Mad, Nicd, Pan, Slp, Stat92E, Tin(2), Twi, Zfh-1, Eve, Eya, Hbr, Mef2, Pnr

Delta, Hh, Med, Pyr, Su\_H\_CSL, Ths, Ci, Htl, Nicd, Pan, Slp, Brk, Hbr, Srp

Delta, Hh, Med, Pyr, Su\_H\_CSL, Ths, Ci, Htl, Nicd, Pan, Slp, Twi, Zfh-1, Brk, D-six4, Eya, Hbr, Poxm, Srp

Med, Pyr, Su\_H\_CSL, Ths, Wg, Da, Htl, Pan, Slp, Twi(2), Zfh-1, Brk, D-six4, Eya, Hbr, Mef2, Poxm

### Genotype: Pan\_1; Slp\_0

Delta, Dpp, En, Hh, Med, Pyr, Shn, Su\_H\_CSL, Ths, Bap(3), Bin, Ci, Doc, Htl, Mad, Nicd, Pan, Tin, Hbr, Mef2, Pnr

Delta, Dpp, En, Med, Pyr, Shn, Spi, Su\_H\_CSL, Ths, Upd, Wg, Bap, Doc, E\_Spl, Htl, Mad, Nicd, Pan, Stat92E, Tin(2), Eya, Hbr, Mef2, Pnr

Delta, En, Hh, Med, Pyr, Su\_H\_CSL, Ths, Ci, Htl, Nicd, Pan, Brk, Hbr, Srp(2)

En, Med, Pyr, Su\_H\_CSL, Ths, Wg, Da, Htl, Pan, Brk, Hbr

En, Med, Pyr, Su\_H\_CSL, Ths, Wg, Da, Htl, Pan, Twi, Zfh-1, Brk, D-six4, Eya, Hbr, Poxm

### Genotype: Mad\_0

Delta, Dpp, En, Hh, Med, Pyr, Shn, Su\_H\_CSL, Ths, Ci, Htl, Nicd, Brk, Srp(2)

Delta, Dpp, Med, Pyr, Shn, Spi, Su\_H\_CSL, Ths, Upd, Wg, E\_Spl, Htl, Nicd, Pan, Slp, Stat92E, Twi, Zfh-1, Brk, D-six4, Eya, Hbr, Poxm

Delta, En, Hh, Med, Pyr, Su\_H\_CSL, Ths, Ci, Htl, Nicd, Brk, Srp(2)

Med, Pyr, Su\_H\_CSL, Ths, Wg, Da, Htl, Pan, Slp, Twi(2), Zfh-1, Brk, D-six4, Eya, Hbr, Mef2, Poxm

### Genotype: Srp\_0; Twi\_2

Delta, Dpp, En, Hh, Med, Pyr, Shn, Su\_H\_CSL, Ths, Bap(3), Bin, Ci, Htl, Mad, Nicd, Tin, Twi(2), Zfh-1, Eya, Mef2

Delta, Dpp, Med, Pyr, Shn, Spi, Su\_H\_CSL, Ths, Upd, Wg, Doc, E\_Spl, Htl, Mad, Nicd, Pan, Slp, Stat92E, Tin(2), Twi(2), Zfh-1, Eve, Eya, Hbr, Mef2, Pnr

Delta, En, Hh, Med, Pyr, Su\_H\_CSL, Ths, Ci, Htl, Nicd, Twi(2), Zfh-1, Brk, D-six4, Eya, Mef2

Med, Pyr, Su\_H\_CSL, Ths, Wg, Da, Htl, Pan, Slp, Twi(2), Zfh-1, Brk, D-six4, Eya, Hbr, Mef2, Poxm

### Genotype: Srp\_0; Twi\_0

Delta, Dpp, En, Hh, Med, Pyr, Shn, Su\_H\_CSL, Ths, Bap(3), Bin, Ci, Htl, Mad, Nicd, Tin, Mef2

Delta, Dpp, Med, Pyr, Shn, Spi, Su\_H\_CSL, Ths, Upd, Wg, Doc, E\_Spl, Htl, Mad, Nicd, Pan, Slp, Stat92E, Tin(2), Eya, Hbr, Mef2, Pnr

Delta, En, Hh, Med, Pyr, Su\_H\_CSL, Ths, Ci, Htl, Nicd, Brk

Med, Pyr, Su\_H\_CSL, Ths, Wg, Da, Htl, Pan, Slp, Brk, Hbr

### Genotype: Bin\_2; Ci\_0

Delta, Dpp, En, Hh, Med, Pyr, Shn, Su\_H\_CSL, Ths, Bap(3), Bin(2), Htl, Mad, Nicd, Tin, Mef2

Delta, Dpp, Med, Pyr, Shn, Spi, Su\_H\_CSL, Ths, Upd, Wg, Bap(3), Bin(2), Doc, E\_Spl, Htl, Mad, Nicd, Pan, Slp, Stat92E, Tin(2), Twi, Zfh-1, Eve, Eya, Hbr, Mef2, Pnr

Delta, En, Hh, Med, Pyr, Su\_H\_CSL, Ths, Bap(3), Bin(2), Htl, Nicd, Brk

Med, Pyr, Su\_H\_CSL, Ths, Wg, Bap(3), Bin(2), Da, Htl, Pan, Slp, Twi(2), Zfh-1, Brk, D-six4, Eya, Hbr, Mef2, Poxm

### Genotype: Bin\_2; Ci\_1

Delta, Dpp, En, Hh, Med, Pyr, Shn, Su\_H\_CSL, Ths, Bap(3), Bin(2), Ci, Htl, Mad, Nicd, Tin, Mef2

Delta, Dpp, Med, Pyr, Shn, Spi, Su\_H\_CSL, Ths, Upd, Wg, Bap(3), Bin(2), Ci, Doc, E\_Spl, Htl, Mad, Nicd, Pan, Slp, Stat92E, Tin(2), Twi, Zfh-1, Eve, Eya, Hbr, Mef2, Pnr

Delta, En, Hh, Med, Pyr, Su\_H\_CSL, Ths, Bap(3), Bin(2), Ci, Htl, Nicd, Brk, Srp(2)

Med, Pyr, Su\_H\_CSL, Ths, Wg, Bap(3), Bin(2), Ci, Da, Htl, Pan, Slp, Twi(2), Zfh-1, Brk, D-six4, Eya, Hbr, Mef2, Poxm, Srp

### Genotype: Slp\_1; Srp\_0

Delta, Dpp, Hh, Med, Pyr, Shn, Su\_H\_CSL, Ths, Bap, Ci, Htl, Mad, Nicd, Slp, Tin, Mef2

Delta, Dpp, Hh, Med, Pyr, Shn, Su\_H\_CSL, Ths, Bap, Ci, Htl, Mad, Nicd, Slp, Tin, Twi, Zfh-1, Eya, Mef2

Delta, Dpp, Med, Pyr, Shn, Spi, Su\_H\_CSL, Ths, Upd, Wg, Doc, E\_Spl, Htl, Mad, Nicd, Pan, Slp, Stat92E, Tin(2), Twi, Zfh-1, Eve, Eya, Hbr, Mef2, Pnr

Delta, Hh, Med, Pyr, Su\_H\_CSL, Ths, Ci, Htl, Nicd, Slp, Brk

Delta, Hh, Med, Pyr, Su\_H\_CSL, Ths, Ci, Htl, Nicd, Slp, Twi, Zfh-1, Brk, D-six4, Eya

Med, Pyr, Su\_H\_CSL, Ths, Wg, Da, Htl, Pan, Slp, Twi(2), Zfh-1, Brk, D-six4, Eya, Hbr, Mef2, Poxm

### Genotype: Slp\_1; Srp\_2

Delta, Dpp, Hh, Med, Pyr, Shn, Su\_H\_CSL, Ths, Bap, Ci, Htl, Mad, Nicd, Slp, Tin, Mef2, Srp(2)

Delta, Dpp, Hh, Med, Pyr, Shn, Su\_H\_CSL, Ths, Bap, Ci, Htl, Mad, Nicd, Slp, Tin, Twi, Zfh-1, Eya, Mef2, Srp(2)

Delta, Dpp, Med, Pyr, Shn, Spi, Su\_H\_CSL, Ths, Upd, Wg, Doc, E\_Spl, Htl, Mad, Nicd, Pan, Slp, Stat92E, Tin(2), Twi, Zfh-1, Eve, Eya, Hbr, Mef2, Pnr, Srp(2)

Delta, Hh, Med, Pyr, Su\_H\_CSL, Ths, Ci, Htl, Nicd, Slp, Brk, Srp(2)

Delta, Hh, Med, Pyr, Su\_H\_CSL, Ths, Ci, Htl, Nicd, Slp, Twi, Zfh-1, Brk, D-six4, Eya, Srp(2)

Med, Pyr, Su\_H\_CSL, Ths, Wg, Da, Htl, Pan, Slp, Twi(2), Zfh-1, Brk, D-six4, Eya, Hbr, Mef2, Poxm, Srp(2)

### Genotype: Pan\_1; Tin\_2

Delta, Dpp, En, Hh, Med, Pyr, Shn, Su\_H\_CSL, Ths, Bap, Ci, Doc, E\_Spl, Htl, Mad, Nicd, Pan, Slp, Stat92E, Tin(2), Eya, Hbr, Mef2, Pnr

Delta, Dpp, En, Hh, Med, Pyr, Shn, Su\_H\_CSL, Ths, Bap, Ci, Doc, E\_Spl, Htl, Mad, Nicd, Pan, Slp, Stat92E, Tin(2), Twi, Zfh-1, Eya, Hbr, Mef2, Pnr

Delta, Dpp, Med, Pyr, Shn, Spi, Su\_H\_CSL, Ths, Upd, Wg, Doc, E\_Spl, Htl, Mad, Nicd, Pan, Slp, Stat92E, Tin(2), Twi, Zfh-1, Eve, Eya, Hbr, Mef2, Pnr

Delta, En, Hh, Med, Pyr, Su\_H\_CSL, Ths, Bap, Ci, E\_Spl, Htl, Nicd, Pan, Slp, Stat92E, Tin(2), Brk, D-six4, Eya, Hbr, Mef2, Srp(2)

Delta, En, Hh, Med, Pyr, Su\_H\_CSL, Ths, Bap, Ci, E\_Spl, Htl, Nicd, Pan, Slp, Stat92E, Tin(2), Twi, Zfh-1, Brk, D-six4, Eya, Hbr, Mef2, Poxm, Srp(2)

Med, Pyr, Su\_H\_CSL, Ths, Wg, Da, Htl, Pan, Slp, Stat92E, Tin(2), Twi(2), Zfh-1, Brk, D-six4, Eya, Hbr, Mef2, Poxm

### Genotype: Pan\_1; Tin\_0

Delta, Dpp, En, Hh, Med, Pyr, Shn, Su\_H\_CSL, Ths, Ci, Doc, Htl, Mad, Nicd, Pan, Slp, Hbr

Delta, Dpp, En, Hh, Med, Pyr, Shn, Su\_H\_CSL, Ths, Ci, Doc, Htl, Mad, Nicd, Pan, Slp, Twi, Zfh-1, Eya, Hbr

Delta, Dpp, Med, Pyr, Shn, Spi, Su\_H\_CSL, Ths, Upd, Wg, Doc, E\_Spl, Htl, Mad, Nicd, Pan, Slp, Stat92E, Twi, Zfh-1, Eya, Hbr

Delta, En, Hh, Med, Pyr, Su\_H\_CSL, Ths, Ci, Htl, Nicd, Pan, Slp, Brk, Hbr, Srp(2)

Delta, En, Hh, Med, Pyr, Su\_H\_CSL, Ths, Ci, Htl, Nicd, Pan, Slp, Twi, Zfh-1, Brk, D-six4, Eya, Hbr, Poxm, Srp(2)

Med, Pyr, Su\_H\_CSL, Ths, Wg, Da, Htl, Pan, Slp, Twi(2), Zfh-1, Brk, D-six4, Eya, Hbr, Mef2, Poxm

### Genotype: Doc\_0; Mad\_0

Delta, Dpp, En, Hh, Med, Pyr, Shn, Su\_H\_CSL, Ths, Ci, Htl, Nicd, Brk, Srp(2)

Delta, Dpp, Med, Pyr, Shn, Spi, Su\_H\_CSL, Ths, Upd, Wg, E\_Spl, Htl, Nicd, Pan, Slp, Stat92E, Twi, Zfh-1, Brk, D-six4, Eya, Hbr, Poxm

Delta, En, Hh, Med, Pyr, Su\_H\_CSL, Ths, Ci, Htl, Nicd, Brk, Srp(2)

Med, Pyr, Su\_H\_CSL, Ths, Wg, Da, Htl, Pan, Slp, Twi(2), Zfh-1, Brk, D-six4, Eya, Hbr, Mef2, Poxm

### Genotype: Doc\_0; Mad\_1

Delta, Dpp, En, Hh, Med, Pyr, Shn, Su\_H\_CSL, Ths, Bap(3), Bin, Ci, Htl, Mad, Nicd, Tin, Mef2

Delta, Dpp, Med, Pyr, Shn, Spi, Su\_H\_CSL, Ths, Upd, Wg, E\_Spl, Htl, Mad, Nicd, Pan, Slp, Stat92E, Tin(2), Twi, Zfh-1, Eve, Eya, Hbr, Mef2

Delta, En, Hh, Med, Pyr, Su\_H\_CSL, Ths, Bap(3), Bin, Ci, Htl, Mad, Nicd, Tin, Brk, Mef2

Med, Pyr, Su\_H\_CSL, Ths, Wg, Da, Htl, Mad, Pan, Slp, Tin, Twi(2), Zfh-1, Brk, Eya, Hbr, Mef2

### Genotype: Mad\_1; Slp\_0

Delta, Dpp, En, Hh, Med, Pyr, Shn, Su\_H\_CSL, Ths, Bap(3), Bin, Ci, Htl, Mad, Nicd, Tin, Mef2

Delta, Dpp, En, Med, Pyr, Shn, Spi, Su\_H\_CSL, Ths, Upd, Wg, Bap, Doc, E\_Spl, Htl, Mad, Nicd, Pan, Stat92E, Tin(2), Eya, Hbr, Mef2, Pnr

Delta, En, Hh, Med, Pyr, Su\_H\_CSL, Ths, Bap(3), Bin, Ci, Htl, Mad, Nicd, Tin, Brk, Mef2

En, Med, Pyr, Su\_H\_CSL, Ths, Wg, Bap, Da, Doc, Htl, Mad, Pan, Tin, Brk, Hbr, Mef2, Pnr

En, Med, Pyr, Su\_H\_CSL, Ths, Wg, Bap, Da, Doc, Htl, Mad, Pan, Tin, Twi, Zfh-1, Brk, Eya, Hbr, Mef2, Pnr

### Genotype: Mad\_1; Slp\_1

Delta, Dpp, Hh, Med, Pyr, Shn, Su\_H\_CSL, Ths, Bap, Ci, Htl, Mad, Nicd, Slp, Tin, Mef2

Delta, Dpp, Hh, Med, Pyr, Shn, Su\_H\_CSL, Ths, Bap, Ci, Htl, Mad, Nicd, Slp, Tin, Twi, Zfh-1, Eya, Mef2

Delta, Dpp, Med, Pyr, Shn, Spi, Su\_H\_CSL, Ths, Upd, Wg, Doc, E\_Spl, Htl, Mad, Nicd, Pan, Slp, Stat92E, Tin(2), Twi, Zfh-1, Eve, Eya, Hbr, Mef2, Pnr

Delta, Hh, Med, Pyr, Su\_H\_CSL, Ths, Bap, Ci, Htl, Mad, Nicd, Slp, Tin, Brk, Mef2

Delta, Hh, Med, Pyr, Su\_H\_CSL, Ths, Bap, Ci, Htl, Mad, Nicd, Slp, Tin, Twi, Zfh-1, Brk, Eya, Mef2

Med, Pyr, Su\_H\_CSL, Ths, Wg, Da, Doc, Htl, Mad, Pan, Slp, Tin, Twi(2), Zfh-1, Brk, Eya, Hbr, Mef2, Pnr

### Genotype: Doc\_0; Nicd\_1

Delta, Dpp, En, Hh, Med, Pyr, Shn, Su\_H\_CSL, Ths, Bap(3), Bin, Ci, Htl, Mad, Nicd, Tin, Mef2

Delta, Dpp, Med, Pyr, Shn, Spi, Su\_H\_CSL, Ths, Upd, Wg, E\_Spl, Htl, Mad, Nicd, Pan, Slp, Stat92E, Tin(2), Twi, Zfh-1, Eve, Eya, Hbr, Mef2

Delta, En, Hh, Med, Pyr, Su\_H\_CSL, Ths, Ci, Htl, Nicd, Brk, Srp(2)

Med, Pyr, Su\_H\_CSL, Ths, Wg, Htl, Nicd, Pan, Slp, Twi, Zfh-1, Brk, D-six4, Eya, Hbr, Poxm

### Genotype: Doc\_0; Nicd\_0

Delta, Dpp, En, Hh, Med, Pyr, Shn, Su\_H\_CSL, Ths, Bap(3), Bin, Ci, Da, Htl, Mad, Tin, Mef2

Delta, Dpp, En, Hh, Med, Pyr, Shn, Su\_H\_CSL, Ths, Bap(3), Bin, Ci, Da, Htl, Mad, Tin, Twi, Zfh-1, Eya, Mef2

Delta, Dpp, Med, Pyr, Shn, Spi, Su\_H\_CSL, Ths, Upd, Wg, Da, Htl, Mad, Pan, Slp, Stat92E, Tin(2), Twi(2), Zfh-1, Eve, Eya, Hbr, Mef2

Delta, En, Hh, Med, Pyr, Su\_H\_CSL, Ths, Ci, Da, Htl, Brk, Srp(2)

Delta, En, Hh, Med, Pyr, Su\_H\_CSL, Ths, Ci, Da, Htl, Twi, Zfh-1, Brk, D-six4, Eya, Srp(2)

Med, Pyr, Su\_H\_CSL, Ths, Wg, Da, Htl, Pan, Slp, Twi(2), Zfh-1, Brk, D-six4, Eya, Hbr, Mef2, Poxm

### Genotype: Srp\_0; Tin\_0

Delta, Dpp, En, Hh, Med, Pyr, Shn, Su\_H\_CSL, Ths, Ci, Htl, Mad, Nicd

Delta, Dpp, Med, Pyr, Shn, Spi, Su\_H\_CSL, Ths, Upd, Wg, Doc, E\_Spl, Htl, Mad, Nicd, Pan, Slp, Stat92E, Twi, Zfh-1, Eya, Hbr

Delta, En, Hh, Med, Pyr, Su\_H\_CSL, Ths, Ci, Htl, Nicd, Brk

Med, Pyr, Su\_H\_CSL, Ths, Wg, Da, Htl, Pan, Slp, Twi(2), Zfh-1, Brk, D-six4, Eya, Hbr, Mef2, Poxm

### Genotype: Nicd\_1; Pnr\_0

Delta, Dpp, En, Hh, Med, Pyr, Shn, Su\_H\_CSL, Ths, Bap(3), Bin, Ci, Htl, Mad, Nicd, Tin, Mef2

Delta, Dpp, Med, Pyr, Shn, Spi, Su\_H\_CSL, Ths, Upd, Wg, Doc, E\_Spl, Htl, Mad, Nicd, Pan, Slp, Stat92E, Tin(2), Twi, Zfh-1, Eve, Eya, Hbr, Mef2

Delta, En, Hh, Med, Pyr, Su\_H\_CSL, Ths, Ci, Htl, Nicd, Brk, Srp(2)

Med, Pyr, Su\_H\_CSL, Ths, Wg, Htl, Nicd, Pan, Slp, Twi, Zfh-1, Brk, D-six4, Eya, Hbr, Poxm

### Genotype: Doc\_1; Nicd\_0

Delta, Dpp, En, Hh, Med, Pyr, Shn, Su\_H\_CSL, Ths, Bap(3), Bin, Ci, Da, Doc, Htl, Mad, Tin, Mef2, Pnr

Delta, Dpp, En, Hh, Med, Pyr, Shn, Su\_H\_CSL, Ths, Bap(3), Bin, Ci, Da, Doc, Htl, Mad, Tin, Twi, Zfh-1, Eya, Mef2, Pnr

Delta, Dpp, Med, Pyr, Shn, Spi, Su\_H\_CSL, Ths, Upd, Wg, Da, Doc, Htl, Mad, Pan, Slp, Stat92E, Tin(2), Twi(2), Zfh-1, Eve, Eya, Hbr, Mef2, Pnr

Delta, En, Hh, Med, Pyr, Su\_H\_CSL, Ths, Ci, Da, Doc, Htl, Brk, Srp(2)

Delta, En, Hh, Med, Pyr, Su\_H\_CSL, Ths, Ci, Da, Doc, Htl, Twi, Zfh-1, Brk, D-six4, Eya, Srp(2)

Med, Pyr, Su\_H\_CSL, Ths, Wg, Da, Doc, Htl, Pan, Slp, Twi(2), Zfh-1, Brk, D-six4, Eya, Hbr, Mef2, Poxm

### Genotype: Doc\_1; Nicd\_1

Delta, Dpp, En, Hh, Med, Pyr, Shn, Su\_H\_CSL, Ths, Bap(3), Bin, Ci, Doc, Htl, Mad, Nicd, Tin, Mef2, Pnr

Delta, Dpp, Med, Pyr, Shn, Spi, Su\_H\_CSL, Ths, Upd, Wg, Doc, E\_Spl, Htl, Mad, Nicd, Pan, Slp, Stat92E, Tin(2), Twi, Zfh-1, Eve, Eya, Hbr, Mef2, Pnr

Delta, En, Hh, Med, Pyr, Su\_H\_CSL, Ths, Ci, Doc, Htl, Nicd, Brk, Srp(2)

Med, Pyr, Su\_H\_CSL, Ths, Wg, Doc, Htl, Nicd, Pan, Slp, Twi, Zfh-1, Brk, D-six4, Eya, Hbr, Poxm

### Genotype: Bin\_2; Pan\_1

Delta, Dpp, En, Hh, Med, Pyr, Shn, Su\_H\_CSL, Ths, Bap(3), Bin(2), Ci, Doc, Htl, Mad, Nicd, Pan, Slp, Tin, Hbr, Mef2, Pnr

Delta, Dpp, En, Hh, Med, Pyr, Shn, Su\_H\_CSL, Ths, Bap(3), Bin(2), Ci, Doc, Htl, Mad, Nicd, Pan, Slp, Tin, Twi, Zfh-1, Eya, Hbr, Mef2, Pnr

Delta, Dpp, Med, Pyr, Shn, Spi, Su\_H\_CSL, Ths, Upd, Wg, Bap(3), Bin(2), Doc, E\_Spl, Htl, Mad, Nicd, Pan, Slp, Stat92E, Tin(2), Twi, Zfh-1, Eve, Eya, Hbr, Mef2, Pnr

Delta, En, Hh, Med, Pyr, Su\_H\_CSL, Ths, Bap(3), Bin(2), Ci, Htl, Nicd, Pan, Slp, Brk, Hbr, Srp(2)

Delta, En, Hh, Med, Pyr, Su\_H\_CSL, Ths, Bap(3), Bin(2), Ci, Htl, Nicd, Pan, Slp, Twi, Zfh-1, Brk, D-six4, Eya, Hbr, Poxm, Srp(2)

Med, Pyr, Su\_H\_CSL, Ths, Wg, Bap(3), Bin(2), Da, Htl, Pan, Slp, Twi(2), Zfh-1, Brk, D-six4, Eya, Hbr, Mef2, Poxm

### Genotype: Doc\_0; Pan\_1

Delta, Dpp, En, Hh, Med, Pyr, Shn, Su\_H\_CSL, Ths, Bap, Ci, Htl, Mad, Nicd, Pan, Slp, Tin, Hbr, Mef2

Delta, Dpp, En, Hh, Med, Pyr, Shn, Su\_H\_CSL, Ths, Bap, Ci, Htl, Mad, Nicd, Pan, Slp, Tin, Twi, Zfh-1, Eya, Hbr, Mef2

Delta, Dpp, Med, Pyr, Shn, Spi, Su\_H\_CSL, Ths, Upd, Wg, E\_Spl, Htl, Mad, Nicd, Pan, Slp, Stat92E, Tin(2), Twi, Zfh-1, Eve, Eya, Hbr, Mef2

Delta, En, Hh, Med, Pyr, Su\_H\_CSL, Ths, Ci, Htl, Nicd, Pan, Slp, Brk, Hbr, Srp(2)

Delta, En, Hh, Med, Pyr, Su\_H\_CSL, Ths, Ci, Htl, Nicd, Pan, Slp, Twi, Zfh-1, Brk, D-six4, Eya, Hbr, Poxm, Srp(2)

Med, Pyr, Su\_H\_CSL, Ths, Wg, Da, Htl, Pan, Slp, Twi(2), Zfh-1, Brk, D-six4, Eya, Hbr, Mef2, Poxm

### Genotype: Doc\_0; Pan\_0

Delta, Dpp, En, Hh, Med, Pyr, Shn, Su\_H\_CSL, Ths, Bap(3), Bin, Ci, Htl, Mad, Nicd, Tin, Mef2

Delta, Dpp, Med, Pyr, Shn, Spi, Su\_H\_CSL, Ths, Upd, Wg, Bap, E\_Spl, Htl, Mad, Nicd, Stat92E, Tin, Mef2

Delta, En, Hh, Med, Pyr, Su\_H\_CSL, Ths, Ci, Htl, Nicd, Brk, Srp(2)

Med, Pyr, Su\_H\_CSL, Ths, Wg, Da, Htl, Brk

Med, Pyr, Su\_H\_CSL, Ths, Wg, Da, Htl, Twi, Zfh-1, Brk, D-six4, Eya

### Genotype: Med\_0; Srp\_0

Delta, Dpp, En, Hh, Pyr, Shn, Su\_H\_CSL, Ths, Bap(2), Bin, Ci, Htl, Mad, Nicd, Tin, Brk, D-six4, Mef2

Delta, Dpp, Pyr, Shn, Spi, Su\_H\_CSL, Ths, Upd, Wg, E\_Spl, Htl, Mad, Nicd, Pan, Slp, Stat92E, Tin, Twi, Zfh-1, Brk, D-six4, Eya, Hbr, Mef2, Poxm

Delta, En, Hh, Pyr, Su\_H\_CSL, Ths, Ci, Htl, Nicd, Brk

Pyr, Su\_H\_CSL, Ths, Wg, Da, Htl, Pan, Slp, Twi(2), Zfh-1, Brk, D-six4, Eya, Hbr, Mef2, Poxm

### Genotype: Pnr\_1

Delta, Dpp, En, Hh, Med, Pyr, Shn, Su\_H\_CSL, Ths, Bap(3), Bin, Ci, Htl, Mad, Nicd, Tin, Mef2, Pnr

Delta, Dpp, Med, Pyr, Shn, Spi, Su\_H\_CSL, Ths, Upd, Wg, Doc, E\_Spl, Htl, Mad, Nicd, Pan, Slp, Stat92E, Tin(2), Twi, Zfh-1, Eve, Eya, Hbr, Mef2, Pnr

Delta, En, Hh, Med, Pyr, Su\_H\_CSL, Ths, Ci, Htl, Nicd, Brk, Pnr, Srp(2)

Med, Pyr, Su\_H\_CSL, Ths, Wg, Da, Htl, Pan, Slp, Twi(2), Zfh-1, Brk, D-six4, Eya, Hbr, Mef2, Pnr, Poxm

### Genotype: Med\_0; Srp\_2

Delta, Dpp, En, Hh, Pyr, Shn, Su\_H\_CSL, Ths, Bap(2), Bin, Ci, Htl, Mad, Nicd, Tin, Brk, D-six4, Mef2, Srp(2)

Delta, Dpp, Pyr, Shn, Spi, Su\_H\_CSL, Ths, Upd, Wg, E\_Spl, Htl, Mad, Nicd, Pan, Slp, Stat92E, Tin, Twi, Zfh-1, Brk, D-six4, Eya, Hbr, Mef2, Poxm, Srp(2)

Delta, En, Hh, Pyr, Su\_H\_CSL, Ths, Ci, Htl, Nicd, Brk, Srp(2)

Pyr, Su\_H\_CSL, Ths, Wg, Da, Htl, Pan, Slp, Twi(2), Zfh-1, Brk, D-six4, Eya, Hbr, Mef2, Poxm, Srp(2)

### Genotype: Doc\_1; Med\_1

Delta, Dpp, En, Hh, Med, Pyr, Shn, Su\_H\_CSL, Ths, Bap(3), Bin, Ci, Doc, Htl, Mad, Nicd, Tin, Mef2, Pnr

Delta, Dpp, Med, Pyr, Shn, Spi, Su\_H\_CSL, Ths, Upd, Wg, Doc, E\_Spl, Htl, Mad, Nicd, Pan, Slp, Stat92E, Tin(2), Twi, Zfh-1, Eve, Eya, Hbr, Mef2, Pnr

Delta, En, Hh, Med, Pyr, Su\_H\_CSL, Ths, Ci, Doc, Htl, Nicd, Brk, Srp(2)

Med, Pyr, Su\_H\_CSL, Ths, Wg, Da, Doc, Htl, Pan, Slp, Twi(2), Zfh-1, Brk, D-six4, Eya, Hbr, Mef2, Poxm

### Genotype: Doc\_1; Med\_0

Delta, Dpp, En, Hh, Pyr, Shn, Su\_H\_CSL, Ths, Bap(2), Bin, Ci, Doc, Htl, Mad, Nicd, Tin, Brk, D-six4, Mef2, Pnr, Srp(2)

Delta, Dpp, Pyr, Shn, Spi, Su\_H\_CSL, Ths, Upd, Wg, Doc, E\_Spl, Htl, Mad, Nicd, Pan, Slp, Stat92E, Tin, Twi, Zfh-1, Brk, D-six4, Eya, Hbr, Mef2, Pnr, Poxm

Delta, En, Hh, Pyr, Su\_H\_CSL, Ths, Ci, Doc, Htl, Nicd, Brk, Srp(2)

Pyr, Su\_H\_CSL, Ths, Wg, Da, Doc, Htl, Pan, Slp, Twi(2), Zfh-1, Brk, D-six4, Eya, Hbr, Mef2, Poxm

### Genotype: Bap\_3; Pnr\_0

Delta, Dpp, En, Hh, Med, Pyr, Shn, Su\_H\_CSL, Ths, Bap(3), Bin, Ci, Htl, Mad, Nicd, Tin, Mef2

Delta, Dpp, Med, Pyr, Shn, Spi, Su\_H\_CSL, Ths, Upd, Wg, Bap(3), Bin, Doc, E\_Spl, Htl, Mad, Nicd, Pan, Slp, Stat92E, Tin(2), Twi, Zfh-1, Eve, Eya, Hbr, Mef2

Delta, En, Hh, Med, Pyr, Su\_H\_CSL, Ths, Bap(3), Bin, Ci, Htl, Nicd, Brk, Srp(2)

Med, Pyr, Su\_H\_CSL, Ths, Wg, Bap(3), Bin, Da, Htl, Pan, Slp, Twi(2), Zfh-1, Brk, D-six4, Eya, Hbr, Mef2, Poxm

### Genotype: Bap\_3; Pnr\_1

Delta, Dpp, En, Hh, Med, Pyr, Shn, Su\_H\_CSL, Ths, Bap(3), Bin, Ci, Htl, Mad, Nicd, Tin, Mef2, Pnr

Delta, Dpp, Med, Pyr, Shn, Spi, Su\_H\_CSL, Ths, Upd, Wg, Bap(3), Bin, Doc, E\_Spl, Htl, Mad, Nicd, Pan, Slp, Stat92E, Tin(2), Twi, Zfh-1, Eve, Eya, Hbr, Mef2, Pnr

Delta, En, Hh, Med, Pyr, Su\_H\_CSL, Ths, Bap(3), Bin, Ci, Htl, Nicd, Brk, Pnr, Srp(2)

Med, Pyr, Su\_H\_CSL, Ths, Wg, Bap(3), Bin, Da, Htl, Pan, Slp, Twi(2), Zfh-1, Brk, D-six4, Eya, Hbr, Mef2, Pnr, Poxm

### Genotype: Nicd\_0; Tin\_0

Delta, Dpp, En, Hh, Med, Pyr, Shn, Su\_H\_CSL, Ths, Ci, Da, Htl, Mad

Delta, Dpp, En, Hh, Med, Pyr, Shn, Su\_H\_CSL, Ths, Ci, Da, Htl, Mad, Twi, Zfh-1, Eya

Delta, Dpp, Med, Pyr, Shn, Spi, Su\_H\_CSL, Ths, Upd, Wg, Da, Doc, Htl, Mad, Pan, Slp, Stat92E, Twi(2), Zfh-1, Eya, Hbr, Mef2

Delta, En, Hh, Med, Pyr, Su\_H\_CSL, Ths, Ci, Da, Htl, Brk, Srp(2)

Delta, En, Hh, Med, Pyr, Su\_H\_CSL, Ths, Ci, Da, Htl, Twi, Zfh-1, Brk, D-six4, Eya, Srp(2)

Med, Pyr, Su\_H\_CSL, Ths, Wg, Da, Htl, Pan, Slp, Twi(2), Zfh-1, Brk, D-six4, Eya, Hbr, Mef2, Poxm

### Genotype: Pan\_0; Pnr\_1

Delta, Dpp, En, Hh, Med, Pyr, Shn, Su\_H\_CSL, Ths, Bap(3), Bin, Ci, Htl, Mad, Nicd, Tin, Mef2, Pnr

Delta, Dpp, Med, Pyr, Shn, Spi, Su\_H\_CSL, Ths, Upd, Wg, Bap, E\_Spl, Htl, Mad, Nicd, Stat92E, Tin, Mef2, Pnr

Delta, En, Hh, Med, Pyr, Su\_H\_CSL, Ths, Ci, Htl, Nicd, Brk, Pnr, Srp(2)

Med, Pyr, Su\_H\_CSL, Ths, Wg, Da, Htl, Brk, Pnr

Med, Pyr, Su\_H\_CSL, Ths, Wg, Da, Htl, Twi, Zfh-1, Brk, D-six4, Eya, Pnr

### Genotype: Pan\_0; Pnr\_0

Delta, Dpp, En, Hh, Med, Pyr, Shn, Su\_H\_CSL, Ths, Bap(3), Bin, Ci, Htl, Mad, Nicd, Tin, Mef2

Delta, Dpp, Med, Pyr, Shn, Spi, Su\_H\_CSL, Ths, Upd, Wg, Bap, E\_Spl, Htl, Mad, Nicd, Stat92E, Tin, Mef2

Delta, En, Hh, Med, Pyr, Su\_H\_CSL, Ths, Ci, Htl, Nicd, Brk, Srp(2)

Med, Pyr, Su\_H\_CSL, Ths, Wg, Da, Htl, Brk

Med, Pyr, Su\_H\_CSL, Ths, Wg, Da, Htl, Twi, Zfh-1, Brk, D-six4, Eya

### Genotype: Ci\_0

Delta, Dpp, En, Hh, Med, Pyr, Shn, Su\_H\_CSL, Ths, Bap, Htl, Mad, Nicd, Tin, Mef2

Delta, Dpp, En, Hh, Med, Pyr, Shn, Su\_H\_CSL, Ths, Bap(2), Bin, Htl, Mad, Nicd, Tin, Mef2

Delta, Dpp, Med, Pyr, Shn, Spi, Su\_H\_CSL, Ths, Upd, Wg, Doc, E\_Spl, Htl, Mad, Nicd, Pan, Slp, Stat92E, Tin(2), Twi, Zfh-1, Eve, Eya, Hbr, Mef2, Pnr

Delta, En, Hh, Med, Pyr, Su\_H\_CSL, Ths, Htl, Nicd, Brk

Med, Pyr, Su\_H\_CSL, Ths, Wg, Da, Htl, Pan, Slp, Twi(2), Zfh-1, Brk, D-six4, Eya, Hbr, Mef2, Poxm

### Genotype: Pnr\_0; Slp\_1

Delta, Dpp, Hh, Med, Pyr, Shn, Su\_H\_CSL, Ths, Bap, Ci, Htl, Mad, Nicd, Slp, Tin, Mef2

Delta, Dpp, Hh, Med, Pyr, Shn, Su\_H\_CSL, Ths, Bap, Ci, Htl, Mad, Nicd, Slp, Tin, Twi, Zfh-1, Eya, Mef2

Delta, Dpp, Med, Pyr, Shn, Spi, Su\_H\_CSL, Ths, Upd, Wg, Doc, E\_Spl, Htl, Mad, Nicd, Pan, Slp, Stat92E, Tin(2), Twi, Zfh-1, Eve, Eya, Hbr, Mef2

Delta, Hh, Med, Pyr, Su\_H\_CSL, Ths, Ci, Htl, Nicd, Slp, Brk, Srp

Delta, Hh, Med, Pyr, Su\_H\_CSL, Ths, Ci, Htl, Nicd, Slp, Twi, Zfh-1, Brk, D-six4, Eya, Srp

Med, Pyr, Su\_H\_CSL, Ths, Wg, Da, Htl, Pan, Slp, Twi(2), Zfh-1, Brk, D-six4, Eya, Hbr, Mef2, Poxm

### Genotype: Pan\_0

Delta, Dpp, En, Hh, Med, Pyr, Shn, Su\_H\_CSL, Ths, Bap(3), Bin, Ci, Htl, Mad, Nicd, Tin, Mef2

Delta, Dpp, Med, Pyr, Shn, Spi, Su\_H\_CSL, Ths, Upd, Wg, Bap, E\_Spl, Htl, Mad, Nicd, Stat92E, Tin, Mef2

Delta, En, Hh, Med, Pyr, Su\_H\_CSL, Ths, Ci, Htl, Nicd, Brk, Srp(2)

Med, Pyr, Su\_H\_CSL, Ths, Wg, Da, Htl, Brk

Med, Pyr, Su\_H\_CSL, Ths, Wg, Da, Htl, Twi, Zfh-1, Brk, D-six4, Eya

### Genotype: Pnr\_0; Slp\_0

Delta, Dpp, En, Hh, Med, Pyr, Shn, Su\_H\_CSL, Ths, Bap(3), Bin, Ci, Htl, Mad, Nicd, Tin, Mef2

Delta, Dpp, En, Med, Pyr, Shn, Spi, Su\_H\_CSL, Ths, Upd, Wg, Bap, Doc, E\_Spl, Htl, Mad, Nicd, Pan, Stat92E, Tin(2), Eya, Hbr, Mef2

Delta, En, Hh, Med, Pyr, Su\_H\_CSL, Ths, Ci, Htl, Nicd, Brk, Srp(2)

En, Med, Pyr, Su\_H\_CSL, Ths, Wg, Da, Htl, Pan, Brk, Hbr

En, Med, Pyr, Su\_H\_CSL, Ths, Wg, Da, Htl, Pan, Twi, Zfh-1, Brk, D-six4, Eya, Hbr, Poxm

### Genotype: Mad\_0; Twi\_0

Delta, Dpp, En, Hh, Med, Pyr, Shn, Su\_H\_CSL, Ths, Ci, Htl, Nicd, Brk, Srp(2)

Delta, Dpp, Med, Pyr, Shn, Spi, Su\_H\_CSL, Ths, Upd, Wg, E\_Spl, Htl, Nicd, Pan, Slp, Stat92E, Brk, Hbr

Delta, En, Hh, Med, Pyr, Su\_H\_CSL, Ths, Ci, Htl, Nicd, Brk, Srp(2)

Med, Pyr, Su\_H\_CSL, Ths, Wg, Da, Htl, Pan, Slp, Brk, Hbr

### Genotype: Mad\_0; Twi\_2

Delta, Dpp, En, Hh, Med, Pyr, Shn, Su\_H\_CSL, Ths, Ci, Htl, Nicd, Twi(2), Zfh-1, Brk, D-six4, Eya, Mef2, Srp(2)

Delta, Dpp, Med, Pyr, Shn, Spi, Su\_H\_CSL, Ths, Upd, Wg, E\_Spl, Htl, Nicd, Pan, Slp, Stat92E, Twi(2), Zfh-1, Brk, D-six4, Eya, Hbr, Mef2, Poxm

Delta, En, Hh, Med, Pyr, Su\_H\_CSL, Ths, Ci, Htl, Nicd, Twi(2), Zfh-1, Brk, D-six4, Eya, Mef2, Srp(2)

Med, Pyr, Su\_H\_CSL, Ths, Wg, Da, Htl, Pan, Slp, Twi(2), Zfh-1, Brk, D-six4, Eya, Hbr, Mef2, Poxm

### Genotype: Bap\_0; Srp\_2

Delta, Dpp, En, Hh, Med, Pyr, Shn, Su\_H\_CSL, Ths, Ci, Htl, Mad, Nicd, Tin, Mef2, Srp(2)

Delta, Dpp, Med, Pyr, Shn, Spi, Su\_H\_CSL, Ths, Upd, Wg, Doc, E\_Spl, Htl, Mad, Nicd, Pan, Slp, Stat92E, Tin(2), Twi, Zfh-1, Eve, Eya, Hbr, Mef2, Pnr, Srp(2)

Delta, En, Hh, Med, Pyr, Su\_H\_CSL, Ths, Ci, Htl, Nicd, Brk, Srp(2)

Med, Pyr, Su\_H\_CSL, Ths, Wg, Da, Htl, Pan, Slp, Twi(2), Zfh-1, Brk, D-six4, Eya, Hbr, Mef2, Poxm, Srp(2)

### Genotype: Bap\_0; Srp\_0

Delta, Dpp, En, Hh, Med, Pyr, Shn, Su\_H\_CSL, Ths, Ci, Htl, Mad, Nicd, Tin, Mef2

Delta, Dpp, Med, Pyr, Shn, Spi, Su\_H\_CSL, Ths, Upd, Wg, Doc, E\_Spl, Htl, Mad, Nicd, Pan, Slp, Stat92E, Tin(2), Twi, Zfh-1, Eve, Eya, Hbr, Mef2, Pnr

Delta, En, Hh, Med, Pyr, Su\_H\_CSL, Ths, Ci, Htl, Nicd, Brk

Med, Pyr, Su\_H\_CSL, Ths, Wg, Da, Htl, Pan, Slp, Twi(2), Zfh-1, Brk, D-six4, Eya, Hbr, Mef2, Poxm

### Genotype: Bin\_2; Doc\_1

Delta, Dpp, En, Hh, Med, Pyr, Shn, Su\_H\_CSL, Ths, Bap(3), Bin(2), Ci, Doc, Htl, Mad, Nicd, Tin, Mef2, Pnr

Delta, Dpp, Med, Pyr, Shn, Spi, Su\_H\_CSL, Ths, Upd, Wg, Bap(3), Bin(2), Doc, E\_Spl, Htl, Mad, Nicd, Pan, Slp, Stat92E, Tin(2), Twi, Zfh-1, Eve, Eya, Hbr, Mef2, Pnr

Delta, En, Hh, Med, Pyr, Su\_H\_CSL, Ths, Bap(3), Bin(2), Ci, Doc, Htl, Nicd, Brk, Srp(2)

Med, Pyr, Su\_H\_CSL, Ths, Wg, Bap(3), Bin(2), Da, Doc, Htl, Pan, Slp, Twi(2), Zfh-1, Brk, D-six4, Eya, Hbr, Mef2, Poxm

### Genotype: Bin\_2; Doc\_0

Delta, Dpp, En, Hh, Med, Pyr, Shn, Su\_H\_CSL, Ths, Bap(3), Bin(2), Ci, Htl, Mad, Nicd, Tin, Mef2

Delta, Dpp, Med, Pyr, Shn, Spi, Su\_H\_CSL, Ths, Upd, Wg, Bap(3), Bin(2), E\_Spl, Htl, Mad, Nicd, Pan, Slp, Stat92E, Tin(2), Twi, Zfh-1, Eve, Eya, Hbr, Mef2

Delta, En, Hh, Med, Pyr, Su\_H\_CSL, Ths, Bap(3), Bin(2), Ci, Htl, Nicd, Brk, Srp(2)

Med, Pyr, Su\_H\_CSL, Ths, Wg, Bap(3), Bin(2), Da, Htl, Pan, Slp, Twi(2), Zfh-1, Brk, D-six4, Eya, Hbr, Mef2, Poxm

### Genotype: Ci\_1; Tin\_2

Delta, Dpp, En, Hh, Med, Pyr, Shn, Su\_H\_CSL, Ths, Bap(3), Bin, Ci, E\_Spl, Htl, Mad, Nicd, Stat92E, Tin(2), Eya, Mef2

Delta, Dpp, Med, Pyr, Shn, Spi, Su\_H\_CSL, Ths, Upd, Wg, Bap, Ci, Doc, E\_Spl, Htl, Mad, Nicd, Pan, Slp, Stat92E, Tin(2), Twi, Zfh-1, Eve, Eya, Hbr, Mef2, Pnr

Delta, En, Hh, Med, Pyr, Su\_H\_CSL, Ths, Bap(2), Bin, Ci, E\_Spl, Htl, Nicd, Stat92E, Tin(2), Brk, D-six4, Eya, Mef2, Srp(2)

Med, Pyr, Su\_H\_CSL, Ths, Wg, Ci, Da, Htl, Pan, Slp, Stat92E, Tin(2), Twi(2), Zfh-1, Brk, D-six4, Eya, Hbr, Mef2, Poxm, Srp

### Genotype: Ci\_1; Tin\_0

Delta, Dpp, En, Hh, Med, Pyr, Shn, Su\_H\_CSL, Ths, Ci, Htl, Mad, Nicd

Delta, Dpp, Med, Pyr, Shn, Spi, Su\_H\_CSL, Ths, Upd, Wg, Ci, Doc, E\_Spl, Htl, Mad, Nicd, Pan, Slp, Stat92E, Twi, Zfh-1, Eya, Hbr

Delta, En, Hh, Med, Pyr, Su\_H\_CSL, Ths, Ci, Htl, Nicd, Brk, Srp(2)

Med, Pyr, Su\_H\_CSL, Ths, Wg, Ci, Da, Htl, Pan, Slp, Twi(2), Zfh-1, Brk, D-six4, Eya, Hbr, Mef2, Poxm, Srp

### Genotype: Bin\_0; Med\_1

Delta, Dpp, En, Hh, Med, Pyr, Shn, Su\_H\_CSL, Ths, Bap(3), Ci, Htl, Mad, Nicd, Tin, Mef2

Delta, Dpp, Med, Pyr, Shn, Spi, Su\_H\_CSL, Ths, Upd, Wg, Doc, E\_Spl, Htl, Mad, Nicd, Pan, Slp, Stat92E, Tin(2), Twi, Zfh-1, Eve, Eya, Hbr, Mef2, Pnr

Delta, En, Hh, Med, Pyr, Su\_H\_CSL, Ths, Ci, Htl, Nicd, Brk, Srp(2)

Med, Pyr, Su\_H\_CSL, Ths, Wg, Da, Htl, Pan, Slp, Twi(2), Zfh-1, Brk, D-six4, Eya, Hbr, Mef2, Poxm

### Genotype: Bin\_0; Med\_0

Delta, Dpp, En, Hh, Pyr, Shn, Su\_H\_CSL, Ths, Bap(2), Ci, Htl, Mad, Nicd, Tin, Brk, D-six4, Mef2, Srp(2)

Delta, Dpp, Pyr, Shn, Spi, Su\_H\_CSL, Ths, Upd, Wg, E\_Spl, Htl, Mad, Nicd, Pan, Slp, Stat92E, Tin, Twi, Zfh-1, Brk, D-six4, Eya, Hbr, Mef2, Poxm

Delta, En, Hh, Pyr, Su\_H\_CSL, Ths, Ci, Htl, Nicd, Brk, Srp(2)

Pyr, Su\_H\_CSL, Ths, Wg, Da, Htl, Pan, Slp, Twi(2), Zfh-1, Brk, D-six4, Eya, Hbr, Mef2, Poxm

### Genotype: Bin\_2; Mad\_1

Delta, Dpp, En, Hh, Med, Pyr, Shn, Su\_H\_CSL, Ths, Bap(3), Bin(2), Ci, Htl, Mad, Nicd, Tin, Mef2

Delta, Dpp, Med, Pyr, Shn, Spi, Su\_H\_CSL, Ths, Upd, Wg, Bap(3), Bin(2), Doc, E\_Spl, Htl, Mad, Nicd, Pan, Slp, Stat92E, Tin(2), Twi, Zfh-1, Eve, Eya, Hbr, Mef2, Pnr

Delta, En, Hh, Med, Pyr, Su\_H\_CSL, Ths, Bap(3), Bin(2), Ci, Htl, Mad, Nicd, Tin, Brk, Mef2

Med, Pyr, Su\_H\_CSL, Ths, Wg, Bap(3), Bin(2), Da, Doc, Htl, Mad, Pan, Slp, Tin, Twi(2), Zfh-1, Brk, Eya, Hbr, Mef2, Pnr

### Genotype: Bin\_2; Mad\_0

Delta, Dpp, En, Hh, Med, Pyr, Shn, Su\_H\_CSL, Ths, Bap(3), Bin(2), Ci, Htl, Nicd, Brk, Srp(2)

Delta, Dpp, Med, Pyr, Shn, Spi, Su\_H\_CSL, Ths, Upd, Wg, Bap(3), Bin(2), E\_Spl, Htl, Nicd, Pan, Slp, Stat92E, Twi, Zfh-1, Brk, D-six4, Eya, Hbr, Poxm

Delta, En, Hh, Med, Pyr, Su\_H\_CSL, Ths, Bap(3), Bin(2), Ci, Htl, Nicd, Brk, Srp(2)

Med, Pyr, Su\_H\_CSL, Ths, Wg, Bap(3), Bin(2), Da, Htl, Pan, Slp, Twi(2), Zfh-1, Brk, D-six4, Eya, Hbr, Mef2, Poxm

### Genotype: Bin\_0; Pan\_0

Delta, Dpp, En, Hh, Med, Pyr, Shn, Su\_H\_CSL, Ths, Bap(3), Ci, Htl, Mad, Nicd, Tin, Mef2

Delta, Dpp, Med, Pyr, Shn, Spi, Su\_H\_CSL, Ths, Upd, Wg, Bap, E\_Spl, Htl, Mad, Nicd, Stat92E, Tin, Mef2

Delta, En, Hh, Med, Pyr, Su\_H\_CSL, Ths, Ci, Htl, Nicd, Brk, Srp(2)

Med, Pyr, Su\_H\_CSL, Ths, Wg, Da, Htl, Brk

Med, Pyr, Su\_H\_CSL, Ths, Wg, Da, Htl, Twi, Zfh-1, Brk, D-six4, Eya

### Genotype: Bin\_0; Pan\_1

Delta, Dpp, En, Hh, Med, Pyr, Shn, Su\_H\_CSL, Ths, Bap, Ci, Doc, Htl, Mad, Nicd, Pan, Slp, Tin, Hbr, Mef2, Pnr

Delta, Dpp, En, Hh, Med, Pyr, Shn, Su\_H\_CSL, Ths, Bap, Ci, Doc, Htl, Mad, Nicd, Pan, Slp, Tin, Twi, Zfh-1, Eya, Hbr, Mef2, Pnr

Delta, Dpp, Med, Pyr, Shn, Spi, Su\_H\_CSL, Ths, Upd, Wg, Doc, E\_Spl, Htl, Mad, Nicd, Pan, Slp, Stat92E, Tin(2), Twi, Zfh-1, Eve, Eya, Hbr, Mef2, Pnr

Delta, En, Hh, Med, Pyr, Su\_H\_CSL, Ths, Ci, Htl, Nicd, Pan, Slp, Brk, Hbr, Srp(2)

Delta, En, Hh, Med, Pyr, Su\_H\_CSL, Ths, Ci, Htl, Nicd, Pan, Slp, Twi, Zfh-1, Brk, D-six4, Eya, Hbr, Poxm, Srp(2)

Med, Pyr, Su\_H\_CSL, Ths, Wg, Da, Htl, Pan, Slp, Twi(2), Zfh-1, Brk, D-six4, Eya, Hbr, Mef2, Poxm

### Genotype: Pnr\_1; Srp\_2

Delta, Dpp, En, Hh, Med, Pyr, Shn, Su\_H\_CSL, Ths, Bap(3), Bin, Ci, Htl, Mad, Nicd, Tin, Mef2, Pnr, Srp(2)

Delta, Dpp, Med, Pyr, Shn, Spi, Su\_H\_CSL, Ths, Upd, Wg, Doc, E\_Spl, Htl, Mad, Nicd, Pan, Slp, Stat92E, Tin(2), Twi, Zfh-1, Eve, Eya, Hbr, Mef2, Pnr, Srp(2)

Delta, En, Hh, Med, Pyr, Su\_H\_CSL, Ths, Ci, Htl, Nicd, Brk, Pnr, Srp(2)

Med, Pyr, Su\_H\_CSL, Ths, Wg, Da, Htl, Pan, Slp, Twi(2), Zfh-1, Brk, D-six4, Eya, Hbr, Mef2, Pnr, Poxm, Srp(2)

### Genotype: Pnr\_1; Srp\_0

Delta, Dpp, En, Hh, Med, Pyr, Shn, Su\_H\_CSL, Ths, Bap(3), Bin, Ci, Htl, Mad, Nicd, Tin, Mef2, Pnr

Delta, Dpp, Med, Pyr, Shn, Spi, Su\_H\_CSL, Ths, Upd, Wg, Doc, E\_Spl, Htl, Mad, Nicd, Pan, Slp, Stat92E, Tin(2), Twi, Zfh-1, Eve, Eya, Hbr, Mef2, Pnr

Delta, En, Hh, Med, Pyr, Su\_H\_CSL, Ths, Ci, Htl, Nicd, Brk, Pnr

Med, Pyr, Su\_H\_CSL, Ths, Wg, Da, Htl, Pan, Slp, Twi(2), Zfh-1, Brk, D-six4, Eya, Hbr, Mef2, Pnr, Poxm

### Genotype: Med\_0; Nicd\_1

Delta, Dpp, En, Hh, Pyr, Shn, Su\_H\_CSL, Ths, Bap(2), Bin, Ci, Htl, Mad, Nicd, Tin, Brk, D-six4, Mef2, Srp(2)

Delta, Dpp, Pyr, Shn, Spi, Su\_H\_CSL, Ths, Upd, Wg, E\_Spl, Htl, Mad, Nicd, Pan, Slp, Stat92E, Tin, Twi, Zfh-1, Brk, D-six4, Eya, Hbr, Mef2, Poxm

Delta, En, Hh, Pyr, Su\_H\_CSL, Ths, Ci, Htl, Nicd, Brk, Srp(2)

Pyr, Su\_H\_CSL, Ths, Wg, Htl, Nicd, Pan, Slp, Twi, Zfh-1, Brk, D-six4, Eya, Hbr, Poxm

### Genotype: Med\_0; Nicd\_0

Delta, Dpp, En, Hh, Pyr, Shn, Su\_H\_CSL, Ths, Bap(2), Bin, Ci, Da, Htl, Mad, Tin, Brk, D-six4, Mef2, Srp(2)

Delta, Dpp, En, Hh, Pyr, Shn, Su\_H\_CSL, Ths, Bap(2), Bin, Ci, Da, Htl, Mad, Tin, Twi, Zfh-1, Brk, D-six4, Eya, Mef2, Srp(2)

Delta, Dpp, Pyr, Shn, Spi, Su\_H\_CSL, Ths, Upd, Wg, Da, Htl, Mad, Pan, Slp, Stat92E, Tin, Twi(2), Zfh-1, Brk, D-six4, Eya, Hbr, Mef2, Poxm

Delta, En, Hh, Pyr, Su\_H\_CSL, Ths, Ci, Da, Htl, Brk, Srp(2)

Delta, En, Hh, Pyr, Su\_H\_CSL, Ths, Ci, Da, Htl, Twi, Zfh-1, Brk, D-six4, Eya, Srp(2)

Pyr, Su\_H\_CSL, Ths, Wg, Da, Htl, Pan, Slp, Twi(2), Zfh-1, Brk, D-six4, Eya, Hbr, Mef2, Poxm

### Genotype: Mef2\_0; Pnr\_1

Delta, Dpp, En, Hh, Med, Pyr, Shn, Su\_H\_CSL, Ths, Bap(3), Bin, Ci, Htl, Mad, Nicd, Tin, Pnr

Delta, Dpp, Med, Pyr, Shn, Spi, Su\_H\_CSL, Ths, Upd, Wg, Doc, E\_Spl, Htl, Mad, Nicd, Pan, Slp, Stat92E, Tin(2), Twi, Zfh-1, Eve, Eya, Hbr, Pnr

Delta, En, Hh, Med, Pyr, Su\_H\_CSL, Ths, Ci, Htl, Nicd, Brk, Pnr, Srp(2)

Med, Pyr, Su\_H\_CSL, Ths, Wg, Da, Htl, Pan, Slp, Twi(2), Zfh-1, Brk, D-six4, Eya, Hbr, Pnr, Poxm

### Genotype: Mef2\_0; Pnr\_0

Delta, Dpp, En, Hh, Med, Pyr, Shn, Su\_H\_CSL, Ths, Bap(3), Bin, Ci, Htl, Mad, Nicd, Tin

Delta, Dpp, Med, Pyr, Shn, Spi, Su\_H\_CSL, Ths, Upd, Wg, Doc, E\_Spl, Htl, Mad, Nicd, Pan, Slp, Stat92E, Tin(2), Twi, Zfh-1, Eve, Eya, Hbr

Delta, En, Hh, Med, Pyr, Su\_H\_CSL, Ths, Ci, Htl, Nicd, Brk, Srp(2)

Med, Pyr, Su\_H\_CSL, Ths, Wg, Da, Htl, Pan, Slp, Twi(2), Zfh-1, Brk, D-six4, Eya, Hbr, Poxm

### Genotype: Pnr\_1; Twi\_2

Delta, Dpp, En, Hh, Med, Pyr, Shn, Su\_H\_CSL, Ths, Bap(3), Bin, Ci, Htl, Mad, Nicd, Tin, Twi(2), Zfh-1, Eya, Mef2, Pnr

Delta, Dpp, Med, Pyr, Shn, Spi, Su\_H\_CSL, Ths, Upd, Wg, Doc, E\_Spl, Htl, Mad, Nicd, Pan, Slp, Stat92E, Tin(2), Twi(2), Zfh-1, Eve, Eya, Hbr, Mef2, Pnr

Delta, En, Hh, Med, Pyr, Su\_H\_CSL, Ths, Ci, Htl, Nicd, Twi(2), Zfh-1, Brk, D-six4, Eya, Mef2, Pnr, Srp(2)

Med, Pyr, Su\_H\_CSL, Ths, Wg, Da, Htl, Pan, Slp, Twi(2), Zfh-1, Brk, D-six4, Eya, Hbr, Mef2, Pnr, Poxm

### Genotype: Pnr\_1; Twi\_0

Delta, Dpp, En, Hh, Med, Pyr, Shn, Su\_H\_CSL, Ths, Bap(3), Bin, Ci, Htl, Mad, Nicd, Tin, Mef2, Pnr

Delta, Dpp, Med, Pyr, Shn, Spi, Su\_H\_CSL, Ths, Upd, Wg, Doc, E\_Spl, Htl, Mad, Nicd, Pan, Slp, Stat92E, Tin(2), Eya, Hbr, Mef2, Pnr

Delta, En, Hh, Med, Pyr, Su\_H\_CSL, Ths, Ci, Htl, Nicd, Brk, Pnr, Srp(2)

Med, Pyr, Su\_H\_CSL, Ths, Wg, Da, Htl, Pan, Slp, Brk, Hbr, Pnr

### Genotype: Doc\_1; Mef2\_0

Delta, Dpp, En, Hh, Med, Pyr, Shn, Su\_H\_CSL, Ths, Bap(3), Bin, Ci, Doc, Htl, Mad, Nicd, Tin, Pnr

Delta, Dpp, Med, Pyr, Shn, Spi, Su\_H\_CSL, Ths, Upd, Wg, Doc, E\_Spl, Htl, Mad, Nicd, Pan, Slp, Stat92E, Tin(2), Twi, Zfh-1, Eve, Eya, Hbr, Pnr

Delta, En, Hh, Med, Pyr, Su\_H\_CSL, Ths, Ci, Doc, Htl, Nicd, Brk, Srp(2)

Med, Pyr, Su\_H\_CSL, Ths, Wg, Da, Doc, Htl, Pan, Slp, Twi(2), Zfh-1, Brk, D-six4, Eya, Hbr, Poxm

### Genotype: Doc\_1; Mef2\_1

Delta, Dpp, En, Hh, Med, Pyr, Shn, Su\_H\_CSL, Ths, Bap(3), Bin, Ci, Doc, Htl, Mad, Nicd, Tin, Mef2, Pnr

Delta, Dpp, Med, Pyr, Shn, Spi, Su\_H\_CSL, Ths, Upd, Wg, Doc, E\_Spl, Htl, Mad, Nicd, Pan, Slp, Stat92E, Tin(2), Twi, Zfh-1, Eve, Eya, Hbr, Mef2, Pnr

Delta, En, Hh, Med, Pyr, Su\_H\_CSL, Ths, Ci, Doc, Htl, Nicd, Brk, Mef2, Srp(2)

Med, Pyr, Su\_H\_CSL, Ths, Wg, Da, Doc, Htl, Pan, Slp, Twi(2), Zfh-1, Brk, D-six4, Eya, Hbr, Mef2, Poxm

### Genotype: Mad\_0; Tin\_0

Delta, Dpp, En, Hh, Med, Pyr, Shn, Su\_H\_CSL, Ths, Ci, Htl, Nicd, Brk, Srp(2)

Delta, Dpp, Med, Pyr, Shn, Spi, Su\_H\_CSL, Ths, Upd, Wg, E\_Spl, Htl, Nicd, Pan, Slp, Stat92E, Twi, Zfh-1, Brk, D-six4, Eya, Hbr, Poxm

Delta, En, Hh, Med, Pyr, Su\_H\_CSL, Ths, Ci, Htl, Nicd, Brk, Srp(2)

Med, Pyr, Su\_H\_CSL, Ths, Wg, Da, Htl, Pan, Slp, Twi(2), Zfh-1, Brk, D-six4, Eya, Hbr, Mef2, Poxm

### Genotype: Mad\_0; Tin\_2

Delta, Dpp, En, Hh, Med, Pyr, Shn, Su\_H\_CSL, Ths, Bap(2), Bin, Ci, E\_Spl, Htl, Nicd, Stat92E, Tin(2), Brk, D-six4, Eya, Mef2, Srp(2)

Delta, Dpp, Med, Pyr, Shn, Spi, Su\_H\_CSL, Ths, Upd, Wg, E\_Spl, Htl, Nicd, Pan, Slp, Stat92E, Tin(2), Twi, Zfh-1, Brk, D-six4, Eya, Hbr, Mef2, Poxm

Delta, En, Hh, Med, Pyr, Su\_H\_CSL, Ths, Bap(2), Bin, Ci, E\_Spl, Htl, Nicd, Stat92E, Tin(2), Brk, D-six4, Eya, Mef2, Srp(2)

Med, Pyr, Su\_H\_CSL, Ths, Wg, Da, Htl, Pan, Slp, Stat92E, Tin(2), Twi(2), Zfh-1, Brk, D-six4, Eya, Hbr, Mef2, Poxm

### Genotype: Med\_1

Delta, Dpp, En, Hh, Med, Pyr, Shn, Su\_H\_CSL, Ths, Bap(3), Bin, Ci, Htl, Mad, Nicd, Tin, Mef2

Delta, Dpp, Med, Pyr, Shn, Spi, Su\_H\_CSL, Ths, Upd, Wg, Doc, E\_Spl, Htl, Mad, Nicd, Pan, Slp, Stat92E, Tin(2), Twi, Zfh-1, Eve, Eya, Hbr, Mef2, Pnr

Delta, En, Hh, Med, Pyr, Su\_H\_CSL, Ths, Ci, Htl, Nicd, Brk, Srp(2)

Med, Pyr, Su\_H\_CSL, Ths, Wg, Da, Htl, Pan, Slp, Twi(2), Zfh-1, Brk, D-six4, Eya, Hbr, Mef2, Poxm

### Genotype: Doc\_1; Slp\_0

Delta, Dpp, En, Hh, Med, Pyr, Shn, Su\_H\_CSL, Ths, Bap(3), Bin, Ci, Doc, Htl, Mad, Nicd, Tin, Mef2, Pnr

Delta, Dpp, En, Med, Pyr, Shn, Spi, Su\_H\_CSL, Ths, Upd, Wg, Bap, Doc, E\_Spl, Htl, Mad, Nicd, Pan, Stat92E, Tin(2), Eya, Hbr, Mef2, Pnr

Delta, En, Hh, Med, Pyr, Su\_H\_CSL, Ths, Ci, Doc, Htl, Nicd, Brk, Srp(2)

En, Med, Pyr, Su\_H\_CSL, Ths, Wg, Da, Doc, Htl, Pan, Brk, Hbr

En, Med, Pyr, Su\_H\_CSL, Ths, Wg, Da, Doc, Htl, Pan, Twi, Zfh-1, Brk, D-six4, Eya, Hbr, Poxm

### Genotype: Doc\_1; Slp\_1

Delta, Dpp, Hh, Med, Pyr, Shn, Su\_H\_CSL, Ths, Bap, Ci, Doc, Htl, Mad, Nicd, Slp, Tin, Mef2, Pnr

Delta, Dpp, Hh, Med, Pyr, Shn, Su\_H\_CSL, Ths, Bap, Ci, Doc, Htl, Mad, Nicd, Slp, Tin, Twi, Zfh-1, Eya, Mef2, Pnr

Delta, Dpp, Med, Pyr, Shn, Spi, Su\_H\_CSL, Ths, Upd, Wg, Doc, E\_Spl, Htl, Mad, Nicd, Pan, Slp, Stat92E, Tin(2), Twi, Zfh-1, Eve, Eya, Hbr, Mef2, Pnr

Delta, Hh, Med, Pyr, Su\_H\_CSL, Ths, Ci, Doc, Htl, Nicd, Slp, Brk, Srp

Delta, Hh, Med, Pyr, Su\_H\_CSL, Ths, Ci, Doc, Htl, Nicd, Slp, Twi, Zfh-1, Brk, D-six4, Eya, Srp

Med, Pyr, Su\_H\_CSL, Ths, Wg, Da, Doc, Htl, Pan, Slp, Twi(2), Zfh-1, Brk, D-six4, Eya, Hbr, Mef2, Poxm

### Genotype: Doc\_0; Med\_0

Delta, Dpp, En, Hh, Pyr, Shn, Su\_H\_CSL, Ths, Bap(2), Bin, Ci, Htl, Mad, Nicd, Tin, Brk, D-six4, Mef2, Srp(2)

Delta, Dpp, Pyr, Shn, Spi, Su\_H\_CSL, Ths, Upd, Wg, E\_Spl, Htl, Mad, Nicd, Pan, Slp, Stat92E, Tin, Twi, Zfh-1, Brk, D-six4, Eya, Hbr, Mef2, Poxm

Delta, En, Hh, Pyr, Su\_H\_CSL, Ths, Ci, Htl, Nicd, Brk, Srp(2)

Pyr, Su\_H\_CSL, Ths, Wg, Da, Htl, Pan, Slp, Twi(2), Zfh-1, Brk, D-six4, Eya, Hbr, Mef2, Poxm

### Genotype: Ci\_1; Srp\_2

Delta, Dpp, En, Hh, Med, Pyr, Shn, Su\_H\_CSL, Ths, Bap(3), Bin, Ci, Htl, Mad, Nicd, Tin, Mef2, Srp(2)

Delta, Dpp, Med, Pyr, Shn, Spi, Su\_H\_CSL, Ths, Upd, Wg, Bap, Ci, Doc, E\_Spl, Htl, Mad, Nicd, Pan, Slp, Stat92E, Tin(2), Twi, Zfh-1, Eve, Eya, Hbr, Mef2, Pnr, Srp(2)

Delta, En, Hh, Med, Pyr, Su\_H\_CSL, Ths, Ci, Htl, Nicd, Brk, Srp(2)

Med, Pyr, Su\_H\_CSL, Ths, Wg, Ci, Da, Htl, Pan, Slp, Twi(2), Zfh-1, Brk, D-six4, Eya, Hbr, Mef2, Poxm, Srp(2)

### Genotype: Mad\_1; Tin\_0

Delta, Dpp, En, Hh, Med, Pyr, Shn, Su\_H\_CSL, Ths, Ci, Htl, Mad, Nicd

Delta, Dpp, Med, Pyr, Shn, Spi, Su\_H\_CSL, Ths, Upd, Wg, Doc, E\_Spl, Htl, Mad, Nicd, Pan, Slp, Stat92E, Twi, Zfh-1, Eya, Hbr

Delta, En, Hh, Med, Pyr, Su\_H\_CSL, Ths, Ci, Htl, Mad, Nicd, Brk

Med, Pyr, Su\_H\_CSL, Ths, Wg, Da, Doc, Htl, Mad, Pan, Slp, Twi(2), Zfh-1, Brk, Eya, Hbr, Mef2

### Genotype: Mad\_1; Tin\_2

Delta, Dpp, En, Hh, Med, Pyr, Shn, Su\_H\_CSL, Ths, Bap(3), Bin, Ci, E\_Spl, Htl, Mad, Nicd, Stat92E, Tin(2), Eya, Mef2

Delta, Dpp, Med, Pyr, Shn, Spi, Su\_H\_CSL, Ths, Upd, Wg, Doc, E\_Spl, Htl, Mad, Nicd, Pan, Slp, Stat92E, Tin(2), Twi, Zfh-1, Eve, Eya, Hbr, Mef2, Pnr

Delta, En, Hh, Med, Pyr, Su\_H\_CSL, Ths, Bap(3), Bin, Ci, E\_Spl, Htl, Mad, Nicd, Stat92E, Tin(2), Brk, Eya, Mef2

Med, Pyr, Su\_H\_CSL, Ths, Wg, Da, Doc, Htl, Mad, Pan, Slp, Stat92E, Tin(2), Twi(2), Zfh-1, Brk, Eya, Hbr, Mef2, Pnr

### Genotype: Bin\_2; Slp\_0

Delta, Dpp, En, Hh, Med, Pyr, Shn, Su\_H\_CSL, Ths, Bap(3), Bin(2), Ci, Htl, Mad, Nicd, Tin, Mef2

Delta, Dpp, En, Med, Pyr, Shn, Spi, Su\_H\_CSL, Ths, Upd, Wg, Bap(3), Bin(2), Doc, E\_Spl, Htl, Mad, Nicd, Pan, Stat92E, Tin(2), Eya, Hbr, Mef2, Pnr

Delta, En, Hh, Med, Pyr, Su\_H\_CSL, Ths, Bap(3), Bin(2), Ci, Htl, Nicd, Brk, Srp(2)

En, Med, Pyr, Su\_H\_CSL, Ths, Wg, Bap(3), Bin(2), Da, Htl, Pan, Brk, Hbr

En, Med, Pyr, Su\_H\_CSL, Ths, Wg, Bap(3), Bin(2), Da, Htl, Pan, Twi, Zfh-1, Brk, D-six4, Eya, Hbr, Poxm

### Genotype: Bin\_2; Slp\_1

Delta, Dpp, Hh, Med, Pyr, Shn, Su\_H\_CSL, Ths, Bap(3), Bin(2), Ci, Htl, Mad, Nicd, Slp, Tin, Mef2

Delta, Dpp, Hh, Med, Pyr, Shn, Su\_H\_CSL, Ths, Bap(3), Bin(2), Ci, Htl, Mad, Nicd, Slp, Tin, Twi, Zfh-1, Eya, Mef2

Delta, Dpp, Med, Pyr, Shn, Spi, Su\_H\_CSL, Ths, Upd, Wg, Bap(3), Bin(2), Doc, E\_Spl, Htl, Mad, Nicd, Pan, Slp, Stat92E, Tin(2), Twi, Zfh-1, Eve, Eya, Hbr, Mef2, Pnr

Delta, Hh, Med, Pyr, Su\_H\_CSL, Ths, Bap(3), Bin(2), Ci, Htl, Nicd, Slp, Brk, Srp

Delta, Hh, Med, Pyr, Su\_H\_CSL, Ths, Bap(3), Bin(2), Ci, Htl, Nicd, Slp, Twi, Zfh-1, Brk, D-six4, Eya, Srp

Med, Pyr, Su\_H\_CSL, Ths, Wg, Bap(3), Bin(2), Da, Htl, Pan, Slp, Twi(2), Zfh-1, Brk, D-six4, Eya, Hbr, Mef2, Poxm

### Genotype: Mef2\_0; Pan\_0

Delta, Dpp, En, Hh, Med, Pyr, Shn, Su\_H\_CSL, Ths, Bap(3), Bin, Ci, Htl, Mad, Nicd, Tin

Delta, Dpp, Med, Pyr, Shn, Spi, Su\_H\_CSL, Ths, Upd, Wg, Bap, E\_Spl, Htl, Mad, Nicd, Stat92E, Tin

Delta, En, Hh, Med, Pyr, Su\_H\_CSL, Ths, Ci, Htl, Nicd, Brk, Srp(2)

Med, Pyr, Su\_H\_CSL, Ths, Wg, Da, Htl, Brk

Med, Pyr, Su\_H\_CSL, Ths, Wg, Da, Htl, Twi, Zfh-1, Brk, D-six4, Eya

### Genotype: Mef2\_0; Pan\_1

Delta, Dpp, En, Hh, Med, Pyr, Shn, Su\_H\_CSL, Ths, Bap, Ci, Doc, Htl, Mad, Nicd, Pan, Slp, Tin, Hbr, Pnr

Delta, Dpp, En, Hh, Med, Pyr, Shn, Su\_H\_CSL, Ths, Bap, Ci, Doc, Htl, Mad, Nicd, Pan, Slp, Tin, Twi, Zfh-1, Eya, Hbr, Pnr

Delta, Dpp, Med, Pyr, Shn, Spi, Su\_H\_CSL, Ths, Upd, Wg, Doc, E\_Spl, Htl, Mad, Nicd, Pan, Slp, Stat92E, Tin(2), Twi, Zfh-1, Eve, Eya, Hbr, Pnr

Delta, En, Hh, Med, Pyr, Su\_H\_CSL, Ths, Ci, Htl, Nicd, Pan, Slp, Brk, Hbr, Srp(2)

Delta, En, Hh, Med, Pyr, Su\_H\_CSL, Ths, Ci, Htl, Nicd, Pan, Slp, Twi, Zfh-1, Brk, D-six4, Eya, Hbr, Poxm, Srp(2)

Med, Pyr, Su\_H\_CSL, Ths, Wg, Da, Htl, Pan, Slp, Twi(2), Zfh-1, Brk, D-six4, Eya, Hbr, Poxm

### Genotype: Mef2\_1; Pnr\_0

Delta, Dpp, En, Hh, Med, Pyr, Shn, Su\_H\_CSL, Ths, Bap(3), Bin, Ci, Htl, Mad, Nicd, Tin, Mef2

Delta, Dpp, Med, Pyr, Shn, Spi, Su\_H\_CSL, Ths, Upd, Wg, Doc, E\_Spl, Htl, Mad, Nicd, Pan, Slp, Stat92E, Tin(2), Twi, Zfh-1, Eve, Eya, Hbr, Mef2

Delta, En, Hh, Med, Pyr, Su\_H\_CSL, Ths, Ci, Htl, Nicd, Brk, Mef2, Srp(2)

Med, Pyr, Su\_H\_CSL, Ths, Wg, Da, Htl, Pan, Slp, Twi(2), Zfh-1, Brk, D-six4, Eya, Hbr, Mef2, Poxm

### Genotype: Mef2\_1; Pnr\_1

Delta, Dpp, En, Hh, Med, Pyr, Shn, Su\_H\_CSL, Ths, Bap(3), Bin, Ci, Htl, Mad, Nicd, Tin, Mef2, Pnr

Delta, Dpp, Med, Pyr, Shn, Spi, Su\_H\_CSL, Ths, Upd, Wg, Doc, E\_Spl, Htl, Mad, Nicd, Pan, Slp, Stat92E, Tin(2), Twi, Zfh-1, Eve, Eya, Hbr, Mef2, Pnr

Delta, En, Hh, Med, Pyr, Su\_H\_CSL, Ths, Ci, Htl, Nicd, Brk, Mef2, Pnr, Srp(2)

Med, Pyr, Su\_H\_CSL, Ths, Wg, Da, Htl, Pan, Slp, Twi(2), Zfh-1, Brk, D-six4, Eya, Hbr, Mef2, Pnr, Poxm

### Genotype: Bap\_3; Doc\_0

Delta, Dpp, En, Hh, Med, Pyr, Shn, Su\_H\_CSL, Ths, Bap(3), Bin, Ci, Htl, Mad, Nicd, Tin, Mef2

Delta, Dpp, Med, Pyr, Shn, Spi, Su\_H\_CSL, Ths, Upd, Wg, Bap(3), Bin, E\_Spl, Htl, Mad, Nicd, Pan, Slp, Stat92E, Tin(2), Twi, Zfh-1, Eve, Eya, Hbr, Mef2

Delta, En, Hh, Med, Pyr, Su\_H\_CSL, Ths, Bap(3), Bin, Ci, Htl, Nicd, Brk, Srp(2)

Med, Pyr, Su\_H\_CSL, Ths, Wg, Bap(3), Bin, Da, Htl, Pan, Slp, Twi(2), Zfh-1, Brk, D-six4, Eya, Hbr, Mef2, Poxm

### Genotype: Tin\_0

Delta, Dpp, En, Hh, Med, Pyr, Shn, Su\_H\_CSL, Ths, Ci, Htl, Mad, Nicd

Delta, Dpp, Med, Pyr, Shn, Spi, Su\_H\_CSL, Ths, Upd, Wg, Doc, E\_Spl, Htl, Mad, Nicd, Pan, Slp, Stat92E, Twi, Zfh-1, Eya, Hbr

Delta, En, Hh, Med, Pyr, Su\_H\_CSL, Ths, Ci, Htl, Nicd, Brk, Srp(2)

Med, Pyr, Su\_H\_CSL, Ths, Wg, Da, Htl, Pan, Slp, Twi(2), Zfh-1, Brk, D-six4, Eya, Hbr, Mef2, Poxm

### Genotype: Mef2\_1

Delta, Dpp, En, Hh, Med, Pyr, Shn, Su\_H\_CSL, Ths, Bap(3), Bin, Ci, Htl, Mad, Nicd, Tin, Mef2

Delta, Dpp, Med, Pyr, Shn, Spi, Su\_H\_CSL, Ths, Upd, Wg, Doc, E\_Spl, Htl, Mad, Nicd, Pan, Slp, Stat92E, Tin(2), Twi, Zfh-1, Eve, Eya, Hbr, Mef2, Pnr

Delta, En, Hh, Med, Pyr, Su\_H\_CSL, Ths, Ci, Htl, Nicd, Brk, Mef2, Srp(2)

Med, Pyr, Su\_H\_CSL, Ths, Wg, Da, Htl, Pan, Slp, Twi(2), Zfh-1, Brk, D-six4, Eya, Hbr, Mef2, Poxm

### Genotype: Bin\_0; Srp\_0

Delta, Dpp, En, Hh, Med, Pyr, Shn, Su\_H\_CSL, Ths, Bap(3), Ci, Htl, Mad, Nicd, Tin, Mef2

Delta, Dpp, Med, Pyr, Shn, Spi, Su\_H\_CSL, Ths, Upd, Wg, Doc, E\_Spl, Htl, Mad, Nicd, Pan, Slp, Stat92E, Tin(2), Twi, Zfh-1, Eve, Eya, Hbr, Mef2, Pnr

Delta, En, Hh, Med, Pyr, Su\_H\_CSL, Ths, Ci, Htl, Nicd, Brk

Med, Pyr, Su\_H\_CSL, Ths, Wg, Da, Htl, Pan, Slp, Twi(2), Zfh-1, Brk, D-six4, Eya, Hbr, Mef2, Poxm

### Genotype: Bin\_0; Srp\_2

Delta, Dpp, En, Hh, Med, Pyr, Shn, Su\_H\_CSL, Ths, Bap(3), Ci, Htl, Mad, Nicd, Tin, Mef2, Srp(2)

Delta, Dpp, Med, Pyr, Shn, Spi, Su\_H\_CSL, Ths, Upd, Wg, Doc, E\_Spl, Htl, Mad, Nicd, Pan, Slp, Stat92E, Tin(2), Twi, Zfh-1, Eve, Eya, Hbr, Mef2, Pnr, Srp(2)

Delta, En, Hh, Med, Pyr, Su\_H\_CSL, Ths, Ci, Htl, Nicd, Brk, Srp(2)

Med, Pyr, Su\_H\_CSL, Ths, Wg, Da, Htl, Pan, Slp, Twi(2), Zfh-1, Brk, D-six4, Eya, Hbr, Mef2, Poxm, Srp(2)

### Genotype: Bap\_3; Ci\_1

Delta, Dpp, En, Hh, Med, Pyr, Shn, Su\_H\_CSL, Ths, Bap(3), Bin, Ci, Htl, Mad, Nicd, Tin, Mef2

Delta, Dpp, Med, Pyr, Shn, Spi, Su\_H\_CSL, Ths, Upd, Wg, Bap(3), Bin, Ci, Doc, E\_Spl, Htl, Mad, Nicd, Pan, Slp, Stat92E, Tin(2), Twi, Zfh-1, Eve, Eya, Hbr, Mef2, Pnr

Delta, En, Hh, Med, Pyr, Su\_H\_CSL, Ths, Bap(3), Bin, Ci, Htl, Nicd, Brk, Srp(2)

Med, Pyr, Su\_H\_CSL, Ths, Wg, Bap(3), Bin, Ci, Da, Htl, Pan, Slp, Twi(2), Zfh-1, Brk, D-six4, Eya, Hbr, Mef2, Poxm, Srp

### Genotype: Bap\_3; Ci\_0

Delta, Dpp, En, Hh, Med, Pyr, Shn, Su\_H\_CSL, Ths, Bap(3), Bin, Htl, Mad, Nicd, Tin, Mef2

Delta, Dpp, Med, Pyr, Shn, Spi, Su\_H\_CSL, Ths, Upd, Wg, Bap(3), Bin, Doc, E\_Spl, Htl, Mad, Nicd, Pan, Slp, Stat92E, Tin(2), Twi, Zfh-1, Eve, Eya, Hbr, Mef2, Pnr

Delta, En, Hh, Med, Pyr, Su\_H\_CSL, Ths, Bap(3), Bin, Htl, Nicd, Brk

Med, Pyr, Su\_H\_CSL, Ths, Wg, Bap(3), Bin, Da, Htl, Pan, Slp, Twi(2), Zfh-1, Brk, D-six4, Eya, Hbr, Mef2, Poxm

### Genotype: Bin\_2; Nicd\_0

Delta, Dpp, En, Hh, Med, Pyr, Shn, Su\_H\_CSL, Ths, Bap(3), Bin(2), Ci, Da, Htl, Mad, Tin, Mef2

Delta, Dpp, En, Hh, Med, Pyr, Shn, Su\_H\_CSL, Ths, Bap(3), Bin(2), Ci, Da, Htl, Mad, Tin, Twi, Zfh-1, Eya, Mef2

Delta, Dpp, Med, Pyr, Shn, Spi, Su\_H\_CSL, Ths, Upd, Wg, Bap(3), Bin(2), Da, Doc, Htl, Mad, Pan, Slp, Stat92E, Tin(2), Twi(2), Zfh-1, Eve, Eya, Hbr, Mef2, Pnr

Delta, En, Hh, Med, Pyr, Su\_H\_CSL, Ths, Bap(3), Bin(2), Ci, Da, Htl, Brk, Srp(2)

Delta, En, Hh, Med, Pyr, Su\_H\_CSL, Ths, Bap(3), Bin(2), Ci, Da, Htl, Twi, Zfh-1, Brk, D-six4, Eya, Srp(2)

Med, Pyr, Su\_H\_CSL, Ths, Wg, Bap(3), Bin(2), Da, Htl, Pan, Slp, Twi(2), Zfh-1, Brk, D-six4, Eya, Hbr, Mef2, Poxm

### Genotype: Bin\_2; Nicd\_1

Delta, Dpp, En, Hh, Med, Pyr, Shn, Su\_H\_CSL, Ths, Bap(3), Bin(2), Ci, Htl, Mad, Nicd, Tin, Mef2

Delta, Dpp, Med, Pyr, Shn, Spi, Su\_H\_CSL, Ths, Upd, Wg, Bap(3), Bin(2), Doc, E\_Spl, Htl, Mad, Nicd, Pan, Slp, Stat92E, Tin(2), Twi, Zfh-1, Eve, Eya, Hbr, Mef2, Pnr

Delta, En, Hh, Med, Pyr, Su\_H\_CSL, Ths, Bap(3), Bin(2), Ci, Htl, Nicd, Brk, Srp(2)

Med, Pyr, Su\_H\_CSL, Ths, Wg, Bap(3), Bin(2), Htl, Nicd, Pan, Slp, Twi, Zfh-1, Brk, D-six4, Eya, Hbr, Poxm

### Genotype: Bap\_3; Mad\_0

Delta, Dpp, En, Hh, Med, Pyr, Shn, Su\_H\_CSL, Ths, Bap(3), Bin, Ci, Htl, Nicd, Brk, Srp(2)

Delta, Dpp, Med, Pyr, Shn, Spi, Su\_H\_CSL, Ths, Upd, Wg, Bap(3), Bin, E\_Spl, Htl, Nicd, Pan, Slp, Stat92E, Twi, Zfh-1, Brk, D-six4, Eya, Hbr, Poxm

Delta, En, Hh, Med, Pyr, Su\_H\_CSL, Ths, Bap(3), Bin, Ci, Htl, Nicd, Brk, Srp(2)

Med, Pyr, Su\_H\_CSL, Ths, Wg, Bap(3), Bin, Da, Htl, Pan, Slp, Twi(2), Zfh-1, Brk, D-six4, Eya, Hbr, Mef2, Poxm

### Genotype: Bap\_3; Mad\_1

Delta, Dpp, En, Hh, Med, Pyr, Shn, Su\_H\_CSL, Ths, Bap(3), Bin, Ci, Htl, Mad, Nicd, Tin, Mef2

Delta, Dpp, Med, Pyr, Shn, Spi, Su\_H\_CSL, Ths, Upd, Wg, Bap(3), Bin, Doc, E\_Spl, Htl, Mad, Nicd, Pan, Slp, Stat92E, Tin(2), Twi, Zfh-1, Eve, Eya, Hbr, Mef2, Pnr

Delta, En, Hh, Med, Pyr, Su\_H\_CSL, Ths, Bap(3), Bin, Ci, Htl, Mad, Nicd, Tin, Brk, Mef2

Med, Pyr, Su\_H\_CSL, Ths, Wg, Bap(3), Bin, Da, Doc, Htl, Mad, Pan, Slp, Tin, Twi(2), Zfh-1, Brk, Eya, Hbr, Mef2, Pnr

### Genotype: Srp\_2; Twi\_0

Delta, Dpp, En, Hh, Med, Pyr, Shn, Su\_H\_CSL, Ths, Bap(3), Bin, Ci, Htl, Mad, Nicd, Tin, Mef2, Srp(2)

Delta, Dpp, Med, Pyr, Shn, Spi, Su\_H\_CSL, Ths, Upd, Wg, Doc, E\_Spl, Htl, Mad, Nicd, Pan, Slp, Stat92E, Tin(2), Eya, Hbr, Mef2, Pnr, Srp(2)

Delta, En, Hh, Med, Pyr, Su\_H\_CSL, Ths, Ci, Htl, Nicd, Brk, Srp(2)

Med, Pyr, Su\_H\_CSL, Ths, Wg, Da, Htl, Pan, Slp, Brk, Hbr, Srp(2)

### Genotype: Srp\_2; Twi\_2

Delta, Dpp, En, Hh, Med, Pyr, Shn, Su\_H\_CSL, Ths, Bap(3), Bin, Ci, Htl, Mad, Nicd, Tin, Twi(2), Zfh-1, Eya, Mef2, Srp(2)

Delta, Dpp, Med, Pyr, Shn, Spi, Su\_H\_CSL, Ths, Upd, Wg, Doc, E\_Spl, Htl, Mad, Nicd, Pan, Slp, Stat92E, Tin(2), Twi(2), Zfh-1, Eve, Eya, Hbr, Mef2, Pnr, Srp(2)

Delta, En, Hh, Med, Pyr, Su\_H\_CSL, Ths, Ci, Htl, Nicd, Twi(2), Zfh-1, Brk, D-six4, Eya, Mef2, Srp(2)

Med, Pyr, Su\_H\_CSL, Ths, Wg, Da, Htl, Pan, Slp, Twi(2), Zfh-1, Brk, D-six4, Eya, Hbr, Mef2, Poxm, Srp(2)

### Genotype: Doc\_0; Tin\_0

Delta, Dpp, En, Hh, Med, Pyr, Shn, Su\_H\_CSL, Ths, Ci, Htl, Mad, Nicd

Delta, Dpp, Med, Pyr, Shn, Spi, Su\_H\_CSL, Ths, Upd, Wg, E\_Spl, Htl, Mad, Nicd, Pan, Slp, Stat92E, Twi, Zfh-1, Eya, Hbr

Delta, En, Hh, Med, Pyr, Su\_H\_CSL, Ths, Ci, Htl, Nicd, Brk, Srp(2)

Med, Pyr, Su\_H\_CSL, Ths, Wg, Da, Htl, Pan, Slp, Twi(2), Zfh-1, Brk, D-six4, Eya, Hbr, Mef2, Poxm

### Genotype: Doc\_0; Tin\_2

Delta, Dpp, En, Hh, Med, Pyr, Shn, Su\_H\_CSL, Ths, Bap(3), Bin, Ci, E\_Spl, Htl, Mad, Nicd, Stat92E, Tin(2), Eya, Mef2

Delta, Dpp, Med, Pyr, Shn, Spi, Su\_H\_CSL, Ths, Upd, Wg, E\_Spl, Htl, Mad, Nicd, Pan, Slp, Stat92E, Tin(2), Twi, Zfh-1, Eve, Eya, Hbr, Mef2

Delta, En, Hh, Med, Pyr, Su\_H\_CSL, Ths, Bap(2), Bin, Ci, E\_Spl, Htl, Nicd, Stat92E, Tin(2), Brk, D-six4, Eya, Mef2, Srp(2)

Med, Pyr, Su\_H\_CSL, Ths, Wg, Da, Htl, Pan, Slp, Stat92E, Tin(2), Twi(2), Zfh-1, Brk, D-six4, Eya, Hbr, Mef2, Poxm

### Genotype: Med\_1; Slp\_0

Delta, Dpp, En, Hh, Med, Pyr, Shn, Su\_H\_CSL, Ths, Bap(3), Bin, Ci, Htl, Mad, Nicd, Tin, Mef2

Delta, Dpp, En, Med, Pyr, Shn, Spi, Su\_H\_CSL, Ths, Upd, Wg, Bap, Doc, E\_Spl, Htl, Mad, Nicd, Pan, Stat92E, Tin(2), Eya, Hbr, Mef2, Pnr

Delta, En, Hh, Med, Pyr, Su\_H\_CSL, Ths, Ci, Htl, Nicd, Brk, Srp(2)

En, Med, Pyr, Su\_H\_CSL, Ths, Wg, Da, Htl, Pan, Brk, Hbr

En, Med, Pyr, Su\_H\_CSL, Ths, Wg, Da, Htl, Pan, Twi, Zfh-1, Brk, D-six4, Eya, Hbr, Poxm

### Genotype: Med\_1; Slp\_1

Delta, Dpp, Hh, Med, Pyr, Shn, Su\_H\_CSL, Ths, Bap, Ci, Htl, Mad, Nicd, Slp, Tin, Mef2

Delta, Dpp, Hh, Med, Pyr, Shn, Su\_H\_CSL, Ths, Bap, Ci, Htl, Mad, Nicd, Slp, Tin, Twi, Zfh-1, Eya, Mef2

Delta, Dpp, Med, Pyr, Shn, Spi, Su\_H\_CSL, Ths, Upd, Wg, Doc, E\_Spl, Htl, Mad, Nicd, Pan, Slp, Stat92E, Tin(2), Twi, Zfh-1, Eve, Eya, Hbr, Mef2, Pnr

Delta, Hh, Med, Pyr, Su\_H\_CSL, Ths, Ci, Htl, Nicd, Slp, Brk, Srp

Delta, Hh, Med, Pyr, Su\_H\_CSL, Ths, Ci, Htl, Nicd, Slp, Twi, Zfh-1, Brk, D-six4, Eya, Srp

Med, Pyr, Su\_H\_CSL, Ths, Wg, Da, Htl, Pan, Slp, Twi(2), Zfh-1, Brk, D-six4, Eya, Hbr, Mef2, Poxm

### Genotype: Doc\_0; Srp\_0

Delta, Dpp, En, Hh, Med, Pyr, Shn, Su\_H\_CSL, Ths, Bap(3), Bin, Ci, Htl, Mad, Nicd, Tin, Mef2

Delta, Dpp, Med, Pyr, Shn, Spi, Su\_H\_CSL, Ths, Upd, Wg, E\_Spl, Htl, Mad, Nicd, Pan, Slp, Stat92E, Tin(2), Twi, Zfh-1, Eve, Eya, Hbr, Mef2

Delta, En, Hh, Med, Pyr, Su\_H\_CSL, Ths, Ci, Htl, Nicd, Brk

Med, Pyr, Su\_H\_CSL, Ths, Wg, Da, Htl, Pan, Slp, Twi(2), Zfh-1, Brk, D-six4, Eya, Hbr, Mef2, Poxm

### Genotype: Bin\_0; Nicd\_0

Delta, Dpp, En, Hh, Med, Pyr, Shn, Su\_H\_CSL, Ths, Bap(3), Ci, Da, Htl, Mad, Tin, Mef2

Delta, Dpp, En, Hh, Med, Pyr, Shn, Su\_H\_CSL, Ths, Bap(3), Ci, Da, Htl, Mad, Tin, Twi, Zfh-1, Eya, Mef2

Delta, Dpp, Med, Pyr, Shn, Spi, Su\_H\_CSL, Ths, Upd, Wg, Da, Doc, Htl, Mad, Pan, Slp, Stat92E, Tin(2), Twi(2), Zfh-1, Eve, Eya, Hbr, Mef2, Pnr

Delta, En, Hh, Med, Pyr, Su\_H\_CSL, Ths, Ci, Da, Htl, Brk, Srp(2)

Delta, En, Hh, Med, Pyr, Su\_H\_CSL, Ths, Ci, Da, Htl, Twi, Zfh-1, Brk, D-six4, Eya, Srp(2)

Med, Pyr, Su\_H\_CSL, Ths, Wg, Da, Htl, Pan, Slp, Twi(2), Zfh-1, Brk, D-six4, Eya, Hbr, Mef2, Poxm

### Genotype: Bin\_0; Nicd\_1

Delta, Dpp, En, Hh, Med, Pyr, Shn, Su\_H\_CSL, Ths, Bap(3), Ci, Htl, Mad, Nicd, Tin, Mef2

Delta, Dpp, Med, Pyr, Shn, Spi, Su\_H\_CSL, Ths, Upd, Wg, Doc, E\_Spl, Htl, Mad, Nicd, Pan, Slp, Stat92E, Tin(2), Twi, Zfh-1, Eve, Eya, Hbr, Mef2, Pnr

Delta, En, Hh, Med, Pyr, Su\_H\_CSL, Ths, Ci, Htl, Nicd, Brk, Srp(2)

Med, Pyr, Su\_H\_CSL, Ths, Wg, Htl, Nicd, Pan, Slp, Twi, Zfh-1, Brk, D-six4, Eya, Hbr, Poxm

### Genotype: Bap\_3; Nicd\_1

Delta, Dpp, En, Hh, Med, Pyr, Shn, Su\_H\_CSL, Ths, Bap(3), Bin, Ci, Htl, Mad, Nicd, Tin, Mef2

Delta, Dpp, Med, Pyr, Shn, Spi, Su\_H\_CSL, Ths, Upd, Wg, Bap(3), Bin, Doc, E\_Spl, Htl, Mad, Nicd, Pan, Slp, Stat92E, Tin(2), Twi, Zfh-1, Eve, Eya, Hbr, Mef2, Pnr

Delta, En, Hh, Med, Pyr, Su\_H\_CSL, Ths, Bap(3), Bin, Ci, Htl, Nicd, Brk, Srp(2)

Med, Pyr, Su\_H\_CSL, Ths, Wg, Bap(3), Bin, Htl, Nicd, Pan, Slp, Twi, Zfh-1, Brk, D-six4, Eya, Hbr, Poxm

### Genotype: Bap\_3; Nicd\_0

Delta, Dpp, En, Hh, Med, Pyr, Shn, Su\_H\_CSL, Ths, Bap(3), Bin, Ci, Da, Htl, Mad, Tin, Mef2

Delta, Dpp, En, Hh, Med, Pyr, Shn, Su\_H\_CSL, Ths, Bap(3), Bin, Ci, Da, Htl, Mad, Tin, Twi, Zfh-1, Eya, Mef2

Delta, Dpp, Med, Pyr, Shn, Spi, Su\_H\_CSL, Ths, Upd, Wg, Bap(3), Bin, Da, Doc, Htl, Mad, Pan, Slp, Stat92E, Tin(2), Twi(2), Zfh-1, Eve, Eya, Hbr, Mef2, Pnr

Delta, En, Hh, Med, Pyr, Su\_H\_CSL, Ths, Bap(3), Bin, Ci, Da, Htl, Brk, Srp(2)

Delta, En, Hh, Med, Pyr, Su\_H\_CSL, Ths, Bap(3), Bin, Ci, Da, Htl, Twi, Zfh-1, Brk, D-six4, Eya, Srp(2)

Med, Pyr, Su\_H\_CSL, Ths, Wg, Bap(3), Bin, Da, Htl, Pan, Slp, Twi(2), Zfh-1, Brk, D-six4, Eya, Hbr, Mef2, Poxm

### Genotype: Pan\_0; Slp\_0

Delta, Dpp, En, Hh, Med, Pyr, Shn, Su\_H\_CSL, Ths, Bap(3), Bin, Ci, Htl, Mad, Nicd, Tin, Mef2

Delta, Dpp, En, Med, Pyr, Shn, Spi, Su\_H\_CSL, Ths, Upd, Wg, Bap, E\_Spl, Htl, Mad, Nicd, Stat92E, Tin, Mef2

Delta, En, Hh, Med, Pyr, Su\_H\_CSL, Ths, Ci, Htl, Nicd, Brk, Srp(2)

En, Med, Pyr, Su\_H\_CSL, Ths, Wg, Da, Htl, Brk

En, Med, Pyr, Su\_H\_CSL, Ths, Wg, Da, Htl, Twi, Zfh-1, Brk, D-six4, Eya

### Genotype: Nicd\_0; Twi\_2

Delta, Dpp, En, Hh, Med, Pyr, Shn, Su\_H\_CSL, Ths, Bap(3), Bin, Ci, Da, Htl, Mad, Tin, Twi(2), Zfh-1, Eya, Mef2

Delta, Dpp, Med, Pyr, Shn, Spi, Su\_H\_CSL, Ths, Upd, Wg, Da, Doc, Htl, Mad, Pan, Slp, Stat92E, Tin(2), Twi(2), Zfh-1, Eve, Eya, Hbr, Mef2, Pnr

Delta, En, Hh, Med, Pyr, Su\_H\_CSL, Ths, Ci, Da, Htl, Twi(2), Zfh-1, Brk, D-six4, Eya, Mef2, Srp(2)

Med, Pyr, Su\_H\_CSL, Ths, Wg, Da, Htl, Pan, Slp, Twi(2), Zfh-1, Brk, D-six4, Eya, Hbr, Mef2, Poxm

### Genotype: Nicd\_0; Twi\_0

Delta, Dpp, En, Hh, Med, Pyr, Shn, Su\_H\_CSL, Ths, Bap(3), Bin, Ci, Da, Htl, Mad, Tin, Mef2

Delta, Dpp, Med, Pyr, Shn, Spi, Su\_H\_CSL, Ths, Upd, Wg, Da, Doc, Htl, Mad, Pan, Slp, Stat92E, Tin(2), Eya, Hbr, Mef2, Pnr

Delta, En, Hh, Med, Pyr, Su\_H\_CSL, Ths, Ci, Da, Htl, Brk, Srp(2)

Med, Pyr, Su\_H\_CSL, Ths, Wg, Da, Htl, Pan, Slp, Brk, Hbr

### Genotype: Bap\_0; Mad\_1

Delta, Dpp, En, Hh, Med, Pyr, Shn, Su\_H\_CSL, Ths, Ci, Htl, Mad, Nicd, Tin, Mef2

Delta, Dpp, Med, Pyr, Shn, Spi, Su\_H\_CSL, Ths, Upd, Wg, Doc, E\_Spl, Htl, Mad, Nicd, Pan, Slp, Stat92E, Tin(2), Twi, Zfh-1, Eve, Eya, Hbr, Mef2, Pnr

Delta, En, Hh, Med, Pyr, Su\_H\_CSL, Ths, Ci, Htl, Mad, Nicd, Tin, Brk, Mef2

Med, Pyr, Su\_H\_CSL, Ths, Wg, Da, Doc, Htl, Mad, Pan, Slp, Tin, Twi(2), Zfh-1, Brk, Eya, Hbr, Mef2, Pnr

### Genotype: Bap\_0; Mad\_0

Delta, Dpp, En, Hh, Med, Pyr, Shn, Su\_H\_CSL, Ths, Ci, Htl, Nicd, Brk, Srp(2)

Delta, Dpp, Med, Pyr, Shn, Spi, Su\_H\_CSL, Ths, Upd, Wg, E\_Spl, Htl, Nicd, Pan, Slp, Stat92E, Twi, Zfh-1, Brk, D-six4, Eya, Hbr, Poxm

Delta, En, Hh, Med, Pyr, Su\_H\_CSL, Ths, Ci, Htl, Nicd, Brk, Srp(2)

Med, Pyr, Su\_H\_CSL, Ths, Wg, Da, Htl, Pan, Slp, Twi(2), Zfh-1, Brk, D-six4, Eya, Hbr, Mef2, Poxm

### Genotype: Bap\_0; Mef2\_0

Delta, Dpp, En, Hh, Med, Pyr, Shn, Su\_H\_CSL, Ths, Ci, Htl, Mad, Nicd, Tin

Delta, Dpp, Med, Pyr, Shn, Spi, Su\_H\_CSL, Ths, Upd, Wg, Doc, E\_Spl, Htl, Mad, Nicd, Pan, Slp, Stat92E, Tin(2), Twi, Zfh-1, Eve, Eya, Hbr, Pnr

Delta, En, Hh, Med, Pyr, Su\_H\_CSL, Ths, Ci, Htl, Nicd, Brk, Srp(2)

Med, Pyr, Su\_H\_CSL, Ths, Wg, Da, Htl, Pan, Slp, Twi(2), Zfh-1, Brk, D-six4, Eya, Hbr, Poxm

### Genotype: Bap\_0; Mef2\_1

Delta, Dpp, En, Hh, Med, Pyr, Shn, Su\_H\_CSL, Ths, Ci, Htl, Mad, Nicd, Tin, Mef2

Delta, Dpp, Med, Pyr, Shn, Spi, Su\_H\_CSL, Ths, Upd, Wg, Doc, E\_Spl, Htl, Mad, Nicd, Pan, Slp, Stat92E, Tin(2), Twi, Zfh-1, Eve, Eya, Hbr, Mef2, Pnr

Delta, En, Hh, Med, Pyr, Su\_H\_CSL, Ths, Ci, Htl, Nicd, Brk, Mef2, Srp(2)

Med, Pyr, Su\_H\_CSL, Ths, Wg, Da, Htl, Pan, Slp, Twi(2), Zfh-1, Brk, D-six4, Eya, Hbr, Mef2, Poxm

### Genotype: Nicd\_1; Srp\_2

Delta, Dpp, En, Hh, Med, Pyr, Shn, Su\_H\_CSL, Ths, Bap(3), Bin, Ci, Htl, Mad, Nicd, Tin, Mef2, Srp(2)

Delta, Dpp, Med, Pyr, Shn, Spi, Su\_H\_CSL, Ths, Upd, Wg, Doc, E\_Spl, Htl, Mad, Nicd, Pan, Slp, Stat92E, Tin(2), Twi, Zfh-1, Eve, Eya, Hbr, Mef2, Pnr, Srp(2)

Delta, En, Hh, Med, Pyr, Su\_H\_CSL, Ths, Ci, Htl, Nicd, Brk, Srp(2)

Med, Pyr, Su\_H\_CSL, Ths, Wg, Htl, Nicd, Pan, Slp, Twi, Zfh-1, Brk, D-six4, Eya, Hbr, Poxm, Srp(2)

### Genotype: Nicd\_1; Srp\_0

Delta, Dpp, En, Hh, Med, Pyr, Shn, Su\_H\_CSL, Ths, Bap(3), Bin, Ci, Htl, Mad, Nicd, Tin, Mef2

Delta, Dpp, Med, Pyr, Shn, Spi, Su\_H\_CSL, Ths, Upd, Wg, Doc, E\_Spl, Htl, Mad, Nicd, Pan, Slp, Stat92E, Tin(2), Twi, Zfh-1, Eve, Eya, Hbr, Mef2, Pnr

Delta, En, Hh, Med, Pyr, Su\_H\_CSL, Ths, Ci, Htl, Nicd, Brk

Med, Pyr, Su\_H\_CSL, Ths, Wg, Htl, Nicd, Pan, Slp, Twi, Zfh-1, Brk, D-six4, Eya, Hbr, Poxm

### Genotype: Mef2\_0; Slp\_0

Delta, Dpp, En, Hh, Med, Pyr, Shn, Su\_H\_CSL, Ths, Bap(3), Bin, Ci, Htl, Mad, Nicd, Tin

Delta, Dpp, En, Med, Pyr, Shn, Spi, Su\_H\_CSL, Ths, Upd, Wg, Bap, Doc, E\_Spl, Htl, Mad, Nicd, Pan, Stat92E, Tin(2), Eya, Hbr, Pnr

Delta, En, Hh, Med, Pyr, Su\_H\_CSL, Ths, Ci, Htl, Nicd, Brk, Srp(2)

En, Med, Pyr, Su\_H\_CSL, Ths, Wg, Da, Htl, Pan, Brk, Hbr

En, Med, Pyr, Su\_H\_CSL, Ths, Wg, Da, Htl, Pan, Twi, Zfh-1, Brk, D-six4, Eya, Hbr, Poxm

### Genotype: Mef2\_0; Slp\_1

Delta, Dpp, Hh, Med, Pyr, Shn, Su\_H\_CSL, Ths, Bap, Ci, Htl, Mad, Nicd, Slp, Tin

Delta, Dpp, Hh, Med, Pyr, Shn, Su\_H\_CSL, Ths, Bap, Ci, Htl, Mad, Nicd, Slp, Tin, Twi, Zfh-1, Eya

Delta, Dpp, Med, Pyr, Shn, Spi, Su\_H\_CSL, Ths, Upd, Wg, Doc, E\_Spl, Htl, Mad, Nicd, Pan, Slp, Stat92E, Tin(2), Twi, Zfh-1, Eve, Eya, Hbr, Pnr

Delta, Hh, Med, Pyr, Su\_H\_CSL, Ths, Ci, Htl, Nicd, Slp, Brk, Srp

Delta, Hh, Med, Pyr, Su\_H\_CSL, Ths, Ci, Htl, Nicd, Slp, Twi, Zfh-1, Brk, D-six4, Eya, Srp

Med, Pyr, Su\_H\_CSL, Ths, Wg, Da, Htl, Pan, Slp, Twi(2), Zfh-1, Brk, D-six4, Eya, Hbr, Poxm

### Genotype: Mef2\_1; Pan\_1

Delta, Dpp, En, Hh, Med, Pyr, Shn, Su\_H\_CSL, Ths, Bap, Ci, Doc, Htl, Mad, Nicd, Pan, Slp, Tin, Hbr, Mef2, Pnr

Delta, Dpp, En, Hh, Med, Pyr, Shn, Su\_H\_CSL, Ths, Bap, Ci, Doc, Htl, Mad, Nicd, Pan, Slp, Tin, Twi, Zfh-1, Eya, Hbr, Mef2, Pnr

Delta, Dpp, Med, Pyr, Shn, Spi, Su\_H\_CSL, Ths, Upd, Wg, Doc, E\_Spl, Htl, Mad, Nicd, Pan, Slp, Stat92E, Tin(2), Twi, Zfh-1, Eve, Eya, Hbr, Mef2, Pnr

Delta, En, Hh, Med, Pyr, Su\_H\_CSL, Ths, Ci, Htl, Nicd, Pan, Slp, Brk, Hbr, Mef2, Srp(2)

Delta, En, Hh, Med, Pyr, Su\_H\_CSL, Ths, Ci, Htl, Nicd, Pan, Slp, Twi, Zfh-1, Brk, D-six4, Eya, Hbr, Mef2, Poxm, Srp(2)

Med, Pyr, Su\_H\_CSL, Ths, Wg, Da, Htl, Pan, Slp, Twi(2), Zfh-1, Brk, D-six4, Eya, Hbr, Mef2, Poxm

### Genotype: Mef2\_1; Pan\_0

Delta, Dpp, En, Hh, Med, Pyr, Shn, Su\_H\_CSL, Ths, Bap(3), Bin, Ci, Htl, Mad, Nicd, Tin, Mef2

Delta, Dpp, Med, Pyr, Shn, Spi, Su\_H\_CSL, Ths, Upd, Wg, Bap, E\_Spl, Htl, Mad, Nicd, Stat92E, Tin, Mef2

Delta, En, Hh, Med, Pyr, Su\_H\_CSL, Ths, Ci, Htl, Nicd, Brk, Mef2, Srp(2)

Med, Pyr, Su\_H\_CSL, Ths, Wg, Da, Htl, Brk, Mef2

Med, Pyr, Su\_H\_CSL, Ths, Wg, Da, Htl, Twi, Zfh-1, Brk, D-six4, Eya, Mef2

### Genotype: Nicd\_0; Pan\_1

Delta, Dpp, En, Hh, Med, Pyr, Shn, Su\_H\_CSL, Ths, Bap, Ci, Da, Doc, Htl, Mad, Pan, Slp, Tin, Hbr, Mef2, Pnr

Delta, Dpp, En, Hh, Med, Pyr, Shn, Su\_H\_CSL, Ths, Bap, Ci, Da, Doc, Htl, Mad, Pan, Slp, Tin, Twi(2), Zfh-1, Eya, Hbr, Mef2, Pnr

Delta, Dpp, Med, Pyr, Shn, Spi, Su\_H\_CSL, Ths, Upd, Wg, Da, Doc, Htl, Mad, Pan, Slp, Stat92E, Tin(2), Twi(2), Zfh-1, Eve, Eya, Hbr, Mef2, Pnr

Delta, En, Hh, Med, Pyr, Su\_H\_CSL, Ths, Ci, Da, Htl, Pan, Slp, Brk, Hbr, Srp(2)

Delta, En, Hh, Med, Pyr, Su\_H\_CSL, Ths, Ci, Da, Htl, Pan, Slp, Twi(2), Zfh-1, Brk, D-six4, Eya, Hbr, Mef2, Poxm, Srp(2)

Med, Pyr, Su\_H\_CSL, Ths, Wg, Da, Htl, Pan, Slp, Twi(2), Zfh-1, Brk, D-six4, Eya, Hbr, Mef2, Poxm

### Genotype: Pan\_0; Twi\_2

Delta, Dpp, En, Hh, Med, Pyr, Shn, Su\_H\_CSL, Ths, Bap(3), Bin, Ci, Htl, Mad, Nicd, Tin, Twi(2), Zfh-1, Eya, Mef2

Delta, Dpp, Med, Pyr, Shn, Spi, Su\_H\_CSL, Ths, Upd, Wg, Bap, E\_Spl, Htl, Mad, Nicd, Stat92E, Tin, Twi(2), Zfh-1, Eya, Mef2

Delta, En, Hh, Med, Pyr, Su\_H\_CSL, Ths, Ci, Htl, Nicd, Twi(2), Zfh-1, Brk, D-six4, Eya, Mef2, Srp(2)

Med, Pyr, Su\_H\_CSL, Ths, Wg, Da, Htl, Twi(2), Zfh-1, Brk, D-six4, Eya, Mef2

### Genotype: Pan\_0; Twi\_0

Delta, Dpp, En, Hh, Med, Pyr, Shn, Su\_H\_CSL, Ths, Bap(3), Bin, Ci, Htl, Mad, Nicd, Tin, Mef2

Delta, Dpp, Med, Pyr, Shn, Spi, Su\_H\_CSL, Ths, Upd, Wg, Bap, E\_Spl, Htl, Mad, Nicd, Stat92E, Tin, Mef2

Delta, En, Hh, Med, Pyr, Su\_H\_CSL, Ths, Ci, Htl, Nicd, Brk, Srp(2)

Med, Pyr, Su\_H\_CSL, Ths, Wg, Da, Htl, Brk

### Genotype: Med\_0; Mef2\_1

Delta, Dpp, En, Hh, Pyr, Shn, Su\_H\_CSL, Ths, Bap(2), Bin, Ci, Htl, Mad, Nicd, Tin, Brk, D-six4, Mef2, Srp(2)

Delta, Dpp, Pyr, Shn, Spi, Su\_H\_CSL, Ths, Upd, Wg, E\_Spl, Htl, Mad, Nicd, Pan, Slp, Stat92E, Tin, Twi, Zfh-1, Brk, D-six4, Eya, Hbr, Mef2, Poxm

Delta, En, Hh, Pyr, Su\_H\_CSL, Ths, Ci, Htl, Nicd, Brk, Mef2, Srp(2)

Pyr, Su\_H\_CSL, Ths, Wg, Da, Htl, Pan, Slp, Twi(2), Zfh-1, Brk, D-six4, Eya, Hbr, Mef2, Poxm

### Genotype: Med\_0; Mef2\_0

Delta, Dpp, En, Hh, Pyr, Shn, Su\_H\_CSL, Ths, Bap(2), Bin, Ci, Htl, Mad, Nicd, Tin, Brk, D-six4, Srp(2)

Delta, Dpp, Pyr, Shn, Spi, Su\_H\_CSL, Ths, Upd, Wg, E\_Spl, Htl, Mad, Nicd, Pan, Slp, Stat92E, Tin, Twi, Zfh-1, Brk, D-six4, Eya, Hbr, Poxm

Delta, En, Hh, Pyr, Su\_H\_CSL, Ths, Ci, Htl, Nicd, Brk, Srp(2)

Pyr, Su\_H\_CSL, Ths, Wg, Da, Htl, Pan, Slp, Twi(2), Zfh-1, Brk, D-six4, Eya, Hbr, Poxm

### Genotype: Mef2\_1; Slp\_1

Delta, Dpp, Hh, Med, Pyr, Shn, Su\_H\_CSL, Ths, Bap, Ci, Htl, Mad, Nicd, Slp, Tin, Mef2

Delta, Dpp, Hh, Med, Pyr, Shn, Su\_H\_CSL, Ths, Bap, Ci, Htl, Mad, Nicd, Slp, Tin, Twi, Zfh-1, Eya, Mef2

Delta, Dpp, Med, Pyr, Shn, Spi, Su\_H\_CSL, Ths, Upd, Wg, Doc, E\_Spl, Htl, Mad, Nicd, Pan, Slp, Stat92E, Tin(2), Twi, Zfh-1, Eve, Eya, Hbr, Mef2, Pnr

Delta, Hh, Med, Pyr, Su\_H\_CSL, Ths, Ci, Htl, Nicd, Slp, Brk, Mef2, Srp

Delta, Hh, Med, Pyr, Su\_H\_CSL, Ths, Ci, Htl, Nicd, Slp, Twi, Zfh-1, Brk, D-six4, Eya, Mef2, Srp

Med, Pyr, Su\_H\_CSL, Ths, Wg, Da, Htl, Pan, Slp, Twi(2), Zfh-1, Brk, D-six4, Eya, Hbr, Mef2, Poxm

### Genotype: Mef2\_1; Slp\_0

Delta, Dpp, En, Hh, Med, Pyr, Shn, Su\_H\_CSL, Ths, Bap(3), Bin, Ci, Htl, Mad, Nicd, Tin, Mef2

Delta, Dpp, En, Med, Pyr, Shn, Spi, Su\_H\_CSL, Ths, Upd, Wg, Bap, Doc, E\_Spl, Htl, Mad, Nicd, Pan, Stat92E, Tin(2), Eya, Hbr, Mef2, Pnr

Delta, En, Hh, Med, Pyr, Su\_H\_CSL, Ths, Ci, Htl, Nicd, Brk, Mef2, Srp(2)

En, Med, Pyr, Su\_H\_CSL, Ths, Wg, Da, Htl, Pan, Brk, Hbr, Mef2

En, Med, Pyr, Su\_H\_CSL, Ths, Wg, Da, Htl, Pan, Twi, Zfh-1, Brk, D-six4, Eya, Hbr, Mef2, Poxm

### Genotype: Mad\_1; Pnr\_1

Delta, Dpp, En, Hh, Med, Pyr, Shn, Su\_H\_CSL, Ths, Bap(3), Bin, Ci, Htl, Mad, Nicd, Tin, Mef2, Pnr

Delta, Dpp, Med, Pyr, Shn, Spi, Su\_H\_CSL, Ths, Upd, Wg, Doc, E\_Spl, Htl, Mad, Nicd, Pan, Slp, Stat92E, Tin(2), Twi, Zfh-1, Eve, Eya, Hbr, Mef2, Pnr

Delta, En, Hh, Med, Pyr, Su\_H\_CSL, Ths, Bap(3), Bin, Ci, Htl, Mad, Nicd, Tin, Brk, Mef2, Pnr

Med, Pyr, Su\_H\_CSL, Ths, Wg, Da, Doc, Htl, Mad, Pan, Slp, Tin, Twi(2), Zfh-1, Brk, Eya, Hbr, Mef2, Pnr

### Genotype: Mad\_1; Pnr\_0

Delta, Dpp, En, Hh, Med, Pyr, Shn, Su\_H\_CSL, Ths, Bap(3), Bin, Ci, Htl, Mad, Nicd, Tin, Mef2

Delta, Dpp, Med, Pyr, Shn, Spi, Su\_H\_CSL, Ths, Upd, Wg, Doc, E\_Spl, Htl, Mad, Nicd, Pan, Slp, Stat92E, Tin(2), Twi, Zfh-1, Eve, Eya, Hbr, Mef2

Delta, En, Hh, Med, Pyr, Su\_H\_CSL, Ths, Bap(3), Bin, Ci, Htl, Mad, Nicd, Tin, Brk, Mef2

Med, Pyr, Su\_H\_CSL, Ths, Wg, Da, Doc, Htl, Mad, Pan, Slp, Tin, Twi(2), Zfh-1, Brk, Eya, Hbr, Mef2

### Genotype: Ci\_1; Srp\_0

Delta, Dpp, En, Hh, Med, Pyr, Shn, Su\_H\_CSL, Ths, Bap(3), Bin, Ci, Htl, Mad, Nicd, Tin, Mef2

Delta, Dpp, Med, Pyr, Shn, Spi, Su\_H\_CSL, Ths, Upd, Wg, Bap, Ci, Doc, E\_Spl, Htl, Mad, Nicd, Pan, Slp, Stat92E, Tin(2), Twi, Zfh-1, Eve, Eya, Hbr, Mef2, Pnr

Delta, En, Hh, Med, Pyr, Su\_H\_CSL, Ths, Ci, Htl, Nicd, Brk

Med, Pyr, Su\_H\_CSL, Ths, Wg, Ci, Da, Htl, Pan, Slp, Twi(2), Zfh-1, Brk, D-six4, Eya, Hbr, Mef2, Poxm

### Genotype: Bin\_0; Mad\_1

Delta, Dpp, En, Hh, Med, Pyr, Shn, Su\_H\_CSL, Ths, Bap(3), Ci, Htl, Mad, Nicd, Tin, Mef2

Delta, Dpp, Med, Pyr, Shn, Spi, Su\_H\_CSL, Ths, Upd, Wg, Doc, E\_Spl, Htl, Mad, Nicd, Pan, Slp, Stat92E, Tin(2), Twi, Zfh-1, Eve, Eya, Hbr, Mef2, Pnr

Delta, En, Hh, Med, Pyr, Su\_H\_CSL, Ths, Bap(3), Ci, Htl, Mad, Nicd, Tin, Brk, Mef2

Med, Pyr, Su\_H\_CSL, Ths, Wg, Da, Doc, Htl, Mad, Pan, Slp, Tin, Twi(2), Zfh-1, Brk, Eya, Hbr, Mef2, Pnr

### Genotype: Bin\_0; Mad\_0

Delta, Dpp, En, Hh, Med, Pyr, Shn, Su\_H\_CSL, Ths, Ci, Htl, Nicd, Brk, Srp(2)

Delta, Dpp, Med, Pyr, Shn, Spi, Su\_H\_CSL, Ths, Upd, Wg, E\_Spl, Htl, Nicd, Pan, Slp, Stat92E, Twi, Zfh-1, Brk, D-six4, Eya, Hbr, Poxm

Delta, En, Hh, Med, Pyr, Su\_H\_CSL, Ths, Ci, Htl, Nicd, Brk, Srp(2)

Med, Pyr, Su\_H\_CSL, Ths, Wg, Da, Htl, Pan, Slp, Twi(2), Zfh-1, Brk, D-six4, Eya, Hbr, Mef2, Poxm

### Genotype: Doc\_1; Pnr\_1

Delta, Dpp, En, Hh, Med, Pyr, Shn, Su\_H\_CSL, Ths, Bap(3), Bin, Ci, Doc, Htl, Mad, Nicd, Tin, Mef2, Pnr

Delta, Dpp, Med, Pyr, Shn, Spi, Su\_H\_CSL, Ths, Upd, Wg, Doc, E\_Spl, Htl, Mad, Nicd, Pan, Slp, Stat92E, Tin(2), Twi, Zfh-1, Eve, Eya, Hbr, Mef2, Pnr

Delta, En, Hh, Med, Pyr, Su\_H\_CSL, Ths, Ci, Doc, Htl, Nicd, Brk, Pnr, Srp(2)

Med, Pyr, Su\_H\_CSL, Ths, Wg, Da, Doc, Htl, Pan, Slp, Twi(2), Zfh-1, Brk, D-six4, Eya, Hbr, Mef2, Pnr, Poxm

### Genotype: Doc\_1; Pnr\_0

Delta, Dpp, En, Hh, Med, Pyr, Shn, Su\_H\_CSL, Ths, Bap(3), Bin, Ci, Doc, Htl, Mad, Nicd, Tin, Mef2

Delta, Dpp, Med, Pyr, Shn, Spi, Su\_H\_CSL, Ths, Upd, Wg, Doc, E\_Spl, Htl, Mad, Nicd, Pan, Slp, Stat92E, Tin(2), Twi, Zfh-1, Eve, Eya, Hbr, Mef2

Delta, En, Hh, Med, Pyr, Su\_H\_CSL, Ths, Ci, Doc, Htl, Nicd, Brk, Srp(2)

Med, Pyr, Su\_H\_CSL, Ths, Wg, Da, Doc, Htl, Pan, Slp, Twi(2), Zfh-1, Brk, D-six4, Eya, Hbr, Mef2, Poxm

### Genotype: Nicd\_1; Twi\_2

Delta, Dpp, En, Hh, Med, Pyr, Shn, Su\_H\_CSL, Ths, Bap(3), Bin, Ci, Htl, Mad, Nicd, Tin, Twi(2), Zfh-1, Eya, Mef2

Delta, Dpp, Med, Pyr, Shn, Spi, Su\_H\_CSL, Ths, Upd, Wg, Doc, E\_Spl, Htl, Mad, Nicd, Pan, Slp, Stat92E, Tin(2), Twi(2), Zfh-1, Eve, Eya, Hbr, Mef2, Pnr

Delta, En, Hh, Med, Pyr, Su\_H\_CSL, Ths, Ci, Htl, Nicd, Twi(2), Zfh-1, Brk, D-six4, Eya, Mef2, Srp(2)

Med, Pyr, Su\_H\_CSL, Ths, Wg, Htl, Nicd, Pan, Slp, Twi(2), Zfh-1, Brk, D-six4, Eya, Hbr, Mef2, Poxm

### Genotype: Doc\_0; Pnr\_1

Delta, Dpp, En, Hh, Med, Pyr, Shn, Su\_H\_CSL, Ths, Bap(3), Bin, Ci, Htl, Mad, Nicd, Tin, Mef2, Pnr

Delta, Dpp, Med, Pyr, Shn, Spi, Su\_H\_CSL, Ths, Upd, Wg, E\_Spl, Htl, Mad, Nicd, Pan, Slp, Stat92E, Tin(2), Twi, Zfh-1, Eve, Eya, Hbr, Mef2, Pnr

Delta, En, Hh, Med, Pyr, Su\_H\_CSL, Ths, Ci, Htl, Nicd, Brk, Pnr, Srp(2)

Med, Pyr, Su\_H\_CSL, Ths, Wg, Da, Htl, Pan, Slp, Twi(2), Zfh-1, Brk, D-six4, Eya, Hbr, Mef2, Pnr, Poxm

### Genotype: Nicd\_1; Twi\_0

Delta, Dpp, En, Hh, Med, Pyr, Shn, Su\_H\_CSL, Ths, Bap(3), Bin, Ci, Htl, Mad, Nicd, Tin, Mef2

Delta, Dpp, Med, Pyr, Shn, Spi, Su\_H\_CSL, Ths, Upd, Wg, Doc, E\_Spl, Htl, Mad, Nicd, Pan, Slp, Stat92E, Tin(2), Eya, Hbr, Mef2, Pnr

Delta, En, Hh, Med, Pyr, Su\_H\_CSL, Ths, Ci, Htl, Nicd, Brk, Srp(2)

Med, Pyr, Su\_H\_CSL, Ths, Wg, Htl, Nicd, Pan, Slp, Brk, Hbr

### Genotype: Srp\_2

Delta, Dpp, En, Hh, Med, Pyr, Shn, Su\_H\_CSL, Ths, Bap(3), Bin, Ci, Htl, Mad, Nicd, Tin, Mef2, Srp(2)

Delta, Dpp, Med, Pyr, Shn, Spi, Su\_H\_CSL, Ths, Upd, Wg, Doc, E\_Spl, Htl, Mad, Nicd, Pan, Slp, Stat92E, Tin(2), Twi, Zfh-1, Eve, Eya, Hbr, Mef2, Pnr, Srp(2)

Delta, En, Hh, Med, Pyr, Su\_H\_CSL, Ths, Ci, Htl, Nicd, Brk, Srp(2)

Med, Pyr, Su\_H\_CSL, Ths, Wg, Da, Htl, Pan, Slp, Twi(2), Zfh-1, Brk, D-six4, Eya, Hbr, Mef2, Poxm, Srp(2)

### Genotype: Pnr\_1; Tin\_2

Delta, Dpp, En, Hh, Med, Pyr, Shn, Su\_H\_CSL, Ths, Bap(3), Bin, Ci, E\_Spl, Htl, Mad, Nicd, Stat92E, Tin(2), Eya, Mef2, Pnr

Delta, Dpp, Med, Pyr, Shn, Spi, Su\_H\_CSL, Ths, Upd, Wg, Doc, E\_Spl, Htl, Mad, Nicd, Pan, Slp, Stat92E, Tin(2), Twi, Zfh-1, Eve, Eya, Hbr, Mef2, Pnr

Delta, En, Hh, Med, Pyr, Su\_H\_CSL, Ths, Bap(2), Bin, Ci, E\_Spl, Htl, Nicd, Stat92E, Tin(2), Brk, D-six4, Eya, Mef2, Pnr, Srp(2)

Med, Pyr, Su\_H\_CSL, Ths, Wg, Da, Htl, Pan, Slp, Stat92E, Tin(2), Twi(2), Zfh-1, Brk, D-six4, Eya, Hbr, Mef2, Pnr, Poxm

### Genotype: Bap\_3; Slp\_1

Delta, Dpp, Hh, Med, Pyr, Shn, Su\_H\_CSL, Ths, Bap(3), Bin, Ci, Htl, Mad, Nicd, Slp, Tin, Mef2

Delta, Dpp, Hh, Med, Pyr, Shn, Su\_H\_CSL, Ths, Bap(3), Bin, Ci, Htl, Mad, Nicd, Slp, Tin, Twi, Zfh-1, Eya, Mef2

Delta, Dpp, Med, Pyr, Shn, Spi, Su\_H\_CSL, Ths, Upd, Wg, Bap(3), Bin, Doc, E\_Spl, Htl, Mad, Nicd, Pan, Slp, Stat92E, Tin(2), Twi, Zfh-1, Eve, Eya, Hbr, Mef2, Pnr

Delta, Hh, Med, Pyr, Su\_H\_CSL, Ths, Bap(3), Bin, Ci, Htl, Nicd, Slp, Brk, Srp

Delta, Hh, Med, Pyr, Su\_H\_CSL, Ths, Bap(3), Bin, Ci, Htl, Nicd, Slp, Twi, Zfh-1, Brk, D-six4, Eya, Srp

Med, Pyr, Su\_H\_CSL, Ths, Wg, Bap(3), Bin, Da, Htl, Pan, Slp, Twi(2), Zfh-1, Brk, D-six4, Eya, Hbr, Mef2, Poxm

### Genotype: Bap\_3; Slp\_0

Delta, Dpp, En, Hh, Med, Pyr, Shn, Su\_H\_CSL, Ths, Bap(3), Bin, Ci, Htl, Mad, Nicd, Tin, Mef2

Delta, Dpp, En, Med, Pyr, Shn, Spi, Su\_H\_CSL, Ths, Upd, Wg, Bap(3), Bin, Doc, E\_Spl, Htl, Mad, Nicd, Pan, Stat92E, Tin(2), Eya, Hbr, Mef2, Pnr

Delta, En, Hh, Med, Pyr, Su\_H\_CSL, Ths, Bap(3), Bin, Ci, Htl, Nicd, Brk, Srp(2)

En, Med, Pyr, Su\_H\_CSL, Ths, Wg, Bap(3), Bin, Da, Htl, Pan, Brk, Hbr

En, Med, Pyr, Su\_H\_CSL, Ths, Wg, Bap(3), Bin, Da, Htl, Pan, Twi, Zfh-1, Brk, D-six4, Eya, Hbr, Poxm

### Genotype: Med\_0; Twi\_0

Delta, Dpp, En, Hh, Pyr, Shn, Su\_H\_CSL, Ths, Bap(2), Bin, Ci, Htl, Mad, Nicd, Tin, Brk, D-six4, Mef2, Srp(2)

Delta, Dpp, Pyr, Shn, Spi, Su\_H\_CSL, Ths, Upd, Wg, E\_Spl, Htl, Mad, Nicd, Pan, Slp, Stat92E, Tin, Brk, D-six4, Hbr, Mef2

Delta, En, Hh, Pyr, Su\_H\_CSL, Ths, Ci, Htl, Nicd, Brk, Srp(2)

Pyr, Su\_H\_CSL, Ths, Wg, Da, Htl, Pan, Slp, Brk, Hbr

### Genotype: Ci\_1; Nicd\_1

Delta, Dpp, En, Hh, Med, Pyr, Shn, Su\_H\_CSL, Ths, Bap(3), Bin, Ci, Htl, Mad, Nicd, Tin, Mef2

Delta, Dpp, Med, Pyr, Shn, Spi, Su\_H\_CSL, Ths, Upd, Wg, Bap, Ci, Doc, E\_Spl, Htl, Mad, Nicd, Pan, Slp, Stat92E, Tin(2), Twi, Zfh-1, Eve, Eya, Hbr, Mef2, Pnr

Delta, En, Hh, Med, Pyr, Su\_H\_CSL, Ths, Ci, Htl, Nicd, Brk, Srp(2)

Med, Pyr, Su\_H\_CSL, Ths, Wg, Ci, Htl, Nicd, Pan, Slp, Twi, Zfh-1, Brk, D-six4, Eya, Hbr, Poxm, Srp

### Genotype: Ci\_1; Nicd\_0

Delta, Dpp, En, Hh, Med, Pyr, Shn, Su\_H\_CSL, Ths, Bap(3), Bin, Ci, Da, Htl, Mad, Tin, Mef2

Delta, Dpp, En, Hh, Med, Pyr, Shn, Su\_H\_CSL, Ths, Bap(3), Bin, Ci, Da, Htl, Mad, Tin, Twi, Zfh-1, Eya, Mef2

Delta, Dpp, Med, Pyr, Shn, Spi, Su\_H\_CSL, Ths, Upd, Wg, Bap, Ci, Da, Doc, Htl, Mad, Pan, Slp, Stat92E, Tin(2), Twi(2), Zfh-1, Eve, Eya, Hbr, Mef2, Pnr

Delta, En, Hh, Med, Pyr, Su\_H\_CSL, Ths, Ci, Da, Htl, Brk, Srp(2)

Delta, En, Hh, Med, Pyr, Su\_H\_CSL, Ths, Ci, Da, Htl, Twi, Zfh-1, Brk, D-six4, Eya, Srp(2)

Med, Pyr, Su\_H\_CSL, Ths, Wg, Ci, Da, Htl, Pan, Slp, Twi(2), Zfh-1, Brk, D-six4, Eya, Hbr, Mef2, Poxm, Srp

### Genotype: Slp\_0; Srp\_0

Delta, Dpp, En, Hh, Med, Pyr, Shn, Su\_H\_CSL, Ths, Bap(3), Bin, Ci, Htl, Mad, Nicd, Tin, Mef2

Delta, Dpp, En, Med, Pyr, Shn, Spi, Su\_H\_CSL, Ths, Upd, Wg, Bap, Doc, E\_Spl, Htl, Mad, Nicd, Pan, Stat92E, Tin(2), Eya, Hbr, Mef2, Pnr

Delta, En, Hh, Med, Pyr, Su\_H\_CSL, Ths, Ci, Htl, Nicd, Brk

En, Med, Pyr, Su\_H\_CSL, Ths, Wg, Da, Htl, Pan, Brk, Hbr

En, Med, Pyr, Su\_H\_CSL, Ths, Wg, Da, Htl, Pan, Twi, Zfh-1, Brk, D-six4, Eya, Hbr, Poxm

### Genotype: Slp\_0; Srp\_2

Delta, Dpp, En, Hh, Med, Pyr, Shn, Su\_H\_CSL, Ths, Bap(3), Bin, Ci, Htl, Mad, Nicd, Tin, Mef2, Srp(2)

Delta, Dpp, En, Med, Pyr, Shn, Spi, Su\_H\_CSL, Ths, Upd, Wg, Bap, Doc, E\_Spl, Htl, Mad, Nicd, Pan, Stat92E, Tin(2), Eya, Hbr, Mef2, Pnr, Srp(2)

Delta, En, Hh, Med, Pyr, Su\_H\_CSL, Ths, Ci, Htl, Nicd, Brk, Srp(2)

En, Med, Pyr, Su\_H\_CSL, Ths, Wg, Da, Htl, Pan, Brk, Hbr, Srp(2)

En, Med, Pyr, Su\_H\_CSL, Ths, Wg, Da, Htl, Pan, Twi, Zfh-1, Brk, D-six4, Eya, Hbr, Poxm, Srp(2)

### Genotype: Ci\_1

Delta, Dpp, En, Hh, Med, Pyr, Shn, Su\_H\_CSL, Ths, Bap(3), Bin, Ci, Htl, Mad, Nicd, Tin, Mef2

Delta, Dpp, Med, Pyr, Shn, Spi, Su\_H\_CSL, Ths, Upd, Wg, Bap, Ci, Doc, E\_Spl, Htl, Mad, Nicd, Pan, Slp, Stat92E, Tin(2), Twi, Zfh-1, Eve, Eya, Hbr, Mef2, Pnr

Delta, En, Hh, Med, Pyr, Su\_H\_CSL, Ths, Ci, Htl, Nicd, Brk, Srp(2)

Med, Pyr, Su\_H\_CSL, Ths, Wg, Ci, Da, Htl, Pan, Slp, Twi(2), Zfh-1, Brk, D-six4, Eya, Hbr, Mef2, Poxm, Srp

### Genotype: Doc\_1; Pan\_0

Delta, Dpp, En, Hh, Med, Pyr, Shn, Su\_H\_CSL, Ths, Bap(3), Bin, Ci, Doc, Htl, Mad, Nicd, Tin, Mef2, Pnr

Delta, Dpp, Med, Pyr, Shn, Spi, Su\_H\_CSL, Ths, Upd, Wg, Bap, Doc, E\_Spl, Htl, Mad, Nicd, Stat92E, Tin, Mef2, Pnr

Delta, En, Hh, Med, Pyr, Su\_H\_CSL, Ths, Ci, Doc, Htl, Nicd, Brk, Srp(2)

Med, Pyr, Su\_H\_CSL, Ths, Wg, Da, Doc, Htl, Brk

Med, Pyr, Su\_H\_CSL, Ths, Wg, Da, Doc, Htl, Twi, Zfh-1, Brk, D-six4, Eya

### Genotype: Doc\_1; Pan\_1

Delta, Dpp, En, Hh, Med, Pyr, Shn, Su\_H\_CSL, Ths, Bap, Ci, Doc, Htl, Mad, Nicd, Pan, Slp, Tin, Hbr, Mef2, Pnr

Delta, Dpp, En, Hh, Med, Pyr, Shn, Su\_H\_CSL, Ths, Bap, Ci, Doc, Htl, Mad, Nicd, Pan, Slp, Tin, Twi, Zfh-1, Eya, Hbr, Mef2, Pnr

Delta, Dpp, Med, Pyr, Shn, Spi, Su\_H\_CSL, Ths, Upd, Wg, Doc, E\_Spl, Htl, Mad, Nicd, Pan, Slp, Stat92E, Tin(2), Twi, Zfh-1, Eve, Eya, Hbr, Mef2, Pnr

Delta, En, Hh, Med, Pyr, Su\_H\_CSL, Ths, Ci, Doc, Htl, Nicd, Pan, Slp, Brk, Hbr, Srp(2)

Delta, En, Hh, Med, Pyr, Su\_H\_CSL, Ths, Ci, Doc, Htl, Nicd, Pan, Slp, Twi, Zfh-1, Brk, D-six4, Eya, Hbr, Poxm, Srp(2)

Med, Pyr, Su\_H\_CSL, Ths, Wg, Da, Doc, Htl, Pan, Slp, Twi(2), Zfh-1, Brk, D-six4, Eya, Hbr, Mef2, Poxm

### Genotype: Srp\_0; Tin\_2

Delta, Dpp, En, Hh, Med, Pyr, Shn, Su\_H\_CSL, Ths, Bap(3), Bin, Ci, E\_Spl, Htl, Mad, Nicd, Stat92E, Tin(2), Eya, Mef2

Delta, Dpp, Med, Pyr, Shn, Spi, Su\_H\_CSL, Ths, Upd, Wg, Doc, E\_Spl, Htl, Mad, Nicd, Pan, Slp, Stat92E, Tin(2), Twi, Zfh-1, Eve, Eya, Hbr, Mef2, Pnr

Delta, En, Hh, Med, Pyr, Su\_H\_CSL, Ths, Bap(2), Bin, Ci, E\_Spl, Htl, Nicd, Stat92E, Tin(2), Brk, D-six4, Eya, Mef2

Med, Pyr, Su\_H\_CSL, Ths, Wg, Da, Htl, Pan, Slp, Stat92E, Tin(2), Twi(2), Zfh-1, Brk, D-six4, Eya, Hbr, Mef2, Poxm

### Genotype: Bap\_0; Tin\_2

Delta, Dpp, En, Hh, Med, Pyr, Shn, Su\_H\_CSL, Ths, Ci, E\_Spl, Htl, Mad, Nicd, Stat92E, Tin(2), Eya, Mef2

Delta, Dpp, Med, Pyr, Shn, Spi, Su\_H\_CSL, Ths, Upd, Wg, Doc, E\_Spl, Htl, Mad, Nicd, Pan, Slp, Stat92E, Tin(2), Twi, Zfh-1, Eve, Eya, Hbr, Mef2, Pnr

Delta, En, Hh, Med, Pyr, Su\_H\_CSL, Ths, Ci, E\_Spl, Htl, Nicd, Stat92E, Tin(2), Brk, D-six4, Eya, Mef2, Srp(2)

Med, Pyr, Su\_H\_CSL, Ths, Wg, Da, Htl, Pan, Slp, Stat92E, Tin(2), Twi(2), Zfh-1, Brk, D-six4, Eya, Hbr, Mef2, Poxm

### Genotype: Bap\_0; Tin\_0

Delta, Dpp, En, Hh, Med, Pyr, Shn, Su\_H\_CSL, Ths, Ci, Htl, Mad, Nicd

Delta, Dpp, Med, Pyr, Shn, Spi, Su\_H\_CSL, Ths, Upd, Wg, Doc, E\_Spl, Htl, Mad, Nicd, Pan, Slp, Stat92E, Twi, Zfh-1, Eya, Hbr

Delta, En, Hh, Med, Pyr, Su\_H\_CSL, Ths, Ci, Htl, Nicd, Brk, Srp(2)

Med, Pyr, Su\_H\_CSL, Ths, Wg, Da, Htl, Pan, Slp, Twi(2), Zfh-1, Brk, D-six4, Eya, Hbr, Mef2, Poxm

### Genotype: Bin\_0; Slp\_0

Delta, Dpp, En, Hh, Med, Pyr, Shn, Su\_H\_CSL, Ths, Bap(3), Ci, Htl, Mad, Nicd, Tin, Mef2

Delta, Dpp, En, Med, Pyr, Shn, Spi, Su\_H\_CSL, Ths, Upd, Wg, Bap, Doc, E\_Spl, Htl, Mad, Nicd, Pan, Stat92E, Tin(2), Eya, Hbr, Mef2, Pnr

Delta, En, Hh, Med, Pyr, Su\_H\_CSL, Ths, Ci, Htl, Nicd, Brk, Srp(2)

En, Med, Pyr, Su\_H\_CSL, Ths, Wg, Da, Htl, Pan, Brk, Hbr

En, Med, Pyr, Su\_H\_CSL, Ths, Wg, Da, Htl, Pan, Twi, Zfh-1, Brk, D-six4, Eya, Hbr, Poxm

### Genotype: Bin\_0; Slp\_1

Delta, Dpp, Hh, Med, Pyr, Shn, Su\_H\_CSL, Ths, Bap, Ci, Htl, Mad, Nicd, Slp, Tin, Mef2

Delta, Dpp, Hh, Med, Pyr, Shn, Su\_H\_CSL, Ths, Bap, Ci, Htl, Mad, Nicd, Slp, Tin, Twi, Zfh-1, Eya, Mef2

Delta, Dpp, Med, Pyr, Shn, Spi, Su\_H\_CSL, Ths, Upd, Wg, Doc, E\_Spl, Htl, Mad, Nicd, Pan, Slp, Stat92E, Tin(2), Twi, Zfh-1, Eve, Eya, Hbr, Mef2, Pnr

Delta, Hh, Med, Pyr, Su\_H\_CSL, Ths, Ci, Htl, Nicd, Slp, Brk, Srp

Delta, Hh, Med, Pyr, Su\_H\_CSL, Ths, Ci, Htl, Nicd, Slp, Twi, Zfh-1, Brk, D-six4, Eya, Srp

Med, Pyr, Su\_H\_CSL, Ths, Wg, Da, Htl, Pan, Slp, Twi(2), Zfh-1, Brk, D-six4, Eya, Hbr, Mef2, Poxm

### Genotype: Ci\_0; Srp\_2

Delta, Dpp, En, Hh, Med, Pyr, Shn, Su\_H\_CSL, Ths, Bap, Htl, Mad, Nicd, Tin, Mef2, Srp(2)

Delta, Dpp, En, Hh, Med, Pyr, Shn, Su\_H\_CSL, Ths, Bap(2), Bin, Htl, Mad, Nicd, Tin, Mef2, Srp(2)

Delta, Dpp, Med, Pyr, Shn, Spi, Su\_H\_CSL, Ths, Upd, Wg, Doc, E\_Spl, Htl, Mad, Nicd, Pan, Slp, Stat92E, Tin(2), Twi, Zfh-1, Eve, Eya, Hbr, Mef2, Pnr, Srp(2)

Delta, En, Hh, Med, Pyr, Su\_H\_CSL, Ths, Htl, Nicd, Brk, Srp(2)

Med, Pyr, Su\_H\_CSL, Ths, Wg, Da, Htl, Pan, Slp, Twi(2), Zfh-1, Brk, D-six4, Eya, Hbr, Mef2, Poxm, Srp(2)

### Genotype: Doc\_1; Srp\_2

Delta, Dpp, En, Hh, Med, Pyr, Shn, Su\_H\_CSL, Ths, Bap(3), Bin, Ci, Doc, Htl, Mad, Nicd, Tin, Mef2, Pnr, Srp(2)

Delta, Dpp, Med, Pyr, Shn, Spi, Su\_H\_CSL, Ths, Upd, Wg, Doc, E\_Spl, Htl, Mad, Nicd, Pan, Slp, Stat92E, Tin(2), Twi, Zfh-1, Eve, Eya, Hbr, Mef2, Pnr, Srp(2)

Delta, En, Hh, Med, Pyr, Su\_H\_CSL, Ths, Ci, Doc, Htl, Nicd, Brk, Srp(2)

Med, Pyr, Su\_H\_CSL, Ths, Wg, Da, Doc, Htl, Pan, Slp, Twi(2), Zfh-1, Brk, D-six4, Eya, Hbr, Mef2, Poxm, Srp(2)

### Genotype: Ci\_0; Tin\_2

Delta, Dpp, En, Hh, Med, Pyr, Shn, Su\_H\_CSL, Ths, Bap, E\_Spl, Htl, Mad, Nicd, Stat92E, Tin(2), Eya, Mef2

Delta, Dpp, En, Hh, Med, Pyr, Shn, Su\_H\_CSL, Ths, Bap(2), Bin, E\_Spl, Htl, Mad, Nicd, Stat92E, Tin(2), Eya, Mef2

Delta, Dpp, Med, Pyr, Shn, Spi, Su\_H\_CSL, Ths, Upd, Wg, Doc, E\_Spl, Htl, Mad, Nicd, Pan, Slp, Stat92E, Tin(2), Twi, Zfh-1, Eve, Eya, Hbr, Mef2, Pnr

Delta, En, Hh, Med, Pyr, Su\_H\_CSL, Ths, E\_Spl, Htl, Nicd, Stat92E, Tin(2), Brk, D-six4, Eya, Mef2

Med, Pyr, Su\_H\_CSL, Ths, Wg, Da, Htl, Pan, Slp, Stat92E, Tin(2), Twi(2), Zfh-1, Brk, D-six4, Eya, Hbr, Mef2, Poxm

### Genotype: Ci\_0; Tin\_0

Delta, Dpp, En, Hh, Med, Pyr, Shn, Su\_H\_CSL, Ths, Htl, Mad, Nicd

Delta, Dpp, Med, Pyr, Shn, Spi, Su\_H\_CSL, Ths, Upd, Wg, Doc, E\_Spl, Htl, Mad, Nicd, Pan, Slp, Stat92E, Twi, Zfh-1, Eya, Hbr

Delta, En, Hh, Med, Pyr, Su\_H\_CSL, Ths, Htl, Nicd, Brk

Med, Pyr, Su\_H\_CSL, Ths, Wg, Da, Htl, Pan, Slp, Twi(2), Zfh-1, Brk, D-six4, Eya, Hbr, Mef2, Poxm

### Genotype: Bap\_3; Mef2\_1

Delta, Dpp, En, Hh, Med, Pyr, Shn, Su\_H\_CSL, Ths, Bap(3), Bin, Ci, Htl, Mad, Nicd, Tin, Mef2

Delta, Dpp, Med, Pyr, Shn, Spi, Su\_H\_CSL, Ths, Upd, Wg, Bap(3), Bin, Doc, E\_Spl, Htl, Mad, Nicd, Pan, Slp, Stat92E, Tin(2), Twi, Zfh-1, Eve, Eya, Hbr, Mef2, Pnr

Delta, En, Hh, Med, Pyr, Su\_H\_CSL, Ths, Bap(3), Bin, Ci, Htl, Nicd, Brk, Mef2, Srp(2)

Med, Pyr, Su\_H\_CSL, Ths, Wg, Bap(3), Bin, Da, Htl, Pan, Slp, Twi(2), Zfh-1, Brk, D-six4, Eya, Hbr, Mef2, Poxm

### Genotype: Bap\_3; Mef2\_0

Delta, Dpp, En, Hh, Med, Pyr, Shn, Su\_H\_CSL, Ths, Bap(3), Bin, Ci, Htl, Mad, Nicd, Tin

Delta, Dpp, Med, Pyr, Shn, Spi, Su\_H\_CSL, Ths, Upd, Wg, Bap(3), Bin, Doc, E\_Spl, Htl, Mad, Nicd, Pan, Slp, Stat92E, Tin(2), Twi, Zfh-1, Eve, Eya, Hbr, Pnr

Delta, En, Hh, Med, Pyr, Su\_H\_CSL, Ths, Bap(3), Bin, Ci, Htl, Nicd, Brk, Srp(2)

Med, Pyr, Su\_H\_CSL, Ths, Wg, Bap(3), Bin, Da, Htl, Pan, Slp, Twi(2), Zfh-1, Brk, D-six4, Eya, Hbr, Poxm

### Genotype: Pan\_1

Delta, Dpp, En, Hh, Med, Pyr, Shn, Su\_H\_CSL, Ths, Bap, Ci, Doc, Htl, Mad, Nicd, Pan, Slp, Tin, Hbr, Mef2, Pnr

Delta, Dpp, En, Hh, Med, Pyr, Shn, Su\_H\_CSL, Ths, Bap, Ci, Doc, Htl, Mad, Nicd, Pan, Slp, Tin, Twi, Zfh-1, Eya, Hbr, Mef2, Pnr

Delta, Dpp, Med, Pyr, Shn, Spi, Su\_H\_CSL, Ths, Upd, Wg, Doc, E\_Spl, Htl, Mad, Nicd, Pan, Slp, Stat92E, Tin(2), Twi, Zfh-1, Eve, Eya, Hbr, Mef2, Pnr

Delta, En, Hh, Med, Pyr, Su\_H\_CSL, Ths, Ci, Htl, Nicd, Pan, Slp, Brk, Hbr, Srp(2)

Delta, En, Hh, Med, Pyr, Su\_H\_CSL, Ths, Ci, Htl, Nicd, Pan, Slp, Twi, Zfh-1, Brk, D-six4, Eya, Hbr, Poxm, Srp(2)

Med, Pyr, Su\_H\_CSL, Ths, Wg, Da, Htl, Pan, Slp, Twi(2), Zfh-1, Brk, D-six4, Eya, Hbr, Mef2, Poxm

### Genotype: Mef2\_1; Twi\_2

Delta, Dpp, En, Hh, Med, Pyr, Shn, Su\_H\_CSL, Ths, Bap(3), Bin, Ci, Htl, Mad, Nicd, Tin, Twi(2), Zfh-1, Eya, Mef2

Delta, Dpp, Med, Pyr, Shn, Spi, Su\_H\_CSL, Ths, Upd, Wg, Doc, E\_Spl, Htl, Mad, Nicd, Pan, Slp, Stat92E, Tin(2), Twi(2), Zfh-1, Eve, Eya, Hbr, Mef2, Pnr

Delta, En, Hh, Med, Pyr, Su\_H\_CSL, Ths, Ci, Htl, Nicd, Twi(2), Zfh-1, Brk, D-six4, Eya, Mef2, Srp(2)

Med, Pyr, Su\_H\_CSL, Ths, Wg, Da, Htl, Pan, Slp, Twi(2), Zfh-1, Brk, D-six4, Eya, Hbr, Mef2, Poxm

### Genotype: Ci\_1; Mad\_1

Delta, Dpp, En, Hh, Med, Pyr, Shn, Su\_H\_CSL, Ths, Bap(3), Bin, Ci, Htl, Mad, Nicd, Tin, Mef2

Delta, Dpp, Med, Pyr, Shn, Spi, Su\_H\_CSL, Ths, Upd, Wg, Bap, Ci, Doc, E\_Spl, Htl, Mad, Nicd, Pan, Slp, Stat92E, Tin(2), Twi, Zfh-1, Eve, Eya, Hbr, Mef2, Pnr

Delta, En, Hh, Med, Pyr, Su\_H\_CSL, Ths, Bap(3), Bin, Ci, Htl, Mad, Nicd, Tin, Brk, Mef2

Med, Pyr, Su\_H\_CSL, Ths, Wg, Bap, Ci, Da, Doc, Htl, Mad, Pan, Slp, Tin, Twi(2), Zfh-1, Brk, Eya, Hbr, Mef2, Pnr

### Genotype: Mef2\_1; Twi\_0

Delta, Dpp, En, Hh, Med, Pyr, Shn, Su\_H\_CSL, Ths, Bap(3), Bin, Ci, Htl, Mad, Nicd, Tin, Mef2

Delta, Dpp, Med, Pyr, Shn, Spi, Su\_H\_CSL, Ths, Upd, Wg, Doc, E\_Spl, Htl, Mad, Nicd, Pan, Slp, Stat92E, Tin(2), Eya, Hbr, Mef2, Pnr

Delta, En, Hh, Med, Pyr, Su\_H\_CSL, Ths, Ci, Htl, Nicd, Brk, Mef2, Srp(2)

Med, Pyr, Su\_H\_CSL, Ths, Wg, Da, Htl, Pan, Slp, Brk, Hbr, Mef2

### Genotype: Mad\_0; Pnr\_0

Delta, Dpp, En, Hh, Med, Pyr, Shn, Su\_H\_CSL, Ths, Ci, Htl, Nicd, Brk, Srp(2)

Delta, Dpp, Med, Pyr, Shn, Spi, Su\_H\_CSL, Ths, Upd, Wg, E\_Spl, Htl, Nicd, Pan, Slp, Stat92E, Twi, Zfh-1, Brk, D-six4, Eya, Hbr, Poxm

Delta, En, Hh, Med, Pyr, Su\_H\_CSL, Ths, Ci, Htl, Nicd, Brk, Srp(2)

Med, Pyr, Su\_H\_CSL, Ths, Wg, Da, Htl, Pan, Slp, Twi(2), Zfh-1, Brk, D-six4, Eya, Hbr, Mef2, Poxm

### Genotype: Mad\_0; Pnr\_1

Delta, Dpp, En, Hh, Med, Pyr, Shn, Su\_H\_CSL, Ths, Ci, Htl, Nicd, Brk, Pnr, Srp(2)

Delta, Dpp, Med, Pyr, Shn, Spi, Su\_H\_CSL, Ths, Upd, Wg, E\_Spl, Htl, Nicd, Pan, Slp, Stat92E, Twi, Zfh-1, Brk, D-six4, Eya, Hbr, Pnr, Poxm

Delta, En, Hh, Med, Pyr, Su\_H\_CSL, Ths, Ci, Htl, Nicd, Brk, Pnr, Srp(2)

Med, Pyr, Su\_H\_CSL, Ths, Wg, Da, Htl, Pan, Slp, Twi(2), Zfh-1, Brk, D-six4, Eya, Hbr, Mef2, Pnr, Poxm

### Genotype: Nicd\_1

Delta, Dpp, En, Hh, Med, Pyr, Shn, Su\_H\_CSL, Ths, Bap(3), Bin, Ci, Htl, Mad, Nicd, Tin, Mef2

Delta, Dpp, Med, Pyr, Shn, Spi, Su\_H\_CSL, Ths, Upd, Wg, Doc, E\_Spl, Htl, Mad, Nicd, Pan, Slp, Stat92E, Tin(2), Twi, Zfh-1, Eve, Eya, Hbr, Mef2, Pnr

Delta, En, Hh, Med, Pyr, Su\_H\_CSL, Ths, Ci, Htl, Nicd, Brk, Srp(2)

Med, Pyr, Su\_H\_CSL, Ths, Wg, Htl, Nicd, Pan, Slp, Twi, Zfh-1, Brk, D-six4, Eya, Hbr, Poxm

### Genotype: Med\_0; Twi\_2

Delta, Dpp, En, Hh, Pyr, Shn, Su\_H\_CSL, Ths, Bap(2), Bin, Ci, Htl, Mad, Nicd, Tin, Twi(2), Zfh-1, Brk, D-six4, Eya, Mef2, Srp(2)

Delta, Dpp, Pyr, Shn, Spi, Su\_H\_CSL, Ths, Upd, Wg, E\_Spl, Htl, Mad, Nicd, Pan, Slp, Stat92E, Tin, Twi(2), Zfh-1, Brk, D-six4, Eya, Hbr, Mef2, Poxm

Delta, En, Hh, Pyr, Su\_H\_CSL, Ths, Ci, Htl, Nicd, Twi(2), Zfh-1, Brk, D-six4, Eya, Mef2, Srp(2)

Pyr, Su\_H\_CSL, Ths, Wg, Da, Htl, Pan, Slp, Twi(2), Zfh-1, Brk, D-six4, Eya, Hbr, Mef2, Poxm

### Genotype: Srp\_0

Delta, Dpp, En, Hh, Med, Pyr, Shn, Su\_H\_CSL, Ths, Bap(3), Bin, Ci, Htl, Mad, Nicd, Tin, Mef2

Delta, Dpp, Med, Pyr, Shn, Spi, Su\_H\_CSL, Ths, Upd, Wg, Doc, E\_Spl, Htl, Mad, Nicd, Pan, Slp, Stat92E, Tin(2), Twi, Zfh-1, Eve, Eya, Hbr, Mef2, Pnr

Delta, En, Hh, Med, Pyr, Su\_H\_CSL, Ths, Ci, Htl, Nicd, Brk

Med, Pyr, Su\_H\_CSL, Ths, Wg, Da, Htl, Pan, Slp, Twi(2), Zfh-1, Brk, D-six4, Eya, Hbr, Mef2, Poxm

### Genotype: Ci\_1; Pnr\_1

Delta, Dpp, En, Hh, Med, Pyr, Shn, Su\_H\_CSL, Ths, Bap(3), Bin, Ci, Htl, Mad, Nicd, Tin, Mef2, Pnr

Delta, Dpp, Med, Pyr, Shn, Spi, Su\_H\_CSL, Ths, Upd, Wg, Bap, Ci, Doc, E\_Spl, Htl, Mad, Nicd, Pan, Slp, Stat92E, Tin(2), Twi, Zfh-1, Eve, Eya, Hbr, Mef2, Pnr

Delta, En, Hh, Med, Pyr, Su\_H\_CSL, Ths, Ci, Htl, Nicd, Brk, Pnr, Srp(2)

Med, Pyr, Su\_H\_CSL, Ths, Wg, Ci, Da, Htl, Pan, Slp, Twi(2), Zfh-1, Brk, D-six4, Eya, Hbr, Mef2, Pnr, Poxm, Srp

### Genotype: Bap\_3; Med\_0

Delta, Dpp, En, Hh, Pyr, Shn, Su\_H\_CSL, Ths, Bap(3), Bin, Ci, Htl, Mad, Nicd, Tin, Brk, D-six4, Mef2, Srp(2)

Delta, Dpp, Pyr, Shn, Spi, Su\_H\_CSL, Ths, Upd, Wg, Bap(3), Bin, E\_Spl, Htl, Mad, Nicd, Pan, Slp, Stat92E, Tin, Twi, Zfh-1, Brk, D-six4, Eya, Hbr, Mef2, Poxm

Delta, En, Hh, Pyr, Su\_H\_CSL, Ths, Bap(3), Bin, Ci, Htl, Nicd, Brk, Srp(2)

Pyr, Su\_H\_CSL, Ths, Wg, Bap(3), Bin, Da, Htl, Pan, Slp, Twi(2), Zfh-1, Brk, D-six4, Eya, Hbr, Mef2, Poxm

### Genotype: Bap\_3; Med\_1

Delta, Dpp, En, Hh, Med, Pyr, Shn, Su\_H\_CSL, Ths, Bap(3), Bin, Ci, Htl, Mad, Nicd, Tin, Mef2

Delta, Dpp, Med, Pyr, Shn, Spi, Su\_H\_CSL, Ths, Upd, Wg, Bap(3), Bin, Doc, E\_Spl, Htl, Mad, Nicd, Pan, Slp, Stat92E, Tin(2), Twi, Zfh-1, Eve, Eya, Hbr, Mef2, Pnr

Delta, En, Hh, Med, Pyr, Su\_H\_CSL, Ths, Bap(3), Bin, Ci, Htl, Nicd, Brk, Srp(2)

Med, Pyr, Su\_H\_CSL, Ths, Wg, Bap(3), Bin, Da, Htl, Pan, Slp, Twi(2), Zfh-1, Brk, D-six4, Eya, Hbr, Mef2, Poxm

### Genotype: Nicd\_0; Srp\_2

Delta, Dpp, En, Hh, Med, Pyr, Shn, Su\_H\_CSL, Ths, Bap(3), Bin, Ci, Da, Htl, Mad, Tin, Mef2, Srp(2)

Delta, Dpp, En, Hh, Med, Pyr, Shn, Su\_H\_CSL, Ths, Bap(3), Bin, Ci, Da, Htl, Mad, Tin, Twi, Zfh-1, Eya, Mef2, Srp(2)

Delta, Dpp, Med, Pyr, Shn, Spi, Su\_H\_CSL, Ths, Upd, Wg, Da, Doc, Htl, Mad, Pan, Slp, Stat92E, Tin(2), Twi(2), Zfh-1, Eve, Eya, Hbr, Mef2, Pnr, Srp(2)

Delta, En, Hh, Med, Pyr, Su\_H\_CSL, Ths, Ci, Da, Htl, Brk, Srp(2)

Delta, En, Hh, Med, Pyr, Su\_H\_CSL, Ths, Ci, Da, Htl, Twi, Zfh-1, Brk, D-six4, Eya, Srp(2)

Med, Pyr, Su\_H\_CSL, Ths, Wg, Da, Htl, Pan, Slp, Twi(2), Zfh-1, Brk, D-six4, Eya, Hbr, Mef2, Poxm, Srp(2)

### Genotype: Nicd\_0; Srp\_0

Delta, Dpp, En, Hh, Med, Pyr, Shn, Su\_H\_CSL, Ths, Bap(3), Bin, Ci, Da, Htl, Mad, Tin, Mef2

Delta, Dpp, En, Hh, Med, Pyr, Shn, Su\_H\_CSL, Ths, Bap(3), Bin, Ci, Da, Htl, Mad, Tin, Twi, Zfh-1, Eya, Mef2

Delta, Dpp, Med, Pyr, Shn, Spi, Su\_H\_CSL, Ths, Upd, Wg, Da, Doc, Htl, Mad, Pan, Slp, Stat92E, Tin(2), Twi(2), Zfh-1, Eve, Eya, Hbr, Mef2, Pnr

Delta, En, Hh, Med, Pyr, Su\_H\_CSL, Ths, Ci, Da, Htl, Brk

Delta, En, Hh, Med, Pyr, Su\_H\_CSL, Ths, Ci, Da, Htl, Twi, Zfh-1, Brk, D-six4, Eya

Med, Pyr, Su\_H\_CSL, Ths, Wg, Da, Htl, Pan, Slp, Twi(2), Zfh-1, Brk, D-six4, Eya, Hbr, Mef2, Poxm

### Genotype: Mad\_1; Mef2\_0

Delta, Dpp, En, Hh, Med, Pyr, Shn, Su\_H\_CSL, Ths, Bap(3), Bin, Ci, Htl, Mad, Nicd, Tin

Delta, Dpp, Med, Pyr, Shn, Spi, Su\_H\_CSL, Ths, Upd, Wg, Doc, E\_Spl, Htl, Mad, Nicd, Pan, Slp, Stat92E, Tin(2), Twi, Zfh-1, Eve, Eya, Hbr, Pnr

Delta, En, Hh, Med, Pyr, Su\_H\_CSL, Ths, Bap(3), Bin, Ci, Htl, Mad, Nicd, Tin, Brk

Med, Pyr, Su\_H\_CSL, Ths, Wg, Da, Doc, Htl, Mad, Pan, Slp, Tin, Twi(2), Zfh-1, Brk, Eya, Hbr, Pnr

### Genotype: Mad\_1; Mef2\_1

Delta, Dpp, En, Hh, Med, Pyr, Shn, Su\_H\_CSL, Ths, Bap(3), Bin, Ci, Htl, Mad, Nicd, Tin, Mef2

Delta, Dpp, Med, Pyr, Shn, Spi, Su\_H\_CSL, Ths, Upd, Wg, Doc, E\_Spl, Htl, Mad, Nicd, Pan, Slp, Stat92E, Tin(2), Twi, Zfh-1, Eve, Eya, Hbr, Mef2, Pnr

Delta, En, Hh, Med, Pyr, Su\_H\_CSL, Ths, Bap(3), Bin, Ci, Htl, Mad, Nicd, Tin, Brk, Mef2

Med, Pyr, Su\_H\_CSL, Ths, Wg, Da, Doc, Htl, Mad, Pan, Slp, Tin, Twi(2), Zfh-1, Brk, Eya, Hbr, Mef2, Pnr

### Genotype: Doc\_0; Twi\_0

Delta, Dpp, En, Hh, Med, Pyr, Shn, Su\_H\_CSL, Ths, Bap(3), Bin, Ci, Htl, Mad, Nicd, Tin, Mef2

Delta, Dpp, Med, Pyr, Shn, Spi, Su\_H\_CSL, Ths, Upd, Wg, E\_Spl, Htl, Mad, Nicd, Pan, Slp, Stat92E, Tin(2), Eya, Hbr, Mef2

Delta, En, Hh, Med, Pyr, Su\_H\_CSL, Ths, Ci, Htl, Nicd, Brk, Srp(2)

Med, Pyr, Su\_H\_CSL, Ths, Wg, Da, Htl, Pan, Slp, Brk, Hbr

### Genotype: Doc\_0; Twi\_2

Delta, Dpp, En, Hh, Med, Pyr, Shn, Su\_H\_CSL, Ths, Bap(3), Bin, Ci, Htl, Mad, Nicd, Tin, Twi(2), Zfh-1, Eya, Mef2

Delta, Dpp, Med, Pyr, Shn, Spi, Su\_H\_CSL, Ths, Upd, Wg, E\_Spl, Htl, Mad, Nicd, Pan, Slp, Stat92E, Tin(2), Twi(2), Zfh-1, Eve, Eya, Hbr, Mef2

Delta, En, Hh, Med, Pyr, Su\_H\_CSL, Ths, Ci, Htl, Nicd, Twi(2), Zfh-1, Brk, D-six4, Eya, Mef2, Srp(2)

Med, Pyr, Su\_H\_CSL, Ths, Wg, Da, Htl, Pan, Slp, Twi(2), Zfh-1, Brk, D-six4, Eya, Hbr, Mef2, Poxm

### Genotype: Bin\_2; Mef2\_0

Delta, Dpp, En, Hh, Med, Pyr, Shn, Su\_H\_CSL, Ths, Bap(3), Bin(2), Ci, Htl, Mad, Nicd, Tin

Delta, Dpp, Med, Pyr, Shn, Spi, Su\_H\_CSL, Ths, Upd, Wg, Bap(3), Bin(2), Doc, E\_Spl, Htl, Mad, Nicd, Pan, Slp, Stat92E, Tin(2), Twi, Zfh-1, Eve, Eya, Hbr, Pnr

Delta, En, Hh, Med, Pyr, Su\_H\_CSL, Ths, Bap(3), Bin(2), Ci, Htl, Nicd, Brk, Srp(2)

Med, Pyr, Su\_H\_CSL, Ths, Wg, Bap(3), Bin(2), Da, Htl, Pan, Slp, Twi(2), Zfh-1, Brk, D-six4, Eya, Hbr, Poxm

### Genotype: Bin\_2; Mef2\_1

Delta, Dpp, En, Hh, Med, Pyr, Shn, Su\_H\_CSL, Ths, Bap(3), Bin(2), Ci, Htl, Mad, Nicd, Tin, Mef2

Delta, Dpp, Med, Pyr, Shn, Spi, Su\_H\_CSL, Ths, Upd, Wg, Bap(3), Bin(2), Doc, E\_Spl, Htl, Mad, Nicd, Pan, Slp, Stat92E, Tin(2), Twi, Zfh-1, Eve, Eya, Hbr, Mef2, Pnr

Delta, En, Hh, Med, Pyr, Su\_H\_CSL, Ths, Bap(3), Bin(2), Ci, Htl, Nicd, Brk, Mef2, Srp(2)

Med, Pyr, Su\_H\_CSL, Ths, Wg, Bap(3), Bin(2), Da, Htl, Pan, Slp, Twi(2), Zfh-1, Brk, D-six4, Eya, Hbr, Mef2, Poxm

### Genotype: Doc\_1; Mad\_1

Delta, Dpp, En, Hh, Med, Pyr, Shn, Su\_H\_CSL, Ths, Bap(3), Bin, Ci, Doc, Htl, Mad, Nicd, Tin, Mef2, Pnr

Delta, Dpp, Med, Pyr, Shn, Spi, Su\_H\_CSL, Ths, Upd, Wg, Doc, E\_Spl, Htl, Mad, Nicd, Pan, Slp, Stat92E, Tin(2), Twi, Zfh-1, Eve, Eya, Hbr, Mef2, Pnr

Delta, En, Hh, Med, Pyr, Su\_H\_CSL, Ths, Bap(3), Bin, Ci, Doc, Htl, Mad, Nicd, Tin, Brk, Mef2, Pnr

Med, Pyr, Su\_H\_CSL, Ths, Wg, Da, Doc, Htl, Mad, Pan, Slp, Tin, Twi(2), Zfh-1, Brk, Eya, Hbr, Mef2, Pnr

### Genotype: Doc\_1; Mad\_0

Delta, Dpp, En, Hh, Med, Pyr, Shn, Su\_H\_CSL, Ths, Ci, Doc, Htl, Nicd, Brk, Srp(2)

Delta, Dpp, Med, Pyr, Shn, Spi, Su\_H\_CSL, Ths, Upd, Wg, Doc, E\_Spl, Htl, Nicd, Pan, Slp, Stat92E, Twi, Zfh-1, Brk, D-six4, Eya, Hbr, Poxm

Delta, En, Hh, Med, Pyr, Su\_H\_CSL, Ths, Ci, Doc, Htl, Nicd, Brk, Srp(2)

Med, Pyr, Su\_H\_CSL, Ths, Wg, Da, Doc, Htl, Pan, Slp, Twi(2), Zfh-1, Brk, D-six4, Eya, Hbr, Mef2, Poxm

### Genotype: Ci\_1; Med\_0

Delta, Dpp, En, Hh, Pyr, Shn, Su\_H\_CSL, Ths, Bap(2), Bin, Ci, Htl, Mad, Nicd, Tin, Brk, D-six4, Mef2, Srp(2)

Delta, Dpp, Pyr, Shn, Spi, Su\_H\_CSL, Ths, Upd, Wg, Ci, E\_Spl, Htl, Mad, Nicd, Pan, Slp, Stat92E, Tin, Twi, Zfh-1, Brk, D-six4, Eya, Hbr, Mef2, Poxm, Srp

Delta, En, Hh, Pyr, Su\_H\_CSL, Ths, Ci, Htl, Nicd, Brk, Srp(2)

Pyr, Su\_H\_CSL, Ths, Wg, Ci, Da, Htl, Pan, Slp, Twi(2), Zfh-1, Brk, D-six4, Eya, Hbr, Mef2, Poxm, Srp

### Genotype: Ci\_1; Med\_1

Delta, Dpp, En, Hh, Med, Pyr, Shn, Su\_H\_CSL, Ths, Bap(3), Bin, Ci, Htl, Mad, Nicd, Tin, Mef2

Delta, Dpp, Med, Pyr, Shn, Spi, Su\_H\_CSL, Ths, Upd, Wg, Bap, Ci, Doc, E\_Spl, Htl, Mad, Nicd, Pan, Slp, Stat92E, Tin(2), Twi, Zfh-1, Eve, Eya, Hbr, Mef2, Pnr

Delta, En, Hh, Med, Pyr, Su\_H\_CSL, Ths, Ci, Htl, Nicd, Brk, Srp(2)

Med, Pyr, Su\_H\_CSL, Ths, Wg, Ci, Da, Htl, Pan, Slp, Twi(2), Zfh-1, Brk, D-six4, Eya, Hbr, Mef2, Poxm, Srp

### Genotype: Ci\_0; Pnr\_1

Delta, Dpp, En, Hh, Med, Pyr, Shn, Su\_H\_CSL, Ths, Bap, Htl, Mad, Nicd, Tin, Mef2, Pnr

Delta, Dpp, En, Hh, Med, Pyr, Shn, Su\_H\_CSL, Ths, Bap(2), Bin, Htl, Mad, Nicd, Tin, Mef2, Pnr

Delta, Dpp, Med, Pyr, Shn, Spi, Su\_H\_CSL, Ths, Upd, Wg, Doc, E\_Spl, Htl, Mad, Nicd, Pan, Slp, Stat92E, Tin(2), Twi, Zfh-1, Eve, Eya, Hbr, Mef2, Pnr

Delta, En, Hh, Med, Pyr, Su\_H\_CSL, Ths, Htl, Nicd, Brk, Pnr

Med, Pyr, Su\_H\_CSL, Ths, Wg, Da, Htl, Pan, Slp, Twi(2), Zfh-1, Brk, D-six4, Eya, Hbr, Mef2, Pnr, Poxm

### Genotype: Ci\_0; Pnr\_0

Delta, Dpp, En, Hh, Med, Pyr, Shn, Su\_H\_CSL, Ths, Bap, Htl, Mad, Nicd, Tin, Mef2

Delta, Dpp, En, Hh, Med, Pyr, Shn, Su\_H\_CSL, Ths, Bap(2), Bin, Htl, Mad, Nicd, Tin, Mef2

Delta, Dpp, Med, Pyr, Shn, Spi, Su\_H\_CSL, Ths, Upd, Wg, Doc, E\_Spl, Htl, Mad, Nicd, Pan, Slp, Stat92E, Tin(2), Twi, Zfh-1, Eve, Eya, Hbr, Mef2

Delta, En, Hh, Med, Pyr, Su\_H\_CSL, Ths, Htl, Nicd, Brk

Med, Pyr, Su\_H\_CSL, Ths, Wg, Da, Htl, Pan, Slp, Twi(2), Zfh-1, Brk, D-six4, Eya, Hbr, Mef2, Poxm

### Genotype: Bin\_2; Srp\_2

Delta, Dpp, En, Hh, Med, Pyr, Shn, Su\_H\_CSL, Ths, Bap(3), Bin(2), Ci, Htl, Mad, Nicd, Tin, Mef2, Srp(2)

Delta, Dpp, Med, Pyr, Shn, Spi, Su\_H\_CSL, Ths, Upd, Wg, Bap(3), Bin(2), Doc, E\_Spl, Htl, Mad, Nicd, Pan, Slp, Stat92E, Tin(2), Twi, Zfh-1, Eve, Eya, Hbr, Mef2, Pnr, Srp(2)

Delta, En, Hh, Med, Pyr, Su\_H\_CSL, Ths, Bap(3), Bin(2), Ci, Htl, Nicd, Brk, Srp(2)

Med, Pyr, Su\_H\_CSL, Ths, Wg, Bap(3), Bin(2), Da, Htl, Pan, Slp, Twi(2), Zfh-1, Brk, D-six4, Eya, Hbr, Mef2, Poxm, Srp(2)

### Genotype: Bin\_2; Srp\_0

Delta, Dpp, En, Hh, Med, Pyr, Shn, Su\_H\_CSL, Ths, Bap(3), Bin(2), Ci, Htl, Mad, Nicd, Tin, Mef2

Delta, Dpp, Med, Pyr, Shn, Spi, Su\_H\_CSL, Ths, Upd, Wg, Bap(3), Bin(2), Doc, E\_Spl, Htl, Mad, Nicd, Pan, Slp, Stat92E, Tin(2), Twi, Zfh-1, Eve, Eya, Hbr, Mef2, Pnr

Delta, En, Hh, Med, Pyr, Su\_H\_CSL, Ths, Bap(3), Bin(2), Ci, Htl, Nicd, Brk

Med, Pyr, Su\_H\_CSL, Ths, Wg, Bap(3), Bin(2), Da, Htl, Pan, Slp, Twi(2), Zfh-1, Brk, D-six4, Eya, Hbr, Mef2, Poxm

### Genotype: Pan\_1; Twi\_2

Delta, Dpp, En, Hh, Med, Pyr, Shn, Su\_H\_CSL, Ths, Bap, Ci, Doc, Htl, Mad, Nicd, Pan, Slp, Tin, Twi(2), Zfh-1, Eya, Hbr, Mef2, Pnr

Delta, Dpp, Med, Pyr, Shn, Spi, Su\_H\_CSL, Ths, Upd, Wg, Doc, E\_Spl, Htl, Mad, Nicd, Pan, Slp, Stat92E, Tin(2), Twi(2), Zfh-1, Eve, Eya, Hbr, Mef2, Pnr

Delta, En, Hh, Med, Pyr, Su\_H\_CSL, Ths, Ci, Htl, Nicd, Pan, Slp, Twi(2), Zfh-1, Brk, D-six4, Eya, Hbr, Mef2, Poxm, Srp(2)

Med, Pyr, Su\_H\_CSL, Ths, Wg, Da, Htl, Pan, Slp, Twi(2), Zfh-1, Brk, D-six4, Eya, Hbr, Mef2, Poxm

### Genotype: Pan\_1; Twi\_0

Delta, Dpp, En, Hh, Med, Pyr, Shn, Su\_H\_CSL, Ths, Bap, Ci, Doc, Htl, Mad, Nicd, Pan, Slp, Tin, Hbr, Mef2, Pnr

Delta, Dpp, Med, Pyr, Shn, Spi, Su\_H\_CSL, Ths, Upd, Wg, Doc, E\_Spl, Htl, Mad, Nicd, Pan, Slp, Stat92E, Tin(2), Eya, Hbr, Mef2, Pnr

Delta, En, Hh, Med, Pyr, Su\_H\_CSL, Ths, Ci, Htl, Nicd, Pan, Slp, Brk, Hbr, Srp(2)

Med, Pyr, Su\_H\_CSL, Ths, Wg, Da, Htl, Pan, Slp, Brk, Hbr

### Genotype: Mad\_1

Delta, Dpp, En, Hh, Med, Pyr, Shn, Su\_H\_CSL, Ths, Bap(3), Bin, Ci, Htl, Mad, Nicd, Tin, Mef2

Delta, Dpp, Med, Pyr, Shn, Spi, Su\_H\_CSL, Ths, Upd, Wg, Doc, E\_Spl, Htl, Mad, Nicd, Pan, Slp, Stat92E, Tin(2), Twi, Zfh-1, Eve, Eya, Hbr, Mef2, Pnr

Delta, En, Hh, Med, Pyr, Su\_H\_CSL, Ths, Bap(3), Bin, Ci, Htl, Mad, Nicd, Tin, Brk, Mef2

Med, Pyr, Su\_H\_CSL, Ths, Wg, Da, Doc, Htl, Mad, Pan, Slp, Tin, Twi(2), Zfh-1, Brk, Eya, Hbr, Mef2, Pnr

### Genotype: Nicd\_1; Pan\_1

Delta, Dpp, En, Hh, Med, Pyr, Shn, Su\_H\_CSL, Ths, Bap, Ci, Doc, Htl, Mad, Nicd, Pan, Slp, Tin, Hbr, Mef2, Pnr

Delta, Dpp, En, Hh, Med, Pyr, Shn, Su\_H\_CSL, Ths, Bap, Ci, Doc, Htl, Mad, Nicd, Pan, Slp, Tin, Twi, Zfh-1, Eya, Hbr, Mef2, Pnr

Delta, Dpp, Med, Pyr, Shn, Spi, Su\_H\_CSL, Ths, Upd, Wg, Doc, E\_Spl, Htl, Mad, Nicd, Pan, Slp, Stat92E, Tin(2), Twi, Zfh-1, Eve, Eya, Hbr, Mef2, Pnr

Delta, En, Hh, Med, Pyr, Su\_H\_CSL, Ths, Ci, Htl, Nicd, Pan, Slp, Brk, Hbr, Srp(2)

Delta, En, Hh, Med, Pyr, Su\_H\_CSL, Ths, Ci, Htl, Nicd, Pan, Slp, Twi, Zfh-1, Brk, D-six4, Eya, Hbr, Poxm, Srp(2)

Med, Pyr, Su\_H\_CSL, Ths, Wg, Htl, Nicd, Pan, Slp, Twi, Zfh-1, Brk, D-six4, Eya, Hbr, Poxm

### Genotype: Nicd\_1; Pan\_0

Delta, Dpp, En, Hh, Med, Pyr, Shn, Su\_H\_CSL, Ths, Bap(3), Bin, Ci, Htl, Mad, Nicd, Tin, Mef2

Delta, Dpp, Med, Pyr, Shn, Spi, Su\_H\_CSL, Ths, Upd, Wg, Bap, E\_Spl, Htl, Mad, Nicd, Stat92E, Tin, Mef2

Delta, En, Hh, Med, Pyr, Su\_H\_CSL, Ths, Ci, Htl, Nicd, Brk, Srp(2)

Med, Pyr, Su\_H\_CSL, Ths, Wg, Htl, Nicd, Brk

### Genotype: Bin\_0; Pnr\_1

Delta, Dpp, En, Hh, Med, Pyr, Shn, Su\_H\_CSL, Ths, Bap(3), Ci, Htl, Mad, Nicd, Tin, Mef2, Pnr

Delta, Dpp, Med, Pyr, Shn, Spi, Su\_H\_CSL, Ths, Upd, Wg, Doc, E\_Spl, Htl, Mad, Nicd, Pan, Slp, Stat92E, Tin(2), Twi, Zfh-1, Eve, Eya, Hbr, Mef2, Pnr

Delta, En, Hh, Med, Pyr, Su\_H\_CSL, Ths, Ci, Htl, Nicd, Brk, Pnr, Srp(2)

Med, Pyr, Su\_H\_CSL, Ths, Wg, Da, Htl, Pan, Slp, Twi(2), Zfh-1, Brk, D-six4, Eya, Hbr, Mef2, Pnr, Poxm

### Genotype: Bin\_0; Pnr\_0

Delta, Dpp, En, Hh, Med, Pyr, Shn, Su\_H\_CSL, Ths, Bap(3), Ci, Htl, Mad, Nicd, Tin, Mef2

Delta, Dpp, Med, Pyr, Shn, Spi, Su\_H\_CSL, Ths, Upd, Wg, Doc, E\_Spl, Htl, Mad, Nicd, Pan, Slp, Stat92E, Tin(2), Twi, Zfh-1, Eve, Eya, Hbr, Mef2

Delta, En, Hh, Med, Pyr, Su\_H\_CSL, Ths, Ci, Htl, Nicd, Brk, Srp(2)

Med, Pyr, Su\_H\_CSL, Ths, Wg, Da, Htl, Pan, Slp, Twi(2), Zfh-1, Brk, D-six4, Eya, Hbr, Mef2, Poxm

### Genotype: Mad\_0; Srp\_0

Delta, Dpp, En, Hh, Med, Pyr, Shn, Su\_H\_CSL, Ths, Ci, Htl, Nicd, Brk

Delta, Dpp, Med, Pyr, Shn, Spi, Su\_H\_CSL, Ths, Upd, Wg, E\_Spl, Htl, Nicd, Pan, Slp, Stat92E, Twi, Zfh-1, Brk, D-six4, Eya, Hbr, Poxm

Delta, En, Hh, Med, Pyr, Su\_H\_CSL, Ths, Ci, Htl, Nicd, Brk

Med, Pyr, Su\_H\_CSL, Ths, Wg, Da, Htl, Pan, Slp, Twi(2), Zfh-1, Brk, D-six4, Eya, Hbr, Mef2, Poxm

### Genotype: Nicd\_0; Pan\_0

Delta, Dpp, En, Hh, Med, Pyr, Shn, Su\_H\_CSL, Ths, Bap(3), Bin, Ci, Da, Htl, Mad, Tin, Mef2

Delta, Dpp, En, Hh, Med, Pyr, Shn, Su\_H\_CSL, Ths, Bap(3), Bin, Ci, Da, Htl, Mad, Tin, Twi, Zfh-1, Eya, Mef2

Delta, Dpp, Med, Pyr, Shn, Spi, Su\_H\_CSL, Ths, Upd, Wg, Bap, Da, Htl, Mad, Stat92E, Tin, Mef2

Delta, Dpp, Med, Pyr, Shn, Spi, Su\_H\_CSL, Ths, Upd, Wg, Bap, Da, Htl, Mad, Stat92E, Tin, Twi, Zfh-1, Eya, Mef2

Delta, En, Hh, Med, Pyr, Su\_H\_CSL, Ths, Ci, Da, Htl, Brk, Srp(2)

Delta, En, Hh, Med, Pyr, Su\_H\_CSL, Ths, Ci, Da, Htl, Twi, Zfh-1, Brk, D-six4, Eya, Srp(2)

Med, Pyr, Su\_H\_CSL, Ths, Wg, Da, Htl, Brk

Med, Pyr, Su\_H\_CSL, Ths, Wg, Da, Htl, Twi, Zfh-1, Brk, D-six4, Eya

### Genotype: Mad\_0; Srp\_2

Delta, Dpp, En, Hh, Med, Pyr, Shn, Su\_H\_CSL, Ths, Ci, Htl, Nicd, Brk, Srp(2)

Delta, Dpp, Med, Pyr, Shn, Spi, Su\_H\_CSL, Ths, Upd, Wg, E\_Spl, Htl, Nicd, Pan, Slp, Stat92E, Twi, Zfh-1, Brk, D-six4, Eya, Hbr, Poxm, Srp(2)

Delta, En, Hh, Med, Pyr, Su\_H\_CSL, Ths, Ci, Htl, Nicd, Brk, Srp(2)

Med, Pyr, Su\_H\_CSL, Ths, Wg, Da, Htl, Pan, Slp, Twi(2), Zfh-1, Brk, D-six4, Eya, Hbr, Mef2, Poxm, Srp(2)

### Genotype: Doc\_0; Slp\_1

Delta, Dpp, Hh, Med, Pyr, Shn, Su\_H\_CSL, Ths, Bap, Ci, Htl, Mad, Nicd, Slp, Tin, Mef2

Delta, Dpp, Hh, Med, Pyr, Shn, Su\_H\_CSL, Ths, Bap, Ci, Htl, Mad, Nicd, Slp, Tin, Twi, Zfh-1, Eya, Mef2

Delta, Dpp, Med, Pyr, Shn, Spi, Su\_H\_CSL, Ths, Upd, Wg, E\_Spl, Htl, Mad, Nicd, Pan, Slp, Stat92E, Tin(2), Twi, Zfh-1, Eve, Eya, Hbr, Mef2

Delta, Hh, Med, Pyr, Su\_H\_CSL, Ths, Ci, Htl, Nicd, Slp, Brk, Srp

Delta, Hh, Med, Pyr, Su\_H\_CSL, Ths, Ci, Htl, Nicd, Slp, Twi, Zfh-1, Brk, D-six4, Eya, Srp

Med, Pyr, Su\_H\_CSL, Ths, Wg, Da, Htl, Pan, Slp, Twi(2), Zfh-1, Brk, D-six4, Eya, Hbr, Mef2, Poxm

### Genotype: Doc\_0; Slp\_0

Delta, Dpp, En, Hh, Med, Pyr, Shn, Su\_H\_CSL, Ths, Bap(3), Bin, Ci, Htl, Mad, Nicd, Tin, Mef2

Delta, Dpp, En, Med, Pyr, Shn, Spi, Su\_H\_CSL, Ths, Upd, Wg, Bap, E\_Spl, Htl, Mad, Nicd, Pan, Stat92E, Tin(2), Eya, Hbr, Mef2

Delta, En, Hh, Med, Pyr, Su\_H\_CSL, Ths, Ci, Htl, Nicd, Brk, Srp(2)

En, Med, Pyr, Su\_H\_CSL, Ths, Wg, Da, Htl, Pan, Brk, Hbr

En, Med, Pyr, Su\_H\_CSL, Ths, Wg, Da, Htl, Pan, Twi, Zfh-1, Brk, D-six4, Eya, Hbr, Poxm

### Genotype: Doc\_0; Med\_1

Delta, Dpp, En, Hh, Med, Pyr, Shn, Su\_H\_CSL, Ths, Bap(3), Bin, Ci, Htl, Mad, Nicd, Tin, Mef2

Delta, Dpp, Med, Pyr, Shn, Spi, Su\_H\_CSL, Ths, Upd, Wg, E\_Spl, Htl, Mad, Nicd, Pan, Slp, Stat92E, Tin(2), Twi, Zfh-1, Eve, Eya, Hbr, Mef2

Delta, En, Hh, Med, Pyr, Su\_H\_CSL, Ths, Ci, Htl, Nicd, Brk, Srp(2)

Med, Pyr, Su\_H\_CSL, Ths, Wg, Da, Htl, Pan, Slp, Twi(2), Zfh-1, Brk, D-six4, Eya, Hbr, Mef2, Poxm

### Genotype: Srp\_2; Tin\_0

Delta, Dpp, En, Hh, Med, Pyr, Shn, Su\_H\_CSL, Ths, Ci, Htl, Mad, Nicd, Srp(2)

Delta, Dpp, Med, Pyr, Shn, Spi, Su\_H\_CSL, Ths, Upd, Wg, Doc, E\_Spl, Htl, Mad, Nicd, Pan, Slp, Stat92E, Twi, Zfh-1, Eya, Hbr, Srp(2)

Delta, En, Hh, Med, Pyr, Su\_H\_CSL, Ths, Ci, Htl, Nicd, Brk, Srp(2)

Med, Pyr, Su\_H\_CSL, Ths, Wg, Da, Htl, Pan, Slp, Twi(2), Zfh-1, Brk, D-six4, Eya, Hbr, Mef2, Poxm, Srp(2)

### Genotype: Srp\_2; Tin\_2

Delta, Dpp, En, Hh, Med, Pyr, Shn, Su\_H\_CSL, Ths, Bap(3), Bin, Ci, E\_Spl, Htl, Mad, Nicd, Stat92E, Tin(2), Eya, Mef2, Srp(2)

Delta, Dpp, Med, Pyr, Shn, Spi, Su\_H\_CSL, Ths, Upd, Wg, Doc, E\_Spl, Htl, Mad, Nicd, Pan, Slp, Stat92E, Tin(2), Twi, Zfh-1, Eve, Eya, Hbr, Mef2, Pnr, Srp(2)

Delta, En, Hh, Med, Pyr, Su\_H\_CSL, Ths, Bap(2), Bin, Ci, E\_Spl, Htl, Nicd, Stat92E, Tin(2), Brk, D-six4, Eya, Mef2, Srp(2)

Med, Pyr, Su\_H\_CSL, Ths, Wg, Da, Htl, Pan, Slp, Stat92E, Tin(2), Twi(2), Zfh-1, Brk, D-six4, Eya, Hbr, Mef2, Poxm, Srp(2)

### Genotype: Mef2\_0; Nicd\_0

Delta, Dpp, En, Hh, Med, Pyr, Shn, Su\_H\_CSL, Ths, Bap(3), Bin, Ci, Da, Htl, Mad, Tin

Delta, Dpp, En, Hh, Med, Pyr, Shn, Su\_H\_CSL, Ths, Bap(3), Bin, Ci, Da, Htl, Mad, Tin, Twi, Zfh-1, Eya

Delta, Dpp, Med, Pyr, Shn, Spi, Su\_H\_CSL, Ths, Upd, Wg, Da, Doc, Htl, Mad, Pan, Slp, Stat92E, Tin(2), Twi(2), Zfh-1, Eve, Eya, Hbr, Pnr

Delta, En, Hh, Med, Pyr, Su\_H\_CSL, Ths, Ci, Da, Htl, Brk, Srp(2)

Delta, En, Hh, Med, Pyr, Su\_H\_CSL, Ths, Ci, Da, Htl, Twi, Zfh-1, Brk, D-six4, Eya, Srp(2)

Med, Pyr, Su\_H\_CSL, Ths, Wg, Da, Htl, Pan, Slp, Twi(2), Zfh-1, Brk, D-six4, Eya, Hbr, Poxm

### Genotype: Mef2\_0; Nicd\_1

Delta, Dpp, En, Hh, Med, Pyr, Shn, Su\_H\_CSL, Ths, Bap(3), Bin, Ci, Htl, Mad, Nicd, Tin

Delta, Dpp, Med, Pyr, Shn, Spi, Su\_H\_CSL, Ths, Upd, Wg, Doc, E\_Spl, Htl, Mad, Nicd, Pan, Slp, Stat92E, Tin(2), Twi, Zfh-1, Eve, Eya, Hbr, Pnr

Delta, En, Hh, Med, Pyr, Su\_H\_CSL, Ths, Ci, Htl, Nicd, Brk, Srp(2)

Med, Pyr, Su\_H\_CSL, Ths, Wg, Htl, Nicd, Pan, Slp, Twi, Zfh-1, Brk, D-six4, Eya, Hbr, Poxm

### Genotype: Doc\_1; Srp\_0

Delta, Dpp, En, Hh, Med, Pyr, Shn, Su\_H\_CSL, Ths, Bap(3), Bin, Ci, Doc, Htl, Mad, Nicd, Tin, Mef2, Pnr

Delta, Dpp, Med, Pyr, Shn, Spi, Su\_H\_CSL, Ths, Upd, Wg, Doc, E\_Spl, Htl, Mad, Nicd, Pan, Slp, Stat92E, Tin(2), Twi, Zfh-1, Eve, Eya, Hbr, Mef2, Pnr

Delta, En, Hh, Med, Pyr, Su\_H\_CSL, Ths, Ci, Doc, Htl, Nicd, Brk

Med, Pyr, Su\_H\_CSL, Ths, Wg, Da, Doc, Htl, Pan, Slp, Twi(2), Zfh-1, Brk, D-six4, Eya, Hbr, Mef2, Poxm

### Genotype: Bap\_0; Med\_1

Delta, Dpp, En, Hh, Med, Pyr, Shn, Su\_H\_CSL, Ths, Ci, Htl, Mad, Nicd, Tin, Mef2

Delta, Dpp, Med, Pyr, Shn, Spi, Su\_H\_CSL, Ths, Upd, Wg, Doc, E\_Spl, Htl, Mad, Nicd, Pan, Slp, Stat92E, Tin(2), Twi, Zfh-1, Eve, Eya, Hbr, Mef2, Pnr

Delta, En, Hh, Med, Pyr, Su\_H\_CSL, Ths, Ci, Htl, Nicd, Brk, Srp(2)

Med, Pyr, Su\_H\_CSL, Ths, Wg, Da, Htl, Pan, Slp, Twi(2), Zfh-1, Brk, D-six4, Eya, Hbr, Mef2, Poxm

### Genotype: Ci\_0; Srp\_0

Delta, Dpp, En, Hh, Med, Pyr, Shn, Su\_H\_CSL, Ths, Bap, Htl, Mad, Nicd, Tin, Mef2

Delta, Dpp, En, Hh, Med, Pyr, Shn, Su\_H\_CSL, Ths, Bap(2), Bin, Htl, Mad, Nicd, Tin, Mef2

Delta, Dpp, Med, Pyr, Shn, Spi, Su\_H\_CSL, Ths, Upd, Wg, Doc, E\_Spl, Htl, Mad, Nicd, Pan, Slp, Stat92E, Tin(2), Twi, Zfh-1, Eve, Eya, Hbr, Mef2, Pnr

Delta, En, Hh, Med, Pyr, Su\_H\_CSL, Ths, Htl, Nicd, Brk

Med, Pyr, Su\_H\_CSL, Ths, Wg, Da, Htl, Pan, Slp, Twi(2), Zfh-1, Brk, D-six4, Eya, Hbr, Mef2, Poxm

### Genotype: Bap\_0; Med\_0

Delta, Dpp, En, Hh, Pyr, Shn, Su\_H\_CSL, Ths, Ci, Htl, Mad, Nicd, Tin, Brk, D-six4, Mef2, Srp(2)

Delta, Dpp, Pyr, Shn, Spi, Su\_H\_CSL, Ths, Upd, Wg, E\_Spl, Htl, Mad, Nicd, Pan, Slp, Stat92E, Tin, Twi, Zfh-1, Brk, D-six4, Eya, Hbr, Mef2, Poxm

Delta, En, Hh, Pyr, Su\_H\_CSL, Ths, Ci, Htl, Nicd, Brk, Srp(2)

Pyr, Su\_H\_CSL, Ths, Wg, Da, Htl, Pan, Slp, Twi(2), Zfh-1, Brk, D-six4, Eya, Hbr, Mef2, Poxm

### Genotype: Bap\_0; Nicd\_1

Delta, Dpp, En, Hh, Med, Pyr, Shn, Su\_H\_CSL, Ths, Ci, Htl, Mad, Nicd, Tin, Mef2

Delta, Dpp, Med, Pyr, Shn, Spi, Su\_H\_CSL, Ths, Upd, Wg, Doc, E\_Spl, Htl, Mad, Nicd, Pan, Slp, Stat92E, Tin(2), Twi, Zfh-1, Eve, Eya, Hbr, Mef2, Pnr

Delta, En, Hh, Med, Pyr, Su\_H\_CSL, Ths, Ci, Htl, Nicd, Brk, Srp(2)

Med, Pyr, Su\_H\_CSL, Ths, Wg, Htl, Nicd, Pan, Slp, Twi, Zfh-1, Brk, D-six4, Eya, Hbr, Poxm

### Genotype: Mef2\_0; Tin\_2

Delta, Dpp, En, Hh, Med, Pyr, Shn, Su\_H\_CSL, Ths, Bap(3), Bin, Ci, E\_Spl, Htl, Mad, Nicd, Stat92E, Tin(2), Eya

Delta, Dpp, Med, Pyr, Shn, Spi, Su\_H\_CSL, Ths, Upd, Wg, Doc, E\_Spl, Htl, Mad, Nicd, Pan, Slp, Stat92E, Tin(2), Twi, Zfh-1, Eve, Eya, Hbr, Pnr

Delta, En, Hh, Med, Pyr, Su\_H\_CSL, Ths, Bap(2), Bin, Ci, E\_Spl, Htl, Nicd, Stat92E, Tin(2), Brk, D-six4, Eya, Srp(2)

Med, Pyr, Su\_H\_CSL, Ths, Wg, Da, Htl, Pan, Slp, Stat92E, Tin(2), Twi(2), Zfh-1, Brk, D-six4, Eya, Hbr, Poxm

### Genotype: Mef2\_0; Tin\_0

Delta, Dpp, En, Hh, Med, Pyr, Shn, Su\_H\_CSL, Ths, Ci, Htl, Mad, Nicd

Delta, Dpp, Med, Pyr, Shn, Spi, Su\_H\_CSL, Ths, Upd, Wg, Doc, E\_Spl, Htl, Mad, Nicd, Pan, Slp, Stat92E, Twi, Zfh-1, Eya, Hbr

Delta, En, Hh, Med, Pyr, Su\_H\_CSL, Ths, Ci, Htl, Nicd, Brk, Srp(2)

Med, Pyr, Su\_H\_CSL, Ths, Wg, Da, Htl, Pan, Slp, Twi(2), Zfh-1, Brk, D-six4, Eya, Hbr, Poxm

### Genotype: Bap\_0; Ci\_0

Delta, Dpp, En, Hh, Med, Pyr, Shn, Su\_H\_CSL, Ths, Htl, Mad, Nicd, Tin, Mef2

Delta, Dpp, Med, Pyr, Shn, Spi, Su\_H\_CSL, Ths, Upd, Wg, Doc, E\_Spl, Htl, Mad, Nicd, Pan, Slp, Stat92E, Tin(2), Twi, Zfh-1, Eve, Eya, Hbr, Mef2, Pnr

Delta, En, Hh, Med, Pyr, Su\_H\_CSL, Ths, Htl, Nicd, Brk

Med, Pyr, Su\_H\_CSL, Ths, Wg, Da, Htl, Pan, Slp, Twi(2), Zfh-1, Brk, D-six4, Eya, Hbr, Mef2, Poxm

### Genotype: Bap\_0; Ci\_1

Delta, Dpp, En, Hh, Med, Pyr, Shn, Su\_H\_CSL, Ths, Ci, Htl, Mad, Nicd, Tin, Mef2

Delta, Dpp, Med, Pyr, Shn, Spi, Su\_H\_CSL, Ths, Upd, Wg, Ci, Doc, E\_Spl, Htl, Mad, Nicd, Pan, Slp, Stat92E, Tin(2), Twi, Zfh-1, Eve, Eya, Hbr, Mef2, Pnr

Delta, En, Hh, Med, Pyr, Su\_H\_CSL, Ths, Ci, Htl, Nicd, Brk, Srp(2)

Med, Pyr, Su\_H\_CSL, Ths, Wg, Ci, Da, Htl, Pan, Slp, Twi(2), Zfh-1, Brk, D-six4, Eya, Hbr, Mef2, Poxm, Srp

### Genotype: Med\_1; Nicd\_0

Delta, Dpp, En, Hh, Med, Pyr, Shn, Su\_H\_CSL, Ths, Bap(3), Bin, Ci, Da, Htl, Mad, Tin, Mef2

Delta, Dpp, En, Hh, Med, Pyr, Shn, Su\_H\_CSL, Ths, Bap(3), Bin, Ci, Da, Htl, Mad, Tin, Twi, Zfh-1, Eya, Mef2

Delta, Dpp, Med, Pyr, Shn, Spi, Su\_H\_CSL, Ths, Upd, Wg, Da, Doc, Htl, Mad, Pan, Slp, Stat92E, Tin(2), Twi(2), Zfh-1, Eve, Eya, Hbr, Mef2, Pnr

Delta, En, Hh, Med, Pyr, Su\_H\_CSL, Ths, Ci, Da, Htl, Brk, Srp(2)

Delta, En, Hh, Med, Pyr, Su\_H\_CSL, Ths, Ci, Da, Htl, Twi, Zfh-1, Brk, D-six4, Eya, Srp(2)

Med, Pyr, Su\_H\_CSL, Ths, Wg, Da, Htl, Pan, Slp, Twi(2), Zfh-1, Brk, D-six4, Eya, Hbr, Mef2, Poxm

### Genotype: Med\_1; Nicd\_1

Delta, Dpp, En, Hh, Med, Pyr, Shn, Su\_H\_CSL, Ths, Bap(3), Bin, Ci, Htl, Mad, Nicd, Tin, Mef2

Delta, Dpp, Med, Pyr, Shn, Spi, Su\_H\_CSL, Ths, Upd, Wg, Doc, E\_Spl, Htl, Mad, Nicd, Pan, Slp, Stat92E, Tin(2), Twi, Zfh-1, Eve, Eya, Hbr, Mef2, Pnr

Delta, En, Hh, Med, Pyr, Su\_H\_CSL, Ths, Ci, Htl, Nicd, Brk, Srp(2)

Med, Pyr, Su\_H\_CSL, Ths, Wg, Htl, Nicd, Pan, Slp, Twi, Zfh-1, Brk, D-six4, Eya, Hbr, Poxm

### Genotype: Bap\_0; Nicd\_0

Delta, Dpp, En, Hh, Med, Pyr, Shn, Su\_H\_CSL, Ths, Ci, Da, Htl, Mad, Tin, Mef2

Delta, Dpp, En, Hh, Med, Pyr, Shn, Su\_H\_CSL, Ths, Ci, Da, Htl, Mad, Tin, Twi, Zfh-1, Eya, Mef2

Delta, Dpp, Med, Pyr, Shn, Spi, Su\_H\_CSL, Ths, Upd, Wg, Da, Doc, Htl, Mad, Pan, Slp, Stat92E, Tin(2), Twi(2), Zfh-1, Eve, Eya, Hbr, Mef2, Pnr

Delta, En, Hh, Med, Pyr, Su\_H\_CSL, Ths, Ci, Da, Htl, Brk, Srp(2)

Delta, En, Hh, Med, Pyr, Su\_H\_CSL, Ths, Ci, Da, Htl, Twi, Zfh-1, Brk, D-six4, Eya, Srp(2)

Med, Pyr, Su\_H\_CSL, Ths, Wg, Da, Htl, Pan, Slp, Twi(2), Zfh-1, Brk, D-six4, Eya, Hbr, Mef2, Poxm

### Genotype: Med\_0; Tin\_0

Delta, Dpp, En, Hh, Pyr, Shn, Su\_H\_CSL, Ths, Ci, Htl, Mad, Nicd, Brk, Srp(2)

Delta, Dpp, Pyr, Shn, Spi, Su\_H\_CSL, Ths, Upd, Wg, E\_Spl, Htl, Mad, Nicd, Pan, Slp, Stat92E, Twi, Zfh-1, Brk, D-six4, Eya, Hbr, Poxm

Delta, En, Hh, Pyr, Su\_H\_CSL, Ths, Ci, Htl, Nicd, Brk, Srp(2)

Pyr, Su\_H\_CSL, Ths, Wg, Da, Htl, Pan, Slp, Twi(2), Zfh-1, Brk, D-six4, Eya, Hbr, Mef2, Poxm

### Genotype: Med\_0; Tin\_2

Delta, Dpp, En, Hh, Pyr, Shn, Su\_H\_CSL, Ths, Bap(2), Bin, Ci, E\_Spl, Htl, Mad, Nicd, Stat92E, Tin(2), Brk, D-six4, Eya, Mef2, Srp(2)

Delta, Dpp, Pyr, Shn, Spi, Su\_H\_CSL, Ths, Upd, Wg, E\_Spl, Htl, Mad, Nicd, Pan, Slp, Stat92E, Tin(2), Twi, Zfh-1, Brk, D-six4, Eya, Hbr, Mef2, Poxm

Delta, En, Hh, Pyr, Su\_H\_CSL, Ths, Bap(2), Bin, Ci, E\_Spl, Htl, Nicd, Stat92E, Tin(2), Brk, D-six4, Eya, Mef2, Srp(2)

Pyr, Su\_H\_CSL, Ths, Wg, Da, Htl, Pan, Slp, Stat92E, Tin(2), Twi(2), Zfh-1, Brk, D-six4, Eya, Hbr, Mef2, Poxm

### Genotype: Slp\_1

Delta, Dpp, Hh, Med, Pyr, Shn, Su\_H\_CSL, Ths, Bap, Ci, Htl, Mad, Nicd, Slp, Tin, Mef2

Delta, Dpp, Hh, Med, Pyr, Shn, Su\_H\_CSL, Ths, Bap, Ci, Htl, Mad, Nicd, Slp, Tin, Twi, Zfh-1, Eya, Mef2

Delta, Dpp, Med, Pyr, Shn, Spi, Su\_H\_CSL, Ths, Upd, Wg, Doc, E\_Spl, Htl, Mad, Nicd, Pan, Slp, Stat92E, Tin(2), Twi, Zfh-1, Eve, Eya, Hbr, Mef2, Pnr

Delta, Hh, Med, Pyr, Su\_H\_CSL, Ths, Ci, Htl, Nicd, Slp, Brk, Srp

Delta, Hh, Med, Pyr, Su\_H\_CSL, Ths, Ci, Htl, Nicd, Slp, Twi, Zfh-1, Brk, D-six4, Eya, Srp

Med, Pyr, Su\_H\_CSL, Ths, Wg, Da, Htl, Pan, Slp, Twi(2), Zfh-1, Brk, D-six4, Eya, Hbr, Mef2, Poxm

### Genotype: Pnr\_0

Delta, Dpp, En, Hh, Med, Pyr, Shn, Su\_H\_CSL, Ths, Bap(3), Bin, Ci, Htl, Mad, Nicd, Tin, Mef2

Delta, Dpp, Med, Pyr, Shn, Spi, Su\_H\_CSL, Ths, Upd, Wg, Doc, E\_Spl, Htl, Mad, Nicd, Pan, Slp, Stat92E, Tin(2), Twi, Zfh-1, Eve, Eya, Hbr, Mef2

Delta, En, Hh, Med, Pyr, Su\_H\_CSL, Ths, Ci, Htl, Nicd, Brk, Srp(2)

Med, Pyr, Su\_H\_CSL, Ths, Wg, Da, Htl, Pan, Slp, Twi(2), Zfh-1, Brk, D-six4, Eya, Hbr, Mef2, Poxm

### Genotype: Ci\_0; Twi\_2

Delta, Dpp, En, Hh, Med, Pyr, Shn, Su\_H\_CSL, Ths, Bap, Htl, Mad, Nicd, Tin, Twi(2), Zfh-1, Eya, Mef2

Delta, Dpp, En, Hh, Med, Pyr, Shn, Su\_H\_CSL, Ths, Bap(2), Bin, Htl, Mad, Nicd, Tin, Twi(2), Zfh-1, Eya, Mef2

Delta, Dpp, Med, Pyr, Shn, Spi, Su\_H\_CSL, Ths, Upd, Wg, Doc, E\_Spl, Htl, Mad, Nicd, Pan, Slp, Stat92E, Tin(2), Twi(2), Zfh-1, Eve, Eya, Hbr, Mef2, Pnr

Delta, En, Hh, Med, Pyr, Su\_H\_CSL, Ths, Htl, Nicd, Twi(2), Zfh-1, Brk, D-six4, Eya, Mef2

Med, Pyr, Su\_H\_CSL, Ths, Wg, Da, Htl, Pan, Slp, Twi(2), Zfh-1, Brk, D-six4, Eya, Hbr, Mef2, Poxm

### Genotype: Ci\_0; Twi\_0

Delta, Dpp, En, Hh, Med, Pyr, Shn, Su\_H\_CSL, Ths, Bap, Htl, Mad, Nicd, Tin, Mef2

Delta, Dpp, En, Hh, Med, Pyr, Shn, Su\_H\_CSL, Ths, Bap(2), Bin, Htl, Mad, Nicd, Tin, Mef2

Delta, Dpp, Med, Pyr, Shn, Spi, Su\_H\_CSL, Ths, Upd, Wg, Doc, E\_Spl, Htl, Mad, Nicd, Pan, Slp, Stat92E, Tin(2), Eya, Hbr, Mef2, Pnr

Delta, En, Hh, Med, Pyr, Su\_H\_CSL, Ths, Htl, Nicd, Brk

Med, Pyr, Su\_H\_CSL, Ths, Wg, Da, Htl, Pan, Slp, Brk, Hbr

### Genotype: Nicd\_1; Slp\_1

Delta, Dpp, Hh, Med, Pyr, Shn, Su\_H\_CSL, Ths, Bap, Ci, Htl, Mad, Nicd, Slp, Tin, Mef2

Delta, Dpp, Hh, Med, Pyr, Shn, Su\_H\_CSL, Ths, Bap, Ci, Htl, Mad, Nicd, Slp, Tin, Twi, Zfh-1, Eya, Mef2

Delta, Dpp, Med, Pyr, Shn, Spi, Su\_H\_CSL, Ths, Upd, Wg, Doc, E\_Spl, Htl, Mad, Nicd, Pan, Slp, Stat92E, Tin(2), Twi, Zfh-1, Eve, Eya, Hbr, Mef2, Pnr

Delta, Hh, Med, Pyr, Su\_H\_CSL, Ths, Ci, Htl, Nicd, Slp, Brk, Srp

Delta, Hh, Med, Pyr, Su\_H\_CSL, Ths, Ci, Htl, Nicd, Slp, Twi, Zfh-1, Brk, D-six4, Eya, Srp

Med, Pyr, Su\_H\_CSL, Ths, Wg, Htl, Nicd, Pan, Slp, Twi, Zfh-1, Brk, D-six4, Eya, Hbr, Poxm

### Genotype: Nicd\_1; Slp\_0

Delta, Dpp, En, Hh, Med, Pyr, Shn, Su\_H\_CSL, Ths, Bap(3), Bin, Ci, Htl, Mad, Nicd, Tin, Mef2

Delta, Dpp, En, Med, Pyr, Shn, Spi, Su\_H\_CSL, Ths, Upd, Wg, Bap, Doc, E\_Spl, Htl, Mad, Nicd, Pan, Stat92E, Tin(2), Eya, Hbr, Mef2, Pnr

Delta, En, Hh, Med, Pyr, Su\_H\_CSL, Ths, Ci, Htl, Nicd, Brk, Srp(2)

En, Med, Pyr, Su\_H\_CSL, Ths, Wg, Htl, Nicd, Pan, Brk, Hbr

### Genotype: Ci\_0; Doc\_1

Delta, Dpp, En, Hh, Med, Pyr, Shn, Su\_H\_CSL, Ths, Bap, Doc, Htl, Mad, Nicd, Tin, Mef2, Pnr

Delta, Dpp, En, Hh, Med, Pyr, Shn, Su\_H\_CSL, Ths, Bap(2), Bin, Doc, Htl, Mad, Nicd, Tin, Mef2, Pnr

Delta, Dpp, Med, Pyr, Shn, Spi, Su\_H\_CSL, Ths, Upd, Wg, Doc, E\_Spl, Htl, Mad, Nicd, Pan, Slp, Stat92E, Tin(2), Twi, Zfh-1, Eve, Eya, Hbr, Mef2, Pnr

Delta, En, Hh, Med, Pyr, Su\_H\_CSL, Ths, Doc, Htl, Nicd, Brk

Med, Pyr, Su\_H\_CSL, Ths, Wg, Da, Doc, Htl, Pan, Slp, Twi(2), Zfh-1, Brk, D-six4, Eya, Hbr, Mef2, Poxm

### Genotype: Ci\_0; Doc\_0

Delta, Dpp, En, Hh, Med, Pyr, Shn, Su\_H\_CSL, Ths, Bap, Htl, Mad, Nicd, Tin, Mef2

Delta, Dpp, En, Hh, Med, Pyr, Shn, Su\_H\_CSL, Ths, Bap(2), Bin, Htl, Mad, Nicd, Tin, Mef2

Delta, Dpp, Med, Pyr, Shn, Spi, Su\_H\_CSL, Ths, Upd, Wg, E\_Spl, Htl, Mad, Nicd, Pan, Slp, Stat92E, Tin(2), Twi, Zfh-1, Eve, Eya, Hbr, Mef2

Delta, En, Hh, Med, Pyr, Su\_H\_CSL, Ths, Htl, Nicd, Brk

Med, Pyr, Su\_H\_CSL, Ths, Wg, Da, Htl, Pan, Slp, Twi(2), Zfh-1, Brk, D-six4, Eya, Hbr, Mef2, Poxm

### Genotype: Tin\_0; Twi\_2

Delta, Dpp, En, Hh, Med, Pyr, Shn, Su\_H\_CSL, Ths, Ci, Htl, Mad, Nicd, Twi(2), Zfh-1, Eya, Mef2

Delta, Dpp, Med, Pyr, Shn, Spi, Su\_H\_CSL, Ths, Upd, Wg, Doc, E\_Spl, Htl, Mad, Nicd, Pan, Slp, Stat92E, Twi(2), Zfh-1, Eya, Hbr, Mef2

Delta, En, Hh, Med, Pyr, Su\_H\_CSL, Ths, Ci, Htl, Nicd, Twi(2), Zfh-1, Brk, D-six4, Eya, Mef2, Srp(2)

Med, Pyr, Su\_H\_CSL, Ths, Wg, Da, Htl, Pan, Slp, Twi(2), Zfh-1, Brk, D-six4, Eya, Hbr, Mef2, Poxm

### Genotype: Tin\_0; Twi\_0

Delta, Dpp, En, Hh, Med, Pyr, Shn, Su\_H\_CSL, Ths, Ci, Htl, Mad, Nicd

Delta, Dpp, Med, Pyr, Shn, Spi, Su\_H\_CSL, Ths, Upd, Wg, Doc, E\_Spl, Htl, Mad, Nicd, Pan, Slp, Stat92E, Hbr

Delta, En, Hh, Med, Pyr, Su\_H\_CSL, Ths, Ci, Htl, Nicd, Brk, Srp(2)

Med, Pyr, Su\_H\_CSL, Ths, Wg, Da, Htl, Pan, Slp, Brk, Hbr

### Genotype: Mad\_1; Med\_1

Delta, Dpp, En, Hh, Med, Pyr, Shn, Su\_H\_CSL, Ths, Bap(3), Bin, Ci, Htl, Mad, Nicd, Tin, Mef2

Delta, Dpp, Med, Pyr, Shn, Spi, Su\_H\_CSL, Ths, Upd, Wg, Doc, E\_Spl, Htl, Mad, Nicd, Pan, Slp, Stat92E, Tin(2), Twi, Zfh-1, Eve, Eya, Hbr, Mef2, Pnr

Delta, En, Hh, Med, Pyr, Su\_H\_CSL, Ths, Bap(3), Bin, Ci, Htl, Mad, Nicd, Tin, Brk, Mef2

Med, Pyr, Su\_H\_CSL, Ths, Wg, Da, Doc, Htl, Mad, Pan, Slp, Tin, Twi(2), Zfh-1, Brk, Eya, Hbr, Mef2, Pnr

### Genotype: Mad\_1; Med\_0

Delta, Dpp, En, Hh, Pyr, Shn, Su\_H\_CSL, Ths, Bap(2), Bin, Ci, Htl, Mad, Nicd, Tin, Brk, D-six4, Mef2, Srp(2)

Delta, Dpp, Pyr, Shn, Spi, Su\_H\_CSL, Ths, Upd, Wg, E\_Spl, Htl, Mad, Nicd, Pan, Slp, Stat92E, Tin, Twi, Zfh-1, Brk, D-six4, Eya, Hbr, Mef2, Poxm

Delta, En, Hh, Pyr, Su\_H\_CSL, Ths, Bap(2), Bin, Ci, Htl, Mad, Nicd, Tin, Brk, D-six4, Mef2, Srp(2)

Delta, En, Hh, Pyr, Su\_H\_CSL, Ths, Ci, Htl, Mad, Nicd, Brk, Srp(2)

Pyr, Su\_H\_CSL, Ths, Wg, Da, Htl, Mad, Pan, Slp, Tin, Twi(2), Zfh-1, Brk, D-six4, Eya, Hbr, Mef2, Poxm

### Genotype: Mad\_1; Twi\_0

Delta, Dpp, En, Hh, Med, Pyr, Shn, Su\_H\_CSL, Ths, Bap(3), Bin, Ci, Htl, Mad, Nicd, Tin, Mef2

Delta, Dpp, Med, Pyr, Shn, Spi, Su\_H\_CSL, Ths, Upd, Wg, Doc, E\_Spl, Htl, Mad, Nicd, Pan, Slp, Stat92E, Tin(2), Eya, Hbr, Mef2, Pnr

Delta, En, Hh, Med, Pyr, Su\_H\_CSL, Ths, Bap(3), Bin, Ci, Htl, Mad, Nicd, Tin, Brk, Mef2

Med, Pyr, Su\_H\_CSL, Ths, Wg, Da, Doc, Htl, Mad, Pan, Slp, Tin, Brk, Hbr, Mef2, Pnr

### Genotype: Twi\_2

Delta, Dpp, En, Hh, Med, Pyr, Shn, Su\_H\_CSL, Ths, Bap(3), Bin, Ci, Htl, Mad, Nicd, Tin, Twi(2), Zfh-1, Eya, Mef2

Delta, Dpp, Med, Pyr, Shn, Spi, Su\_H\_CSL, Ths, Upd, Wg, Doc, E\_Spl, Htl, Mad, Nicd, Pan, Slp, Stat92E, Tin(2), Twi(2), Zfh-1, Eve, Eya, Hbr, Mef2, Pnr

Delta, En, Hh, Med, Pyr, Su\_H\_CSL, Ths, Ci, Htl, Nicd, Twi(2), Zfh-1, Brk, D-six4, Eya, Mef2, Srp(2)

Med, Pyr, Su\_H\_CSL, Ths, Wg, Da, Htl, Pan, Slp, Twi(2), Zfh-1, Brk, D-six4, Eya, Hbr, Mef2, Poxm

### Genotype: Mad\_1; Twi\_2

Delta, Dpp, En, Hh, Med, Pyr, Shn, Su\_H\_CSL, Ths, Bap(3), Bin, Ci, Htl, Mad, Nicd, Tin, Twi(2), Zfh-1, Eya, Mef2

Delta, Dpp, Med, Pyr, Shn, Spi, Su\_H\_CSL, Ths, Upd, Wg, Doc, E\_Spl, Htl, Mad, Nicd, Pan, Slp, Stat92E, Tin(2), Twi(2), Zfh-1, Eve, Eya, Hbr, Mef2, Pnr

Delta, En, Hh, Med, Pyr, Su\_H\_CSL, Ths, Bap(3), Bin, Ci, Htl, Mad, Nicd, Tin, Twi(2), Zfh-1, Brk, Eya, Mef2

Med, Pyr, Su\_H\_CSL, Ths, Wg, Da, Doc, Htl, Mad, Pan, Slp, Tin, Twi(2), Zfh-1, Brk, Eya, Hbr, Mef2, Pnr

### Genotype: Ci\_1; Pan\_1

Delta, Dpp, En, Hh, Med, Pyr, Shn, Su\_H\_CSL, Ths, Bap, Ci, Doc, Htl, Mad, Nicd, Pan, Slp, Tin, Hbr, Mef2, Pnr

Delta, Dpp, En, Hh, Med, Pyr, Shn, Su\_H\_CSL, Ths, Bap, Ci, Doc, Htl, Mad, Nicd, Pan, Slp, Tin, Twi, Zfh-1, Eya, Hbr, Mef2, Pnr

Delta, Dpp, Med, Pyr, Shn, Spi, Su\_H\_CSL, Ths, Upd, Wg, Bap, Ci, Doc, E\_Spl, Htl, Mad, Nicd, Pan, Slp, Stat92E, Tin(2), Twi, Zfh-1, Eve, Eya, Hbr, Mef2, Pnr

Delta, En, Hh, Med, Pyr, Su\_H\_CSL, Ths, Ci, Htl, Nicd, Pan, Slp, Brk, Hbr, Srp(2)

Delta, En, Hh, Med, Pyr, Su\_H\_CSL, Ths, Ci, Htl, Nicd, Pan, Slp, Twi, Zfh-1, Brk, D-six4, Eya, Hbr, Poxm, Srp(2)

Med, Pyr, Su\_H\_CSL, Ths, Wg, Ci, Da, Htl, Pan, Slp, Twi(2), Zfh-1, Brk, D-six4, Eya, Hbr, Mef2, Poxm, Srp

### Genotype: Ci\_1; Pan\_0

Delta, Dpp, En, Hh, Med, Pyr, Shn, Su\_H\_CSL, Ths, Bap(3), Bin, Ci, Htl, Mad, Nicd, Tin, Mef2

Delta, Dpp, Med, Pyr, Shn, Spi, Su\_H\_CSL, Ths, Upd, Wg, Bap, Ci, E\_Spl, Htl, Mad, Nicd, Stat92E, Tin, Mef2

Delta, En, Hh, Med, Pyr, Su\_H\_CSL, Ths, Ci, Htl, Nicd, Brk, Srp(2)

Med, Pyr, Su\_H\_CSL, Ths, Wg, Ci, Da, Htl, Brk, Srp

Med, Pyr, Su\_H\_CSL, Ths, Wg, Ci, Da, Htl, Twi, Zfh-1, Brk, D-six4, Eya, Srp

### Genotype: Bap\_3; Tin\_0

Delta, Dpp, En, Hh, Med, Pyr, Shn, Su\_H\_CSL, Ths, Bap(3), Bin, Ci, Htl, Mad, Nicd

Delta, Dpp, Med, Pyr, Shn, Spi, Su\_H\_CSL, Ths, Upd, Wg, Bap(3), Bin, Doc, E\_Spl, Htl, Mad, Nicd, Pan, Slp, Stat92E, Twi, Zfh-1, Eya, Hbr

Delta, En, Hh, Med, Pyr, Su\_H\_CSL, Ths, Bap(3), Bin, Ci, Htl, Nicd, Brk, Srp(2)

Med, Pyr, Su\_H\_CSL, Ths, Wg, Bap(3), Bin, Da, Htl, Pan, Slp, Twi(2), Zfh-1, Brk, D-six4, Eya, Hbr, Mef2, Poxm

### Genotype: Nicd\_0; Pnr\_1

Delta, Dpp, En, Hh, Med, Pyr, Shn, Su\_H\_CSL, Ths, Bap(3), Bin, Ci, Da, Htl, Mad, Tin, Mef2, Pnr

Delta, Dpp, En, Hh, Med, Pyr, Shn, Su\_H\_CSL, Ths, Bap(3), Bin, Ci, Da, Htl, Mad, Tin, Twi, Zfh-1, Eya, Mef2, Pnr

Delta, Dpp, Med, Pyr, Shn, Spi, Su\_H\_CSL, Ths, Upd, Wg, Da, Doc, Htl, Mad, Pan, Slp, Stat92E, Tin(2), Twi(2), Zfh-1, Eve, Eya, Hbr, Mef2, Pnr

Delta, En, Hh, Med, Pyr, Su\_H\_CSL, Ths, Ci, Da, Htl, Brk, Pnr, Srp(2)

Delta, En, Hh, Med, Pyr, Su\_H\_CSL, Ths, Ci, Da, Htl, Twi, Zfh-1, Brk, D-six4, Eya, Pnr, Srp(2)

Med, Pyr, Su\_H\_CSL, Ths, Wg, Da, Htl, Pan, Slp, Twi(2), Zfh-1, Brk, D-six4, Eya, Hbr, Mef2, Pnr, Poxm

### Genotype: Bap\_3; Tin\_2

Delta, Dpp, En, Hh, Med, Pyr, Shn, Su\_H\_CSL, Ths, Bap(3), Bin, Ci, E\_Spl, Htl, Mad, Nicd, Stat92E, Tin(2), Eya, Mef2

Delta, Dpp, Med, Pyr, Shn, Spi, Su\_H\_CSL, Ths, Upd, Wg, Bap(3), Bin, Doc, E\_Spl, Htl, Mad, Nicd, Pan, Slp, Stat92E, Tin(2), Twi, Zfh-1, Eve, Eya, Hbr, Mef2, Pnr

Delta, En, Hh, Med, Pyr, Su\_H\_CSL, Ths, Bap(3), Bin, Ci, E\_Spl, Htl, Nicd, Stat92E, Tin(2), Brk, D-six4, Eya, Mef2, Srp(2)

Med, Pyr, Su\_H\_CSL, Ths, Wg, Bap(3), Bin, Da, Htl, Pan, Slp, Stat92E, Tin(2), Twi(2), Zfh-1, Brk, D-six4, Eya, Hbr, Mef2, Poxm

### Genotype: Bin\_0; Doc\_1

Delta, Dpp, En, Hh, Med, Pyr, Shn, Su\_H\_CSL, Ths, Bap(3), Ci, Doc, Htl, Mad, Nicd, Tin, Mef2, Pnr

Delta, Dpp, Med, Pyr, Shn, Spi, Su\_H\_CSL, Ths, Upd, Wg, Doc, E\_Spl, Htl, Mad, Nicd, Pan, Slp, Stat92E, Tin(2), Twi, Zfh-1, Eve, Eya, Hbr, Mef2, Pnr

Delta, En, Hh, Med, Pyr, Su\_H\_CSL, Ths, Ci, Doc, Htl, Nicd, Brk, Srp(2)

Med, Pyr, Su\_H\_CSL, Ths, Wg, Da, Doc, Htl, Pan, Slp, Twi(2), Zfh-1, Brk, D-six4, Eya, Hbr, Mef2, Poxm

### Genotype: Bin\_0; Doc\_0

Delta, Dpp, En, Hh, Med, Pyr, Shn, Su\_H\_CSL, Ths, Bap(3), Ci, Htl, Mad, Nicd, Tin, Mef2

Delta, Dpp, Med, Pyr, Shn, Spi, Su\_H\_CSL, Ths, Upd, Wg, E\_Spl, Htl, Mad, Nicd, Pan, Slp, Stat92E, Tin(2), Twi, Zfh-1, Eve, Eya, Hbr, Mef2

Delta, En, Hh, Med, Pyr, Su\_H\_CSL, Ths, Ci, Htl, Nicd, Brk, Srp(2)

Med, Pyr, Su\_H\_CSL, Ths, Wg, Da, Htl, Pan, Slp, Twi(2), Zfh-1, Brk, D-six4, Eya, Hbr, Mef2, Poxm

### Genotype: Med\_1; Srp\_0

Delta, Dpp, En, Hh, Med, Pyr, Shn, Su\_H\_CSL, Ths, Bap(3), Bin, Ci, Htl, Mad, Nicd, Tin, Mef2

Delta, Dpp, Med, Pyr, Shn, Spi, Su\_H\_CSL, Ths, Upd, Wg, Doc, E\_Spl, Htl, Mad, Nicd, Pan, Slp, Stat92E, Tin(2), Twi, Zfh-1, Eve, Eya, Hbr, Mef2, Pnr

Delta, En, Hh, Med, Pyr, Su\_H\_CSL, Ths, Ci, Htl, Nicd, Brk

Med, Pyr, Su\_H\_CSL, Ths, Wg, Da, Htl, Pan, Slp, Twi(2), Zfh-1, Brk, D-six4, Eya, Hbr, Mef2, Poxm

### Genotype: Med\_1; Srp\_2

Delta, Dpp, En, Hh, Med, Pyr, Shn, Su\_H\_CSL, Ths, Bap(3), Bin, Ci, Htl, Mad, Nicd, Tin, Mef2, Srp(2)

Delta, Dpp, Med, Pyr, Shn, Spi, Su\_H\_CSL, Ths, Upd, Wg, Doc, E\_Spl, Htl, Mad, Nicd, Pan, Slp, Stat92E, Tin(2), Twi, Zfh-1, Eve, Eya, Hbr, Mef2, Pnr, Srp(2)

Delta, En, Hh, Med, Pyr, Su\_H\_CSL, Ths, Ci, Htl, Nicd, Brk, Srp(2)

Med, Pyr, Su\_H\_CSL, Ths, Wg, Da, Htl, Pan, Slp, Twi(2), Zfh-1, Brk, D-six4, Eya, Hbr, Mef2, Poxm, Srp(2)

### Genotype: Mad\_0; Med\_0

Delta, Dpp, En, Hh, Pyr, Shn, Su\_H\_CSL, Ths, Ci, Htl, Nicd, Brk, Srp(2)

Delta, Dpp, Pyr, Shn, Spi, Su\_H\_CSL, Ths, Upd, Wg, E\_Spl, Htl, Nicd, Pan, Slp, Stat92E, Twi, Zfh-1, Brk, D-six4, Eya, Hbr, Poxm

Delta, En, Hh, Pyr, Su\_H\_CSL, Ths, Ci, Htl, Nicd, Brk, Srp(2)

Pyr, Su\_H\_CSL, Ths, Wg, Da, Htl, Pan, Slp, Twi(2), Zfh-1, Brk, D-six4, Eya, Hbr, Mef2, Poxm

### Genotype: Mad\_0; Med\_1

Delta, Dpp, En, Hh, Med, Pyr, Shn, Su\_H\_CSL, Ths, Ci, Htl, Nicd, Brk, Srp(2)

Delta, Dpp, Med, Pyr, Shn, Spi, Su\_H\_CSL, Ths, Upd, Wg, E\_Spl, Htl, Nicd, Pan, Slp, Stat92E, Twi, Zfh-1, Brk, D-six4, Eya, Hbr, Poxm

Delta, En, Hh, Med, Pyr, Su\_H\_CSL, Ths, Ci, Htl, Nicd, Brk, Srp(2)

Med, Pyr, Su\_H\_CSL, Ths, Wg, Da, Htl, Pan, Slp, Twi(2), Zfh-1, Brk, D-six4, Eya, Hbr, Mef2, Poxm

### Genotype: Ci\_0; Nicd\_0

Delta, Dpp, En, Hh, Med, Pyr, Shn, Su\_H\_CSL, Ths, Bap, Da, Htl, Mad, Tin, Mef2

Delta, Dpp, En, Hh, Med, Pyr, Shn, Su\_H\_CSL, Ths, Bap(2), Bin, Da, Htl, Mad, Tin, Mef2

Delta, Dpp, En, Hh, Med, Pyr, Shn, Su\_H\_CSL, Ths, Bap, Da, Htl, Mad, Tin, Twi, Zfh-1, Eya, Mef2

Delta, Dpp, En, Hh, Med, Pyr, Shn, Su\_H\_CSL, Ths, Bap(2), Bin, Da, Htl, Mad, Tin, Twi, Zfh-1, Eya, Mef2

Delta, Dpp, Med, Pyr, Shn, Spi, Su\_H\_CSL, Ths, Upd, Wg, Da, Doc, Htl, Mad, Pan, Slp, Stat92E, Tin(2), Twi(2), Zfh-1, Eve, Eya, Hbr, Mef2, Pnr

Delta, En, Hh, Med, Pyr, Su\_H\_CSL, Ths, Da, Htl, Brk

Delta, En, Hh, Med, Pyr, Su\_H\_CSL, Ths, Da, Htl, Twi, Zfh-1, Brk, D-six4, Eya

Med, Pyr, Su\_H\_CSL, Ths, Wg, Da, Htl, Pan, Slp, Twi(2), Zfh-1, Brk, D-six4, Eya, Hbr, Mef2, Poxm

### Genotype: Ci\_0; Nicd\_1

Delta, Dpp, En, Hh, Med, Pyr, Shn, Su\_H\_CSL, Ths, Bap, Htl, Mad, Nicd, Tin, Mef2

Delta, Dpp, En, Hh, Med, Pyr, Shn, Su\_H\_CSL, Ths, Bap(2), Bin, Htl, Mad, Nicd, Tin, Mef2

Delta, Dpp, Med, Pyr, Shn, Spi, Su\_H\_CSL, Ths, Upd, Wg, Doc, E\_Spl, Htl, Mad, Nicd, Pan, Slp, Stat92E, Tin(2), Twi, Zfh-1, Eve, Eya, Hbr, Mef2, Pnr

Delta, En, Hh, Med, Pyr, Su\_H\_CSL, Ths, Htl, Nicd, Brk

Med, Pyr, Su\_H\_CSL, Ths, Wg, Htl, Nicd, Pan, Slp, Twi, Zfh-1, Brk, D-six4, Eya, Hbr, Poxm

### Genotype: Doc\_0

Delta, Dpp, En, Hh, Med, Pyr, Shn, Su\_H\_CSL, Ths, Bap(3), Bin, Ci, Htl, Mad, Nicd, Tin, Mef2

Delta, Dpp, Med, Pyr, Shn, Spi, Su\_H\_CSL, Ths, Upd, Wg, E\_Spl, Htl, Mad, Nicd, Pan, Slp, Stat92E, Tin(2), Twi, Zfh-1, Eve, Eya, Hbr, Mef2

Delta, En, Hh, Med, Pyr, Su\_H\_CSL, Ths, Ci, Htl, Nicd, Brk, Srp(2)

Med, Pyr, Su\_H\_CSL, Ths, Wg, Da, Htl, Pan, Slp, Twi(2), Zfh-1, Brk, D-six4, Eya, Hbr, Mef2, Poxm

### Genotype: Slp\_0; Tin\_0

Delta, Dpp, En, Hh, Med, Pyr, Shn, Su\_H\_CSL, Ths, Ci, Htl, Mad, Nicd

Delta, Dpp, En, Med, Pyr, Shn, Spi, Su\_H\_CSL, Ths, Upd, Wg, Doc, E\_Spl, Htl, Mad, Nicd, Pan, Stat92E, Hbr

Delta, En, Hh, Med, Pyr, Su\_H\_CSL, Ths, Ci, Htl, Nicd, Brk, Srp(2)

En, Med, Pyr, Su\_H\_CSL, Ths, Wg, Da, Htl, Pan, Brk, Hbr

En, Med, Pyr, Su\_H\_CSL, Ths, Wg, Da, Htl, Pan, Twi, Zfh-1, Brk, D-six4, Eya, Hbr, Poxm

### Genotype: Slp\_0; Tin\_2

Delta, Dpp, En, Hh, Med, Pyr, Shn, Su\_H\_CSL, Ths, Bap(3), Bin, Ci, E\_Spl, Htl, Mad, Nicd, Stat92E, Tin(2), Eya, Mef2

Delta, Dpp, En, Med, Pyr, Shn, Spi, Su\_H\_CSL, Ths, Upd, Wg, Bap, Doc, E\_Spl, Htl, Mad, Nicd, Pan, Stat92E, Tin(2), Eya, Hbr, Mef2, Pnr

Delta, En, Hh, Med, Pyr, Su\_H\_CSL, Ths, Bap(2), Bin, Ci, E\_Spl, Htl, Nicd, Stat92E, Tin(2), Brk, D-six4, Eya, Mef2, Srp(2)

En, Med, Pyr, Su\_H\_CSL, Ths, Wg, Da, Htl, Pan, Stat92E, Tin(2), Brk, D-six4, Eya, Hbr, Mef2

En, Med, Pyr, Su\_H\_CSL, Ths, Wg, Da, Htl, Pan, Stat92E, Tin(2), Twi, Zfh-1, Brk, D-six4, Eya, Hbr, Mef2, Poxm

### Genotype: Bin\_2; Pan\_0

Delta, Dpp, En, Hh, Med, Pyr, Shn, Su\_H\_CSL, Ths, Bap(3), Bin(2), Ci, Htl, Mad, Nicd, Tin, Mef2

Delta, Dpp, Med, Pyr, Shn, Spi, Su\_H\_CSL, Ths, Upd, Wg, Bap(3), Bin(2), E\_Spl, Htl, Mad, Nicd, Stat92E, Tin, Mef2

Delta, En, Hh, Med, Pyr, Su\_H\_CSL, Ths, Bap(3), Bin(2), Ci, Htl, Nicd, Brk, Srp(2)

Med, Pyr, Su\_H\_CSL, Ths, Wg, Bap(3), Bin(2), Da, Htl, Brk

Med, Pyr, Su\_H\_CSL, Ths, Wg, Bap(3), Bin(2), Da, Htl, Twi, Zfh-1, Brk, D-six4, Eya

### Genotype: Nicd\_1; Pnr\_1

Delta, Dpp, En, Hh, Med, Pyr, Shn, Su\_H\_CSL, Ths, Bap(3), Bin, Ci, Htl, Mad, Nicd, Tin, Mef2, Pnr

Delta, Dpp, Med, Pyr, Shn, Spi, Su\_H\_CSL, Ths, Upd, Wg, Doc, E\_Spl, Htl, Mad, Nicd, Pan, Slp, Stat92E, Tin(2), Twi, Zfh-1, Eve, Eya, Hbr, Mef2, Pnr

Delta, En, Hh, Med, Pyr, Su\_H\_CSL, Ths, Ci, Htl, Nicd, Brk, Pnr, Srp(2)

Med, Pyr, Su\_H\_CSL, Ths, Wg, Htl, Nicd, Pan, Slp, Twi, Zfh-1, Brk, D-six4, Eya, Hbr, Pnr, Poxm

### Genotype: Ci\_1; Mef2\_1

Delta, Dpp, En, Hh, Med, Pyr, Shn, Su\_H\_CSL, Ths, Bap(3), Bin, Ci, Htl, Mad, Nicd, Tin, Mef2

Delta, Dpp, Med, Pyr, Shn, Spi, Su\_H\_CSL, Ths, Upd, Wg, Bap, Ci, Doc, E\_Spl, Htl, Mad, Nicd, Pan, Slp, Stat92E, Tin(2), Twi, Zfh-1, Eve, Eya, Hbr, Mef2, Pnr

Delta, En, Hh, Med, Pyr, Su\_H\_CSL, Ths, Ci, Htl, Nicd, Brk, Mef2, Srp(2)

Med, Pyr, Su\_H\_CSL, Ths, Wg, Ci, Da, Htl, Pan, Slp, Twi(2), Zfh-1, Brk, D-six4, Eya, Hbr, Mef2, Poxm, Srp

### Genotype: Ci\_1; Mef2\_0

Delta, Dpp, En, Hh, Med, Pyr, Shn, Su\_H\_CSL, Ths, Bap(3), Bin, Ci, Htl, Mad, Nicd, Tin

Delta, Dpp, Med, Pyr, Shn, Spi, Su\_H\_CSL, Ths, Upd, Wg, Bap, Ci, Doc, E\_Spl, Htl, Mad, Nicd, Pan, Slp, Stat92E, Tin(2), Twi, Zfh-1, Eve, Eya, Hbr, Pnr

Delta, En, Hh, Med, Pyr, Su\_H\_CSL, Ths, Ci, Htl, Nicd, Brk, Srp(2)

Med, Pyr, Su\_H\_CSL, Ths, Wg, Ci, Da, Htl, Pan, Slp, Twi(2), Zfh-1, Brk, D-six4, Eya, Hbr, Poxm, Srp

### Genotype: Bap\_0; Doc\_1

Delta, Dpp, En, Hh, Med, Pyr, Shn, Su\_H\_CSL, Ths, Ci, Doc, Htl, Mad, Nicd, Tin, Mef2, Pnr

Delta, Dpp, Med, Pyr, Shn, Spi, Su\_H\_CSL, Ths, Upd, Wg, Doc, E\_Spl, Htl, Mad, Nicd, Pan, Slp, Stat92E, Tin(2), Twi, Zfh-1, Eve, Eya, Hbr, Mef2, Pnr

Delta, En, Hh, Med, Pyr, Su\_H\_CSL, Ths, Ci, Doc, Htl, Nicd, Brk, Srp(2)

Med, Pyr, Su\_H\_CSL, Ths, Wg, Da, Doc, Htl, Pan, Slp, Twi(2), Zfh-1, Brk, D-six4, Eya, Hbr, Mef2, Poxm

### Genotype: Bap\_0; Doc\_0

Delta, Dpp, En, Hh, Med, Pyr, Shn, Su\_H\_CSL, Ths, Ci, Htl, Mad, Nicd, Tin, Mef2

Delta, Dpp, Med, Pyr, Shn, Spi, Su\_H\_CSL, Ths, Upd, Wg, E\_Spl, Htl, Mad, Nicd, Pan, Slp, Stat92E, Tin(2), Twi, Zfh-1, Eve, Eya, Hbr, Mef2

Delta, En, Hh, Med, Pyr, Su\_H\_CSL, Ths, Ci, Htl, Nicd, Brk, Srp(2)

Med, Pyr, Su\_H\_CSL, Ths, Wg, Da, Htl, Pan, Slp, Twi(2), Zfh-1, Brk, D-six4, Eya, Hbr, Mef2, Poxm

### Genotype: Pnr\_0; Srp\_2

Delta, Dpp, En, Hh, Med, Pyr, Shn, Su\_H\_CSL, Ths, Bap(3), Bin, Ci, Htl, Mad, Nicd, Tin, Mef2, Srp(2)

Delta, Dpp, Med, Pyr, Shn, Spi, Su\_H\_CSL, Ths, Upd, Wg, Doc, E\_Spl, Htl, Mad, Nicd, Pan, Slp, Stat92E, Tin(2), Twi, Zfh-1, Eve, Eya, Hbr, Mef2, Srp(2)

Delta, En, Hh, Med, Pyr, Su\_H\_CSL, Ths, Ci, Htl, Nicd, Brk, Srp(2)

Med, Pyr, Su\_H\_CSL, Ths, Wg, Da, Htl, Pan, Slp, Twi(2), Zfh-1, Brk, D-six4, Eya, Hbr, Mef2, Poxm, Srp(2)

### Genotype: Pnr\_0; Srp\_0

Delta, Dpp, En, Hh, Med, Pyr, Shn, Su\_H\_CSL, Ths, Bap(3), Bin, Ci, Htl, Mad, Nicd, Tin, Mef2

Delta, Dpp, Med, Pyr, Shn, Spi, Su\_H\_CSL, Ths, Upd, Wg, Doc, E\_Spl, Htl, Mad, Nicd, Pan, Slp, Stat92E, Tin(2), Twi, Zfh-1, Eve, Eya, Hbr, Mef2

Delta, En, Hh, Med, Pyr, Su\_H\_CSL, Ths, Ci, Htl, Nicd, Brk

Med, Pyr, Su\_H\_CSL, Ths, Wg, Da, Htl, Pan, Slp, Twi(2), Zfh-1, Brk, D-six4, Eya, Hbr, Mef2, Poxm

### Genotype: Tin\_2; Twi\_0

Delta, Dpp, En, Hh, Med, Pyr, Shn, Su\_H\_CSL, Ths, Bap(3), Bin, Ci, E\_Spl, Htl, Mad, Nicd, Stat92E, Tin(2), Eya, Mef2

Delta, Dpp, Med, Pyr, Shn, Spi, Su\_H\_CSL, Ths, Upd, Wg, Doc, E\_Spl, Htl, Mad, Nicd, Pan, Slp, Stat92E, Tin(2), Eya, Hbr, Mef2, Pnr

Delta, En, Hh, Med, Pyr, Su\_H\_CSL, Ths, Bap(2), Bin, Ci, E\_Spl, Htl, Nicd, Stat92E, Tin(2), Brk, D-six4, Eya, Mef2, Srp(2)

Med, Pyr, Su\_H\_CSL, Ths, Wg, Da, Htl, Pan, Slp, Stat92E, Tin(2), Brk, D-six4, Eya, Hbr, Mef2

### Genotype: Tin\_2; Twi\_2

Delta, Dpp, En, Hh, Med, Pyr, Shn, Su\_H\_CSL, Ths, Bap(3), Bin, Ci, E\_Spl, Htl, Mad, Nicd, Stat92E, Tin(2), Twi(2), Zfh-1, Eya, Mef2

Delta, Dpp, Med, Pyr, Shn, Spi, Su\_H\_CSL, Ths, Upd, Wg, Doc, E\_Spl, Htl, Mad, Nicd, Pan, Slp, Stat92E, Tin(2), Twi(2), Zfh-1, Eve, Eya, Hbr, Mef2, Pnr

Delta, En, Hh, Med, Pyr, Su\_H\_CSL, Ths, Bap(2), Bin, Ci, E\_Spl, Htl, Nicd, Stat92E, Tin(2), Twi(2), Zfh-1, Brk, D-six4, Eya, Mef2, Srp(2)

Med, Pyr, Su\_H\_CSL, Ths, Wg, Da, Htl, Pan, Slp, Stat92E, Tin(2), Twi(2), Zfh-1, Brk, D-six4, Eya, Hbr, Mef2, Poxm

### Genotype: Ci\_1; Mad\_0

Delta, Dpp, En, Hh, Med, Pyr, Shn, Su\_H\_CSL, Ths, Ci, Htl, Nicd, Brk, Srp(2)

Delta, Dpp, Med, Pyr, Shn, Spi, Su\_H\_CSL, Ths, Upd, Wg, Ci, E\_Spl, Htl, Nicd, Pan, Slp, Stat92E, Twi, Zfh-1, Brk, D-six4, Eya, Hbr, Poxm, Srp

Delta, En, Hh, Med, Pyr, Su\_H\_CSL, Ths, Ci, Htl, Nicd, Brk, Srp(2)

Med, Pyr, Su\_H\_CSL, Ths, Wg, Ci, Da, Htl, Pan, Slp, Twi(2), Zfh-1, Brk, D-six4, Eya, Hbr, Mef2, Poxm, Srp

### Genotype: Bap\_3; Bin\_2

Delta, Dpp, En, Hh, Med, Pyr, Shn, Su\_H\_CSL, Ths, Bap(3), Bin(2), Ci, Htl, Mad, Nicd, Tin, Mef2

Delta, Dpp, Med, Pyr, Shn, Spi, Su\_H\_CSL, Ths, Upd, Wg, Bap(3), Bin(2), Doc, E\_Spl, Htl, Mad, Nicd, Pan, Slp, Stat92E, Tin(2), Twi, Zfh-1, Eve, Eya, Hbr, Mef2, Pnr

Delta, En, Hh, Med, Pyr, Su\_H\_CSL, Ths, Bap(3), Bin(2), Ci, Htl, Nicd, Brk, Srp(2)

Med, Pyr, Su\_H\_CSL, Ths, Wg, Bap(3), Bin(2), Da, Htl, Pan, Slp, Twi(2), Zfh-1, Brk, D-six4, Eya, Hbr, Mef2, Poxm

### Genotype: Bap\_0

Delta, Dpp, En, Hh, Med, Pyr, Shn, Su\_H\_CSL, Ths, Ci, Htl, Mad, Nicd, Tin, Mef2

Delta, Dpp, Med, Pyr, Shn, Spi, Su\_H\_CSL, Ths, Upd, Wg, Doc, E\_Spl, Htl, Mad, Nicd, Pan, Slp, Stat92E, Tin(2), Twi, Zfh-1, Eve, Eya, Hbr, Mef2, Pnr

Delta, En, Hh, Med, Pyr, Su\_H\_CSL, Ths, Ci, Htl, Nicd, Brk, Srp(2)

Med, Pyr, Su\_H\_CSL, Ths, Wg, Da, Htl, Pan, Slp, Twi(2), Zfh-1, Brk, D-six4, Eya, Hbr, Mef2, Poxm

### Genotype: Bap\_3; Bin\_0

Delta, Dpp, En, Hh, Med, Pyr, Shn, Su\_H\_CSL, Ths, Bap(3), Ci, Htl, Mad, Nicd, Tin, Mef2

Delta, Dpp, Med, Pyr, Shn, Spi, Su\_H\_CSL, Ths, Upd, Wg, Bap(3), Doc, E\_Spl, Htl, Mad, Nicd, Pan, Slp, Stat92E, Tin(2), Twi, Zfh-1, Eve, Eya, Hbr, Mef2, Pnr

Delta, En, Hh, Med, Pyr, Su\_H\_CSL, Ths, Bap(3), Ci, Htl, Nicd, Brk, Srp(2)

Med, Pyr, Su\_H\_CSL, Ths, Wg, Bap(3), Da, Htl, Pan, Slp, Twi(2), Zfh-1, Brk, D-six4, Eya, Hbr, Mef2, Poxm

### Genotype: Nicd\_1; Tin\_2

Delta, Dpp, En, Hh, Med, Pyr, Shn, Su\_H\_CSL, Ths, Bap(3), Bin, Ci, E\_Spl, Htl, Mad, Nicd, Stat92E, Tin(2), Eya, Mef2

Delta, Dpp, Med, Pyr, Shn, Spi, Su\_H\_CSL, Ths, Upd, Wg, Doc, E\_Spl, Htl, Mad, Nicd, Pan, Slp, Stat92E, Tin(2), Twi, Zfh-1, Eve, Eya, Hbr, Mef2, Pnr

Delta, En, Hh, Med, Pyr, Su\_H\_CSL, Ths, Bap(2), Bin, Ci, E\_Spl, Htl, Nicd, Stat92E, Tin(2), Brk, D-six4, Eya, Mef2, Srp(2)

Med, Pyr, Su\_H\_CSL, Ths, Wg, E\_Spl, Htl, Nicd, Pan, Slp, Stat92E, Tin(2), Twi, Zfh-1, Brk, D-six4, Eya, Hbr, Mef2, Poxm

### Genotype: Nicd\_1; Tin\_0

Delta, Dpp, En, Hh, Med, Pyr, Shn, Su\_H\_CSL, Ths, Ci, Htl, Mad, Nicd

Delta, Dpp, Med, Pyr, Shn, Spi, Su\_H\_CSL, Ths, Upd, Wg, Doc, E\_Spl, Htl, Mad, Nicd, Pan, Slp, Stat92E, Twi, Zfh-1, Eya, Hbr

Delta, En, Hh, Med, Pyr, Su\_H\_CSL, Ths, Ci, Htl, Nicd, Brk, Srp(2)

Med, Pyr, Su\_H\_CSL, Ths, Wg, Htl, Nicd, Pan, Slp, Twi, Zfh-1, Brk, D-six4, Eya, Hbr, Poxm

### Genotype: Med\_1; Mef2\_0

Delta, Dpp, En, Hh, Med, Pyr, Shn, Su\_H\_CSL, Ths, Bap(3), Bin, Ci, Htl, Mad, Nicd, Tin

Delta, Dpp, Med, Pyr, Shn, Spi, Su\_H\_CSL, Ths, Upd, Wg, Doc, E\_Spl, Htl, Mad, Nicd, Pan, Slp, Stat92E, Tin(2), Twi, Zfh-1, Eve, Eya, Hbr, Pnr

Delta, En, Hh, Med, Pyr, Su\_H\_CSL, Ths, Ci, Htl, Nicd, Brk, Srp(2)

Med, Pyr, Su\_H\_CSL, Ths, Wg, Da, Htl, Pan, Slp, Twi(2), Zfh-1, Brk, D-six4, Eya, Hbr, Poxm

### Genotype: Med\_1; Mef2\_1

Delta, Dpp, En, Hh, Med, Pyr, Shn, Su\_H\_CSL, Ths, Bap(3), Bin, Ci, Htl, Mad, Nicd, Tin, Mef2

Delta, Dpp, Med, Pyr, Shn, Spi, Su\_H\_CSL, Ths, Upd, Wg, Doc, E\_Spl, Htl, Mad, Nicd, Pan, Slp, Stat92E, Tin(2), Twi, Zfh-1, Eve, Eya, Hbr, Mef2, Pnr

Delta, En, Hh, Med, Pyr, Su\_H\_CSL, Ths, Ci, Htl, Nicd, Brk, Mef2, Srp(2)

Med, Pyr, Su\_H\_CSL, Ths, Wg, Da, Htl, Pan, Slp, Twi(2), Zfh-1, Brk, D-six4, Eya, Hbr, Mef2, Poxm

### Genotype: Med\_1; Tin\_0

Delta, Dpp, En, Hh, Med, Pyr, Shn, Su\_H\_CSL, Ths, Ci, Htl, Mad, Nicd

Delta, Dpp, Med, Pyr, Shn, Spi, Su\_H\_CSL, Ths, Upd, Wg, Doc, E\_Spl, Htl, Mad, Nicd, Pan, Slp, Stat92E, Twi, Zfh-1, Eya, Hbr

Delta, En, Hh, Med, Pyr, Su\_H\_CSL, Ths, Ci, Htl, Nicd, Brk, Srp(2)

Med, Pyr, Su\_H\_CSL, Ths, Wg, Da, Htl, Pan, Slp, Twi(2), Zfh-1, Brk, D-six4, Eya, Hbr, Mef2, Poxm

### Genotype: Med\_1; Tin\_2

Delta, Dpp, En, Hh, Med, Pyr, Shn, Su\_H\_CSL, Ths, Bap(3), Bin, Ci, E\_Spl, Htl, Mad, Nicd, Stat92E, Tin(2), Eya, Mef2

Delta, Dpp, Med, Pyr, Shn, Spi, Su\_H\_CSL, Ths, Upd, Wg, Doc, E\_Spl, Htl, Mad, Nicd, Pan, Slp, Stat92E, Tin(2), Twi, Zfh-1, Eve, Eya, Hbr, Mef2, Pnr

Delta, En, Hh, Med, Pyr, Su\_H\_CSL, Ths, Bap(2), Bin, Ci, E\_Spl, Htl, Nicd, Stat92E, Tin(2), Brk, D-six4, Eya, Mef2, Srp(2)

Med, Pyr, Su\_H\_CSL, Ths, Wg, Da, Htl, Pan, Slp, Stat92E, Tin(2), Twi(2), Zfh-1, Brk, D-six4, Eya, Hbr, Mef2, Poxm

### Genotype: Bap\_3; Doc\_1

Delta, Dpp, En, Hh, Med, Pyr, Shn, Su\_H\_CSL, Ths, Bap(3), Bin, Ci, Doc, Htl, Mad, Nicd, Tin, Mef2, Pnr

Delta, Dpp, Med, Pyr, Shn, Spi, Su\_H\_CSL, Ths, Upd, Wg, Bap(3), Bin, Doc, E\_Spl, Htl, Mad, Nicd, Pan, Slp, Stat92E, Tin(2), Twi, Zfh-1, Eve, Eya, Hbr, Mef2, Pnr

Delta, En, Hh, Med, Pyr, Su\_H\_CSL, Ths, Bap(3), Bin, Ci, Doc, Htl, Nicd, Brk, Srp(2)

Med, Pyr, Su\_H\_CSL, Ths, Wg, Bap(3), Bin, Da, Doc, Htl, Pan, Slp, Twi(2), Zfh-1, Brk, D-six4, Eya, Hbr, Mef2, Poxm

### Genotype: Bap\_0; Bin\_0

Delta, Dpp, En, Hh, Med, Pyr, Shn, Su\_H\_CSL, Ths, Ci, Htl, Mad, Nicd, Tin, Mef2

Delta, Dpp, Med, Pyr, Shn, Spi, Su\_H\_CSL, Ths, Upd, Wg, Doc, E\_Spl, Htl, Mad, Nicd, Pan, Slp, Stat92E, Tin(2), Twi, Zfh-1, Eve, Eya, Hbr, Mef2, Pnr

Delta, En, Hh, Med, Pyr, Su\_H\_CSL, Ths, Ci, Htl, Nicd, Brk, Srp(2)

Med, Pyr, Su\_H\_CSL, Ths, Wg, Da, Htl, Pan, Slp, Twi(2), Zfh-1, Brk, D-six4, Eya, Hbr, Mef2, Poxm

### Genotype: Bap\_0; Bin\_2

Delta, Dpp, En, Hh, Med, Pyr, Shn, Su\_H\_CSL, Ths, Bin(2), Ci, Htl, Mad, Nicd, Tin, Mef2

Delta, Dpp, Med, Pyr, Shn, Spi, Su\_H\_CSL, Ths, Upd, Wg, Bin(2), Doc, E\_Spl, Htl, Mad, Nicd, Pan, Slp, Stat92E, Tin(2), Twi, Zfh-1, Eve, Eya, Hbr, Mef2, Pnr

Delta, En, Hh, Med, Pyr, Su\_H\_CSL, Ths, Bin(2), Ci, Htl, Nicd, Brk, Srp(2)

Med, Pyr, Su\_H\_CSL, Ths, Wg, Bin(2), Da, Htl, Pan, Slp, Twi(2), Zfh-1, Brk, D-six4, Eya, Hbr, Mef2, Poxm

### Genotype: Tin\_2

Delta, Dpp, En, Hh, Med, Pyr, Shn, Su\_H\_CSL, Ths, Bap(3), Bin, Ci, E\_Spl, Htl, Mad, Nicd, Stat92E, Tin(2), Eya, Mef2

Delta, Dpp, Med, Pyr, Shn, Spi, Su\_H\_CSL, Ths, Upd, Wg, Doc, E\_Spl, Htl, Mad, Nicd, Pan, Slp, Stat92E, Tin(2), Twi, Zfh-1, Eve, Eya, Hbr, Mef2, Pnr

Delta, En, Hh, Med, Pyr, Su\_H\_CSL, Ths, Bap(2), Bin, Ci, E\_Spl, Htl, Nicd, Stat92E, Tin(2), Brk, D-six4, Eya, Mef2, Srp(2)

Med, Pyr, Su\_H\_CSL, Ths, Wg, Da, Htl, Pan, Slp, Stat92E, Tin(2), Twi(2), Zfh-1, Brk, D-six4, Eya, Hbr, Mef2, Poxm

### Genotype: Slp\_1; Twi\_0

Delta, Dpp, Hh, Med, Pyr, Shn, Su\_H\_CSL, Ths, Bap, Ci, Htl, Mad, Nicd, Slp, Tin, Mef2

Delta, Dpp, Med, Pyr, Shn, Spi, Su\_H\_CSL, Ths, Upd, Wg, Doc, E\_Spl, Htl, Mad, Nicd, Pan, Slp, Stat92E, Tin(2), Eya, Hbr, Mef2, Pnr

Delta, Hh, Med, Pyr, Su\_H\_CSL, Ths, Ci, Htl, Nicd, Slp, Brk, Srp

Med, Pyr, Su\_H\_CSL, Ths, Wg, Da, Htl, Pan, Slp, Brk, Hbr

### Genotype: Slp\_1; Twi\_2

Delta, Dpp, Hh, Med, Pyr, Shn, Su\_H\_CSL, Ths, Bap, Ci, Htl, Mad, Nicd, Slp, Tin, Twi(2), Zfh-1, Eya, Mef2

Delta, Dpp, Med, Pyr, Shn, Spi, Su\_H\_CSL, Ths, Upd, Wg, Doc, E\_Spl, Htl, Mad, Nicd, Pan, Slp, Stat92E, Tin(2), Twi(2), Zfh-1, Eve, Eya, Hbr, Mef2, Pnr

Delta, Hh, Med, Pyr, Su\_H\_CSL, Ths, Ci, Htl, Nicd, Slp, Twi(2), Zfh-1, Brk, D-six4, Eya, Mef2, Srp

Med, Pyr, Su\_H\_CSL, Ths, Wg, Da, Htl, Pan, Slp, Twi(2), Zfh-1, Brk, D-six4, Eya, Hbr, Mef2, Poxm

### Genotype: Doc\_1; Twi\_0

Delta, Dpp, En, Hh, Med, Pyr, Shn, Su\_H\_CSL, Ths, Bap(3), Bin, Ci, Doc, Htl, Mad, Nicd, Tin, Mef2, Pnr

Delta, Dpp, Med, Pyr, Shn, Spi, Su\_H\_CSL, Ths, Upd, Wg, Doc, E\_Spl, Htl, Mad, Nicd, Pan, Slp, Stat92E, Tin(2), Eya, Hbr, Mef2, Pnr

Delta, En, Hh, Med, Pyr, Su\_H\_CSL, Ths, Ci, Doc, Htl, Nicd, Brk, Srp(2)

Med, Pyr, Su\_H\_CSL, Ths, Wg, Da, Doc, Htl, Pan, Slp, Brk, Hbr

### Genotype: Bin\_0

Delta, Dpp, En, Hh, Med, Pyr, Shn, Su\_H\_CSL, Ths, Bap(3), Ci, Htl, Mad, Nicd, Tin, Mef2

Delta, Dpp, Med, Pyr, Shn, Spi, Su\_H\_CSL, Ths, Upd, Wg, Doc, E\_Spl, Htl, Mad, Nicd, Pan, Slp, Stat92E, Tin(2), Twi, Zfh-1, Eve, Eya, Hbr, Mef2, Pnr

Delta, En, Hh, Med, Pyr, Su\_H\_CSL, Ths, Ci, Htl, Nicd, Brk, Srp(2)

Med, Pyr, Su\_H\_CSL, Ths, Wg, Da, Htl, Pan, Slp, Twi(2), Zfh-1, Brk, D-six4, Eya, Hbr, Mef2, Poxm

### Genotype: Doc\_1; Twi\_2

Delta, Dpp, En, Hh, Med, Pyr, Shn, Su\_H\_CSL, Ths, Bap(3), Bin, Ci, Doc, Htl, Mad, Nicd, Tin, Twi(2), Zfh-1, Eya, Mef2, Pnr

Delta, Dpp, Med, Pyr, Shn, Spi, Su\_H\_CSL, Ths, Upd, Wg, Doc, E\_Spl, Htl, Mad, Nicd, Pan, Slp, Stat92E, Tin(2), Twi(2), Zfh-1, Eve, Eya, Hbr, Mef2, Pnr

Delta, En, Hh, Med, Pyr, Su\_H\_CSL, Ths, Ci, Doc, Htl, Nicd, Twi(2), Zfh-1, Brk, D-six4, Eya, Mef2, Srp(2)

Med, Pyr, Su\_H\_CSL, Ths, Wg, Da, Doc, Htl, Pan, Slp, Twi(2), Zfh-1, Brk, D-six4, Eya, Hbr, Mef2, Poxm

### Genotype: Mef2\_0

Delta, Dpp, En, Hh, Med, Pyr, Shn, Su\_H\_CSL, Ths, Bap(3), Bin, Ci, Htl, Mad, Nicd, Tin

Delta, Dpp, Med, Pyr, Shn, Spi, Su\_H\_CSL, Ths, Upd, Wg, Doc, E\_Spl, Htl, Mad, Nicd, Pan, Slp, Stat92E, Tin(2), Twi, Zfh-1, Eve, Eya, Hbr, Pnr

Delta, En, Hh, Med, Pyr, Su\_H\_CSL, Ths, Ci, Htl, Nicd, Brk, Srp(2)

Med, Pyr, Su\_H\_CSL, Ths, Wg, Da, Htl, Pan, Slp, Twi(2), Zfh-1, Brk, D-six4, Eya, Hbr, Poxm

### Genotype: Ci\_0; Pan\_0

Delta, Dpp, En, Hh, Med, Pyr, Shn, Su\_H\_CSL, Ths, Bap, Htl, Mad, Nicd, Tin, Mef2

Delta, Dpp, En, Hh, Med, Pyr, Shn, Su\_H\_CSL, Ths, Bap(2), Bin, Htl, Mad, Nicd, Tin, Mef2

Delta, Dpp, Med, Pyr, Shn, Spi, Su\_H\_CSL, Ths, Upd, Wg, Bap, E\_Spl, Htl, Mad, Nicd, Stat92E, Tin, Mef2

Delta, En, Hh, Med, Pyr, Su\_H\_CSL, Ths, Htl, Nicd, Brk

Med, Pyr, Su\_H\_CSL, Ths, Wg, Da, Htl, Brk

Med, Pyr, Su\_H\_CSL, Ths, Wg, Da, Htl, Twi, Zfh-1, Brk, D-six4, Eya

### Genotype: Ci\_0; Pan\_1

Delta, Dpp, En, Hh, Med, Pyr, Shn, Su\_H\_CSL, Ths, Doc, Htl, Mad, Nicd, Pan, Slp, Tin, Hbr, Mef2, Pnr

Delta, Dpp, En, Hh, Med, Pyr, Shn, Su\_H\_CSL, Ths, Doc, Htl, Mad, Nicd, Pan, Slp, Tin, Twi, Zfh-1, Eya, Hbr, Mef2, Pnr

Delta, Dpp, Med, Pyr, Shn, Spi, Su\_H\_CSL, Ths, Upd, Wg, Doc, E\_Spl, Htl, Mad, Nicd, Pan, Slp, Stat92E, Tin(2), Twi, Zfh-1, Eve, Eya, Hbr, Mef2, Pnr

Delta, En, Hh, Med, Pyr, Su\_H\_CSL, Ths, Htl, Nicd, Pan, Slp, Brk, Hbr

Delta, En, Hh, Med, Pyr, Su\_H\_CSL, Ths, Htl, Nicd, Pan, Slp, Twi, Zfh-1, Brk, D-six4, Eya, Hbr, Poxm

Med, Pyr, Su\_H\_CSL, Ths, Wg, Da, Htl, Pan, Slp, Twi(2), Zfh-1, Brk, D-six4, Eya, Hbr, Mef2, Poxm

### Genotype: Ci\_1; Doc\_0

Delta, Dpp, En, Hh, Med, Pyr, Shn, Su\_H\_CSL, Ths, Bap(3), Bin, Ci, Htl, Mad, Nicd, Tin, Mef2

Delta, Dpp, Med, Pyr, Shn, Spi, Su\_H\_CSL, Ths, Upd, Wg, Bap, Ci, E\_Spl, Htl, Mad, Nicd, Pan, Slp, Stat92E, Tin(2), Twi, Zfh-1, Eve, Eya, Hbr, Mef2

Delta, En, Hh, Med, Pyr, Su\_H\_CSL, Ths, Ci, Htl, Nicd, Brk, Srp(2)

Med, Pyr, Su\_H\_CSL, Ths, Wg, Ci, Da, Htl, Pan, Slp, Twi(2), Zfh-1, Brk, D-six4, Eya, Hbr, Mef2, Poxm, Srp

### Genotype: Ci\_1; Doc\_1

Delta, Dpp, En, Hh, Med, Pyr, Shn, Su\_H\_CSL, Ths, Bap(3), Bin, Ci, Doc, Htl, Mad, Nicd, Tin, Mef2, Pnr

Delta, Dpp, Med, Pyr, Shn, Spi, Su\_H\_CSL, Ths, Upd, Wg, Bap, Ci, Doc, E\_Spl, Htl, Mad, Nicd, Pan, Slp, Stat92E, Tin(2), Twi, Zfh-1, Eve, Eya, Hbr, Mef2, Pnr

Delta, En, Hh, Med, Pyr, Su\_H\_CSL, Ths, Ci, Doc, Htl, Nicd, Brk, Srp(2)

Med, Pyr, Su\_H\_CSL, Ths, Wg, Ci, Da, Doc, Htl, Pan, Slp, Twi(2), Zfh-1, Brk, D-six4, Eya, Hbr, Mef2, Poxm, Srp

### Genotype: Mef2\_0; Twi\_2

Delta, Dpp, En, Hh, Med, Pyr, Shn, Su\_H\_CSL, Ths, Bap(3), Bin, Ci, Htl, Mad, Nicd, Tin, Twi(2), Zfh-1, Eya

Delta, Dpp, Med, Pyr, Shn, Spi, Su\_H\_CSL, Ths, Upd, Wg, Doc, E\_Spl, Htl, Mad, Nicd, Pan, Slp, Stat92E, Tin(2), Twi(2), Zfh-1, Eve, Eya, Hbr, Pnr

Delta, En, Hh, Med, Pyr, Su\_H\_CSL, Ths, Ci, Htl, Nicd, Twi(2), Zfh-1, Brk, D-six4, Eya, Srp(2)

Med, Pyr, Su\_H\_CSL, Ths, Wg, Da, Htl, Pan, Slp, Twi(2), Zfh-1, Brk, D-six4, Eya, Hbr, Poxm

### Genotype: Med\_0

Delta, Dpp, En, Hh, Pyr, Shn, Su\_H\_CSL, Ths, Bap(2), Bin, Ci, Htl, Mad, Nicd, Tin, Brk, D-six4, Mef2, Srp(2)

Delta, Dpp, Pyr, Shn, Spi, Su\_H\_CSL, Ths, Upd, Wg, E\_Spl, Htl, Mad, Nicd, Pan, Slp, Stat92E, Tin, Twi, Zfh-1, Brk, D-six4, Eya, Hbr, Mef2, Poxm

Delta, En, Hh, Pyr, Su\_H\_CSL, Ths, Ci, Htl, Nicd, Brk, Srp(2)

Pyr, Su\_H\_CSL, Ths, Wg, Da, Htl, Pan, Slp, Twi(2), Zfh-1, Brk, D-six4, Eya, Hbr, Mef2, Poxm

### Genotype: Mef2\_0; Twi\_0

Delta, Dpp, En, Hh, Med, Pyr, Shn, Su\_H\_CSL, Ths, Bap(3), Bin, Ci, Htl, Mad, Nicd, Tin

Delta, Dpp, Med, Pyr, Shn, Spi, Su\_H\_CSL, Ths, Upd, Wg, Doc, E\_Spl, Htl, Mad, Nicd, Pan, Slp, Stat92E, Tin(2), Eya, Hbr, Pnr

Delta, En, Hh, Med, Pyr, Su\_H\_CSL, Ths, Ci, Htl, Nicd, Brk, Srp(2)

Med, Pyr, Su\_H\_CSL, Ths, Wg, Da, Htl, Pan, Slp, Brk, Hbr

### Genotype: Pan\_1; Srp\_2

Delta, Dpp, En, Hh, Med, Pyr, Shn, Su\_H\_CSL, Ths, Bap, Ci, Doc, Htl, Mad, Nicd, Pan, Slp, Tin, Hbr, Mef2, Pnr, Srp(2)

Delta, Dpp, En, Hh, Med, Pyr, Shn, Su\_H\_CSL, Ths, Bap, Ci, Doc, Htl, Mad, Nicd, Pan, Slp, Tin, Twi, Zfh-1, Eya, Hbr, Mef2, Pnr, Srp(2)

Delta, Dpp, Med, Pyr, Shn, Spi, Su\_H\_CSL, Ths, Upd, Wg, Doc, E\_Spl, Htl, Mad, Nicd, Pan, Slp, Stat92E, Tin(2), Twi, Zfh-1, Eve, Eya, Hbr, Mef2, Pnr, Srp(2)

Delta, En, Hh, Med, Pyr, Su\_H\_CSL, Ths, Ci, Htl, Nicd, Pan, Slp, Brk, Hbr, Srp(2)

Delta, En, Hh, Med, Pyr, Su\_H\_CSL, Ths, Ci, Htl, Nicd, Pan, Slp, Twi, Zfh-1, Brk, D-six4, Eya, Hbr, Poxm, Srp(2)

Med, Pyr, Su\_H\_CSL, Ths, Wg, Da, Htl, Pan, Slp, Twi(2), Zfh-1, Brk, D-six4, Eya, Hbr, Mef2, Poxm, Srp(2)

### Genotype: Pan\_1; Srp\_0

Delta, Dpp, En, Hh, Med, Pyr, Shn, Su\_H\_CSL, Ths, Bap, Ci, Doc, Htl, Mad, Nicd, Pan, Slp, Tin, Hbr, Mef2, Pnr

Delta, Dpp, En, Hh, Med, Pyr, Shn, Su\_H\_CSL, Ths, Bap, Ci, Doc, Htl, Mad, Nicd, Pan, Slp, Tin, Twi, Zfh-1, Eya, Hbr, Mef2, Pnr

Delta, Dpp, Med, Pyr, Shn, Spi, Su\_H\_CSL, Ths, Upd, Wg, Doc, E\_Spl, Htl, Mad, Nicd, Pan, Slp, Stat92E, Tin(2), Twi, Zfh-1, Eve, Eya, Hbr, Mef2, Pnr

Delta, En, Hh, Med, Pyr, Su\_H\_CSL, Ths, Ci, Htl, Nicd, Pan, Slp, Brk, Hbr

Delta, En, Hh, Med, Pyr, Su\_H\_CSL, Ths, Ci, Htl, Nicd, Pan, Slp, Twi, Zfh-1, Brk, D-six4, Eya, Hbr, Poxm

Med, Pyr, Su\_H\_CSL, Ths, Wg, Da, Htl, Pan, Slp, Twi(2), Zfh-1, Brk, D-six4, Eya, Hbr, Mef2, Poxm

### Genotype: Twi\_0

Delta, Dpp, En, Hh, Med, Pyr, Shn, Su\_H\_CSL, Ths, Bap(3), Bin, Ci, Htl, Mad, Nicd, Tin, Mef2

Delta, Dpp, Med, Pyr, Shn, Spi, Su\_H\_CSL, Ths, Upd, Wg, Doc, E\_Spl, Htl, Mad, Nicd, Pan, Slp, Stat92E, Tin(2), Eya, Hbr, Mef2, Pnr

Delta, En, Hh, Med, Pyr, Su\_H\_CSL, Ths, Ci, Htl, Nicd, Brk, Srp(2)

Med, Pyr, Su\_H\_CSL, Ths, Wg, Da, Htl, Pan, Slp, Brk, Hbr

### Genotype: Bap\_0; Twi\_2

Delta, Dpp, En, Hh, Med, Pyr, Shn, Su\_H\_CSL, Ths, Ci, Htl, Mad, Nicd, Tin, Twi(2), Zfh-1, Eya, Mef2

Delta, Dpp, Med, Pyr, Shn, Spi, Su\_H\_CSL, Ths, Upd, Wg, Doc, E\_Spl, Htl, Mad, Nicd, Pan, Slp, Stat92E, Tin(2), Twi(2), Zfh-1, Eve, Eya, Hbr, Mef2, Pnr

Delta, En, Hh, Med, Pyr, Su\_H\_CSL, Ths, Ci, Htl, Nicd, Twi(2), Zfh-1, Brk, D-six4, Eya, Mef2, Srp(2)

Med, Pyr, Su\_H\_CSL, Ths, Wg, Da, Htl, Pan, Slp, Twi(2), Zfh-1, Brk, D-six4, Eya, Hbr, Mef2, Poxm

### Genotype: Bap\_0; Twi\_0

Delta, Dpp, En, Hh, Med, Pyr, Shn, Su\_H\_CSL, Ths, Ci, Htl, Mad, Nicd, Tin, Mef2

Delta, Dpp, Med, Pyr, Shn, Spi, Su\_H\_CSL, Ths, Upd, Wg, Doc, E\_Spl, Htl, Mad, Nicd, Pan, Slp, Stat92E, Tin(2), Eya, Hbr, Mef2, Pnr

Delta, En, Hh, Med, Pyr, Su\_H\_CSL, Ths, Ci, Htl, Nicd, Brk, Srp(2)

Med, Pyr, Su\_H\_CSL, Ths, Wg, Da, Htl, Pan, Slp, Brk, Hbr

### Genotype: Mad\_1; Pan\_0

Delta, Dpp, En, Hh, Med, Pyr, Shn, Su\_H\_CSL, Ths, Bap(3), Bin, Ci, Htl, Mad, Nicd, Tin, Mef2

Delta, Dpp, Med, Pyr, Shn, Spi, Su\_H\_CSL, Ths, Upd, Wg, Bap, E\_Spl, Htl, Mad, Nicd, Stat92E, Tin, Mef2

Delta, En, Hh, Med, Pyr, Su\_H\_CSL, Ths, Bap(3), Bin, Ci, Htl, Mad, Nicd, Tin, Brk, Mef2

Med, Pyr, Su\_H\_CSL, Ths, Wg, Bap, Da, Htl, Mad, Tin, Brk, Mef2

Med, Pyr, Su\_H\_CSL, Ths, Wg, Bap, Da, Htl, Mad, Tin, Twi, Zfh-1, Brk, Eya, Mef2

### Genotype: Mad\_1; Pan\_1

Delta, Dpp, En, Hh, Med, Pyr, Shn, Su\_H\_CSL, Ths, Bap, Ci, Doc, Htl, Mad, Nicd, Pan, Slp, Tin, Hbr, Mef2, Pnr

Delta, Dpp, En, Hh, Med, Pyr, Shn, Su\_H\_CSL, Ths, Bap, Ci, Doc, Htl, Mad, Nicd, Pan, Slp, Tin, Twi, Zfh-1, Eya, Hbr, Mef2, Pnr

Delta, Dpp, Med, Pyr, Shn, Spi, Su\_H\_CSL, Ths, Upd, Wg, Doc, E\_Spl, Htl, Mad, Nicd, Pan, Slp, Stat92E, Tin(2), Twi, Zfh-1, Eve, Eya, Hbr, Mef2, Pnr

Delta, En, Hh, Med, Pyr, Su\_H\_CSL, Ths, Bap, Ci, Doc, Htl, Mad, Nicd, Pan, Slp, Tin, Brk, Hbr, Mef2, Pnr

Delta, En, Hh, Med, Pyr, Su\_H\_CSL, Ths, Bap, Ci, Doc, Htl, Mad, Nicd, Pan, Slp, Tin, Twi, Zfh-1, Brk, Eya, Hbr, Mef2, Pnr

Med, Pyr, Su\_H\_CSL, Ths, Wg, Da, Doc, Htl, Mad, Pan, Slp, Tin, Twi(2), Zfh-1, Brk, Eya, Hbr, Mef2, Pnr

### Genotype: Slp\_0

Delta, Dpp, En, Hh, Med, Pyr, Shn, Su\_H\_CSL, Ths, Bap(3), Bin, Ci, Htl, Mad, Nicd, Tin, Mef2

Delta, Dpp, En, Med, Pyr, Shn, Spi, Su\_H\_CSL, Ths, Upd, Wg, Bap, Doc, E\_Spl, Htl, Mad, Nicd, Pan, Stat92E, Tin(2), Eya, Hbr, Mef2, Pnr

Delta, En, Hh, Med, Pyr, Su\_H\_CSL, Ths, Ci, Htl, Nicd, Brk, Srp(2)

En, Med, Pyr, Su\_H\_CSL, Ths, Wg, Da, Htl, Pan, Brk, Hbr

En, Med, Pyr, Su\_H\_CSL, Ths, Wg, Da, Htl, Pan, Twi, Zfh-1, Brk, D-six4, Eya, Hbr, Poxm

### Genotype: Ci\_1; Twi\_2

Delta, Dpp, En, Hh, Med, Pyr, Shn, Su\_H\_CSL, Ths, Bap(3), Bin, Ci, Htl, Mad, Nicd, Tin, Twi(2), Zfh-1, Eya, Mef2

Delta, Dpp, Med, Pyr, Shn, Spi, Su\_H\_CSL, Ths, Upd, Wg, Bap, Ci, Doc, E\_Spl, Htl, Mad, Nicd, Pan, Slp, Stat92E, Tin(2), Twi(2), Zfh-1, Eve, Eya, Hbr, Mef2, Pnr

Delta, En, Hh, Med, Pyr, Su\_H\_CSL, Ths, Ci, Htl, Nicd, Twi(2), Zfh-1, Brk, D-six4, Eya, Mef2, Srp(2)

Med, Pyr, Su\_H\_CSL, Ths, Wg, Ci, Da, Htl, Pan, Slp, Twi(2), Zfh-1, Brk, D-six4, Eya, Hbr, Mef2, Poxm, Srp

### Genotype: Ci\_1; Twi\_0

Delta, Dpp, En, Hh, Med, Pyr, Shn, Su\_H\_CSL, Ths, Bap(3), Bin, Ci, Htl, Mad, Nicd, Tin, Mef2

Delta, Dpp, Med, Pyr, Shn, Spi, Su\_H\_CSL, Ths, Upd, Wg, Bap, Ci, Doc, E\_Spl, Htl, Mad, Nicd, Pan, Slp, Stat92E, Tin(2), Eya, Hbr, Mef2, Pnr

Delta, En, Hh, Med, Pyr, Su\_H\_CSL, Ths, Ci, Htl, Nicd, Brk, Srp(2)

Med, Pyr, Su\_H\_CSL, Ths, Wg, Ci, Da, Htl, Pan, Slp, Brk, Hbr, Srp

Bap\_0

Bap\_0

Bap\_0

Bap\_0

Bap\_3

Bap\_3

Bap\_3

Bap\_3

Bin\_0

Bin\_0

Bin\_0

Bin\_0

Bin\_2

Bin\_2

Bin\_2

Bin\_2

Ci\_0

Ci\_0

Ci\_0

Ci\_0

Ci\_1

Ci\_1

Ci\_1

Ci\_1

Doc\_0

Doc\_0

Doc\_0

Doc\_0

Doc\_1

Doc\_1

Doc\_1

Doc\_1

Mad\_0

Mad\_0

Mad\_0

Mad\_0

Mad\_1

Mad\_1

Mad\_1

Mad\_1

Med\_0

Med\_0

Med\_0

Med\_0

Med\_1

Med\_1

Med\_1

Med\_1

Mef2\_0

Mef2\_0

Mef2\_0

Mef2\_0

Mef2\_1

Mef2\_1

Mef2\_1

Mef2\_1

Nicd\_0

Nicd\_0

Nicd\_0

Nicd\_0

Nicd\_1

Nicd\_1

Nicd\_1

Nicd\_1

Pan\_0

Pan\_0

Pan\_0

Pan\_0

Pan\_1

Pan\_1

Pan\_1

Pan\_1

Pnr\_0

Pnr\_0

Pnr\_0

Pnr\_0

Pnr\_1

Pnr\_1

Pnr\_1

Pnr\_1

Slp\_0

Slp\_0

Slp\_0

Slp\_0

Slp\_1

Slp\_1

Slp\_1

Slp\_1

Srp\_0

Srp\_0

Srp\_0

Srp\_0

Srp\_2

Srp\_2

Srp\_2

Srp\_2

Tin\_0

Tin\_0

Tin\_0

Tin\_0

Tin\_2

Tin\_2

Tin\_2

Tin\_2

Twi\_0

Twi\_0

Twi\_0

Twi\_0

Twi\_2

Twi\_2

Twi\_2

Twi\_2
