# Supplementary material for: Qualitative Dynamical Modelling Can Formally Explain Mesoderm Specification and Predict Novel Developmental Phenotypes
Source: PLoS Comput Biol. 2016 Sep 6;12(9):e1005073. doi: 10.1371/journal.pcbi.1005073 (PMC5012701; doi:10.1371/journal.pcbi.1005073)
Supplement: S2 File — The content of this supporting web archive folder documents known and novel gene expression pattern simulated with the drosophila mesoderm specification. Open the file “index.html” with a web browser to access this information. (ZIP) [file pcbi.1005073.s008.zip › SupWebArchive_W1/model.html]

Drosophila Mesoderm Model


- About
- Model
- Selected simulations
- Perturbation matrix

# Regulatory graph of the signalling/transcriptional network controlling drosophila mesoderm specification.

Built with the software GINsim (http://ginsim.org), this regulatory graph encompasses the main regulatory factors and interactions involved in mesoderm specification (stages 8-10), as documented by published (molecular) genetic data and functional genomic data. Ellipses denote Boolean nodes, whereas rectangles denote multilevel nodes. Light green filling denote input nodes, most corresponding to factors expressed in and acting from the ectoderm. Yellow filling denote output factors, mostly effector genes and tissue markers. Blue, and grey filling represent internal nodes expressed in the mesoderm. Green arrows and red blunt arrows denote activations and inhibitions, respectively. Logical rules are further associated with each node to define its behaviour depending on regulatory inputs.
