# Supplementary material for: Qualitative Dynamical Modelling Can Formally Explain Mesoderm Specification and Predict Novel Developmental Phenotypes
Source: PLoS Comput Biol. 2016 Sep 6;12(9):e1005073. doi: 10.1371/journal.pcbi.1005073 (PMC5012701; doi:10.1371/journal.pcbi.1005073)
Supplement: S2 File — The content of this supporting web archive folder documents known and novel gene expression pattern simulated with the drosophila mesoderm specification. Open the file “index.html” with a web browser to access this information. (ZIP) [file pcbi.1005073.s008.zip › SupWebArchive_W1/simulations.html]

Drosophila Mesoderm Model


- About
- Model
- Selected simulations
- Perturbation matrix

# Simulations of WT and known genetic perturbations

The results of the simulation of the wild-type situation and of seven know genetic perturbations are shown in the form of coloured square vignettes. The first vignette (top left) correspond to the wild type situation, with VM, H, FB and SM presumptive territories coloured in blue, red, green and orange, respectively. In the following vignettes, the coloration of the four presumptive territories are modified to reflect the absence of important markers, or the combination of markers associated with different tissues (cf. coulour key). Wg loss-of-function (lof) leads to the loss of Wg/Slp domain, resulting in an expansion of the En/Hh domain; consequently, the model correctly predicts the loss of H along with a potential perturbation of SM (yellow domains). Dpp lof leads to the loss of dorsal derivatives (VM and H), along with ann expansion of FB. Dpp gain-of-funciton (gof) leads to an expansion of VM at the expense of FB, along with a perturbation of SM. Tin lof shows a loss of dorsal tissues, while Bap lof exhibits only the loss of VM. Finally, the combination of Wg gof and Hh lof leads to a dorsal expansion of H, along with a loss of FB, while the combination of Dpp gof, Hh gof and Wg lof leads to an expansion of VM in the whole mesoderm.

## Cell types

|  |  |
| --- | --- |
| Visceral Mesoderm | Heart |
| Fat Body | Somatic Muscle |

## Phenotype keys

|  |  |  |  |  |
| --- | --- | --- | --- | --- |
| VM     VM (expected) | H     H (expected) | FB     FB (expected) | SM     SM (expected) | Unknown |

## Simulations

Back


WT

### Genotype: WT

Delta, Dpp, En, Hh, Med, Pyr, Shn, Su\_H\_CSL, Ths, Bap(3), Bin, Ci, Htl, Mad, Nicd, Tin, Mef2

Delta, Dpp, Med, Pyr, Shn, Spi, Su\_H\_CSL, Ths, Upd, Wg, Doc, E\_Spl, Htl, Mad, Nicd, Pan, Slp, Stat92E, Tin(2), Twi, Zfh-1, Eve, Eya, Hbr, Mef2, Pnr

Delta, En, Hh, Med, Pyr, Su\_H\_CSL, Ths, Ci, Htl, Nicd, Brk, Srp(2)

Med, Pyr, Su\_H\_CSL, Ths, Wg, Da, Htl, Pan, Slp, Twi(2), Zfh-1, Brk, D-six4, Eya, Hbr, Mef2, Poxm

Wg\_0

### Genotype: Wg\_0

Delta, Dpp, En, Hh, Med, Pyr, Shn, Su\_H\_CSL, Ths, Bap(3), Bin, Ci, Htl, Mad, Nicd, Tin, Mef2

Delta, Dpp, Med, Pyr, Shn, Spi, Su\_H\_CSL, Ths, Upd, Bap, E\_Spl, Htl, Mad, Nicd, Stat92E, Tin, Mef2

Delta, En, Hh, Med, Pyr, Su\_H\_CSL, Ths, Ci, Htl, Nicd, Brk, Srp(2)

Med, Pyr, Su\_H\_CSL, Ths, Da, Htl, Twi, Zfh-1, Brk, D-six4, Eya

Med, Pyr, Su\_H\_CSL, Ths, Da, Htl, Brk

Dpp\_0

### Genotype: Dpp\_0

Delta, En, Hh, Med, Pyr, Shn, Su\_H\_CSL, Ths, Ci, Htl, Nicd, Brk, Srp(2)

Delta, Med, Pyr, Shn, Spi, Su\_H\_CSL, Ths, Upd, Wg, E\_Spl, Htl, Nicd, Pan, Slp, Stat92E, Twi, Zfh-1, Brk, D-six4, Eya, Hbr, Poxm

Delta, En, Hh, Med, Pyr, Su\_H\_CSL, Ths, Ci, Htl, Nicd, Brk, Srp(2)

Med, Pyr, Su\_H\_CSL, Ths, Wg, Da, Htl, Pan, Slp, Twi(2), Zfh-1, Brk, D-six4, Eya, Hbr, Mef2, Poxm

Dpp\_1

### Genotype: Dpp\_1

Delta, Dpp, En, Hh, Med, Pyr, Shn, Su\_H\_CSL, Ths, Bap(3), Bin, Ci, Htl, Mad, Nicd, Tin, Mef2

Delta, Dpp, Med, Pyr, Shn, Spi, Su\_H\_CSL, Ths, Upd, Wg, Doc, E\_Spl, Htl, Mad, Nicd, Pan, Slp, Stat92E, Tin(2), Twi, Zfh-1, Eve, Eya, Hbr, Mef2, Pnr

Delta, Dpp, En, Hh, Med, Pyr, Su\_H\_CSL, Ths, Bap(3), Bin, Ci, Htl, Mad, Nicd, Tin, Brk, Mef2

Dpp, Med, Pyr, Su\_H\_CSL, Ths, Wg, Da, Doc, Htl, Mad, Pan, Slp, Tin, Twi(2), Zfh-1, Brk, Eya, Hbr, Mef2, Pnr

Tin\_0

### Genotype: Tin\_0

Delta, Dpp, En, Hh, Med, Pyr, Shn, Su\_H\_CSL, Ths, Ci, Htl, Mad, Nicd

Delta, Dpp, Med, Pyr, Shn, Spi, Su\_H\_CSL, Ths, Upd, Wg, Doc, E\_Spl, Htl, Mad, Nicd, Pan, Slp, Stat92E, Twi, Zfh-1, Eya, Hbr

Delta, En, Hh, Med, Pyr, Su\_H\_CSL, Ths, Ci, Htl, Nicd, Brk, Srp(2)

Med, Pyr, Su\_H\_CSL, Ths, Wg, Da, Htl, Pan, Slp, Twi(2), Zfh-1, Brk, D-six4, Eya, Hbr, Mef2, Poxm

Bap\_0

### Genotype: Bap\_0

Delta, Dpp, En, Hh, Med, Pyr, Shn, Su\_H\_CSL, Ths, Ci, Htl, Mad, Nicd, Tin, Mef2

Delta, Dpp, Med, Pyr, Shn, Spi, Su\_H\_CSL, Ths, Upd, Wg, Doc, E\_Spl, Htl, Mad, Nicd, Pan, Slp, Stat92E, Tin(2), Twi, Zfh-1, Eve, Eya, Hbr, Mef2, Pnr

Delta, En, Hh, Med, Pyr, Su\_H\_CSL, Ths, Ci, Htl, Nicd, Brk, Srp(2)

Med, Pyr, Su\_H\_CSL, Ths, Wg, Da, Htl, Pan, Slp, Twi(2), Zfh-1, Brk, D-six4, Eya, Hbr, Mef2, Poxm

Wg\_1  
Hh\_0

### Genotype: Wg\_1; Hh\_0

Delta, Dpp, En, Med, Pyr, Shn, Su\_H\_CSL, Ths, Wg, Doc, Htl, Mad, Nicd, Pan, Slp, Tin, Hbr, Mef2, Pnr

Delta, Dpp, En, Med, Pyr, Shn, Su\_H\_CSL, Ths, Wg, Doc, Htl, Mad, Nicd, Pan, Slp, Tin, Twi, Zfh-1, Eya, Hbr, Mef2, Pnr

Delta, Dpp, Med, Pyr, Shn, Spi, Su\_H\_CSL, Ths, Upd, Wg, Doc, E\_Spl, Htl, Mad, Nicd, Pan, Slp, Stat92E, Tin(2), Twi, Zfh-1, Eve, Eya, Hbr, Mef2, Pnr

Delta, En, Med, Pyr, Su\_H\_CSL, Ths, Wg, Htl, Nicd, Pan, Slp, Brk, Hbr

Delta, En, Med, Pyr, Su\_H\_CSL, Ths, Wg, Htl, Nicd, Pan, Slp, Twi, Zfh-1, Brk, D-six4, Eya, Hbr, Poxm

Med, Pyr, Su\_H\_CSL, Ths, Wg, Da, Htl, Pan, Slp, Twi(2), Zfh-1, Brk, D-six4, Eya, Hbr, Mef2, Poxm

Dpp\_1  
Hh\_1  
Wg\_0


### Genotype: Dpp\_1; Hh\_1; Wg\_0

Delta, Dpp, En, Hh, Med, Pyr, Shn, Su\_H\_CSL, Ths, Bap(3), Bin, Ci, Htl, Mad, Nicd, Tin, Mef2

Delta, Dpp, En, Hh, Med, Pyr, Shn, Spi, Su\_H\_CSL, Ths, Upd, Bap(3), Bin, Ci, E\_Spl, Htl, Mad, Nicd, Stat92E, Tin, Mef2

Delta, Dpp, En, Hh, Med, Pyr, Su\_H\_CSL, Ths, Bap(3), Bin, Ci, Htl, Mad, Nicd, Tin, Brk, Mef2

Dpp, En, Hh, Med, Pyr, Su\_H\_CSL, Ths, Bap(3), Bin, Ci, Da, Htl, Mad, Tin, Twi, Zfh-1, Brk, Eya, Mef2

Dpp, En, Hh, Med, Pyr, Su\_H\_CSL, Ths, Bap(3), Bin, Ci, Da, Htl, Mad, Tin, Brk, Mef2
